# Supplementary material for: Biosynthesis of helvolic acid and identification of an unusual C-4-demethylation process distinct from sterol biosynthesis
Source: Nat Commun. 2017 Nov 21;8:1644. doi: 10.1038/s41467-017-01813-9 (PMC5696383; doi:10.1038/s41467-017-01813-9)
Supplement: Supplementary file 1 — Supplementary Information [file 41467_2017_1813_MOESM1_ESM.pdf]

Supplementary Figures

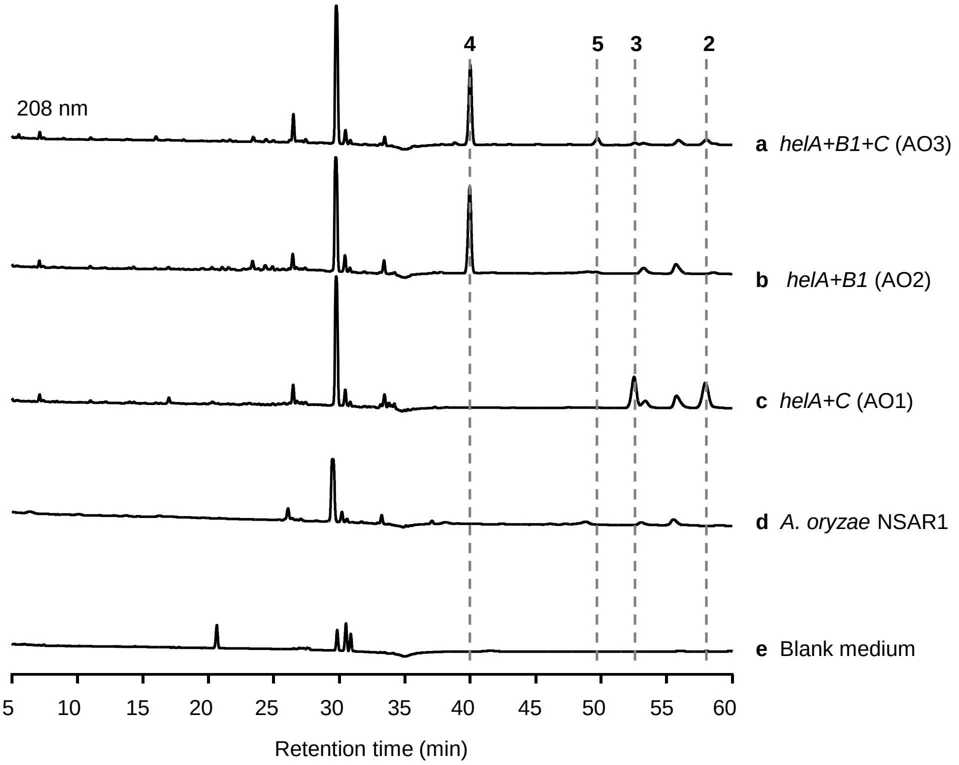

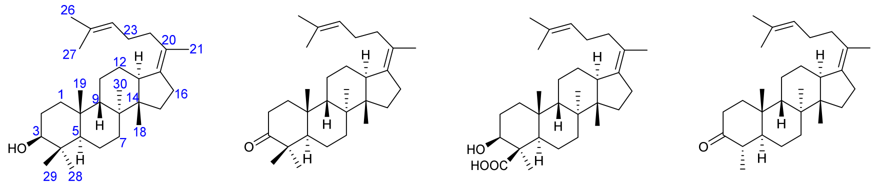

2 **3** **4** **5**

Supplementary Figure 1. HPLC analysis for identification of the C-4 demethylation triggered by HelB1 and HelC

a Mycelia extract of *A. oryzae* harboring *helA*, *helB1* and *helC*; **b** Mycelia extract of *A. oryzae* harboring *helA* and *helB1*; **c** Mycelia extract of *A. oryzae* harboring *helA* and *helC*; **d** Mycelia extract of *A. oryzae*; **e** Blank medium.

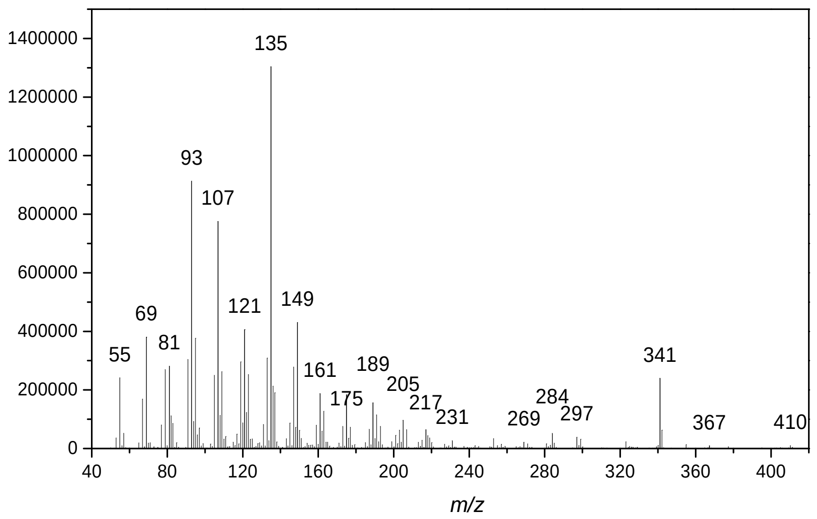

Supplementary Figure 2. Electron impact mass spectrum of 5 in AO3

The *A. oryzae* transformant AO3 harbors *helA*, *helB1* and *helC*.

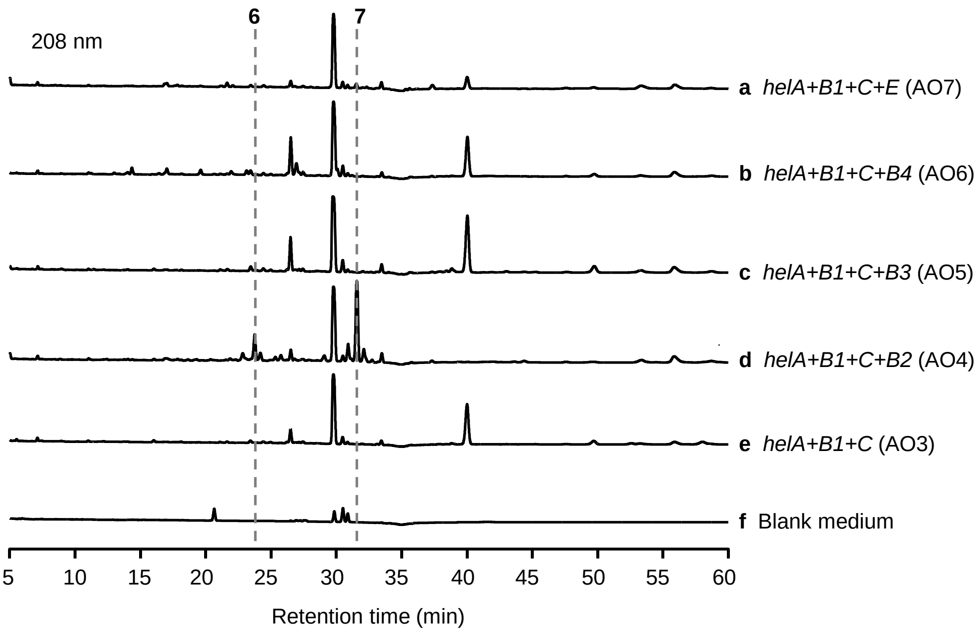

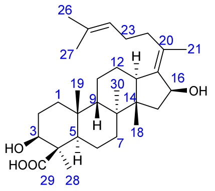

6

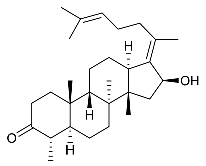

7

Supplementary Figure 3. HPLC analysis for identification of HelB2

a Mycelia extract of *A. oryzae* harboring *helA*, *helB1*, *helC* and *helE*; **b** Mycelia extract of *A. oryzae* harboring *helA*, *helB1*, *helC* and *helB4*; **c** Mycelia extract of *A. oryzae* harboring *helA*, *helB1*, *helC* and *helB3*; **d** Mycelia extract of *A. oryzae* harboring *helA*, *helB1*, *helC* and *helB2*; **e** Mycelia extract of *A. oryzae* harboring *helA*, *helB1* and *helC*; **f** Blank medium.

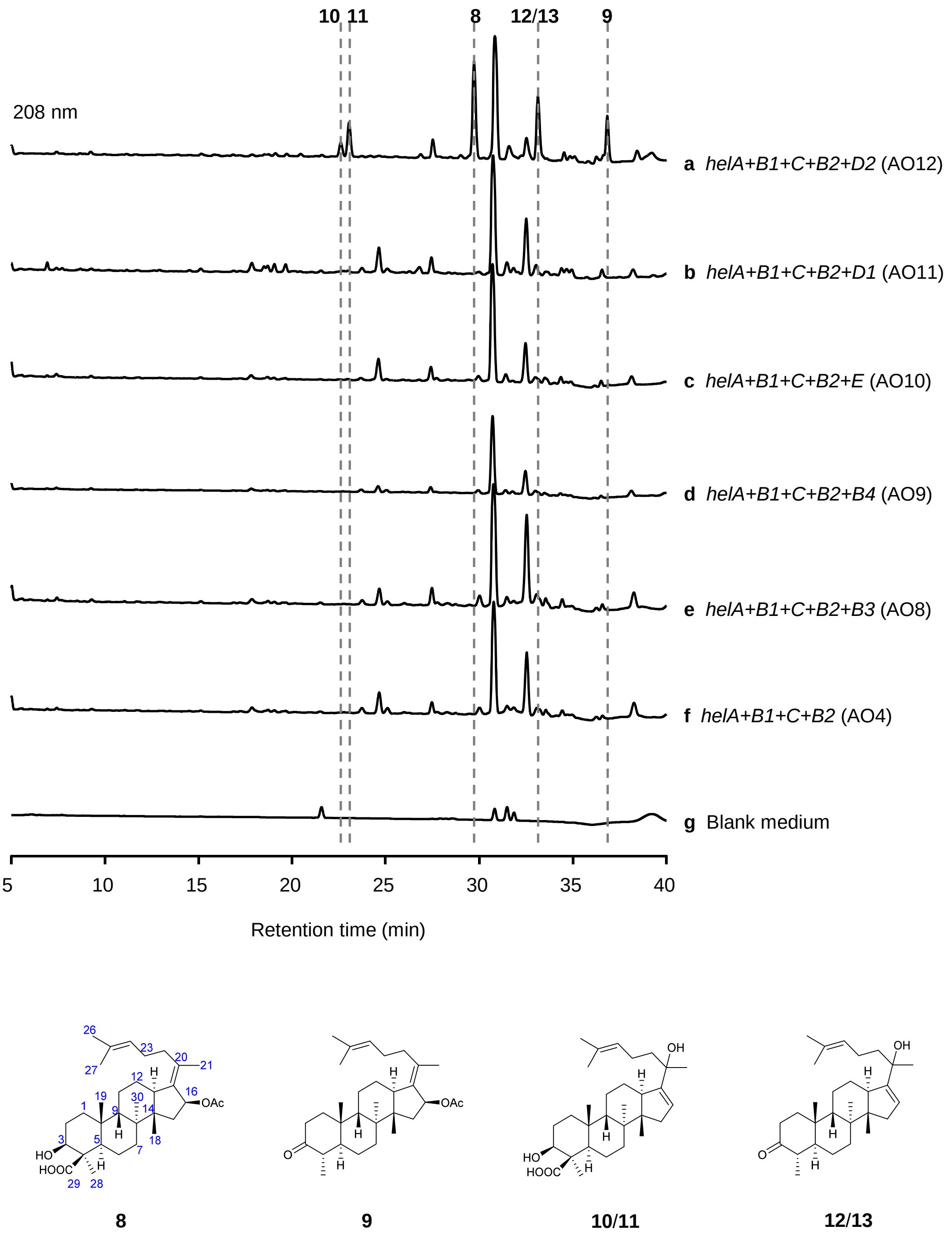

Supplementary Figure 4. HPLC analysis for identification of HelD2

a Mycelia extract of *A. oryzae* harboring *helA*, *helB1*, *helC*, *helB2* and *helD2*; **b** Mycelia extract of *A. oryzae* harboring *helA*, *helB1*, *helC*, *helB2* and *helD1*; **c** Mycelia extract of *A. oryzae* harboring *helA*, *helB1*, *helC*, *helB2* and *helE*; **d** Mycelia extract of *A. oryzae* harboring *helA*, *helB1*, *helC*, *helB2* and *helB4*; **e** Mycelia extract of *A. oryzae* harboring *helA*, *helB1*, *helC*, *helB2* and *helB3*; **f** Mycelia extract of *A. oryzae* harboring *helA*, *helB1*, *helC* and *helB2*; **g** Blank medium.

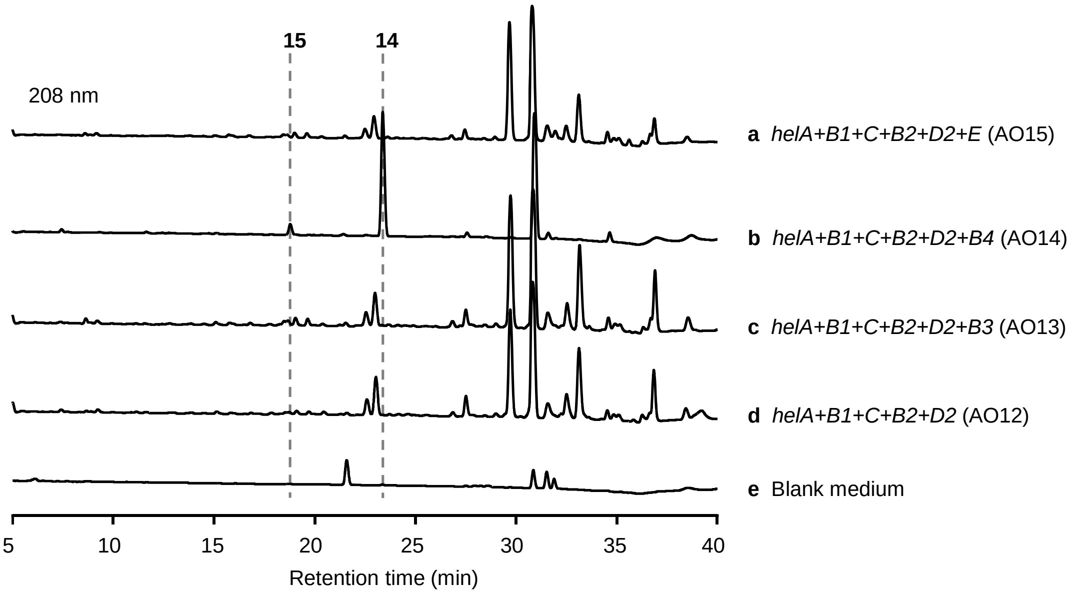

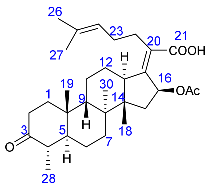

14

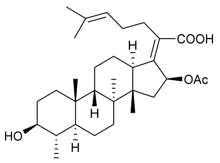

15

Supplementary Figure 5. HPLC analysis for identification of HelB4

a Mycelia extract of *A. oryzae* harboring *helA*, *helB1*, *helC*, *helB2*, *helD2* and *helE*; **b** Mycelia extract of *A. oryzae* harboring *helA*, *helB1*, *helC*, *helB2*, *helD2* and *helB4*; **c** Mycelia extract of *A. oryzae* harboring *helA*, *helB1*, *helC*, *helB2*, *helD2* and *helB3*; **d** Mycelia extract of *A. oryzae* harboring *helA*, *helB1*, *helC*, *helB2* and *helD2*; **e** Blank medium.

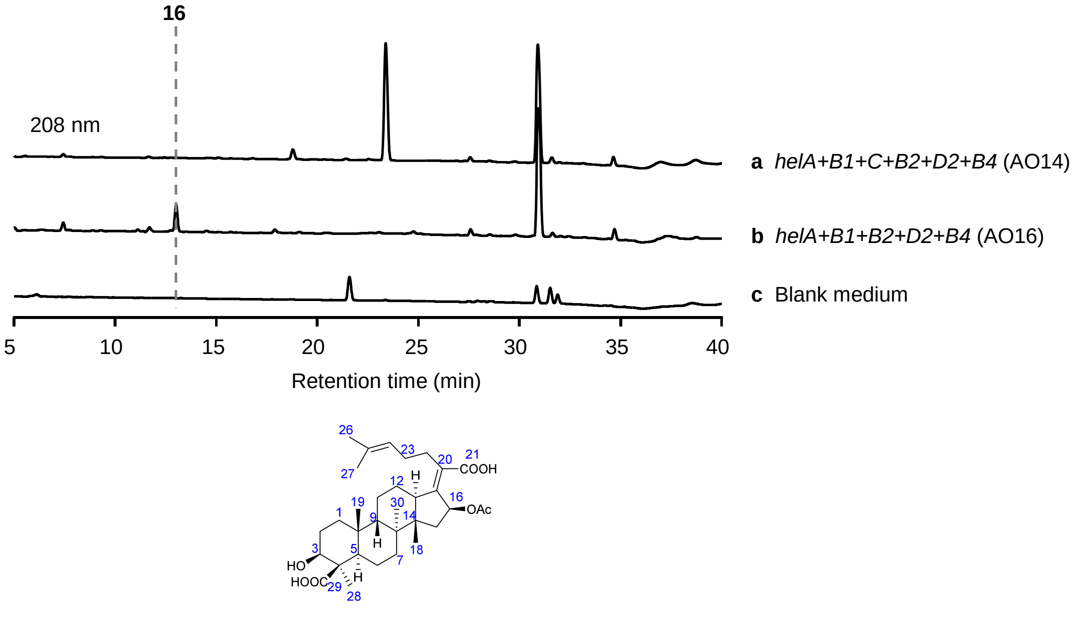

16

Supplementary Figure 6. HPLC analysis for tailoring by HelB4 in the absence of HelC

a Mycelia extract of *A. oryzae* harboring *helA*, *helB1*, *helC*, *helB2*, *helD2* and *helB4*; **b** Mycelia extract of *A. oryzae* harboring *helA*, *helB1*, *helB2*, *helD2* and *helB4*; **c** Blank medium.

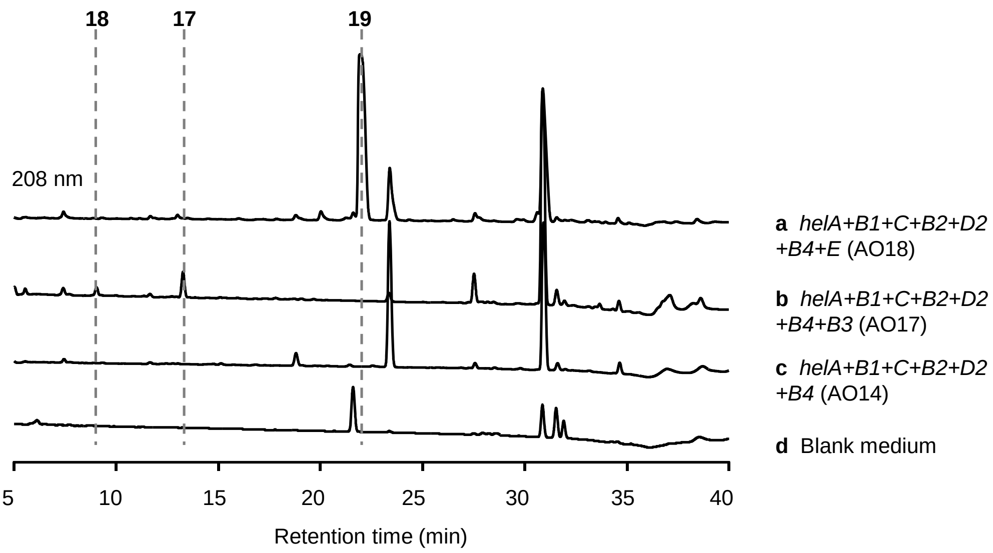

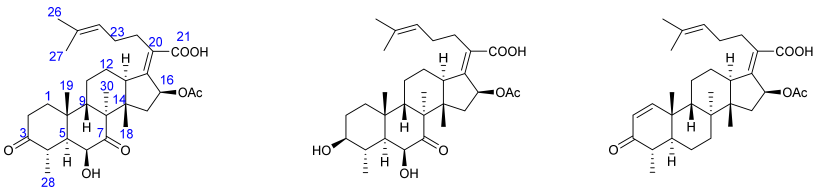

17 **18** **19**

Supplementary Figure 7. HPLC analysis for identification of HelB3 and HelE

a Mycelia extract of *A. oryzae* harboring *helA*, *helB1*, *helC*, *helB2*, *helD2*, *helB4* and *helE*; **b** Mycelia extract of *A. oryzae* harboring *helA*, *helB1*, *helC*, *helB2*, *helD2*, *helB4* and *helB3*; **c** Mycelia extract of *A. oryzae* harboring *helA*, *helB1*, *helC*, *helB2*, *helD2* and *helB4*; **d** Blank medium.

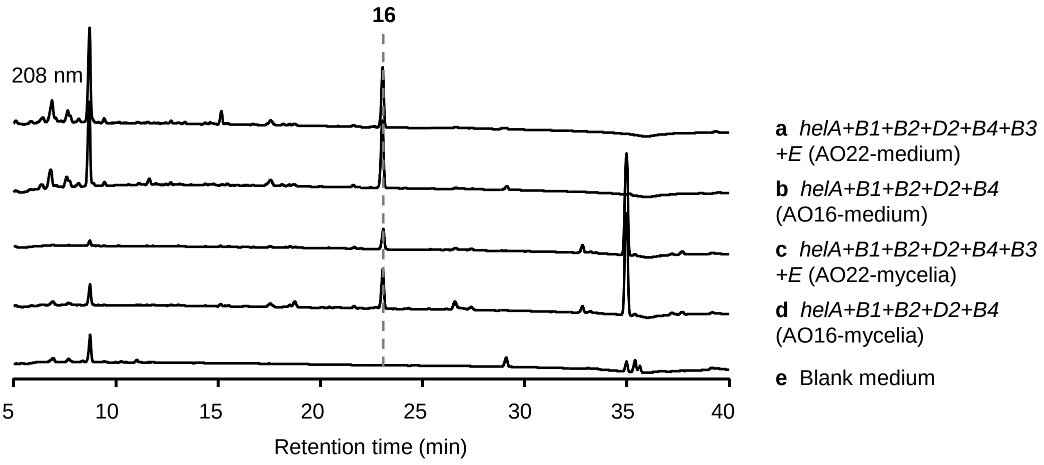

Supplementary Figure 8. HPLC analysis for tailoring by HelB3 and HelE in the absence of HelC

a Medium extract of *A. oryzae* harboring *helA*, *helB1*, *helB2*, *helD2*, *helB4*, *helB3* and *helE*; **b** Medium extract of *A. oryzae* harboring *helA*, *helB1*, *helB2*, *helD2* and *helB4*; **c** Mycelia extract of *A. oryzae* harboring *helA*, *helB1*, *helB2*, *helD2*, *helB4*, *helB3* and *helE*; **d** Mycelia extract of *A. oryzae* harboring *helA*, *helB1*, *helB2*, *helD2* and *helB4*; **e** Blank medium. The HPLC condition was as follows: H_2O containing 0.1% formic acid (A) and CH_3CN containing 0.1% formic acid (B); 20%-100% B (0-30 min), and 100%-100% B (30-40 min); 1 mL min^{-1}; 208 nm.

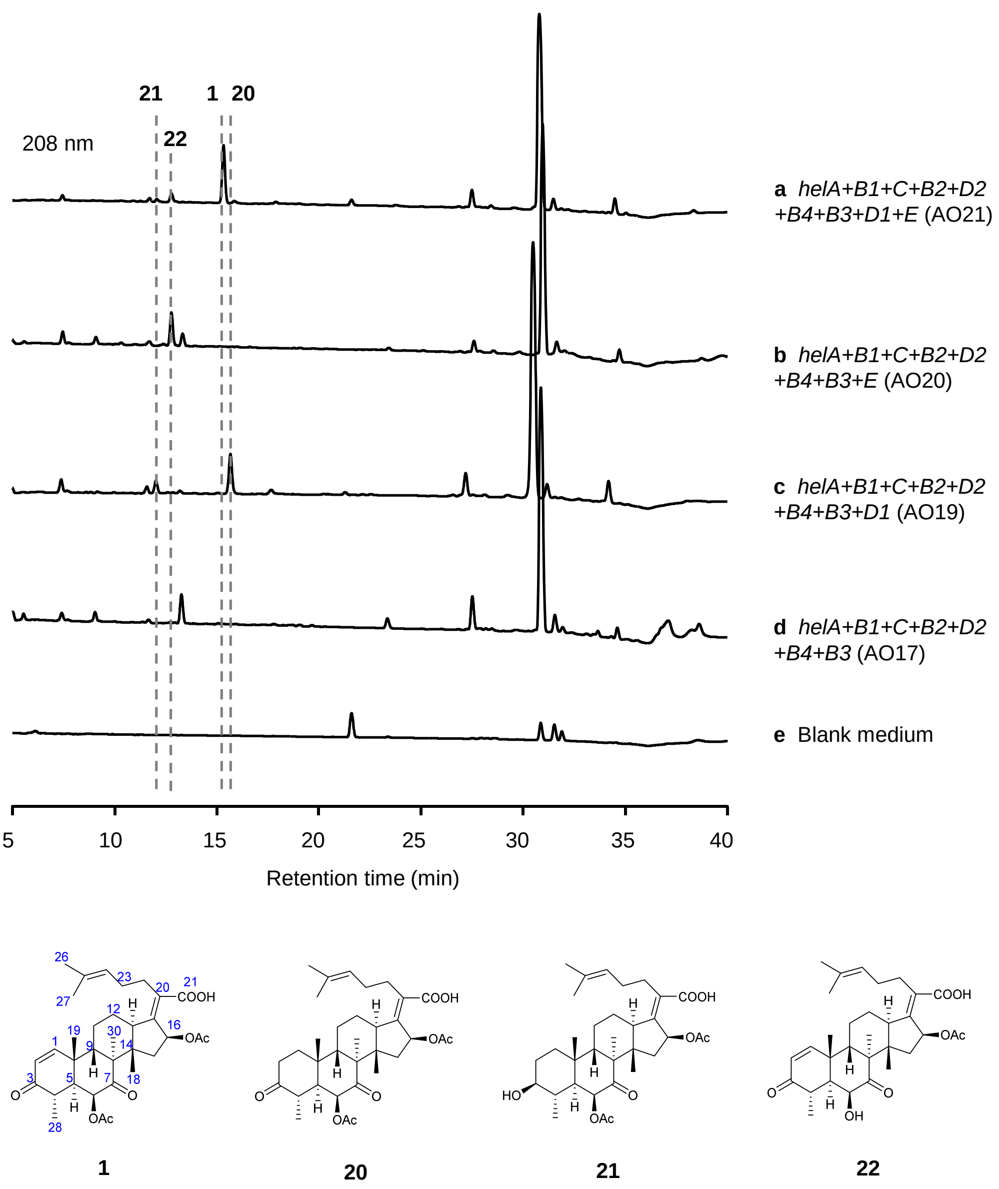

Supplementary Figure 9. HPLC analysis for identification of HelD1

a Mycelia extract of *A. oryzae* harboring *helA*, *helB1*, *helC*, *helB2*, *helD2*, *helB4*, *helB3*, *helD1* and *helE*; **b** Mycelia extract of *A. oryzae* harboring *helA*, *helB1*, *helC*, *helB2*, *helD2*, *helB4*, *helB3* and *helE*; **c** Mycelia extract of *A. oryzae* harboring *helA*, *helB1*, *helC*, *helB2*, *helD2*, *helB4*, *helB3* and *helD1*; **d** Mycelia extract of *A. oryzae* harboring *helA*, *helB1*, *helC*, *helB2*, *helD2*, *helB4* and *helB3*; **e** Blank medium.

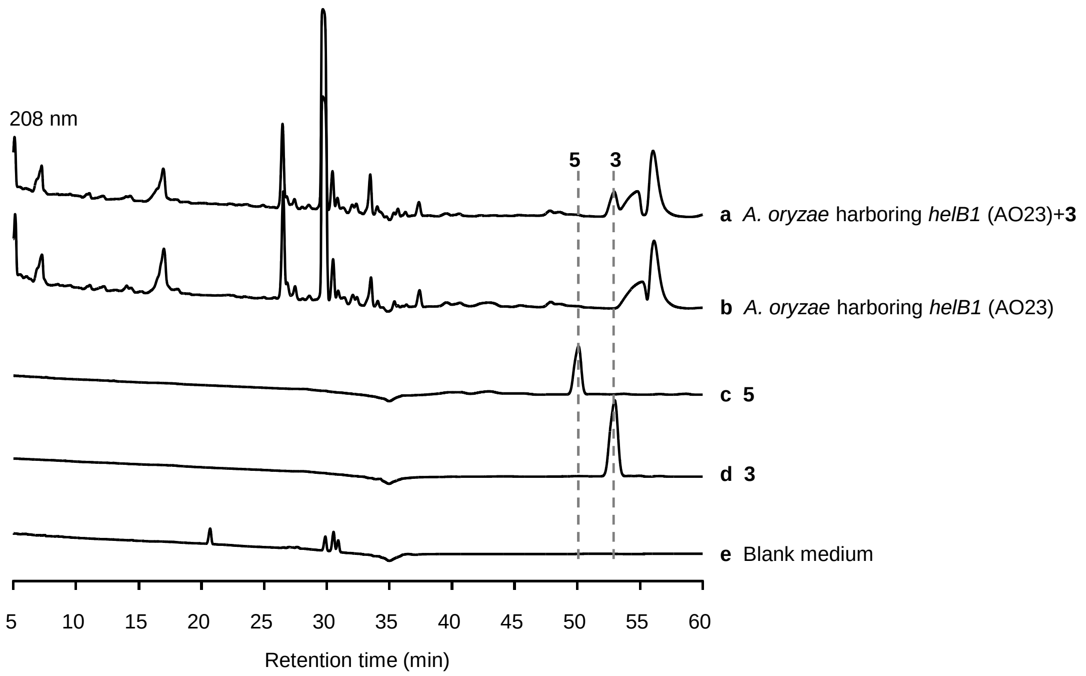

Supplementary Figure 10. HPLC analysis of mycelia extract of *A. oryzae* harboring *helB1* incubated with 3

a *A. oryzae* harboring *helB1* incubated with **3**; **b** *A. oryzae* harboring *helB1*; **c** Compound **5**; **d** Compound **3**; **e** Blank medium.

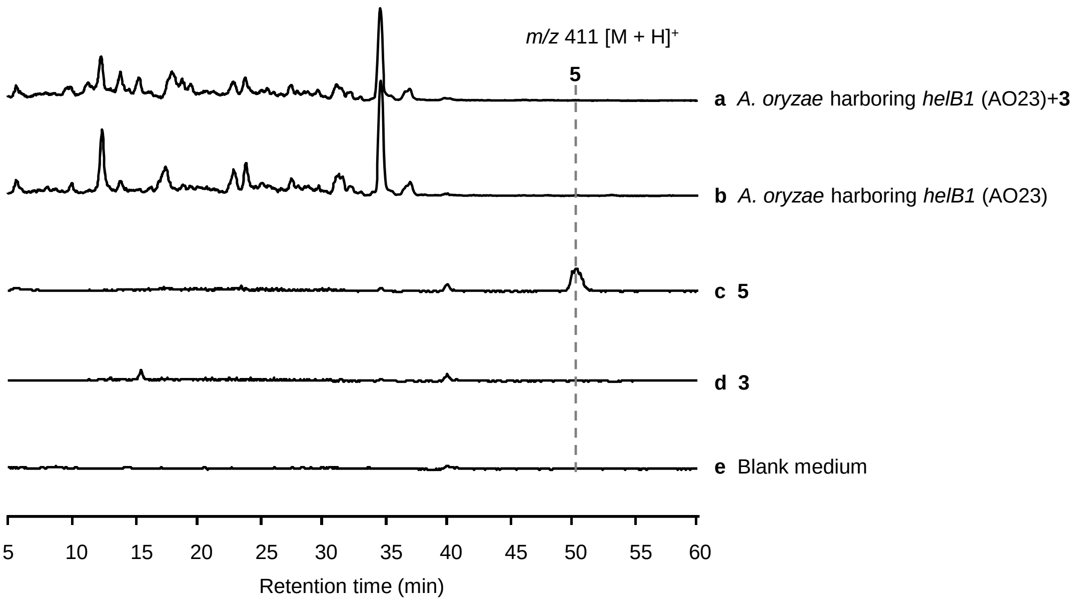

Supplementary Figure 11. EIC analysis of mycelia extract of *A. oryzae* harboring *helB1* incubated with 3

a *A. oryzae* harboring *helB1* incubated with **3**; **b** *A. oryzae* harboring *helB1*; **c** Compound **5**; **d** Compound **3**; **e** Blank medium.

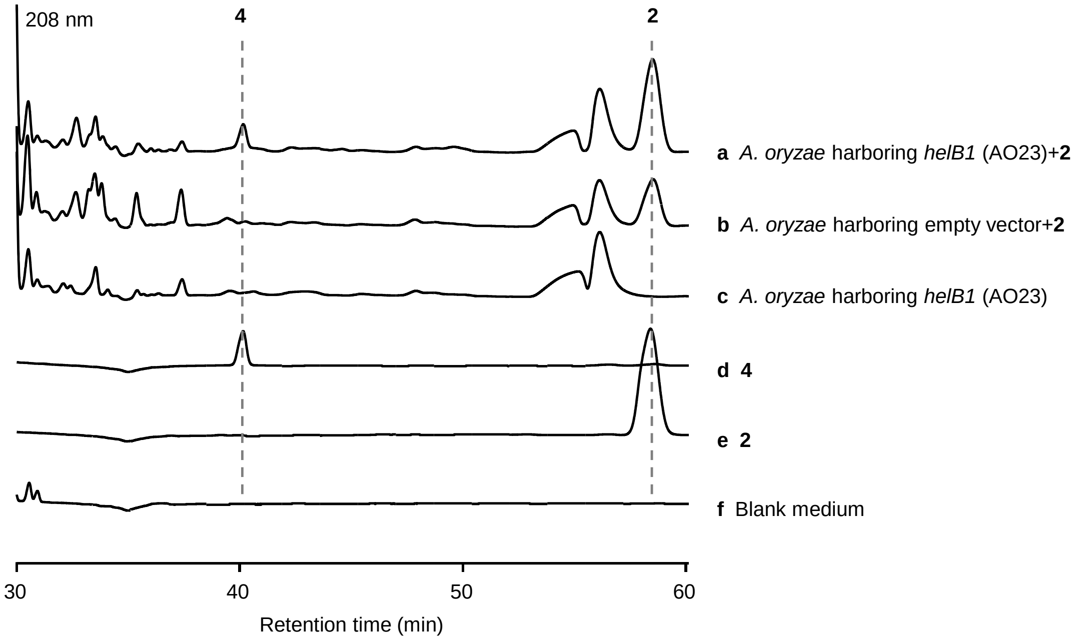

Supplementary Figure 12. HPLC analysis of mycelia extract of *A. oryzae* harboring *helB1* incubated with 2

a *A. oryzae* harboring *helB1* incubated with **2**; **b** *A. oryzae* harboring the empty vector incubated with **2**; **c** *A. oryzae* harboring *helB1*; **d** Compound **4**; **e** Compound **2**; **f** Blank medium.

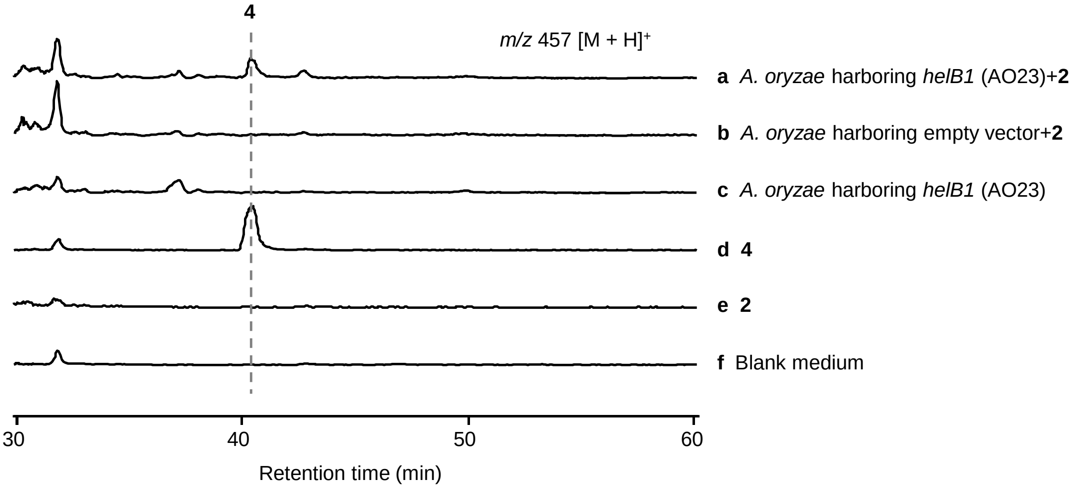

Supplementary Figure 13. EIC analysis of mycelia extract of *A. oryzae* harboring *helB1* incubated with 2

a *A. oryzae* harboring *helB1* incubated with **2**; **b** *A. oryzae* harboring the empty vector incubated with **2**; **c** *A. oryzae* harboring *helB1*; **d** Compound **4**; **e** Compound **2**; **f** Blank medium.

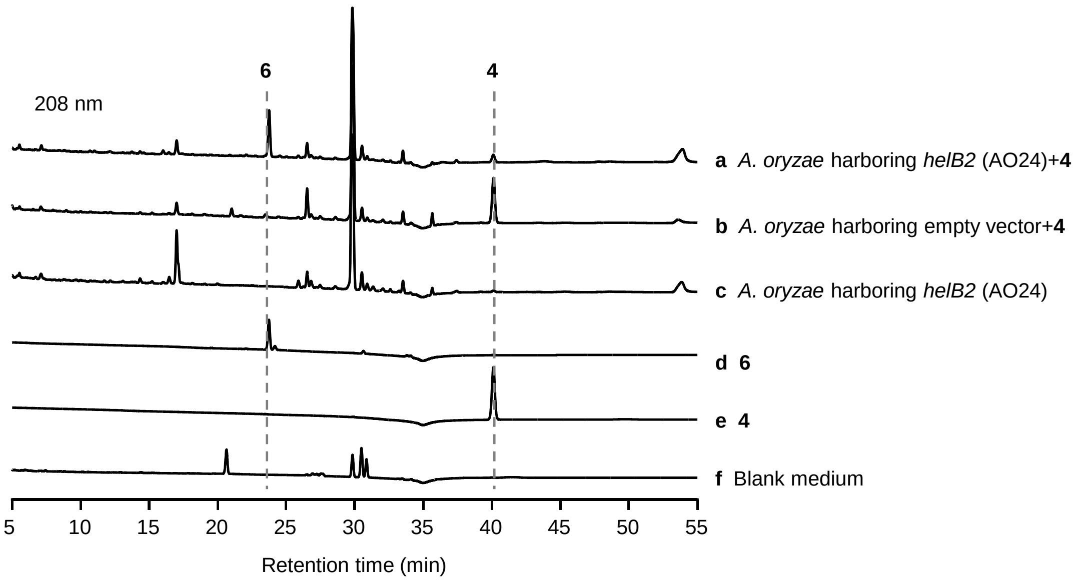

Supplementary Figure 14. HPLC analysis of mycelia extract of *A. oryzae* harboring *helB2* incubated with 4

a *A. oryzae* harboring *helB2* incubated with **4**; **b** *A. oryzae* harboring the empty vector incubated with **4**; **c** *A. oryzae* harboring *helB2*; **d** Compound **6**; **e** Compound **4**; **f** Blank medium.

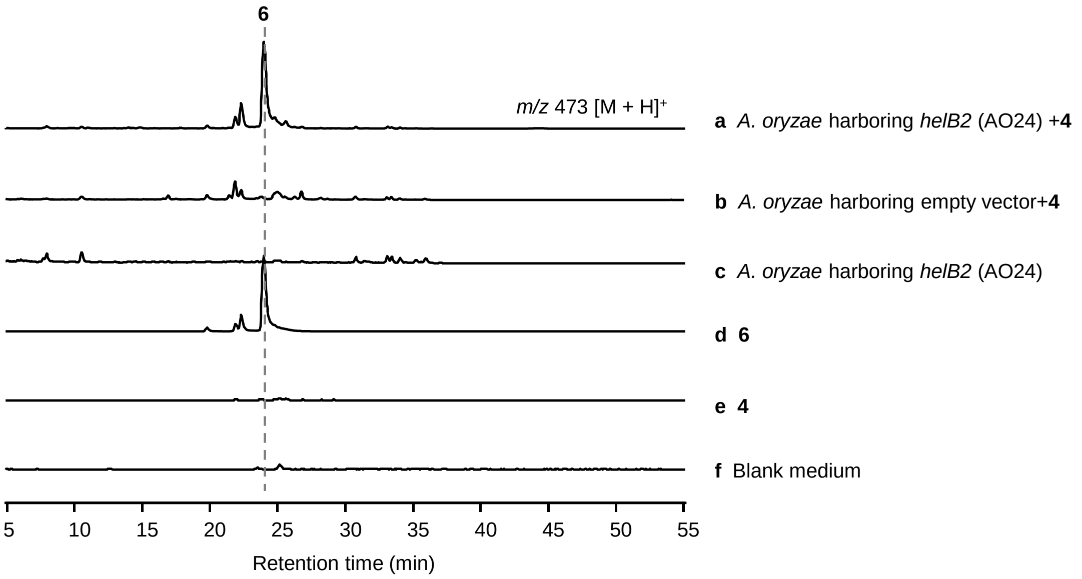

Supplementary Figure 15. EIC analysis of mycelia extract of *A. oryzae* harboring *helB2* incubated with 4

a *A. oryzae* harboring *helB2* incubated with **4**; **b** *A. oryzae* harboring the empty vector incubated with **4**; **c** *A. oryzae* harboring *helB2*; **d** Compound **6**; **e** Compound **4**; **f** Blank medium.

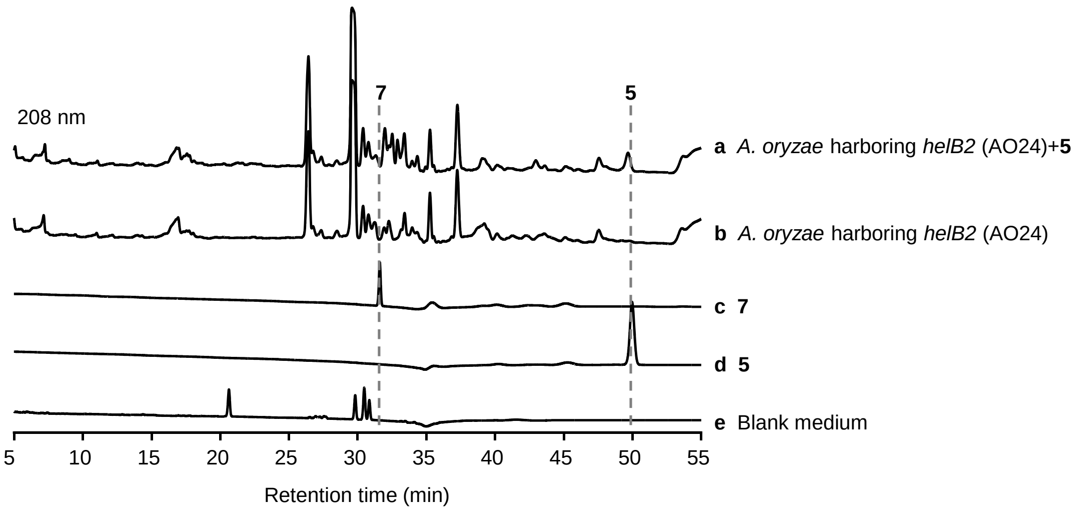

Supplementary Figure 16. HPLC analysis of mycelia extract of *A. oryzae* harboring *helB2* incubated with 5

a *A. oryzae* harboring *helB2* incubated with **5**; **b** *A. oryzae* harboring *helB2*; **c** Compound **7**; **d** Compound **5**; **e** Blank medium.

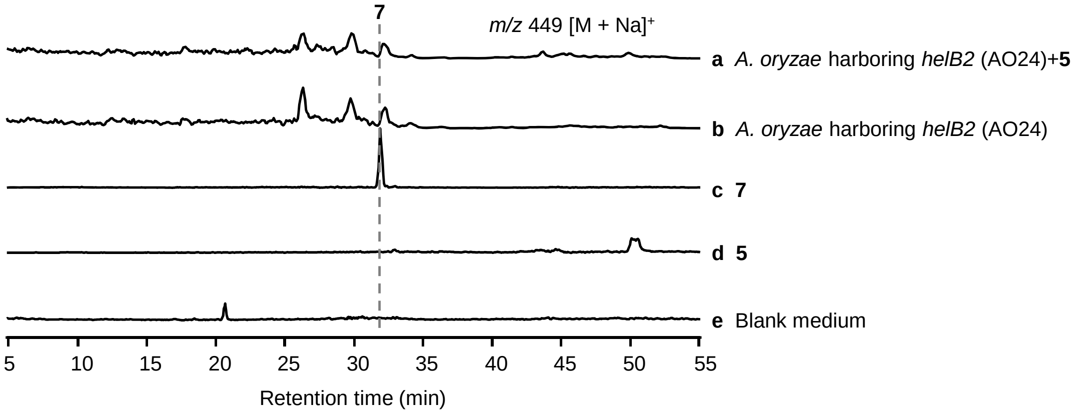

Supplementary Figure 17. EIC analysis of mycelia extract of *A. oryzae* harboring *helB2* incubated with 5

a *A. oryzae* harboring *helB2* incubated with **5**; **b** *A. oryzae* harboring *helB2*; **c** Compound **7**; **d** Compound **5**; **e** Blank medium.

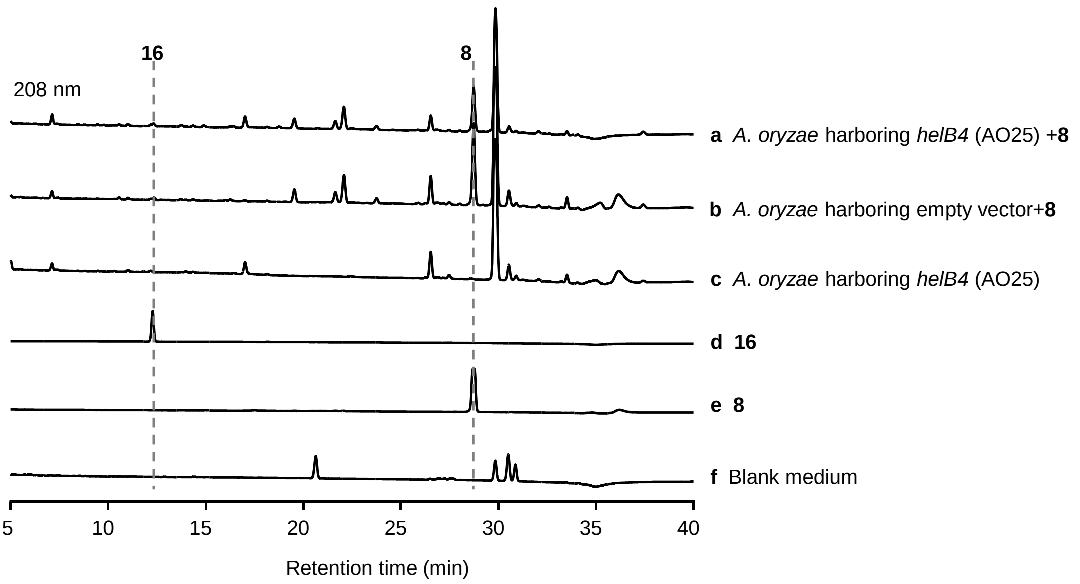

Supplementary Figure 18. HPLC analysis of mycelia extract of *A. oryzae* harboring *helB4* incubated with 8

a *A. oryzae* harboring *helB4* incubated with **8**; **b** *A. oryzae* harboring the empty vector incubated with **8**; **c** *A. oryzae* harboring *helB4*; **d** Compound **16**; **e** Compound **8**; **f** Blank medium.

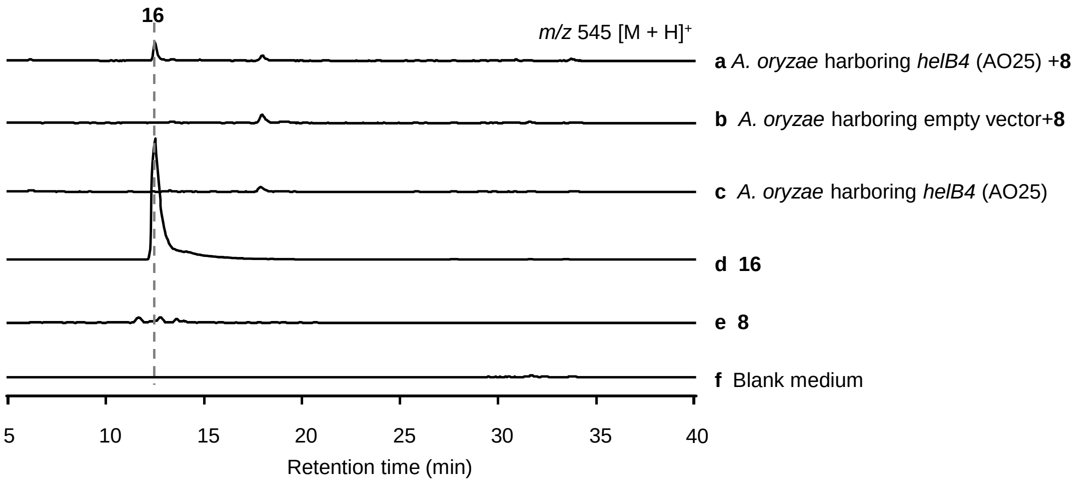

Supplementary Figure 19. EIC analysis of mycelia extract of *A. oryzae* harboring *helB4* incubated with 8

a *A. oryzae* harboring *helB4* incubated with **8**; **b** *A. oryzae* harboring the empty vector incubated with **8**; **c** *A. oryzae* harboring *helB4*; **d** Compound **16**; **e** Compound **8**; **f** Blank medium.

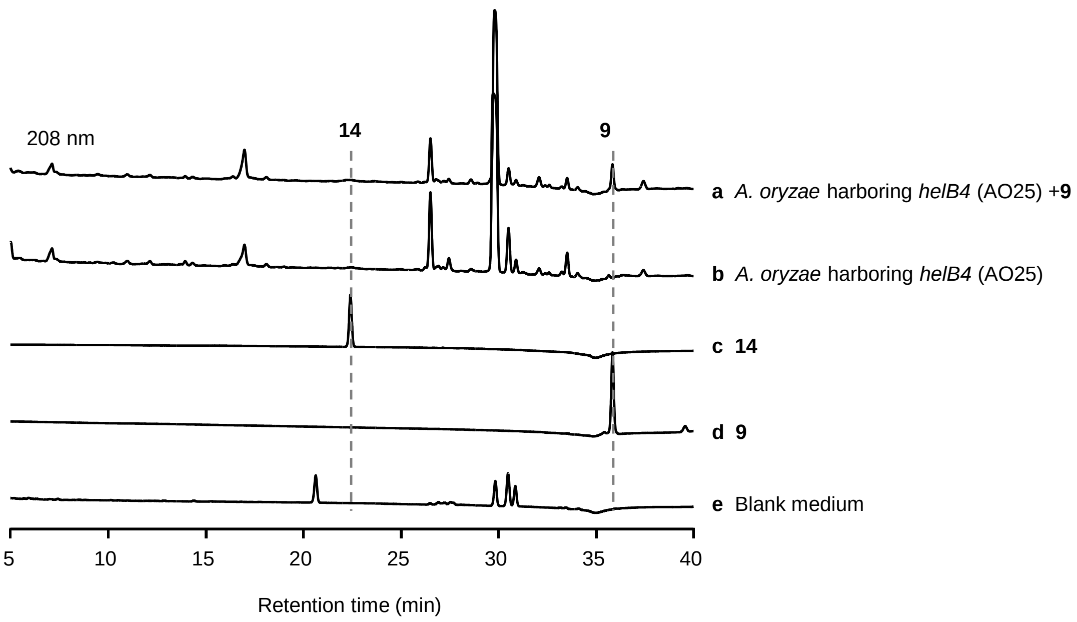

Supplementary Figure 20. HPLC analysis of mycelia extract of *A. oryzae* harboring *helB4* incubated with 9

a *A. oryzae* harboring *helB4* incubated with **9**; **b** *A. oryzae* harboring *helB4*; **c** Compound **14**; **d** Compound **9**; **e** Blank medium.

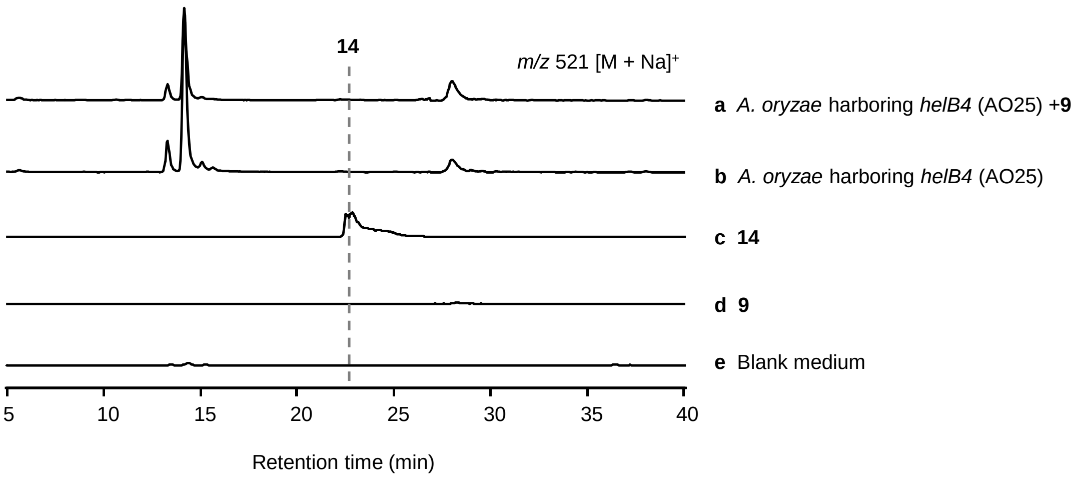

Supplementary Figure 21. EIC analysis of mycelia extract of *A. oryzae* harboring *helB4* incubated with 9

a *A. oryzae* harboring *helB4* incubated with **9**; **b** *A. oryzae* harboring *helB4*; **c** Compound **14**; **d** Compound **9**; **e** Blank medium.

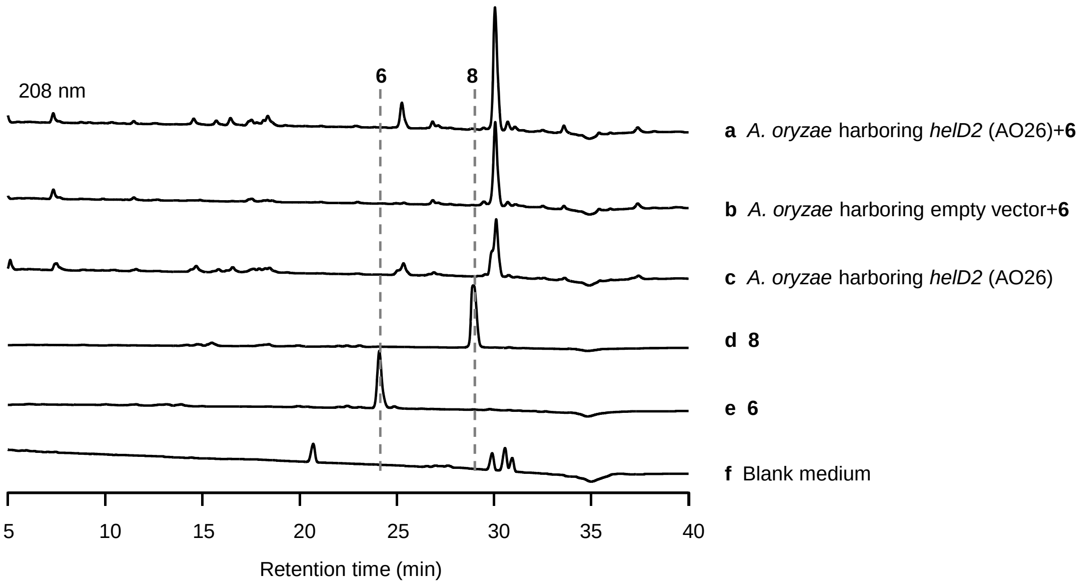

Supplementary Figure 22. HPLC analysis of mycelia extract of *A. oryzae* harboring *helD2* incubated with 6

a *A. oryzae* harboring *helD2* incubated with **6**; **b** *A. oryzae* harboring the empty vector incubated with **6**; **c** *A. oryzae* harboring *helD2*; **d** Compound **8**; **e** Compound **6**; **f** Blank medium.

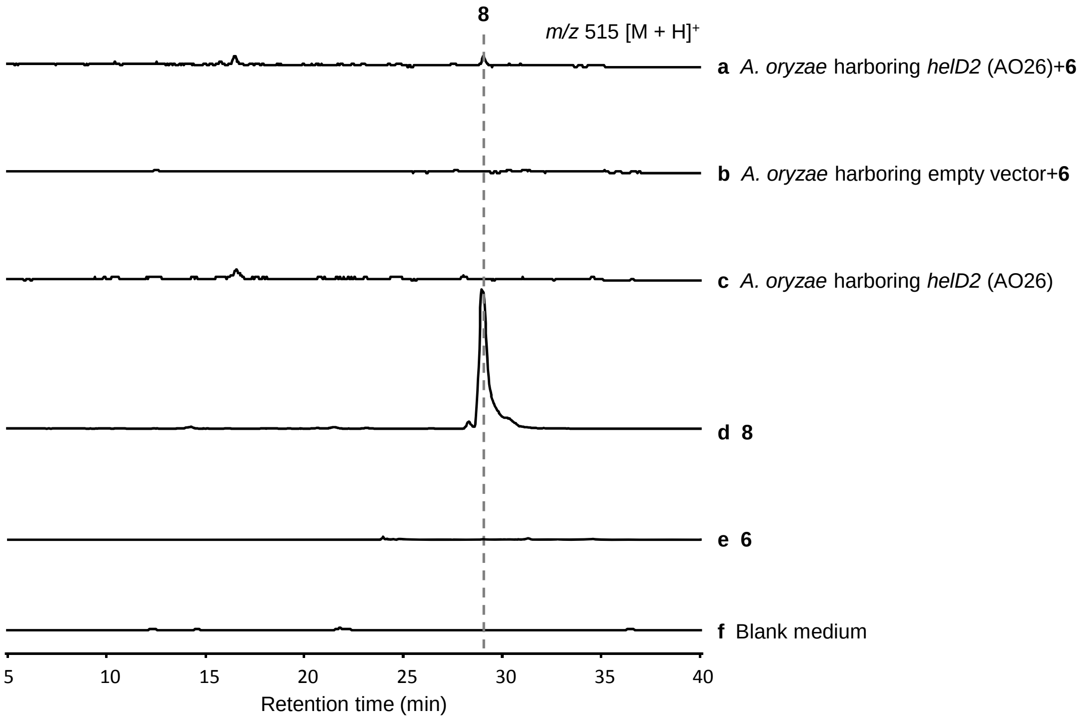

Supplementary Figure 23. EIC analysis of mycelia extract of *A. oryzae* harboring *helD2* incubated with 6

a *A. oryzae* harboring *helD2* incubated with **6**; **b** *A. oryzae* harboring the empty vector incubated with **6**; **c** *A. oryzae* harboring *helD2*; **d** Compound **8**; **e** Compound **6**; **f** Blank medium.

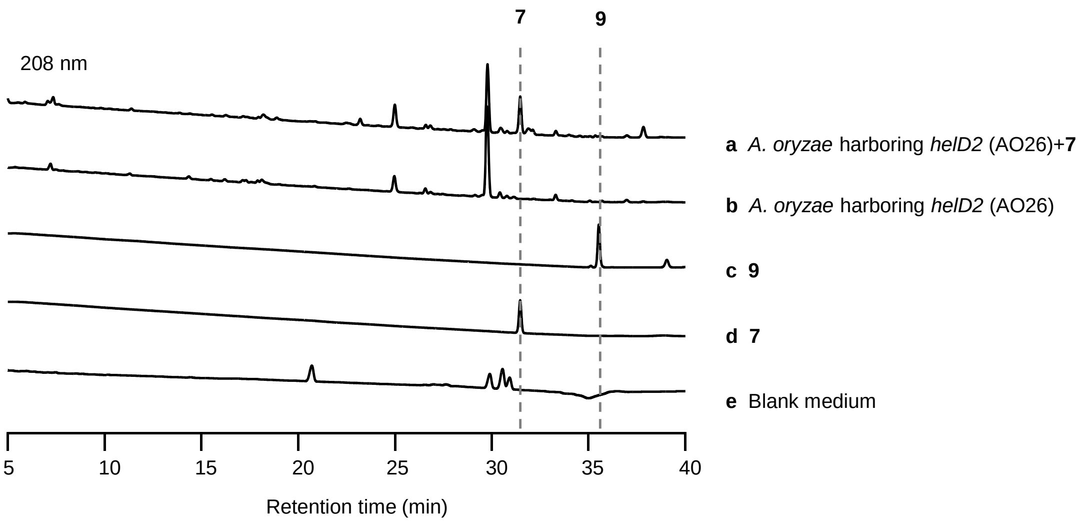

Supplementary Figure 24. HPLC analysis of mycelia extract of *A. oryzae* harboring *helD2* incubated with 7

a *A. oryzae* harboring *helD2* incubated with **7**; **b** *A. oryzae* harboring *helD2*; **c** Compound **9**; **d** Compound **7**; **e** Blank medium.

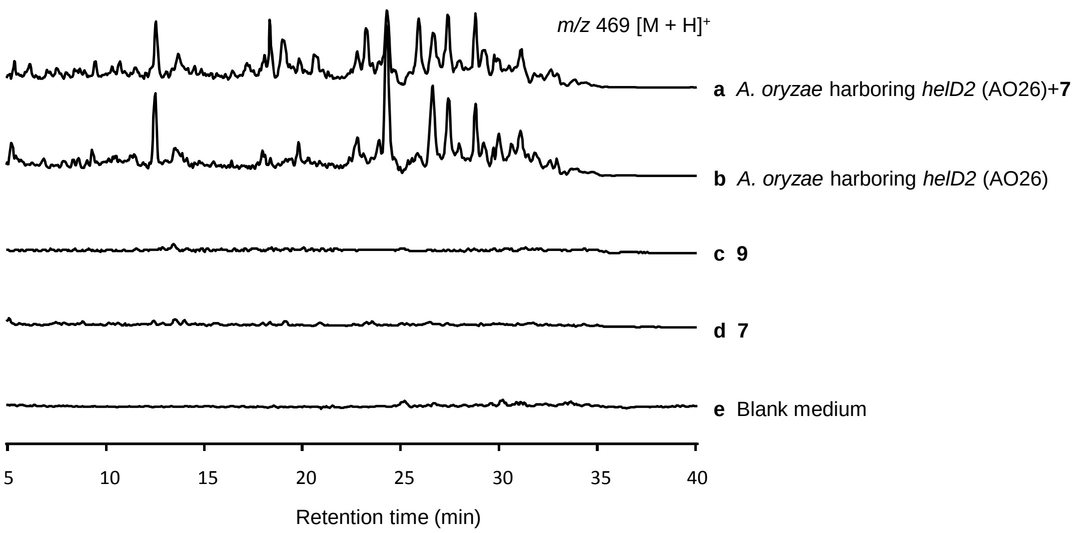

Supplementary Figure 25. EIC analysis of mycelia extract of *A. oryzae* harboring *helD2* incubated with 7

a *A. oryzae* harboring *helD2* incubated with **7**; **b** *A. oryzae* harboring *helD2*; **c** Compound **9**; **d** Compound **7**; **e** Blank medium.

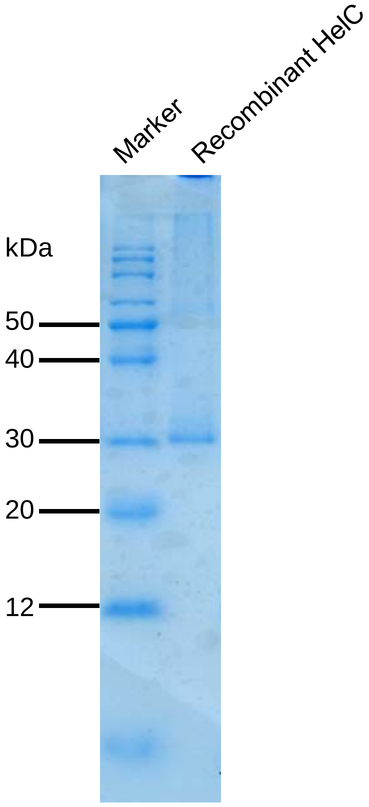

Supplementary Figure 26. SDS-PAGE analysis of recombinant HelC

HelC was tagged with 6 × His in *N*-terminal, and the molecular weight of recombinant HelC was about 29.8 kDa.

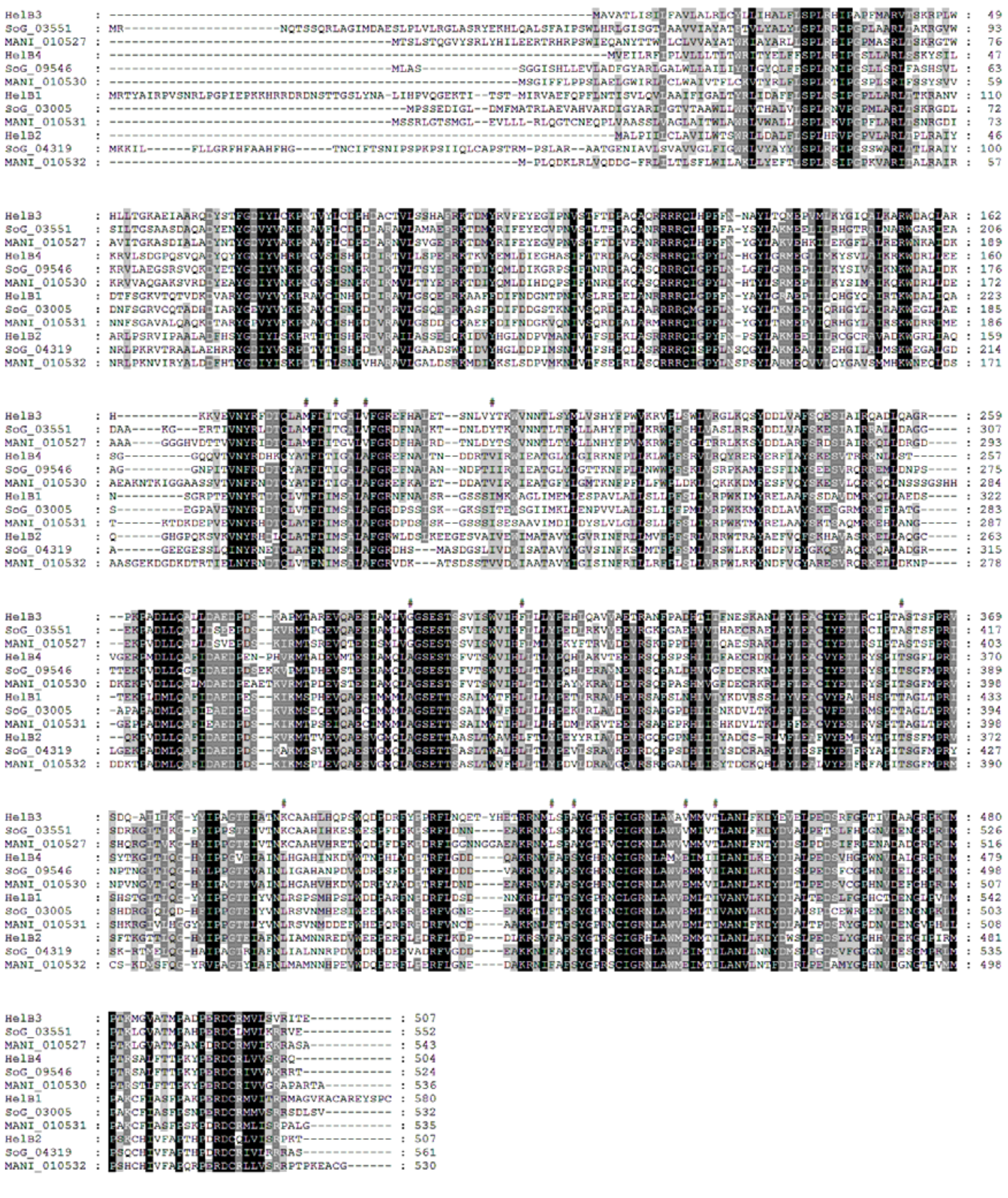

Supplementary Figure 27. Alignment of the amino acid sequences of P450s from the helvolic acid gene clusters

SoG_03005, SoG_03551, SoG_04319, and SoG_09546 are from *S. oryzae*. MANI_010527, MANI_010530, MANI_010531, and MANI_010532 are from *M. anisopliae*. SoG_03005 and MANI_010531 are the homologues of HelB1, SoG_04319 and MANI_010532 are the homologues of HelB2, SoG_03551 and MANI_010527 are the homologues of HelB3, and SoG_09546 and MANI_010530 are the homologues of HelB4. Lanes marked with # indicate that the conserved residues in HelB3 homologues are different from those in the rest P450s.

a

Single Mass Analysis
Tolerance = 5.0 mDa / DBE: min = -1.5, max = 15.0
Element prediction: Off
Number of isotope peaks used for i-FIT = 3

Monoisotopic Mass, Even Electron Ions
332 formula(e) evaluated with 2 results within limits (up to 50 closest results for each mass)
Elements Used:
C: 0-500 H: 0-1000 O: 0-200 Na: 0-1
afu-7
2016052325 194 (1.563) Cm (193:194)

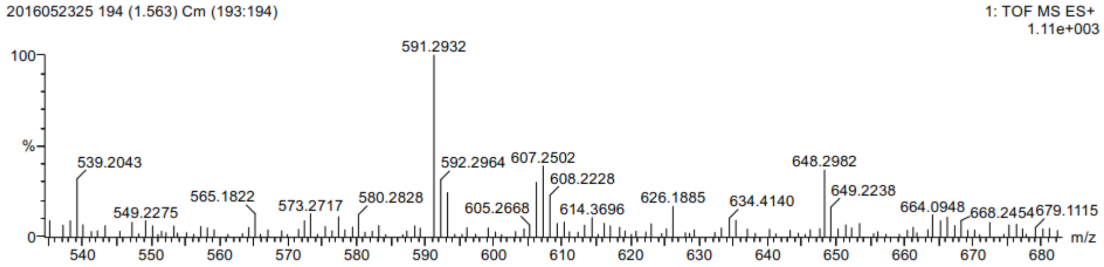

Minimum:				-1.5				
Maximum:		5.0	10.0	15.0				
Mass	Calc. Mass	mDa	PPM	DBE	i-FIT	Norm	Conf(%)	Formula
591.2932	591.2934	-0.2	-0.3	11.5	23.2	0.678	50.78	C33 H44 O8 Na
	591.2958	-2.6	-4.4	14.5	23.2	0.709	49.22	C35 H43 O8

b

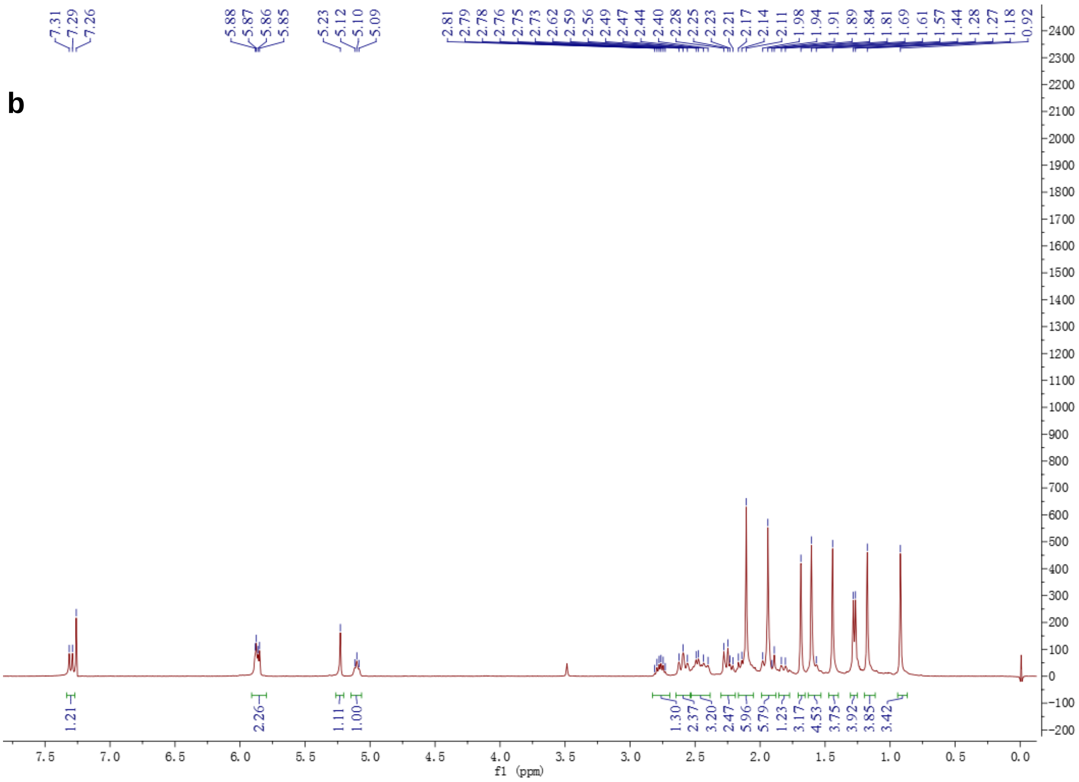

c

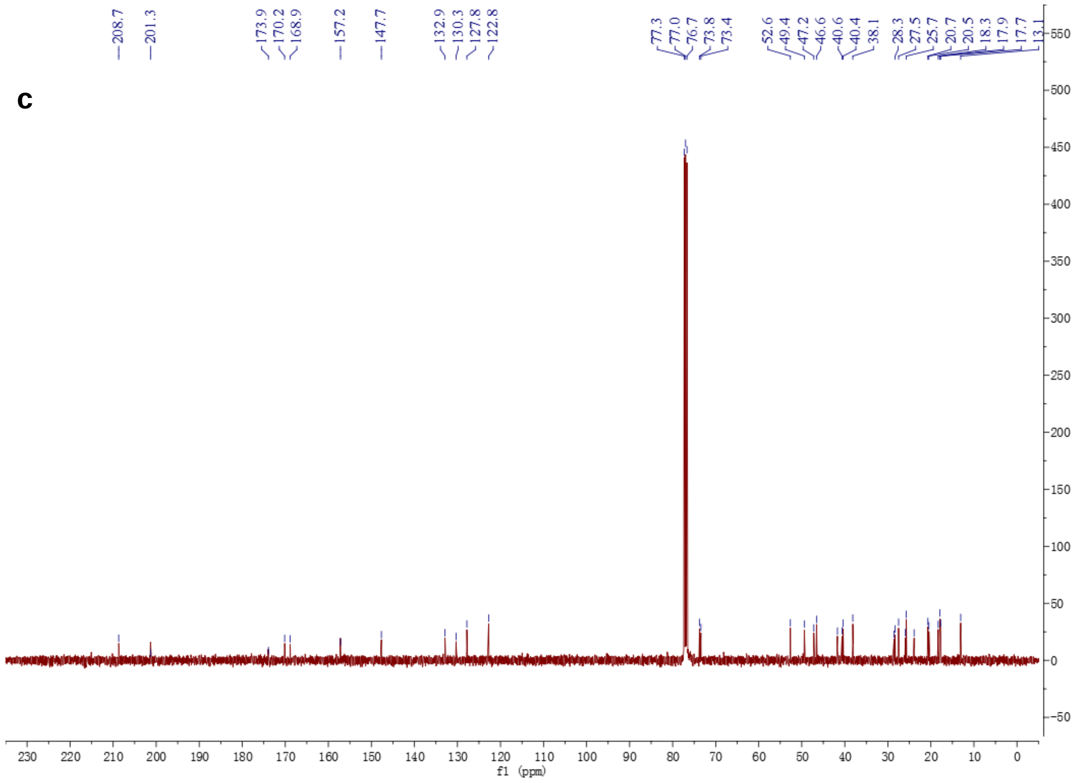

Supplementary Figure 28. HRESIMS and NMR spectra of 1

a HRESIMS spectrum; **b** ^{1}H NMR spectrum in $CDCl_3$ at 400 MHz; **c** ^{13}C NMR spectrum in $CDCl_3$ at 100 MHz.

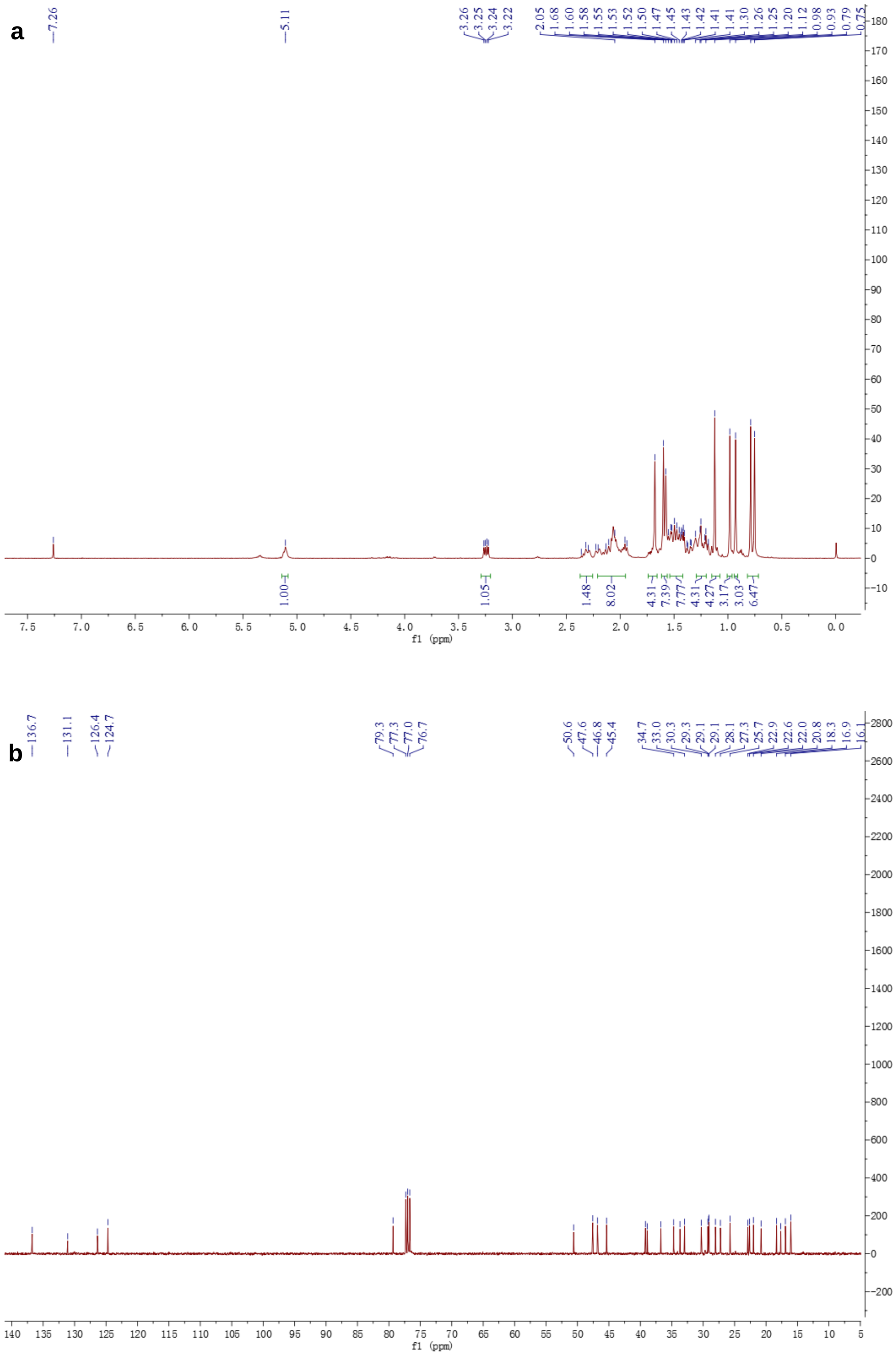

Supplementary Figure 29. NMR spectra of 2

a ^{1}H NMR spectrum in $CDCl_3$ at 400 MHz; **b** ^{13}C NMR spectrum in $CDCl_3$ at 100 MHz.

a

Single Mass Analysis
Tolerance = 5.0 mDa / DBE: min = -1.5, max = 15.0
Element prediction: Off
Number of isotope peaks used for i-FIT = 3

Monoisotopic Mass, Even Electron Ions
95 formula(e) evaluated with 1 results within limits (up to 50 closest results for each mass)
Elements Used:
C: 0-500 H: 0-1000 O: 0-200
AFLL-16
2016080102 345 (2.782)

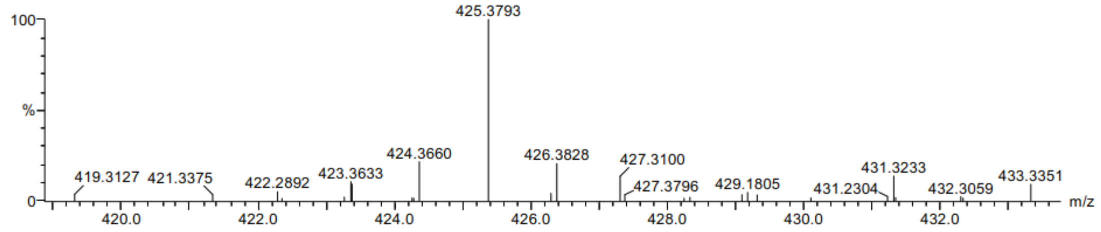

Minimum:				-1.5				
Maximum:		5.0	10.0	15.0				
Mass	Calc. Mass	mDa	PPM	DBE	i-FIT	Norm	Conf(%)	Formula
425.3793	425.3783	1.0	2.4	6.5	29.8	n/a	n/a	C30 H49 O

b

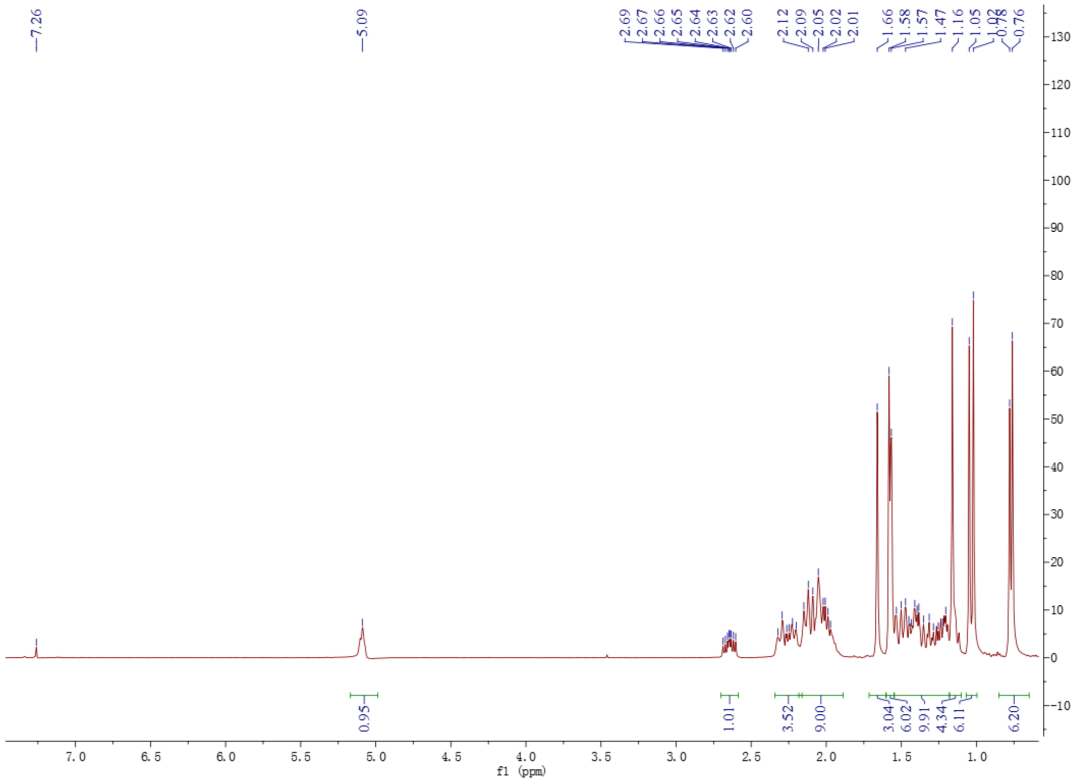

c

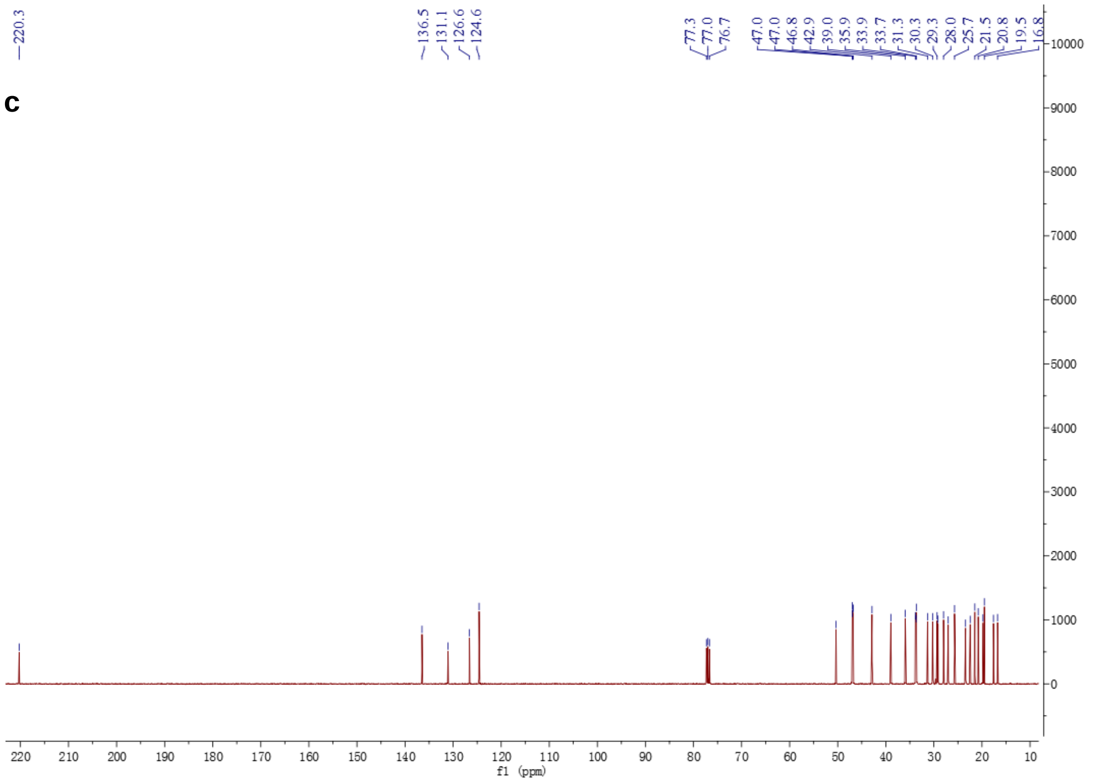

Supplementary Figure 30. HRESIMS and NMR spectra of 3

a HRESIMS spectrum; **b** ^{1}H NMR spectrum in $CDCl_3$ at 400 MHz; **c** ^{13}C NMR spectrum in $CDCl_3$ at 100 MHz.

a

Single Mass Analysis
Tolerance = 5.0 mDa / DBE: min = -1.5, max = 15.0
Element prediction: Off
Number of isotope peaks used for i-FIT = 3

Monoisotopic Mass, Even Electron Ions
108 formula(e) evaluated with 1 results within limits (up to 50 closest results for each mass)
Elements Used:
C: 0-500 H: 0-1000 O: 0-200
AFU-14
2016070402 295 (2.375)

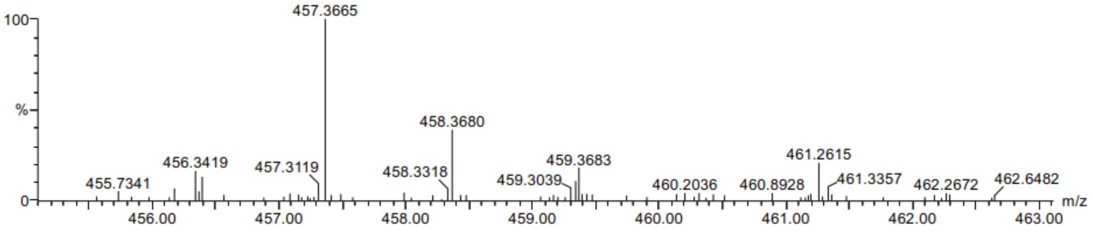

Minimum:				-1.5				
Maximum:		5.0	10.0	15.0				
Mass	Calc. Mass	mDa	PPM	DBE	i-FIT	Norm	Conf(%)	Formula
457.3665	457.3682	-1.7	-3.7	6.5	151.5	n/a	n/a	C30 H49 O3

b

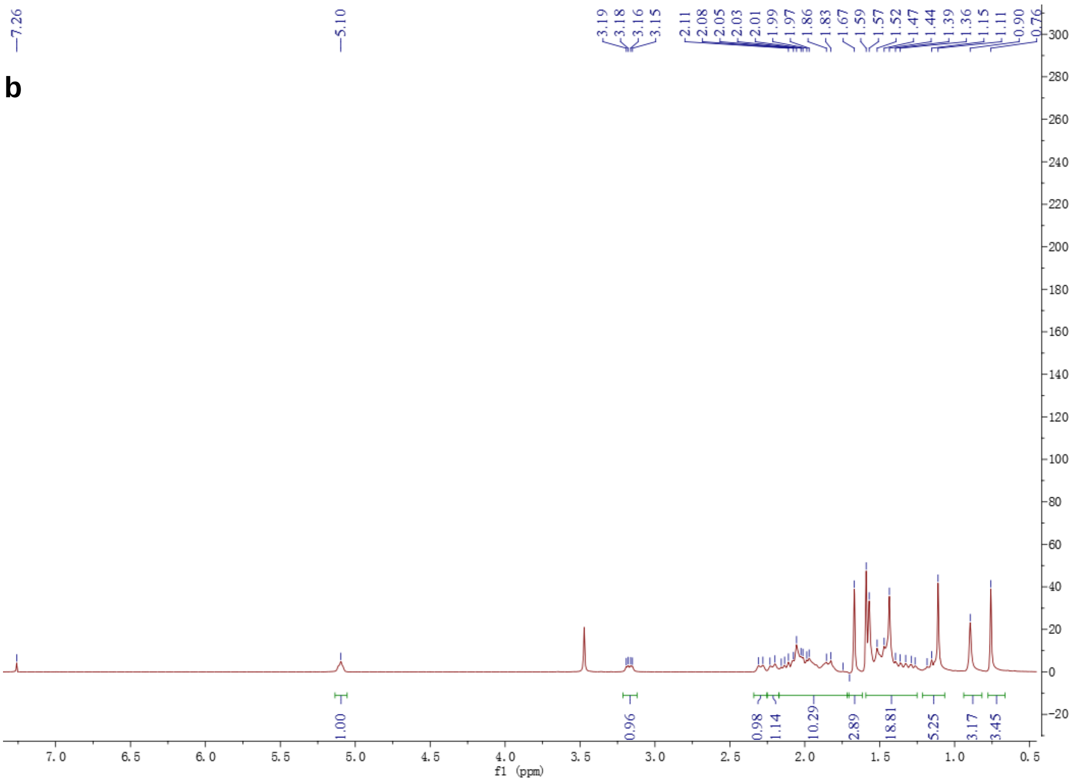

c

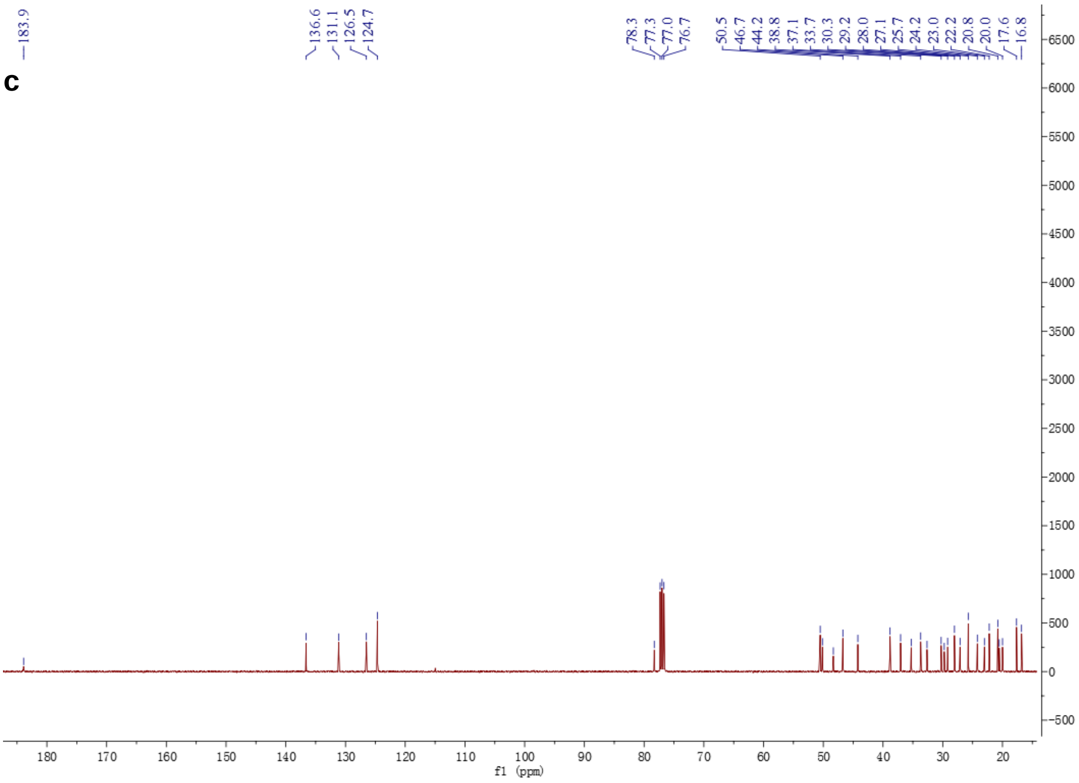

183.9
136.6
131.1
126.5
124.7
78.3
77.3
77.0
76.7
50.5
46.7
44.2
38.8
37.1
33.7
30.3
29.2
28.0
27.1
25.7
24.2
23.0
22.2
20.8
20.0
17.6
16.8
f1 (ppm)

d

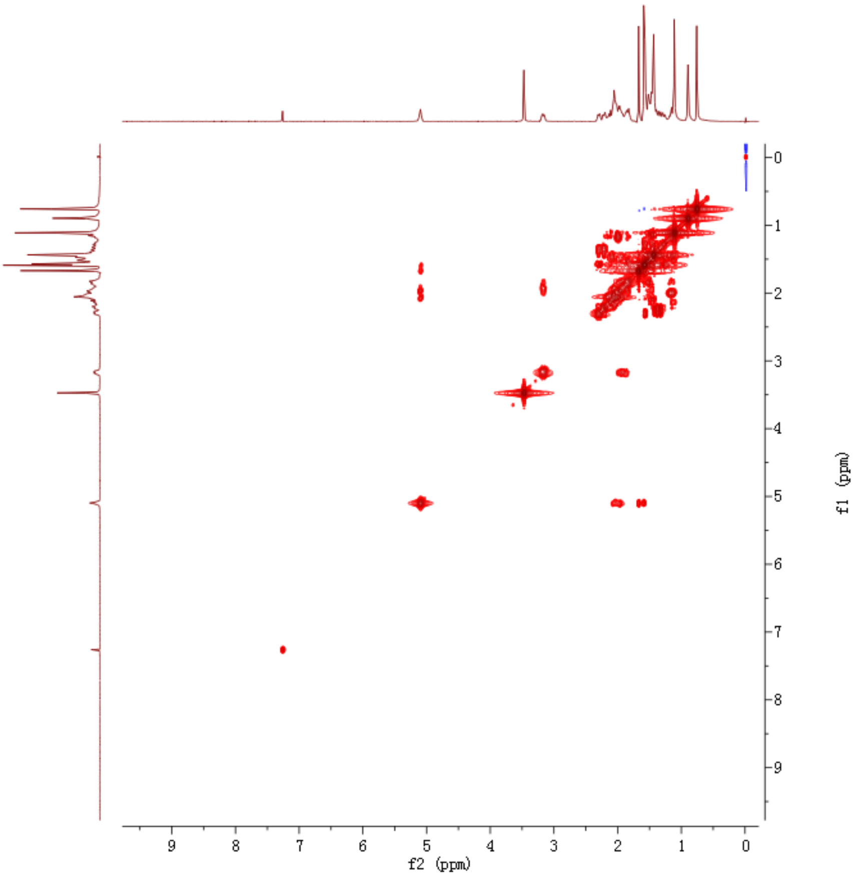

f2 (ppm)
f1 (ppm)

e

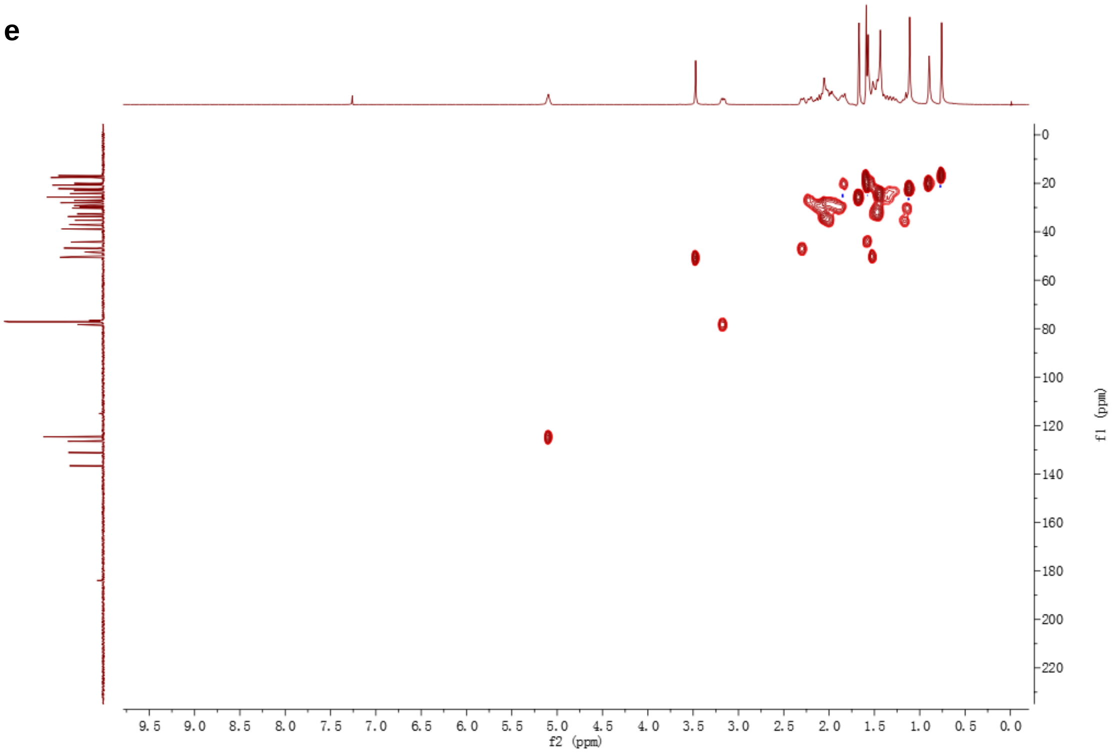

0
20
40
60
80
100
120
140
160
180
200
220
f1 (ppm)
9.5 9.0 8.5 8.0 7.5 7.0 6.5 6.0 5.5 5.0 4.5 4.0 3.5 3.0 2.5 2.0 1.5 1.0 0.5 0.0
f2 (ppm)

f

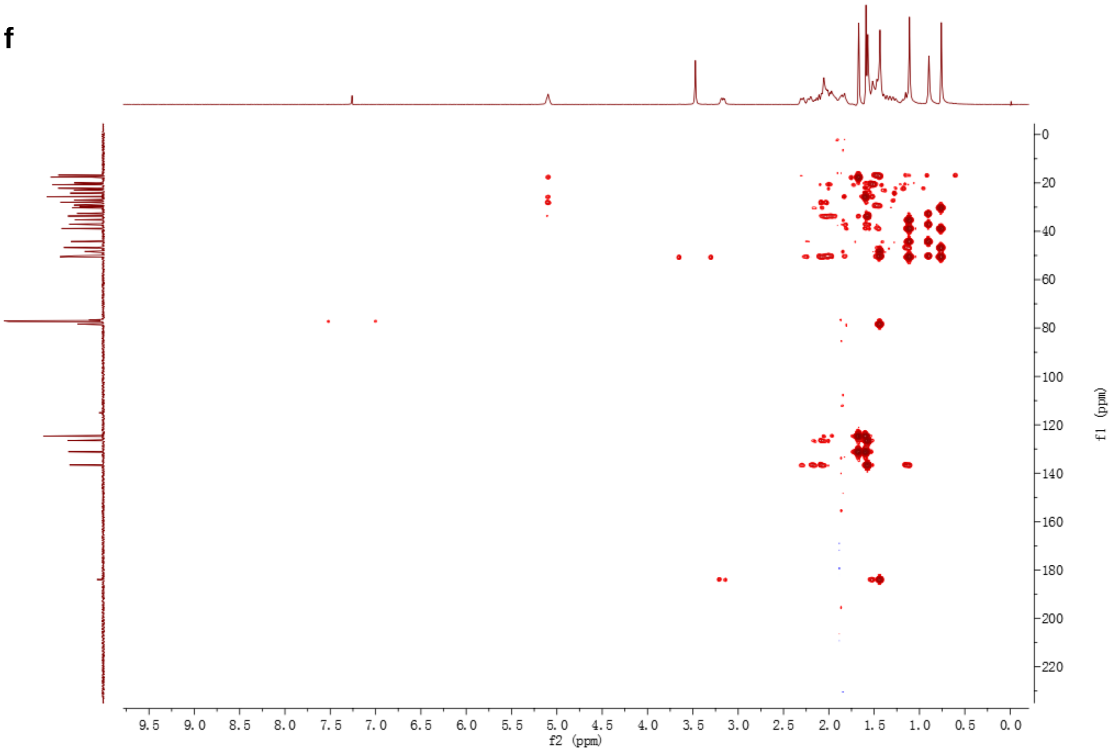

0
20
40
60
80
100
120
140
160
180
200
220
f1 (ppm)
9.5 9.0 8.5 8.0 7.5 7.0 6.5 6.0 5.5 5.0 4.5 4.0 3.5 3.0 2.5 2.0 1.5 1.0 0.5 0.0
f2 (ppm)

g

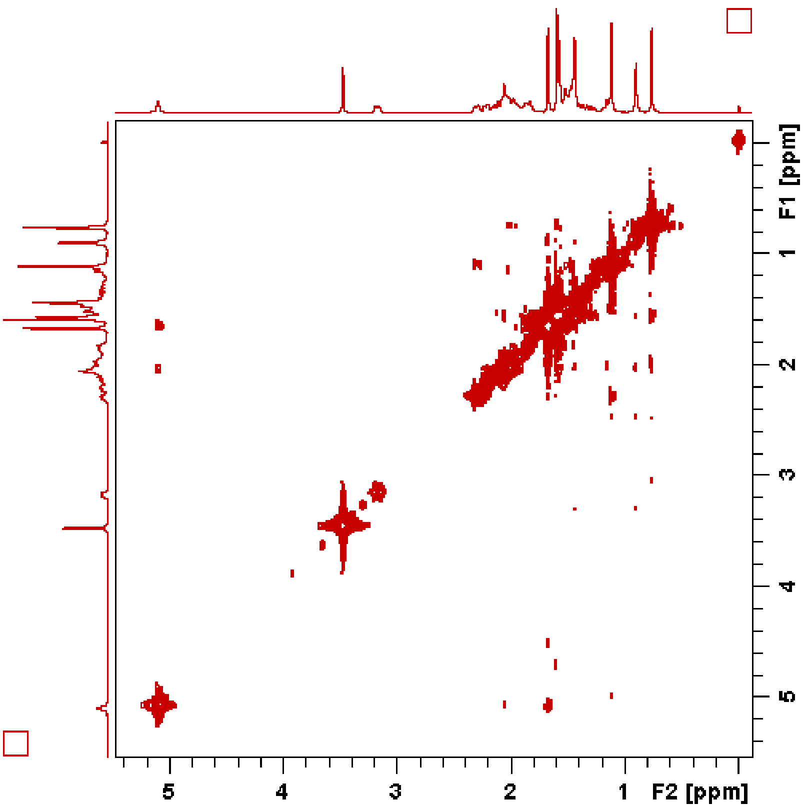

Supplementary Figure 31. HRESIMS and NMR spectra of 4

a HRESIMS spectrum; **b** ^{1}H NMR spectrum in $CDCl_3$ at 400 MHz; **c** ^{13}C NMR spectrum in $CDCl_3$ at 100 MHz; **d** ^{1}H-^{1}H COSY spectrum in $CDCl_3$ at 400 MHz; **e** HSQC spectrum in $CDCl_3$ at 400 MHz; **f** HMBC spectrum in $CDCl_3$ at 400 MHz; **g** NOESY spectrum in $CDCl_3$ at 400 MHz.

a

Single Mass Analysis
Tolerance = 5.0 mDa / DBE: min = -1.5, max = 15.0
Element prediction: Off
Number of isotope peaks used for i-FIT = 3

Monoisotopic Mass, Even Electron Ions
94 formula(e) evaluated with 1 results within limits (up to 50 closest results for each mass)
Elements Used:
C: 0-500 H: 0-1000 O: 0-200
AO-1
20151116010 340 (2.734)

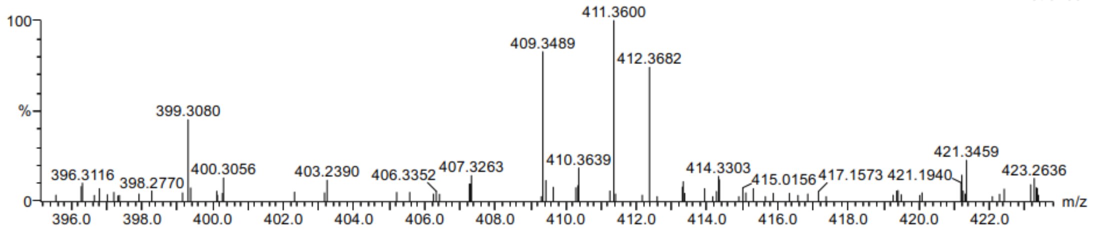

	Mass	Calc. Mass	mDa	PPM	DBE	i-FIT	Norm	Conf(%)	Formula
Minimum:					-1.5				
Maximum:			5.0	10.0	15.0				
	411.3600	411.3627	-2.7	-6.6	6.5	48.7	n/a	n/a	C29 H47 O

b

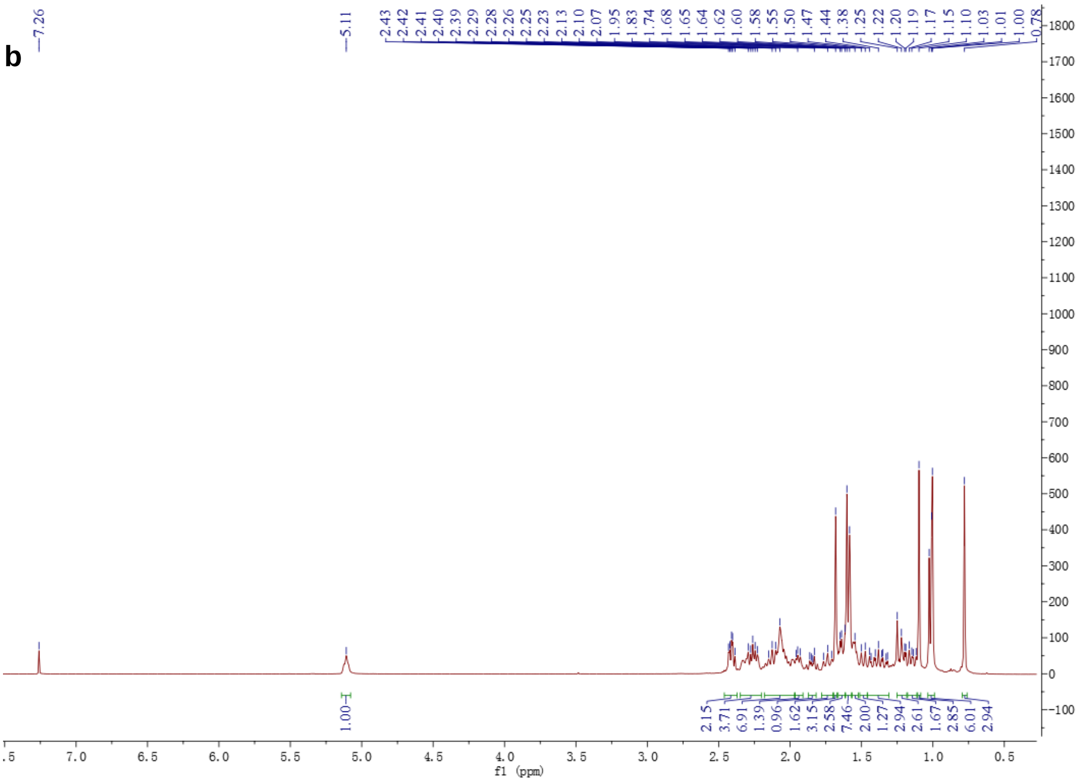

c

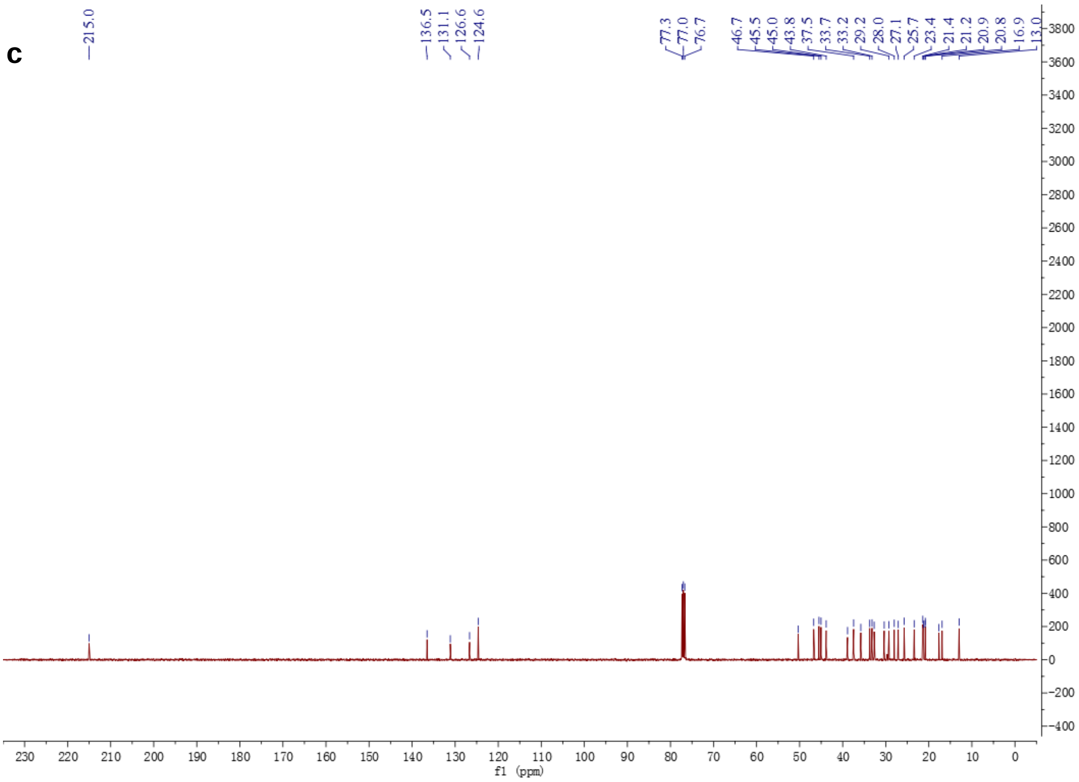
215.0
136.5
131.1
126.6
124.6
77.3
77.0
76.7
46.7
45.5
45.0
43.8
37.5
33.7
33.2
29.2
28.0
27.1
25.7
23.4
21.4
21.2
20.9
20.8
16.9
13.0
f1 (ppm)

d

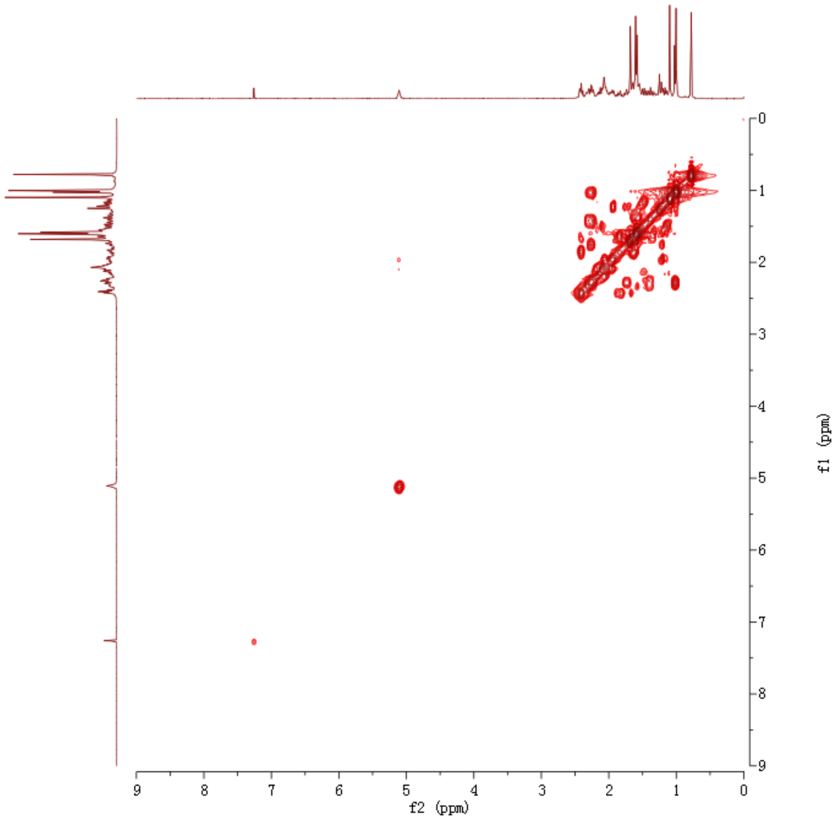
f2 (ppm)
f1 (ppm)

e

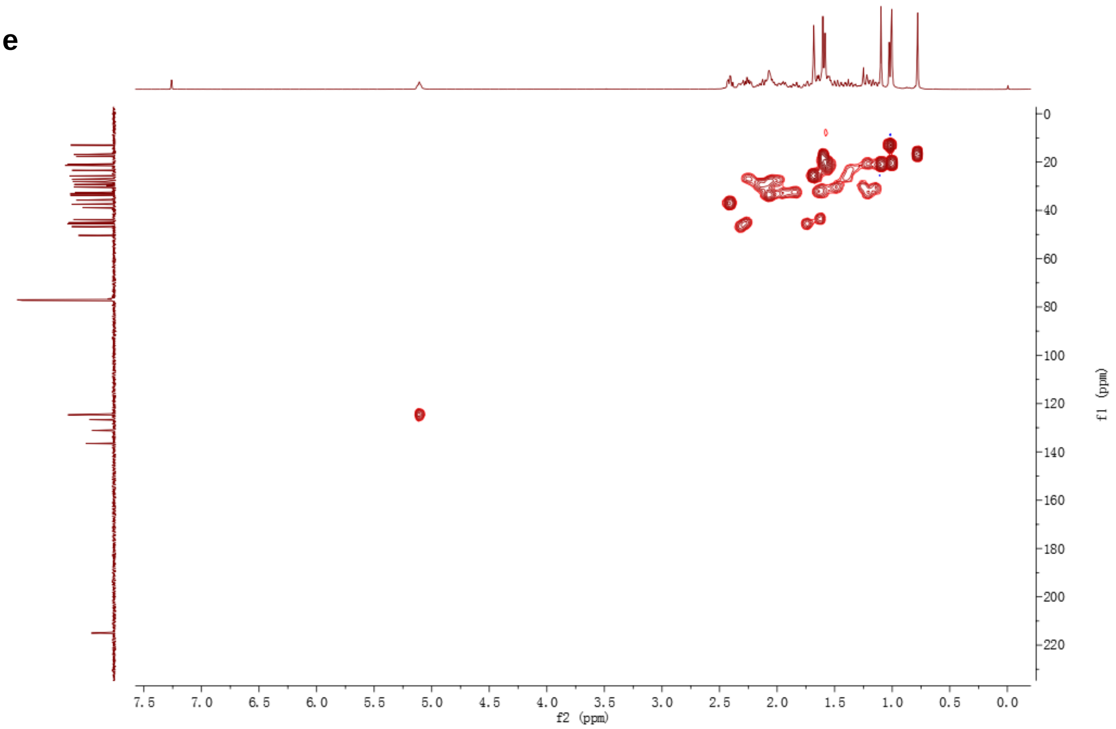

0
20
40
60
80
100
120
140
160
180
200
220
f1 (ppm)
7.5
7.0
6.5
6.0
5.5
5.0
4.5
4.0
3.5
3.0
2.5
2.0
1.5
1.0
0.5
0.0
f2 (ppm)

f

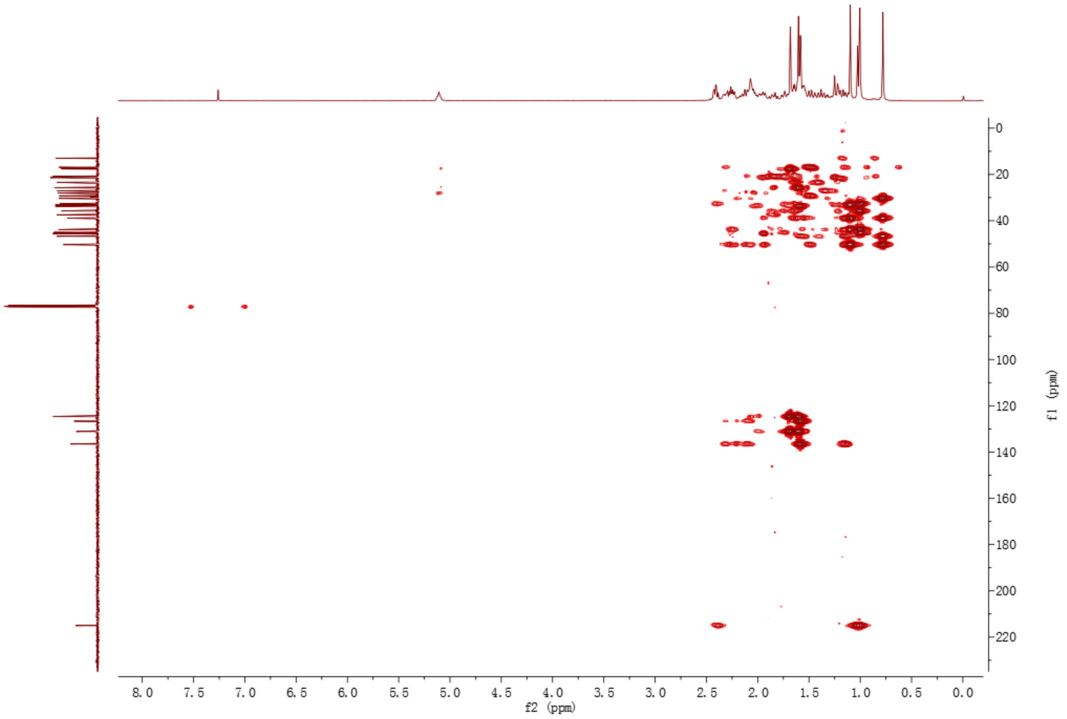

0
20
40
60
80
100
120
140
160
180
200
220
f1 (ppm)
8.0
7.5
7.0
6.5
6.0
5.5
5.0
4.5
4.0
3.5
3.0
2.5
2.0
1.5
1.0
0.5
0.0
f2 (ppm)

g

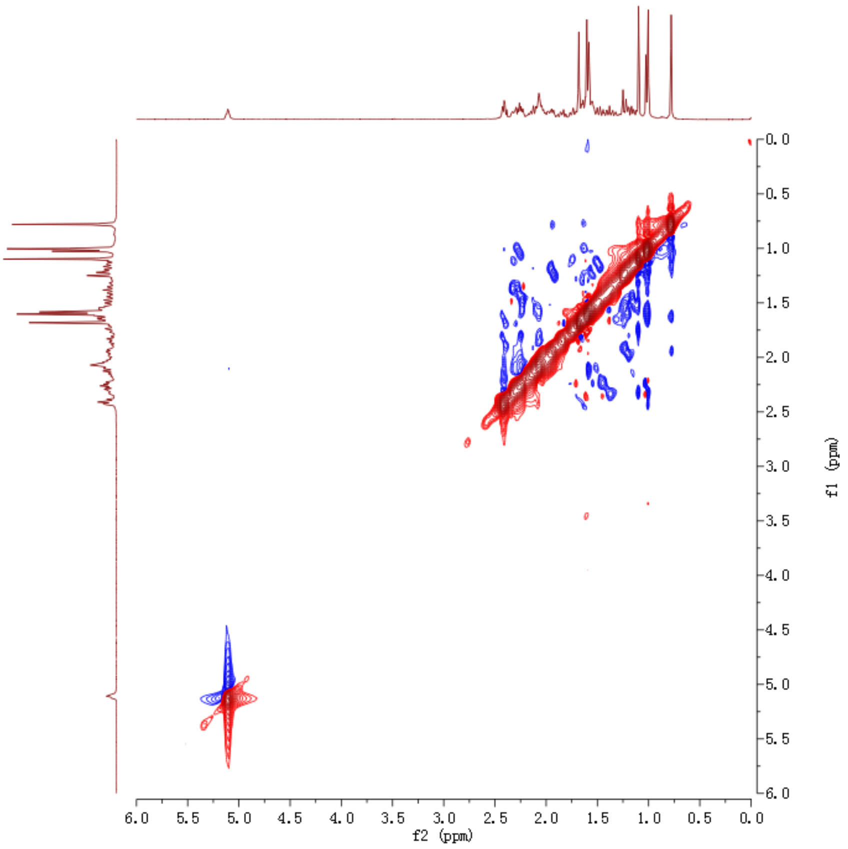

Supplementary Figure 32. HRESIMS and NMR spectra of 5

a HRESIMS spectrum; **b** ^{1}H NMR spectrum in $CDCl_3$ at 400 MHz; **c** ^{13}C NMR spectrum in $CDCl_3$ at 100 MHz; **d** ^{1}H-^{1}H COSY spectrum in $CDCl_3$ at 400 MHz; **e** HSQC spectrum in $CDCl_3$ at 400 MHz; **f** HMBC spectrum in $CDCl_3$ at 400 MHz; **g** ROESY spectrum in $CDCl_3$ at 400 MHz.

a

Single Mass Analysis
Tolerance = 5.0 mDa / DBE: min = -1.5, max = 15.0
Element prediction: Off
Number of isotope peaks used for i-FIT = 3

Monoisotopic Mass, Even Electron Ions
115 formula(e) evaluated with 1 results within limits (up to 50 closest results for each mass)
Elements Used:
C: 0-500 H: 0-1000 O: 0-200
AFU-3
2016030701-34 232 (1.870) Cm (231:233)

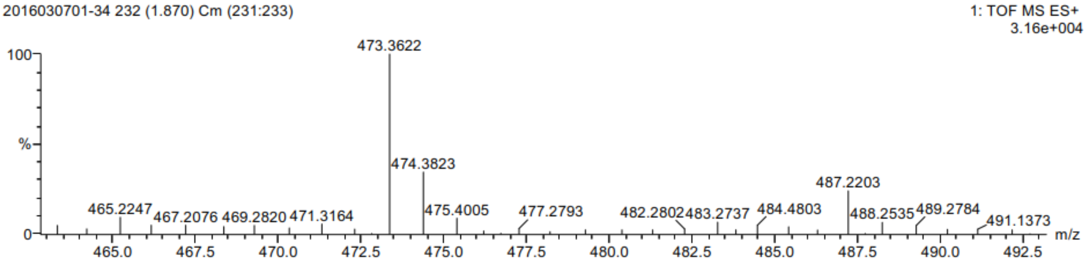

Minimum:				-1.5				
Maximum:		5.0	10.0	15.0				
Mass	Calc. Mass	mDa	PPM	DBE	i-FIT	Norm	Conf(%)	Formula
473.3622	473.3631	-0.9	-1.9	6.5	29.5	n/a	n/a	C30 H49 O4

b

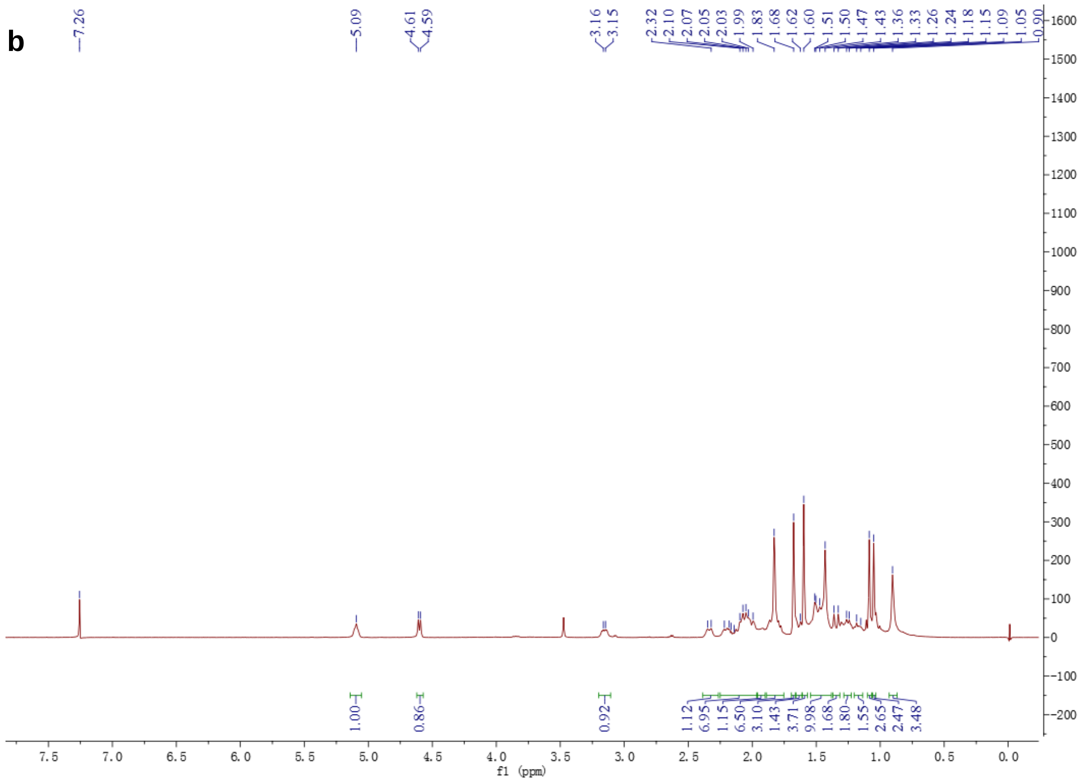

c

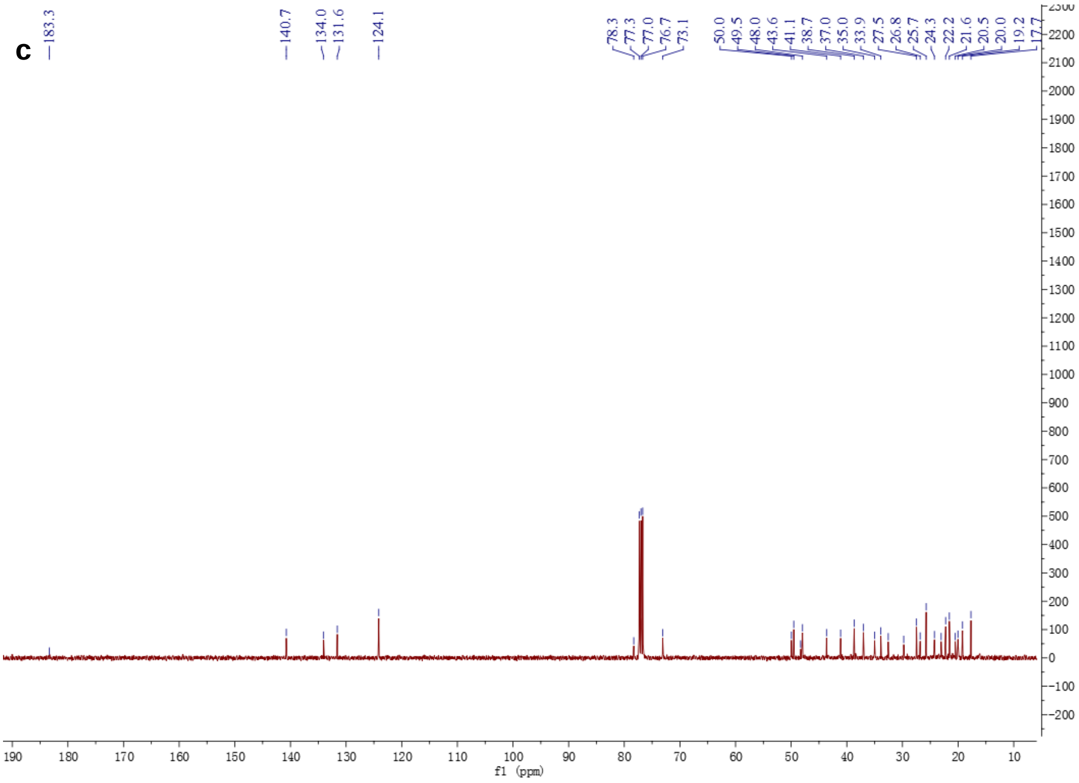
183.3
140.7
134.0
131.6
124.1
78.3
77.3
77.0
76.7
73.1
50.0
49.5
48.0
43.6
41.1
38.7
37.0
35.0
33.9
27.5
26.8
25.7
24.3
22.2
21.6
20.5
20.0
19.2
17.7
f1 (ppm)

d

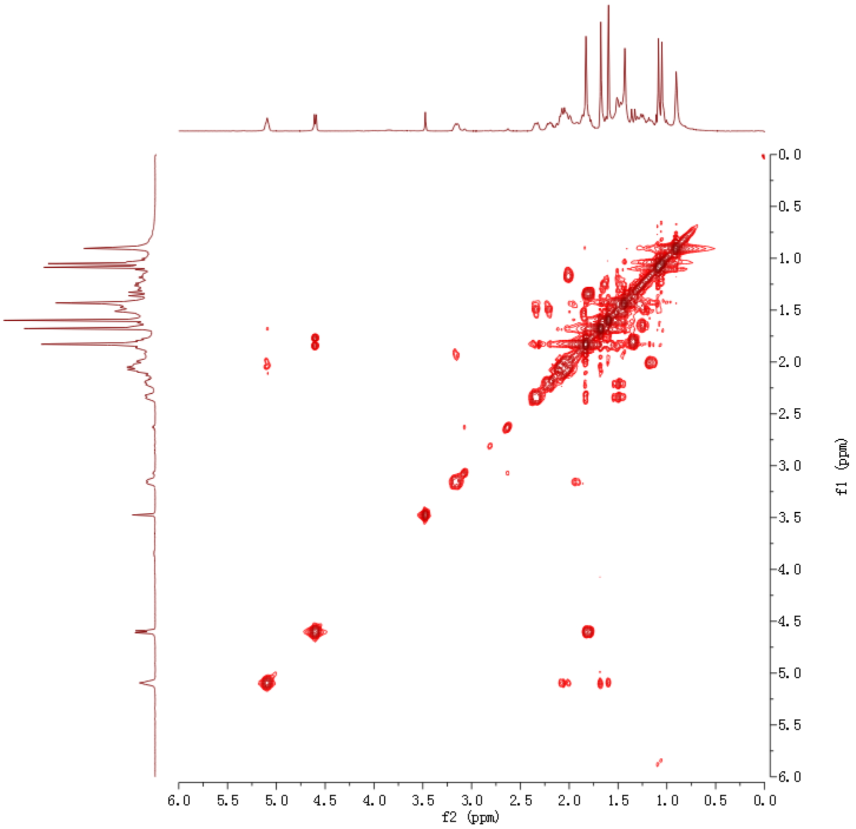
f2 (ppm)
f1 (ppm)

e

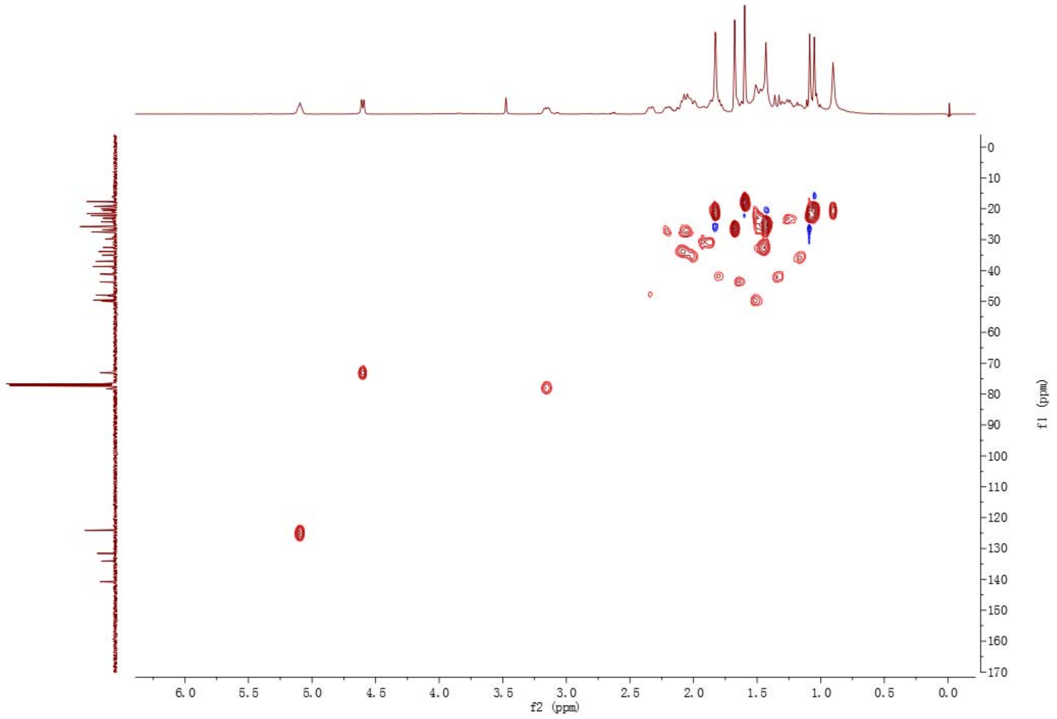

0
10
20
30
40
50
60
70
80
90
100
110
120
130
140
150
160
170
f1 (ppm)
6.0
5.5
5.0
4.5
4.0
3.5
3.0
2.5
2.0
1.5
1.0
0.5
0.0
f2 (ppm)

f

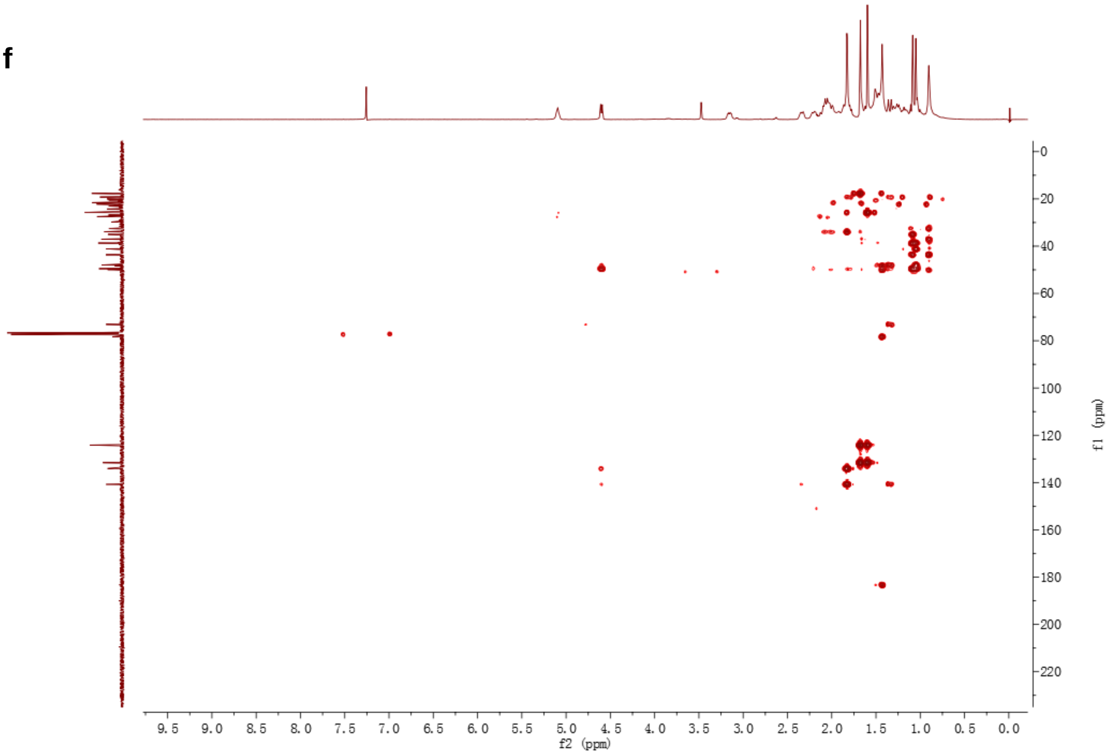

0
20
40
60
80
100
120
140
160
180
200
220
f1 (ppm)
9.5
9.0
8.5
8.0
7.5
7.0
6.5
6.0
5.5
5.0
4.5
4.0
3.5
3.0
2.5
2.0
1.5
1.0
0.5
0.0
f2 (ppm)

g

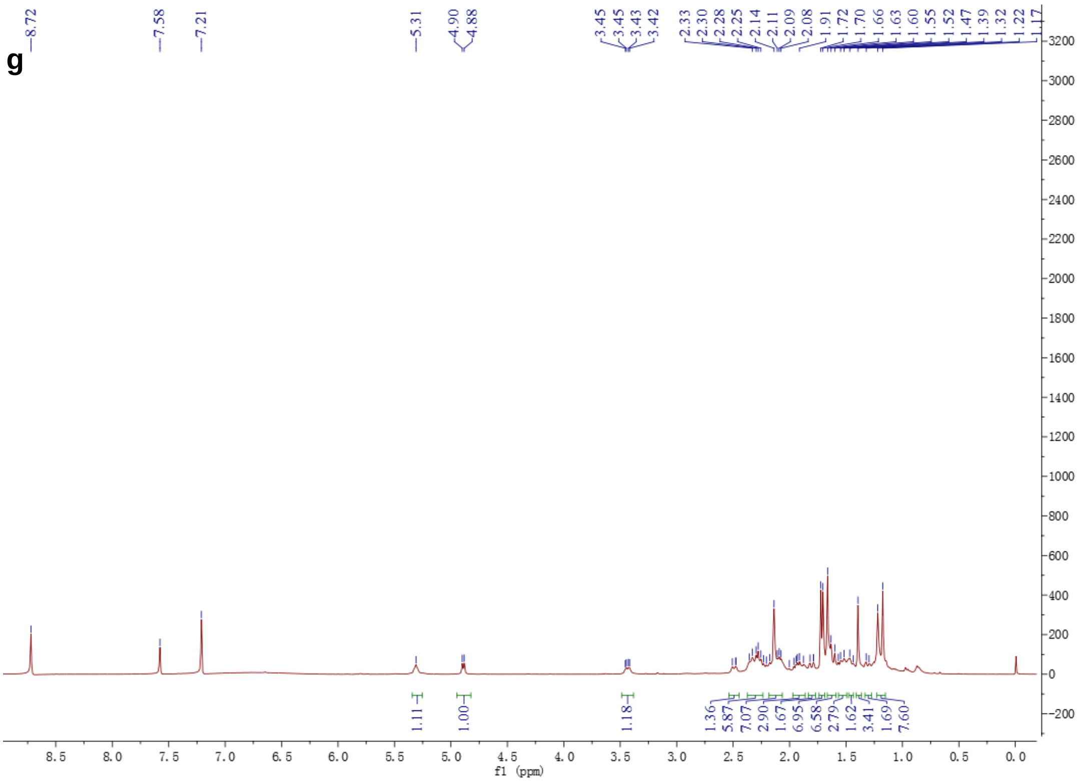

8.72
7.58
7.21
5.31
4.90
4.88
3.45
3.45
3.43
3.42
2.33
2.30
2.28
2.25
2.14
2.11
2.09
2.08
1.91
1.72
1.70
1.66
1.63
1.60
1.55
1.52
1.47
1.39
1.32
1.22
1.17
1.11
1.00
1.18
1.36
5.87
7.07
2.90
1.67
6.95
6.58
2.79
1.62
3.41
1.69
7.60
3200
3000
2800
2600
2400
2200
2000
1800
1600
1400
1200
1000
800
600
400
200
0
-200
8.5
8.0
7.5
7.0
6.5
6.0
5.5
5.0
4.5
4.0
3.5
3.0
2.5
2.0
1.5
1.0
0.5
0.0
f1 (ppm)

h

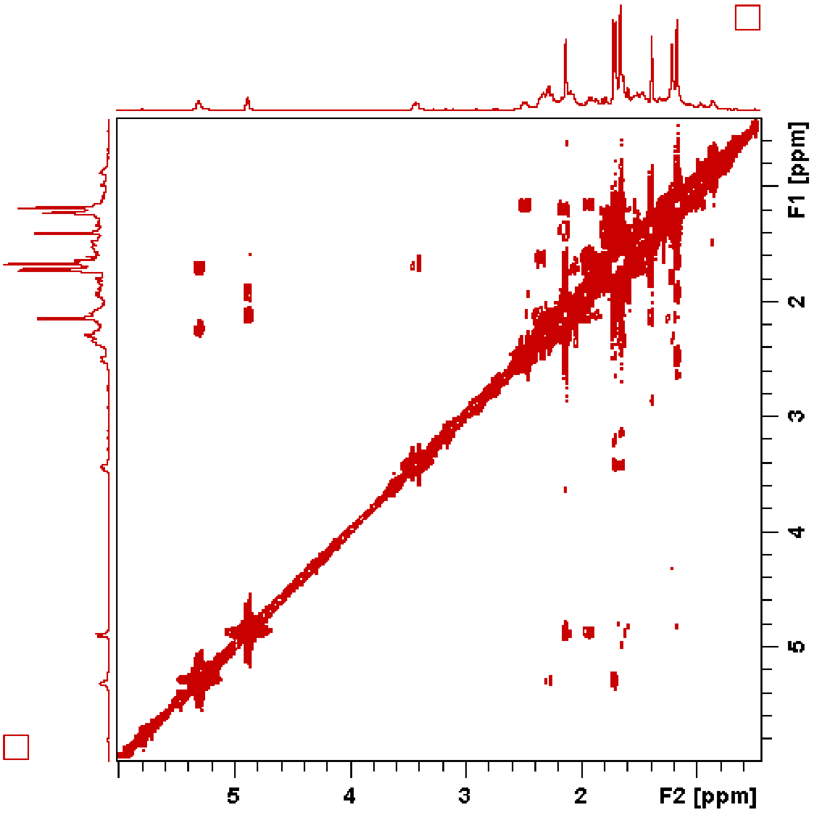

F1 [ppm]
2
3
4
5
5
4
3
2
F2 [ppm]

i

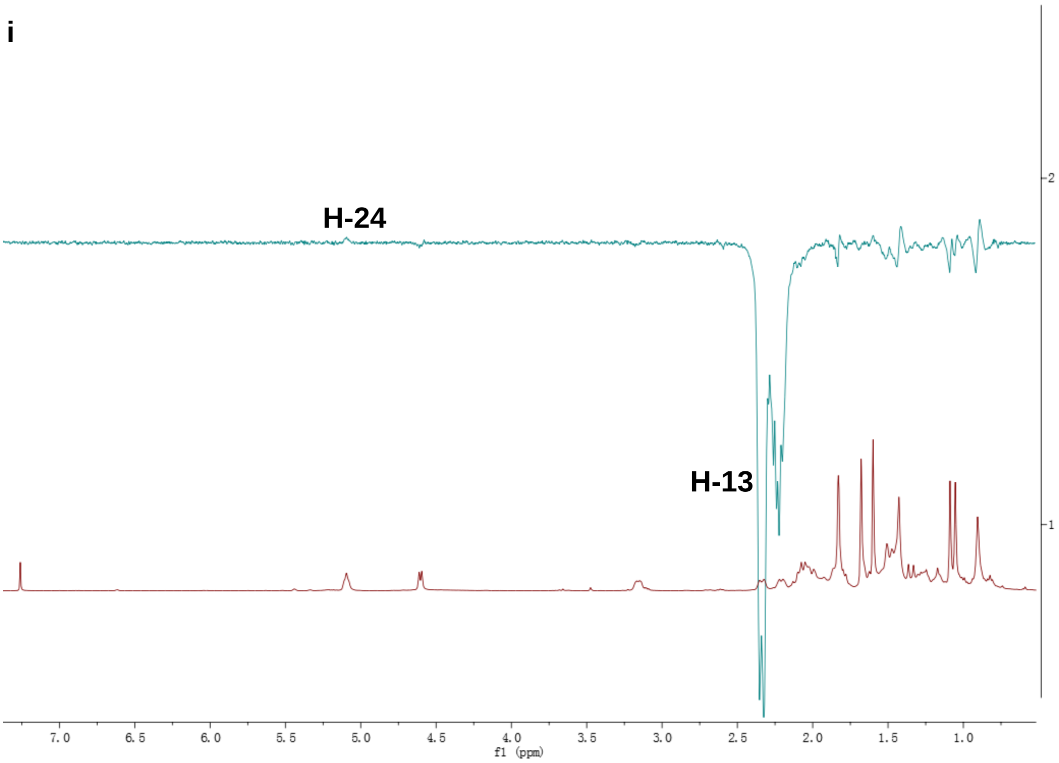

Supplementary Figure 33. HRESIMS and NMR spectra of 6

a HRESIMS spectrum; **b** ^{1}H NMR spectrum in $CDCl_3$ at 400 MHz; **c** ^{13}C NMR spectrum in $CDCl_3$ at 100 MHz; **d** ^{1}H-^{1}H COSY spectrum in $CDCl_3$ at 400 MHz; **e** HSQC spectrum in $CDCl_3$ at 400 MHz; **f** HMBC spectrum in $CDCl_3$ at 400 MHz; **g** ^{1}H NMR spectrum in pyridine-d_5 at 400 MHz; **h** NOESY spectrum in pyridine-d_5 at 400 MHz; **i** 1D-selective NOE experiment in $CDCl_3$ at 400 MHz.

a

Single Mass Analysis

Tolerance = 10.0 PPM / DBE: min = -1.5, max = 50.0
Element prediction: Off
Number of isotope peaks used for i-FIT = 3

Monoisotopic Mass, Even Electron Ions
100 formula(e) evaluated with 1 results within limits (up to 20 best isotopic matches for each mass)
Elements Used:
C: 0-85 H: 0-130 O: 0-70
AFU-2
201601110026 263 (2.124)

1: TOF MS ES+
1.70e+004

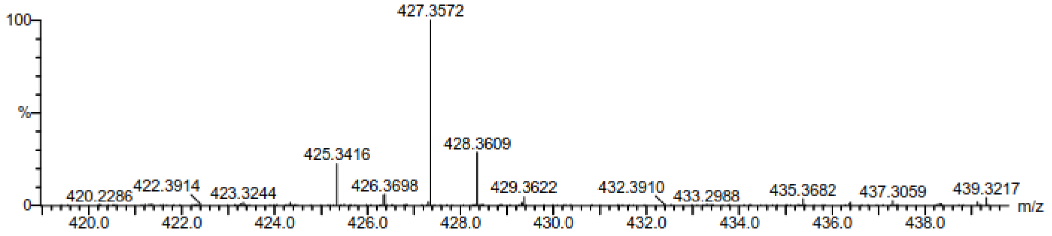

Minimum:				-1.5				
Maximum:		5.0	10.0	50.0				
Mass	Calc. Mass	mDa	PPM	DBE	i-FIT	Norm	Conf(%)	Formula
427.3572	427.3576	-0.4	-0.9	6.5	154.2	n/a	n/a	C29 H47 O2

b

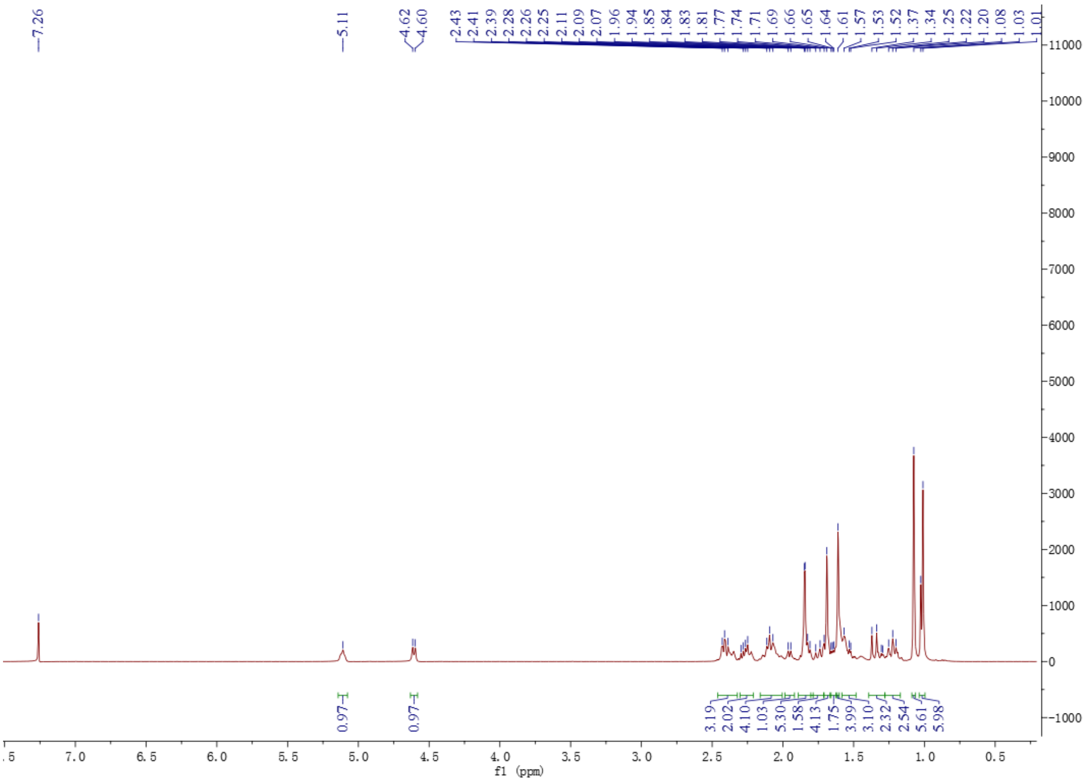

c

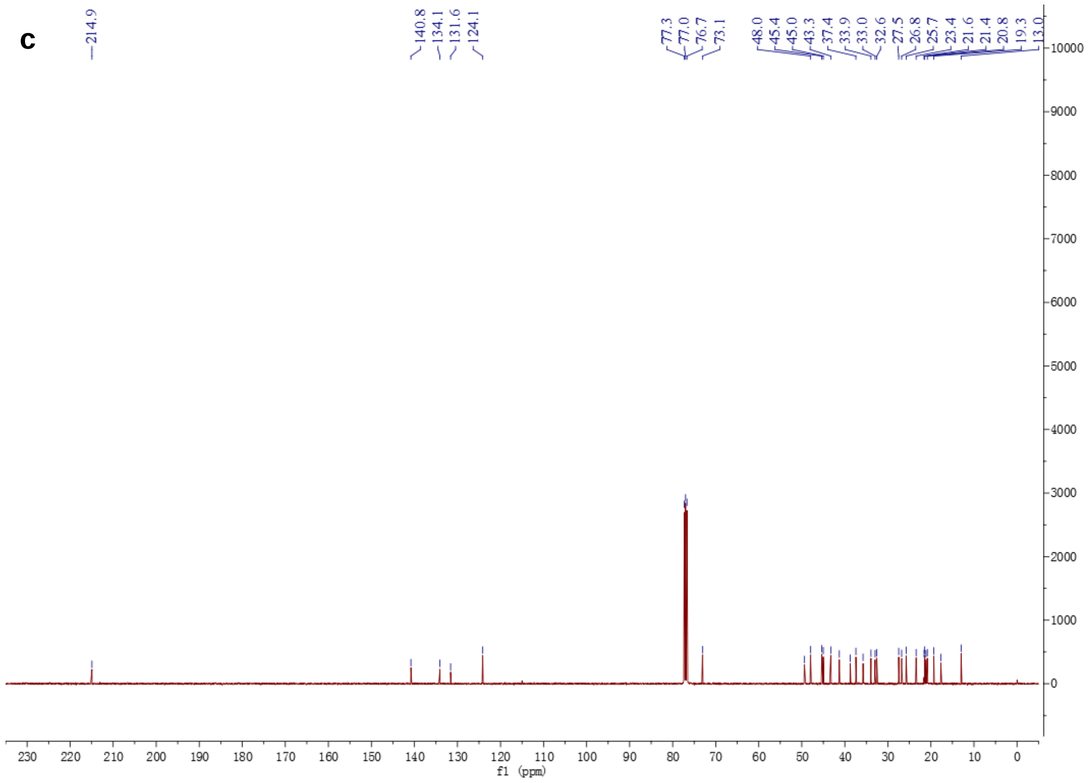

214.9
140.8
134.1
131.6
124.1
77.3
77.0
76.7
73.1
48.0
45.4
45.0
43.3
37.4
33.9
33.0
32.6
27.5
26.8
25.7
23.4
21.6
21.4
20.8
19.3
13.0
f1 (ppm)

d

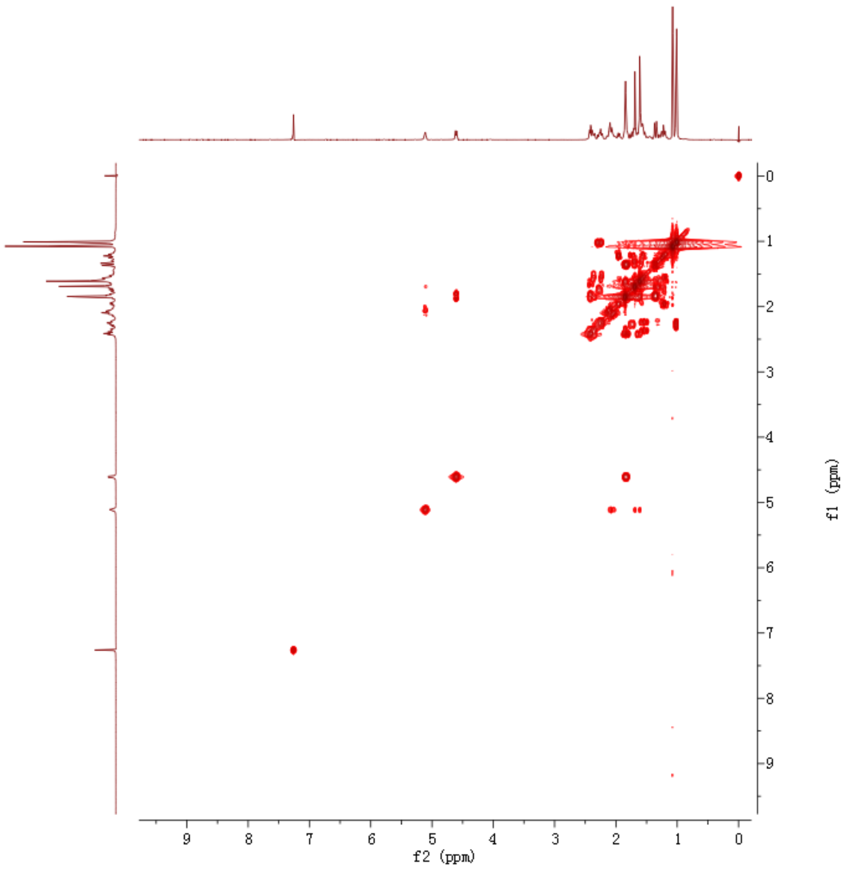

f2 (ppm)
f1 (ppm)

e

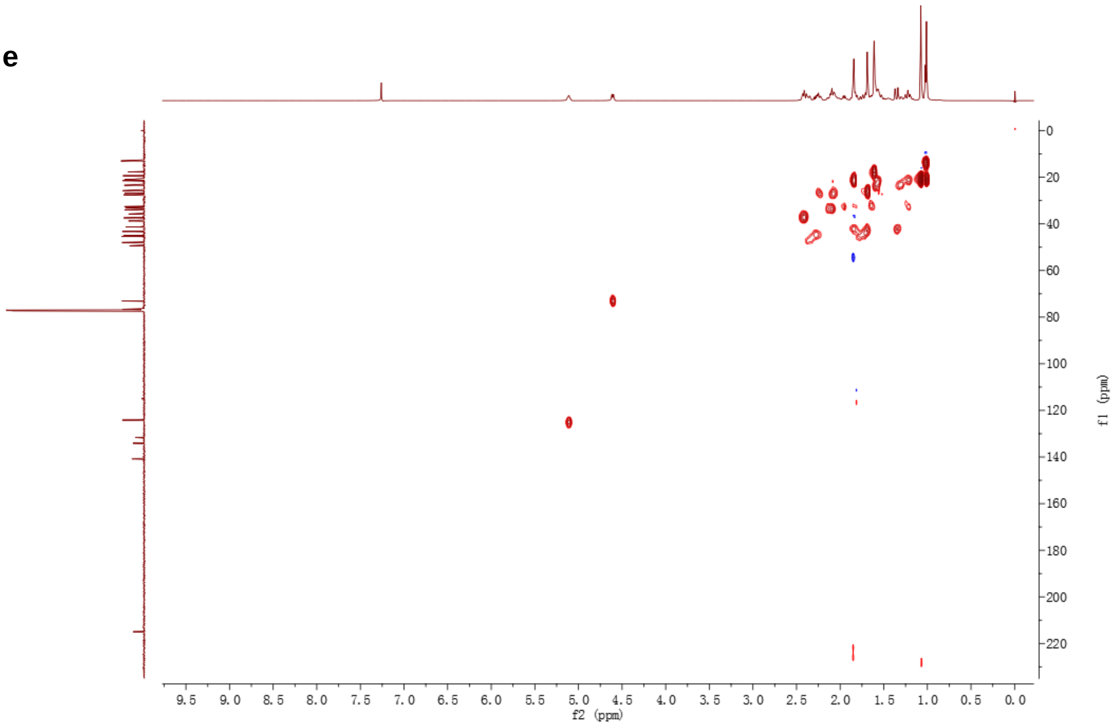

f

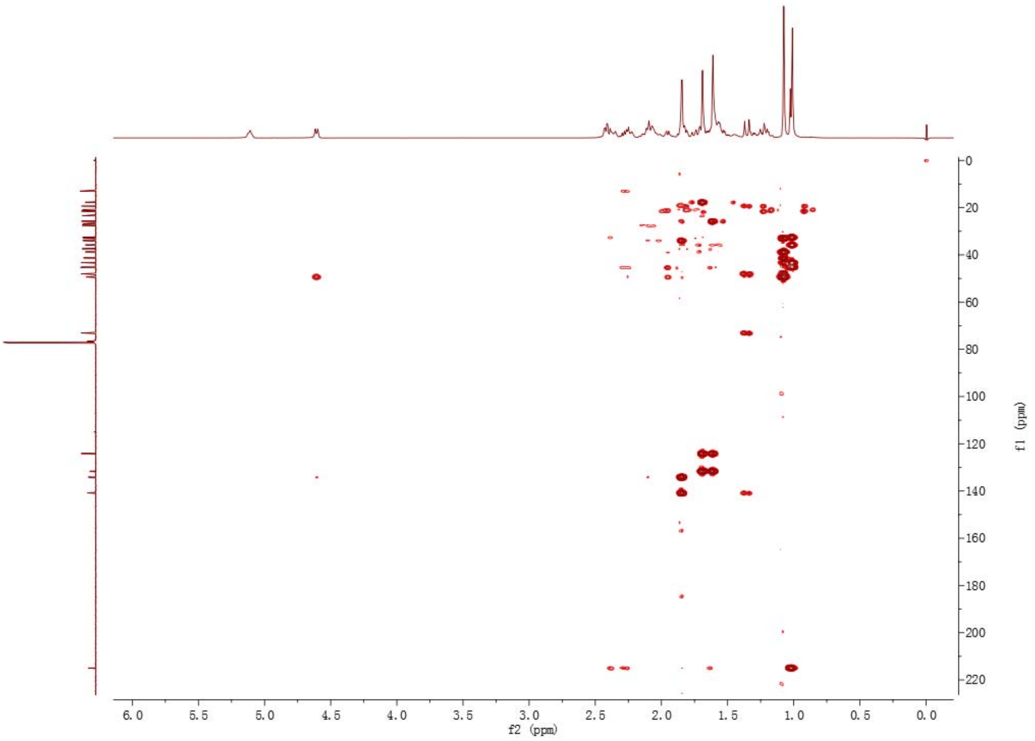

g

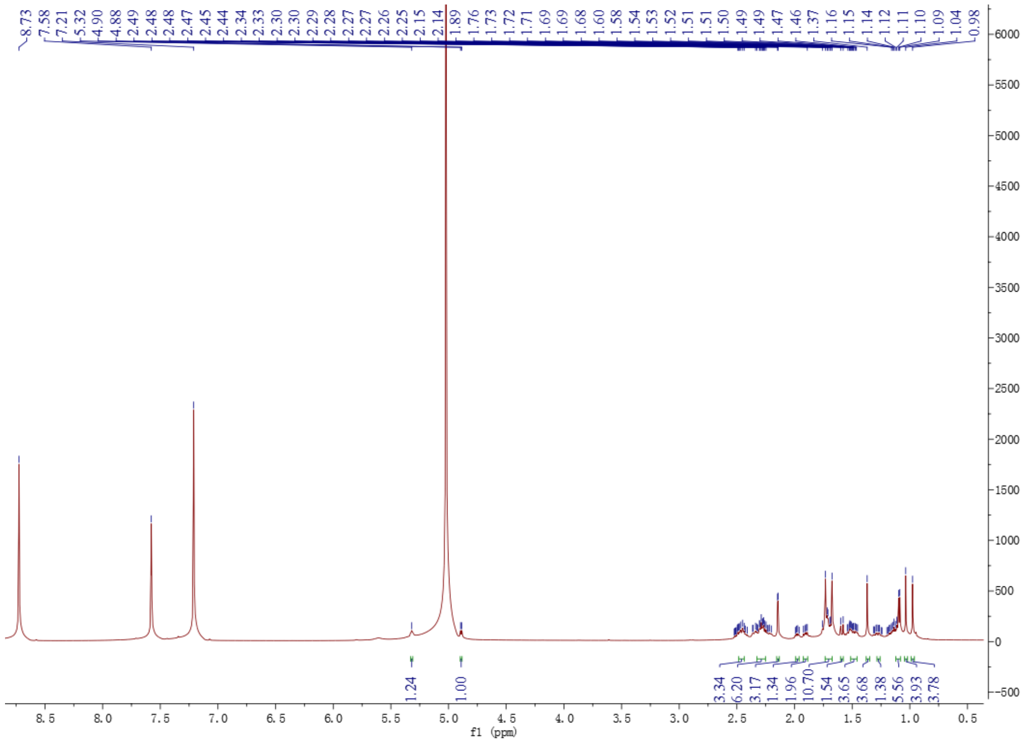

h

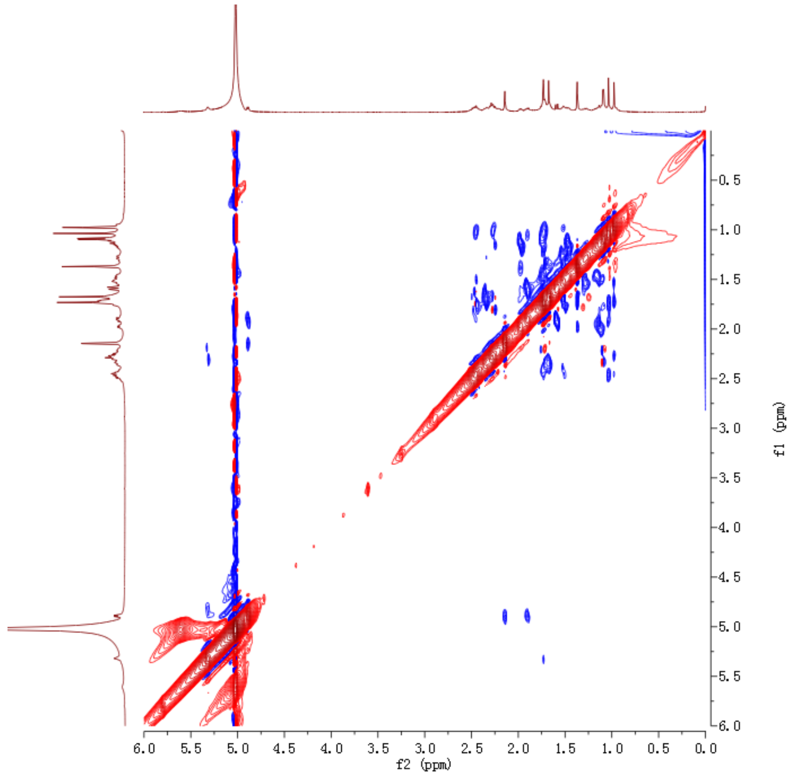

Supplementary Figure 34. HRESIMS and NMR spectra of 7

a HRESIMS spectrum; **b** ^{1}H NMR spectrum in $CDCl_3$ at 400 MHz; **c** ^{13}C NMR spectrum in $CDCl_3$ at 100 MHz; **d** ^{1}H-^{1}H COSY spectrum in $CDCl_3$ at 400 MHz; **e** HSQC spectrum in $CDCl_3$ at 400 MHz; **f** HMBC spectrum in $CDCl_3$ at 400 MHz; **g** ^{1}H NMR spectrum in pyridine-d_5 at 600 MHz; **h** ROESY spectrum in pyridine-d_5 at 600 MHz.

a

Single Mass Analysis
Tolerance = 5.0 mDa / DBE: min = -1.5, max = 15.0
Element prediction: Off
Number of isotope peaks used for i-FIT = 3

Monoisotopic Mass, Even Electron Ions
272 formula(e) evaluated with 2 results within limits (up to 50 closest results for each mass)
Elements Used:
C: 0-500 H: 0-1000 O: 0-200 Na: 0-1

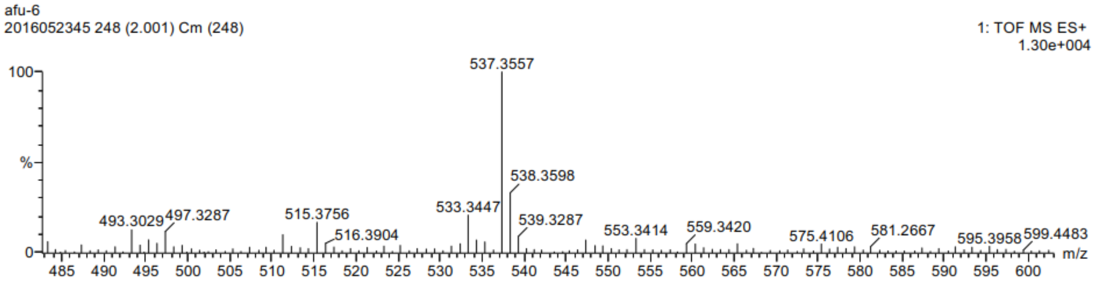

Minimum:				-1.5				
Maximum:		5.0	10.0	15.0				
Mass	Calc. Mass	mDa	PPM	DBE	i-FIT	Norm	Conf(%)	Formula
537.3557	537.3556	0.1	0.2	7.5	26.3	0.508	60.17	C32 H50 O5 Na
	537.3580	-2.3	-4.3	10.5	26.7	0.921	39.83	C34 H49 O5

b

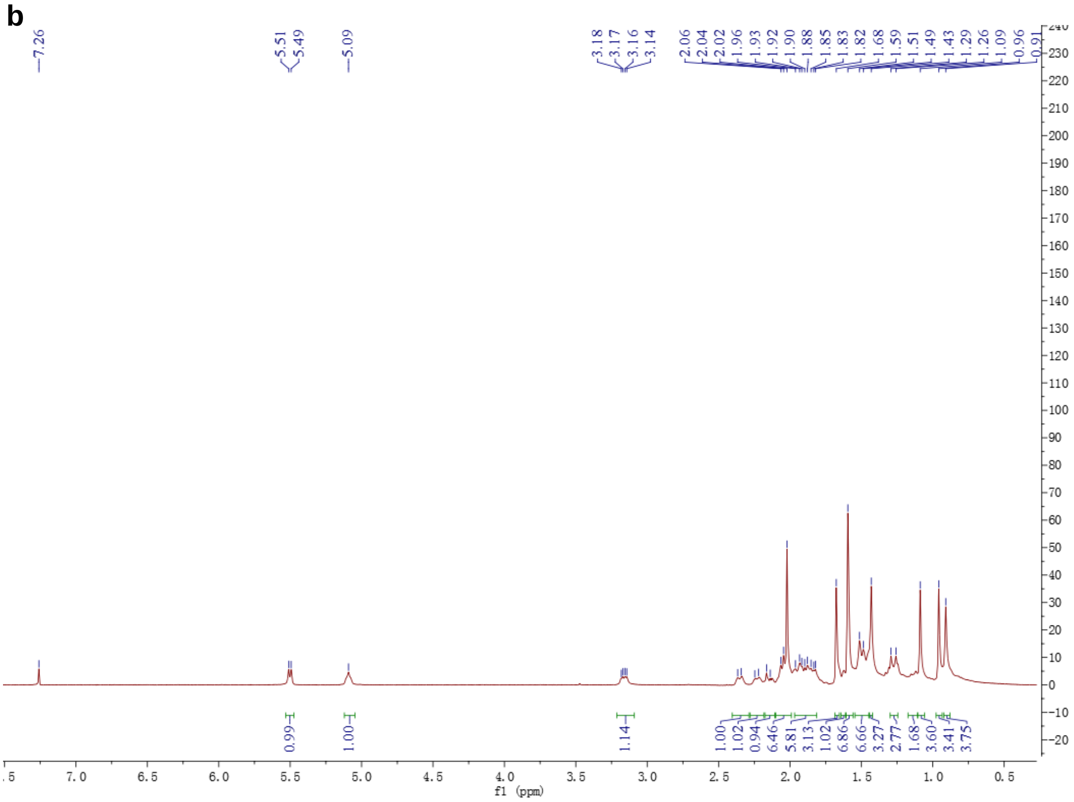

c

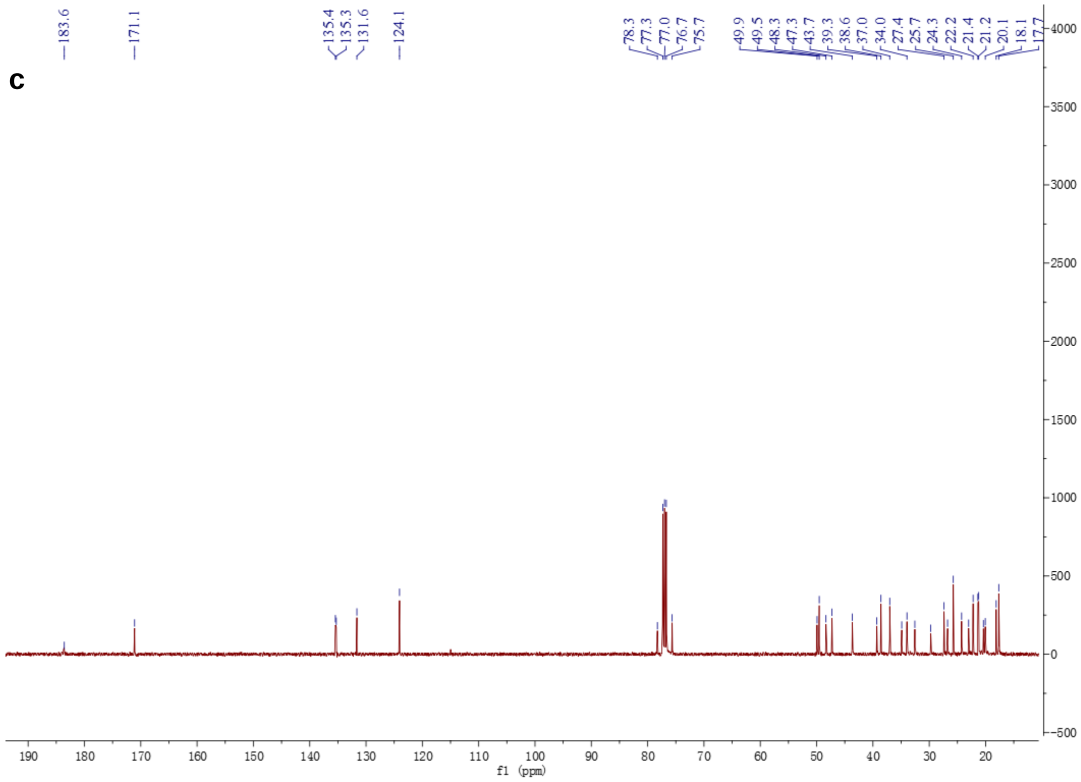
183.6
171.1
135.4
135.3
131.6
124.1
78.3
77.3
77.0
76.7
75.7
49.9
49.5
48.3
47.3
43.7
39.3
38.6
37.0
34.0
27.4
25.7
24.3
22.2
21.4
21.2
20.1
18.1
17.7
f1 (ppm)

d

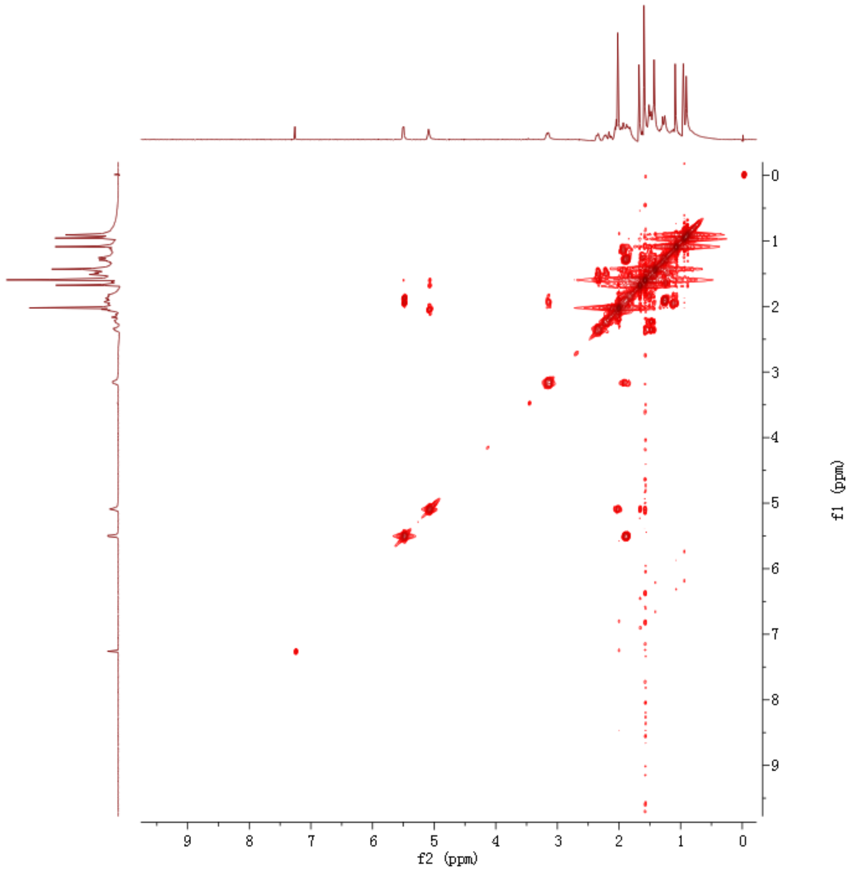
f2 (ppm)
f1 (ppm)

e

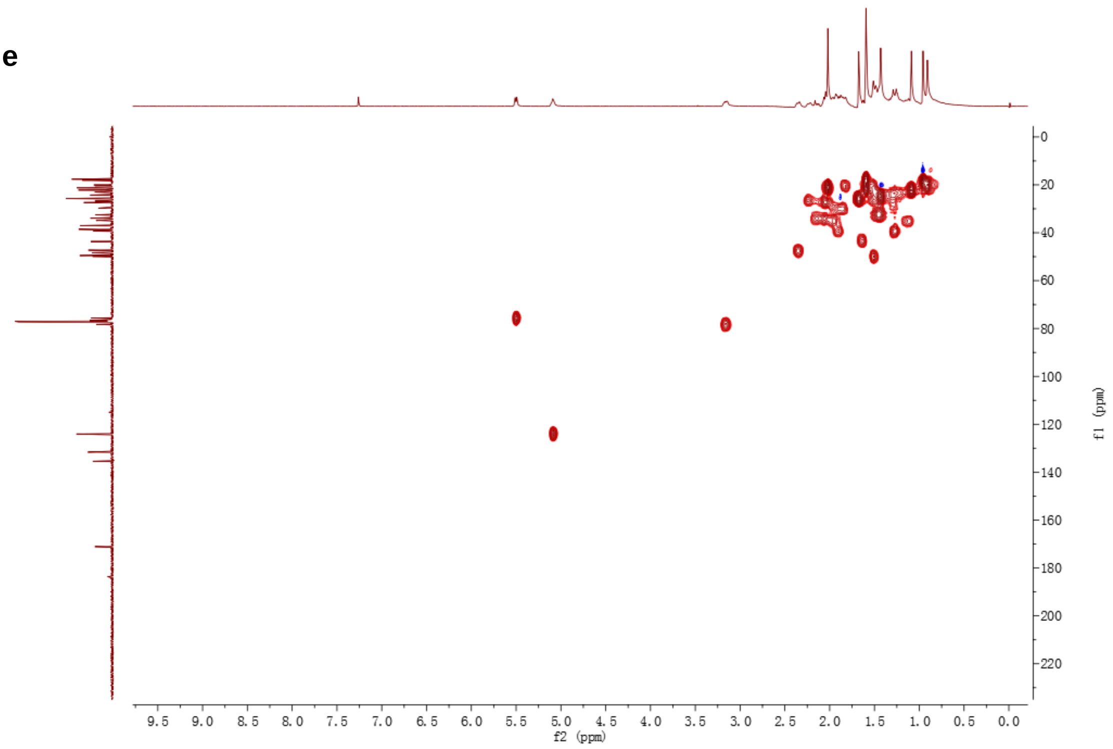

f

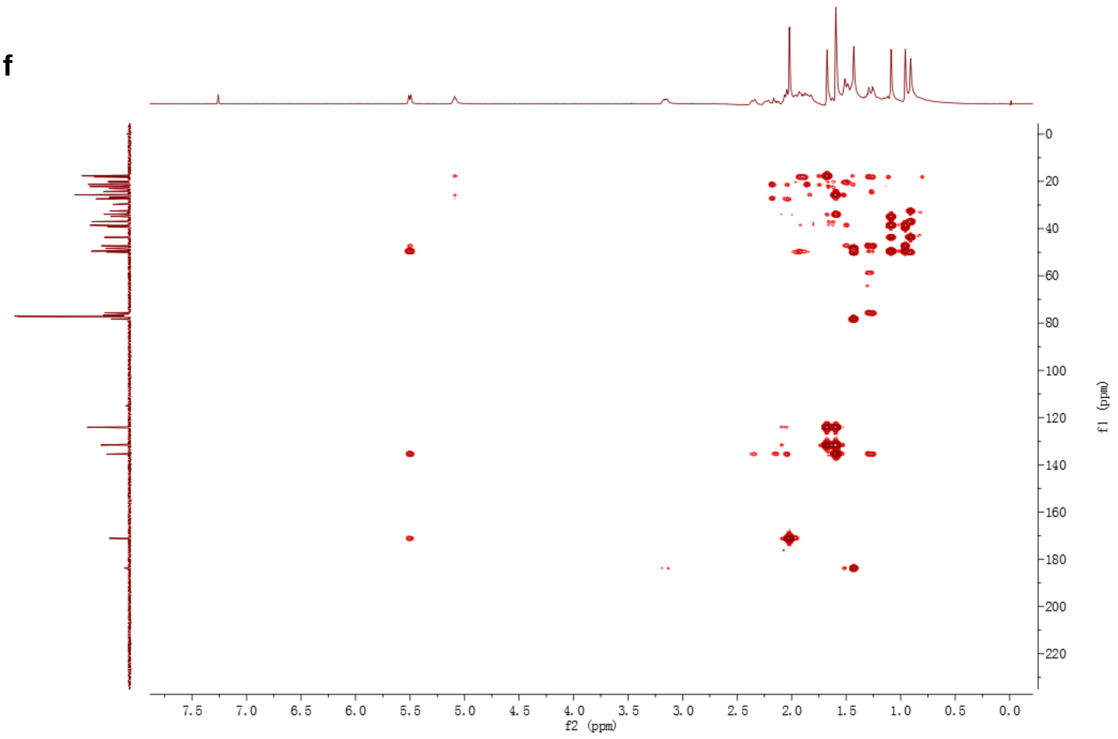

g

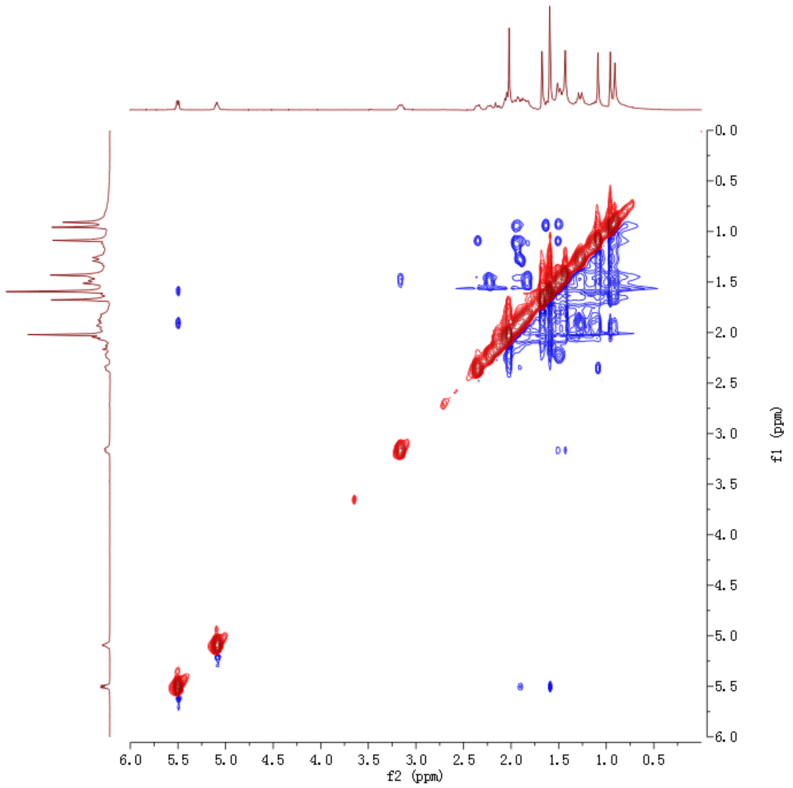

h

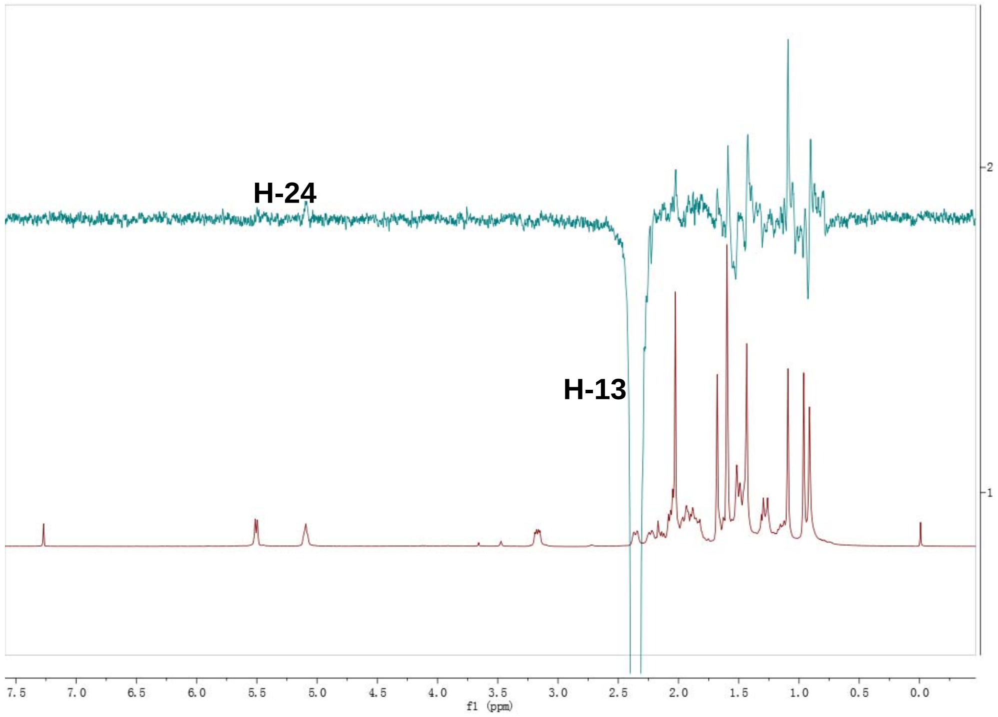

Supplementary Figure 35. HRESIMS and NMR spectra of 8

a HRESIMS spectrum; **b** ^{1}H NMR spectrum in $CDCl_3$ at 400 MHz; **c** ^{13}C NMR spectrum in $CDCl_3$ at 100 MHz; **d** ^{1}H-^{1}H COSY spectrum in $CDCl_3$ at 400 MHz; **e** HSQC spectrum in $CDCl_3$ at 400 MHz; **f** HMBC spectrum in $CDCl_3$ at 400 MHz; **g** ROESY spectrum in $CDCl_3$ at 400 MHz; **h** 1D-selective NOE experiment in $CDCl_3$ at 400 MHz.

a

Single Mass Analysis
Tolerance = 5.0 mDa / DBE: min = -1.5, max = 15.0
Element prediction: Off
Number of isotope peaks used for i-FIT = 3

Monoisotopic Mass, Even Electron Ions
240 formula(e) evaluated with 1 results within limits (up to 50 closest results for each mass)
Elements Used:
C: 0-500 H: 0-1000 O: 0-200 Na: 0-1

AFU-4
2016041819 282 (2.277)

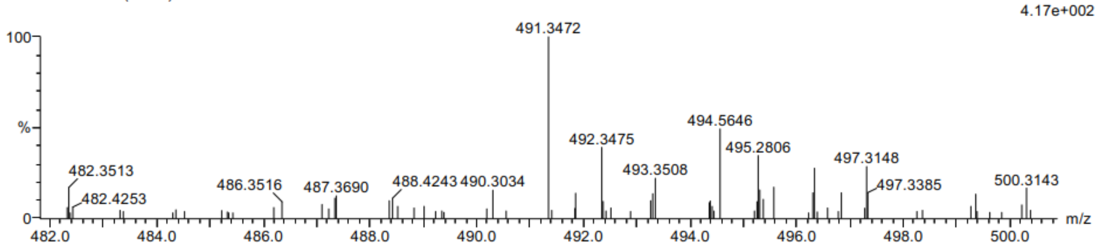

Minimum:				-1.5				
Maximum:		5.0	10.0	15.0				
Mass	Calc. Mass	mDa	PPM	DBE	i-FIT	Norm	Conf(%)	Formula
491.3472	491.3501	-2.9	-5.9	7.5	74.6	n/a	n/a	C31 H48 O3 Na

b

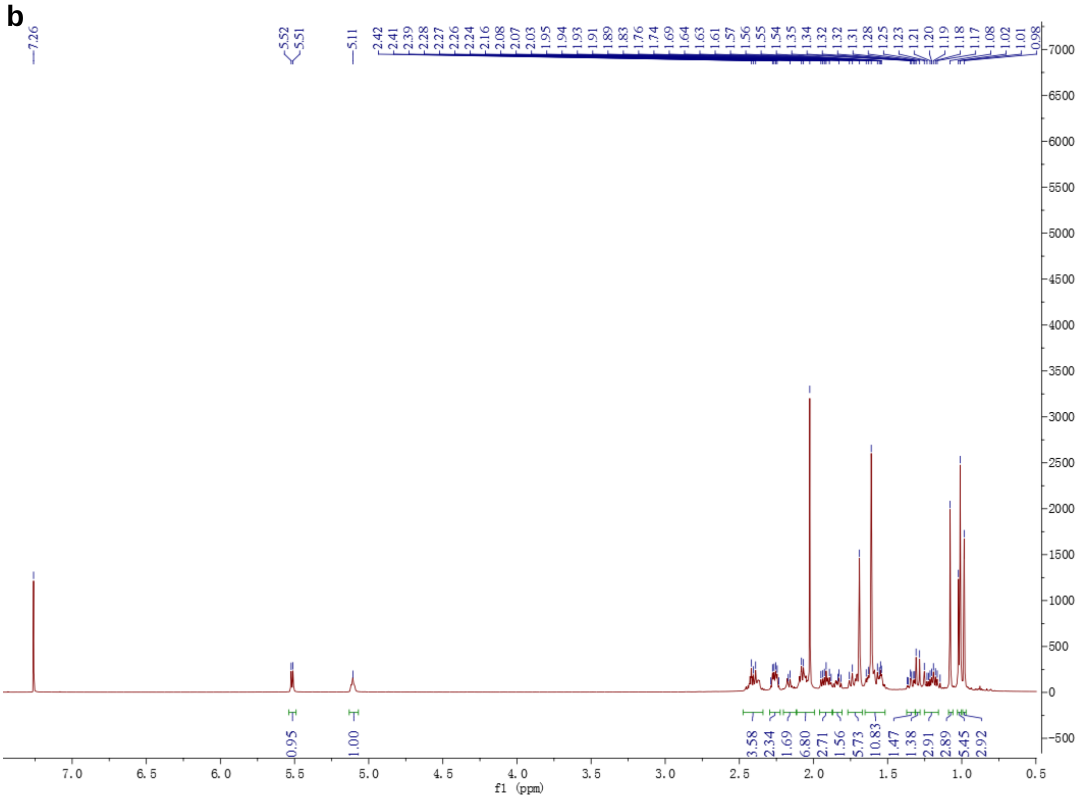

c

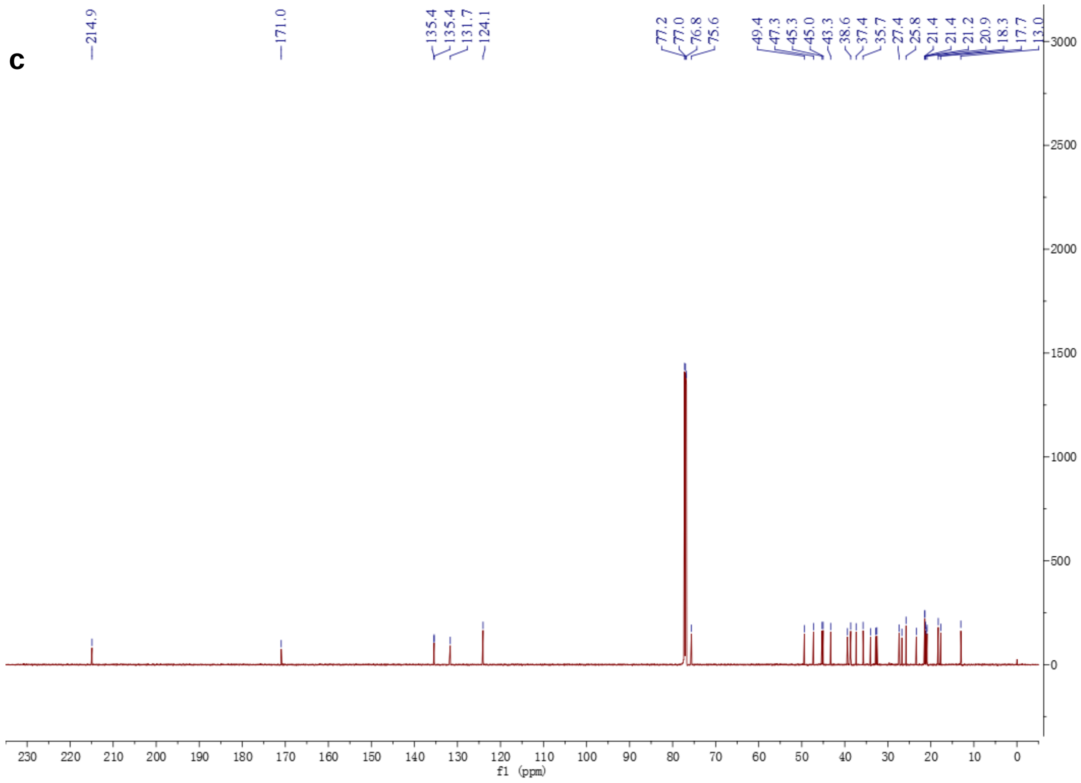
214.9
171.0
135.4
135.4
131.7
124.1
77.2
77.0
76.8
75.6
49.4
47.3
45.3
45.0
43.3
38.6
37.4
35.7
27.4
25.8
21.4
21.4
21.2
20.9
18.3
17.7
13.0
f1 (ppm)

d

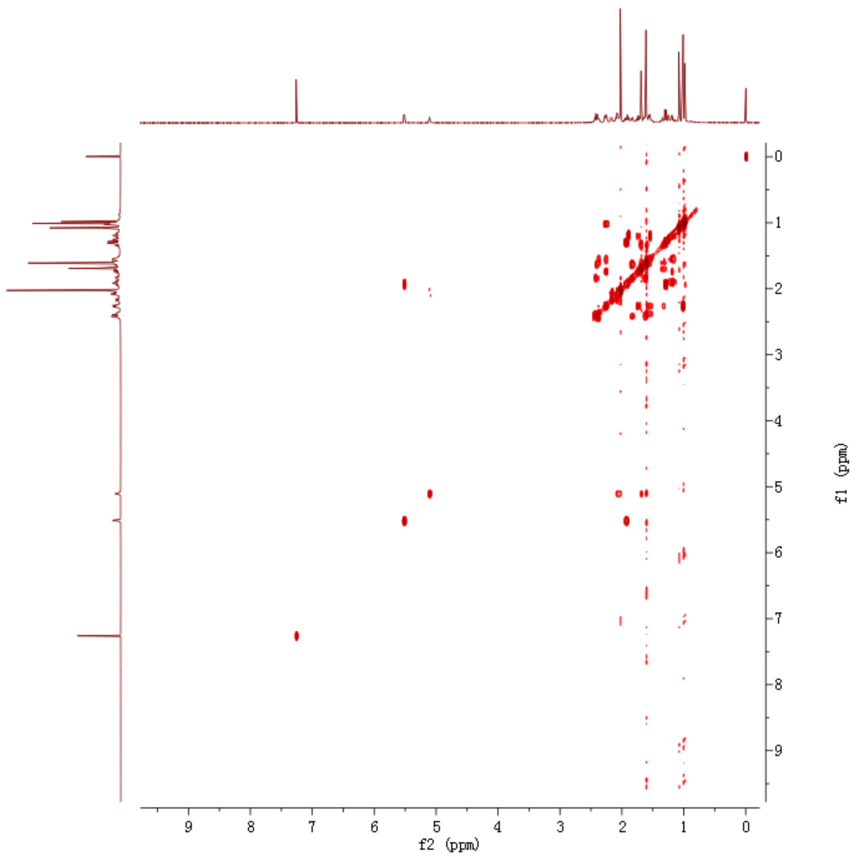
f2 (ppm)
f1 (ppm)

e

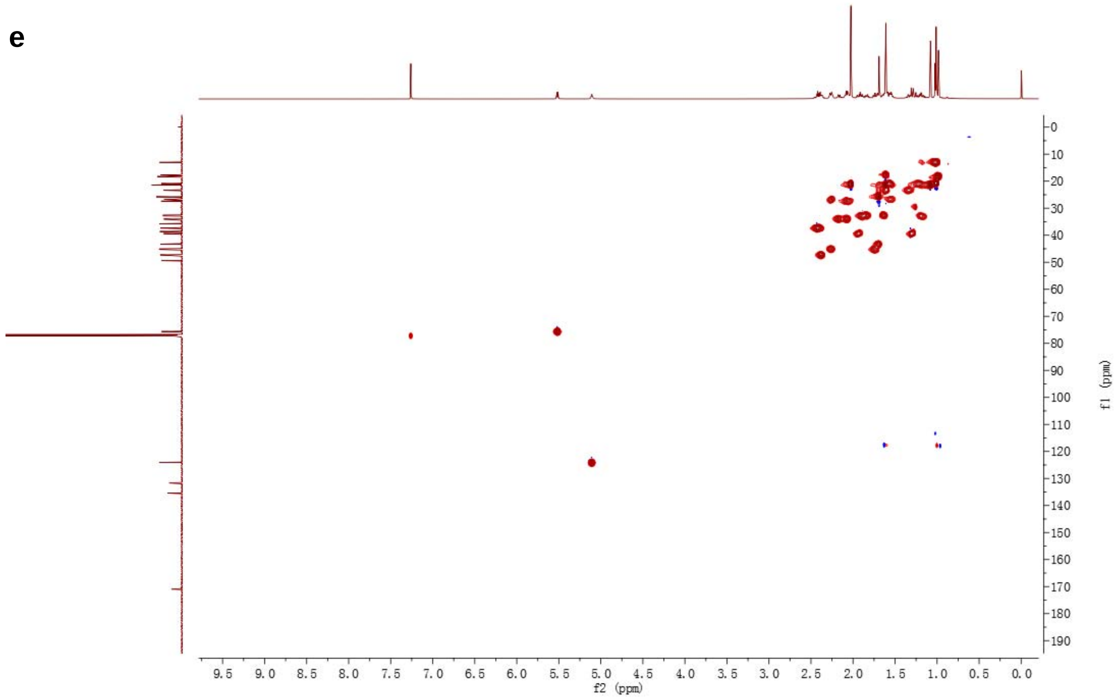

f

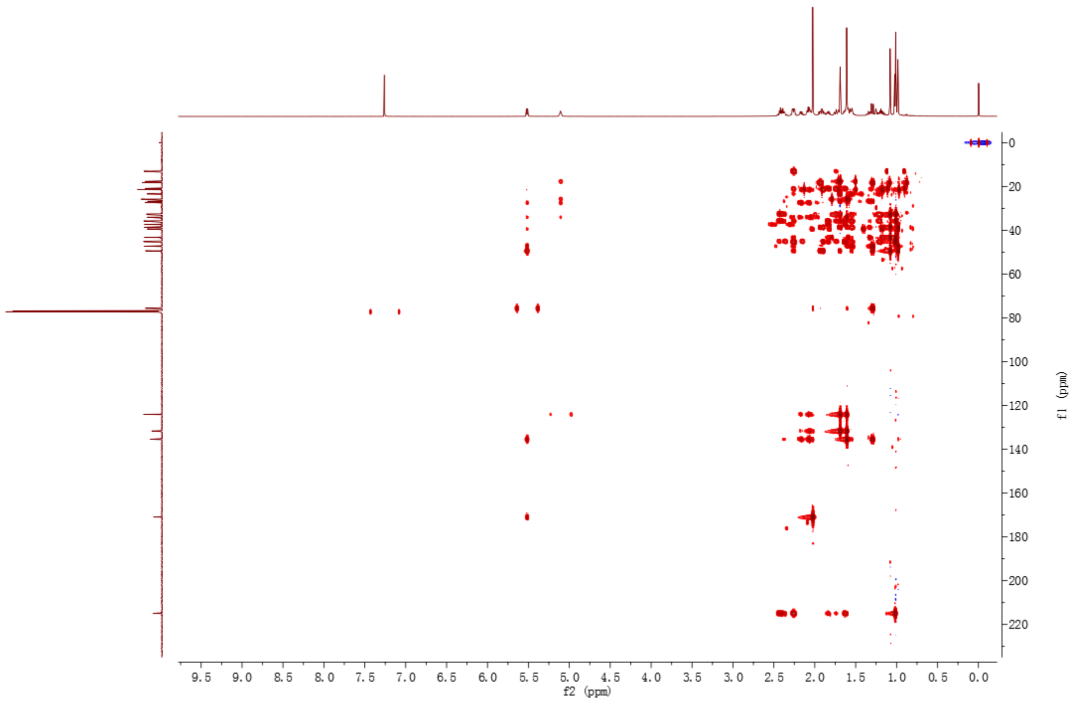

g

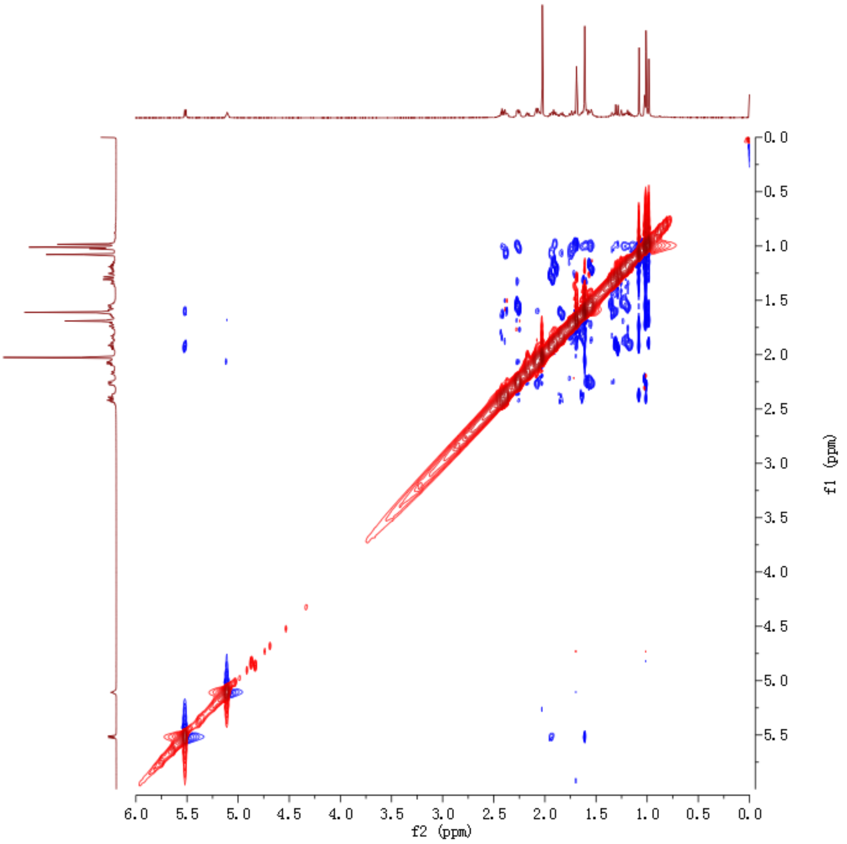

Supplementary Figure 36. HRESIMS and NMR spectra of 9

a HRESIMS spectrum; **b** ^{1}H NMR spectrum in $CDCl_3$ at 600 MHz; **c** ^{13}C NMR spectrum in $CDCl_3$ at 150 MHz; **d** ^{1}H-^{1}H COSY spectrum in $CDCl_3$ at 600 MHz; **e** HSQC spectrum in $CDCl_3$ at 600 MHz; **f** HMBC spectrum in $CDCl_3$ at 600 MHz; **g** ROESY spectrum in $CDCl_3$ at 600 MHz.

a

Single Mass Analysis
Tolerance = 10.0 PPM / DBE: min = -1.5, max = 50.0
Element prediction: Off
Number of isotope peaks used for i-FIT = 3

Monoisotopic Mass, Even Electron Ions
217 formula(e) evaluated with 2 results within limits (up to 20 best isotopic matches for each mass)
Elements Used:
C: 0-800 H: 0-200 O: 0-100 Na: 0-1
AFU-11
2016082226 221 (1.788) 1: TOF MS ES+ 4.80e+003

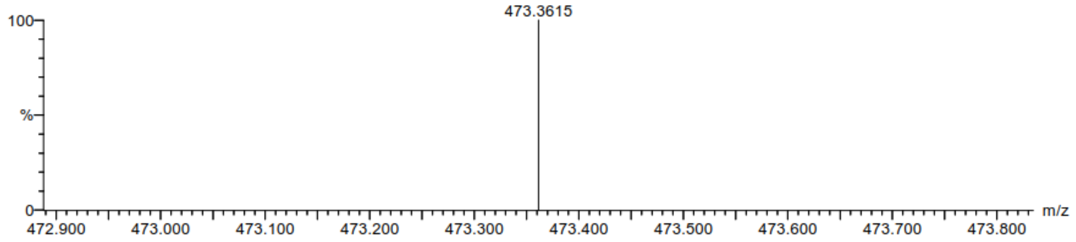

Minimum:				-1.5				
Maximum:		5.0	10.0	50.0				
Mass	Calc. Mass	mDa	PPM	DBE	i-FIT	Norm	Conf(%)	Formula
473.3615	473.3607	0.8	1.7	3.5	28.3	0.544	58.04	C28 H50 O4 Na
	473.3631	-1.6	-3.4	6.5	28.6	0.869	41.96	C30 H49 O4

b

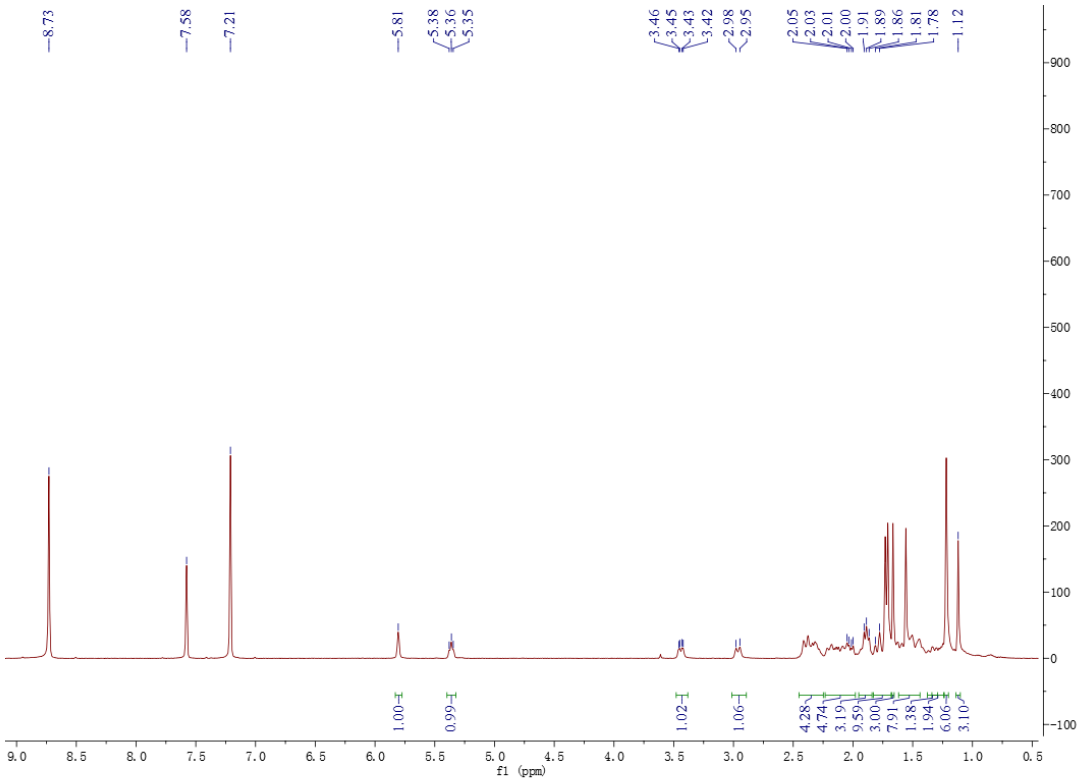

c

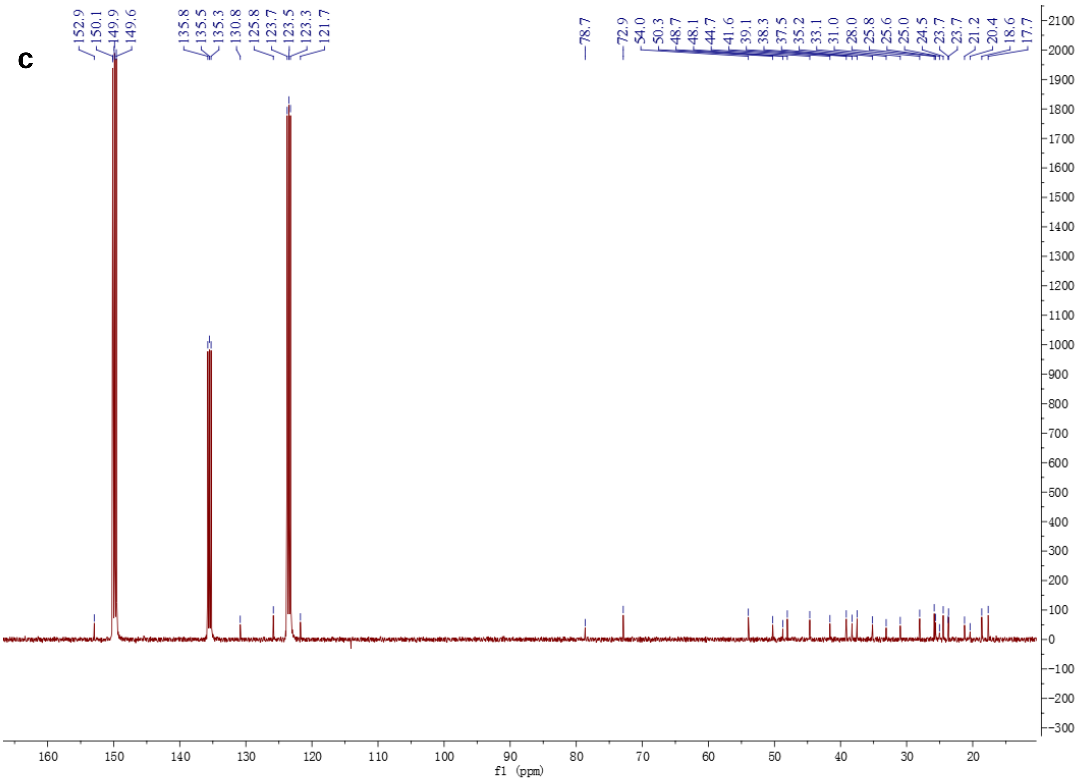
152.9
150.1
149.9
149.6
135.8
135.5
135.3
130.8
125.8
123.7
123.5
123.3
121.7
78.7
72.9
54.0
50.3
48.7
48.1
44.7
41.6
39.1
38.3
37.5
35.2
33.1
31.0
28.0
25.8
25.6
25.0
24.5
23.7
23.7
21.2
20.4
18.6
17.7
f1 (ppm)

d

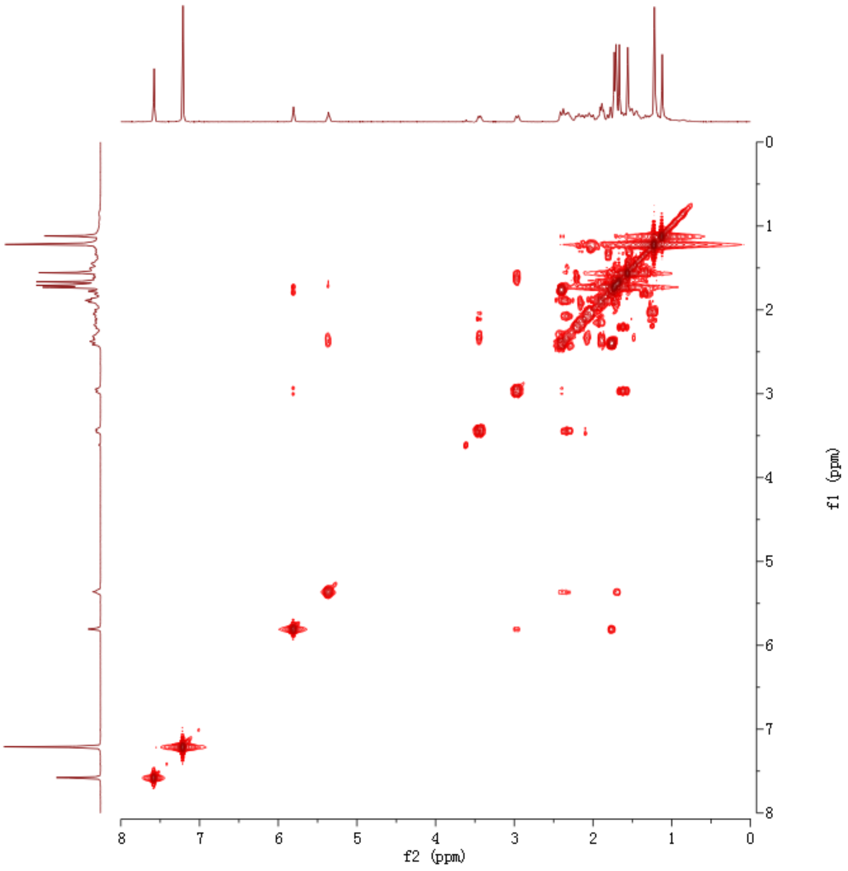
f2 (ppm)
f1 (ppm)

e

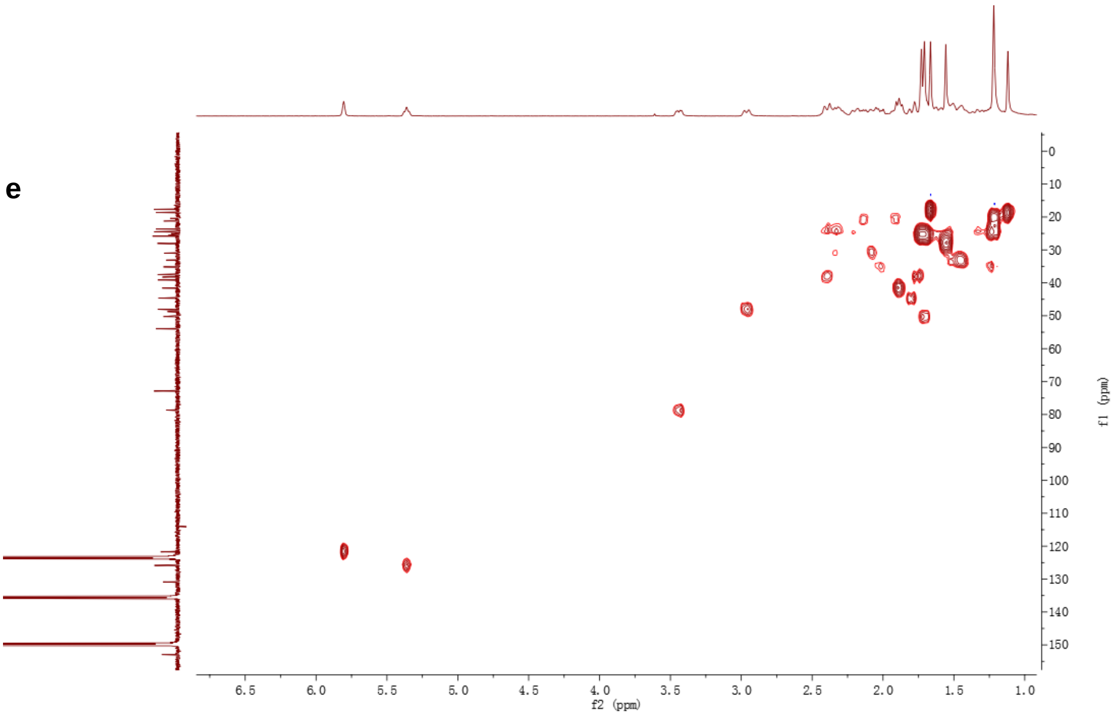

f

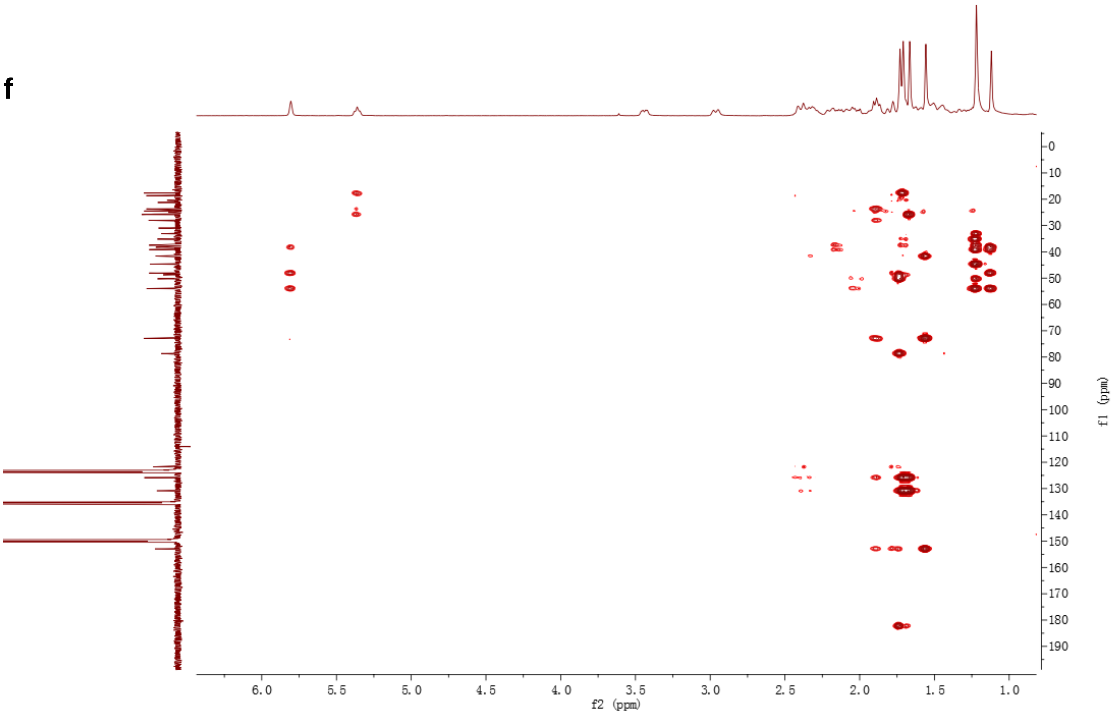

g

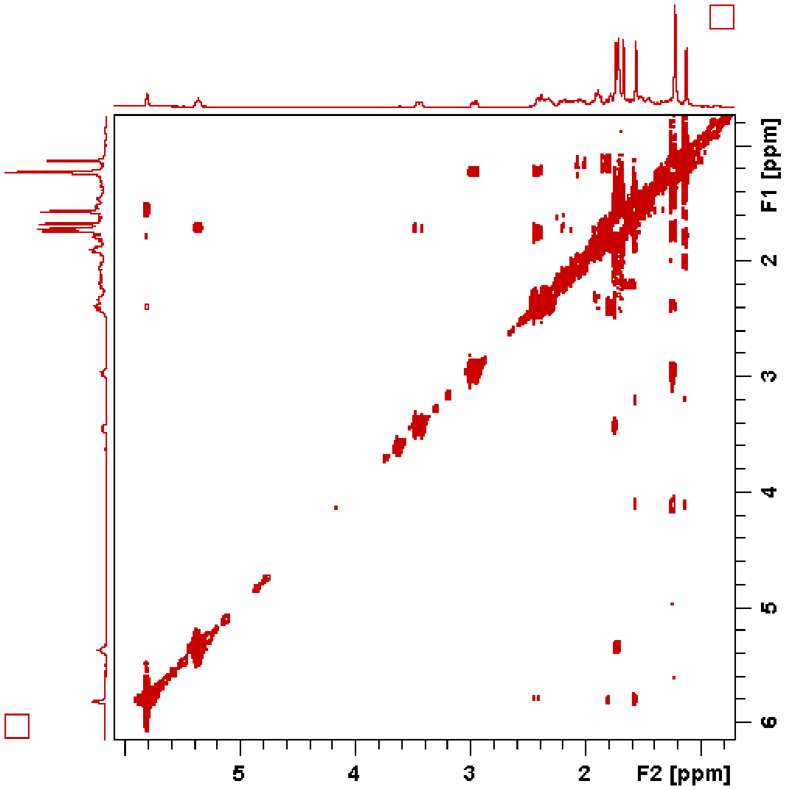

Supplementary Figure 37. HRESIMS and NMR spectra of 10

a HRESIMS spectrum; **b** ^{1}H NMR spectrum in pyridine-d_5 at 400 MHz; **c** ^{13}C NMR spectrum in pyridine-d_5 at 100 MHz; **d** ^{1}H-^{1}H COSY spectrum in pyridine-d_5 at 400 MHz; **e** HSQC spectrum in pyridine-d_5 at 400 MHz; **f** HMBC spectrum in pyridine-d_5 at 400 MHz; **g** NOESY spectrum in pyridine-d_5 at 400 MHz.

a

Single Mass Analysis
Tolerance = 5.0 mDa / DBE: min = -1.5, max = 15.0
Element prediction: Off
Number of isotope peaks used for i-FIT = 3

Monoisotopic Mass, Even Electron Ions
115 formula(e) evaluated with 1 results within limits (up to 50 closest results for each mass)
Elements Used:
C: 0-500 H: 0-1000 O: 0-200
AFU-12
2016082229 225 (1.818) Cm (225:230)

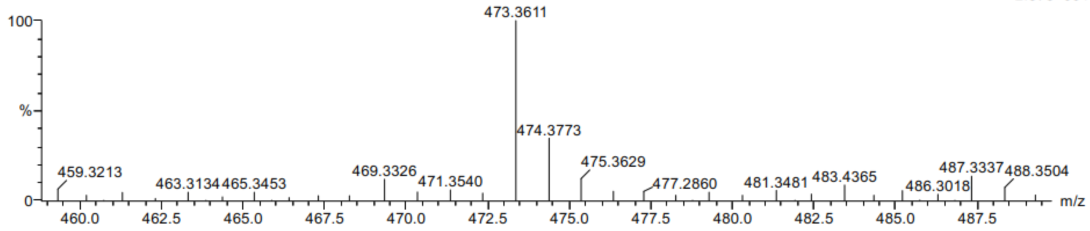

Minimum:				-1.5				
Maximum:		5.0	10.0	15.0				
Mass	Calc. Mass	mDa	PPM	DBE	i-FIT	Norm	Conf(%)	Formula
473.3611	473.3631	-2.0	-4.2	6.5	29.8	n/a	n/a	C30 H49 O4

b

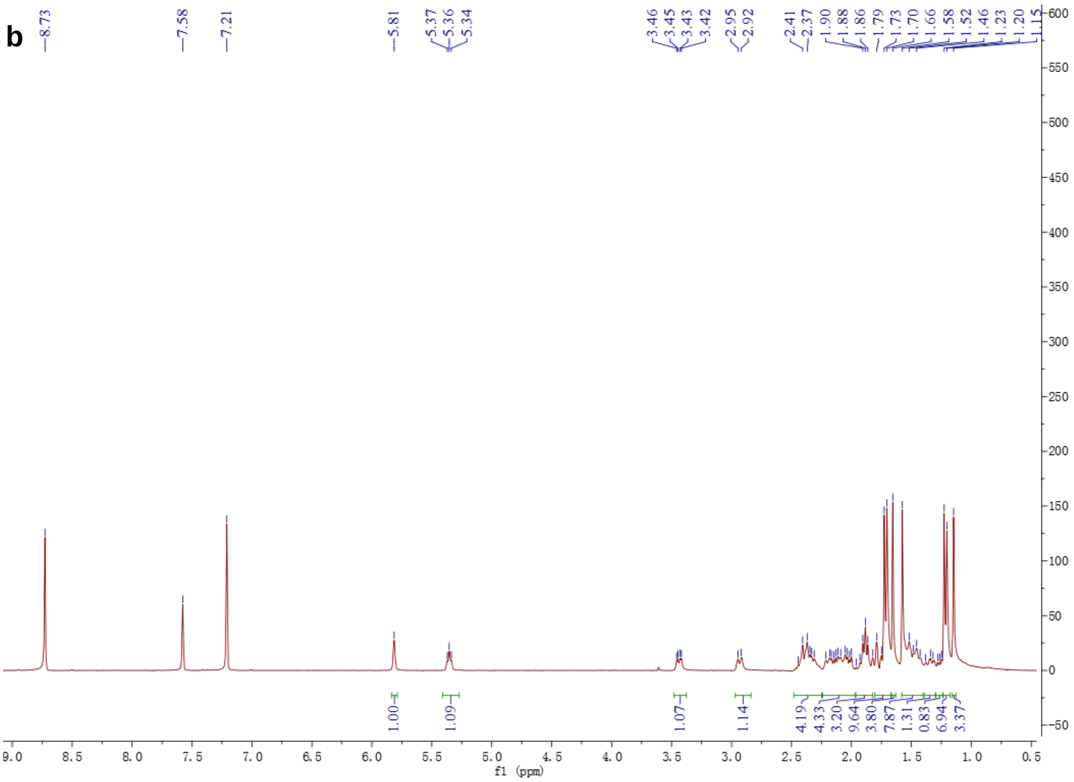

c

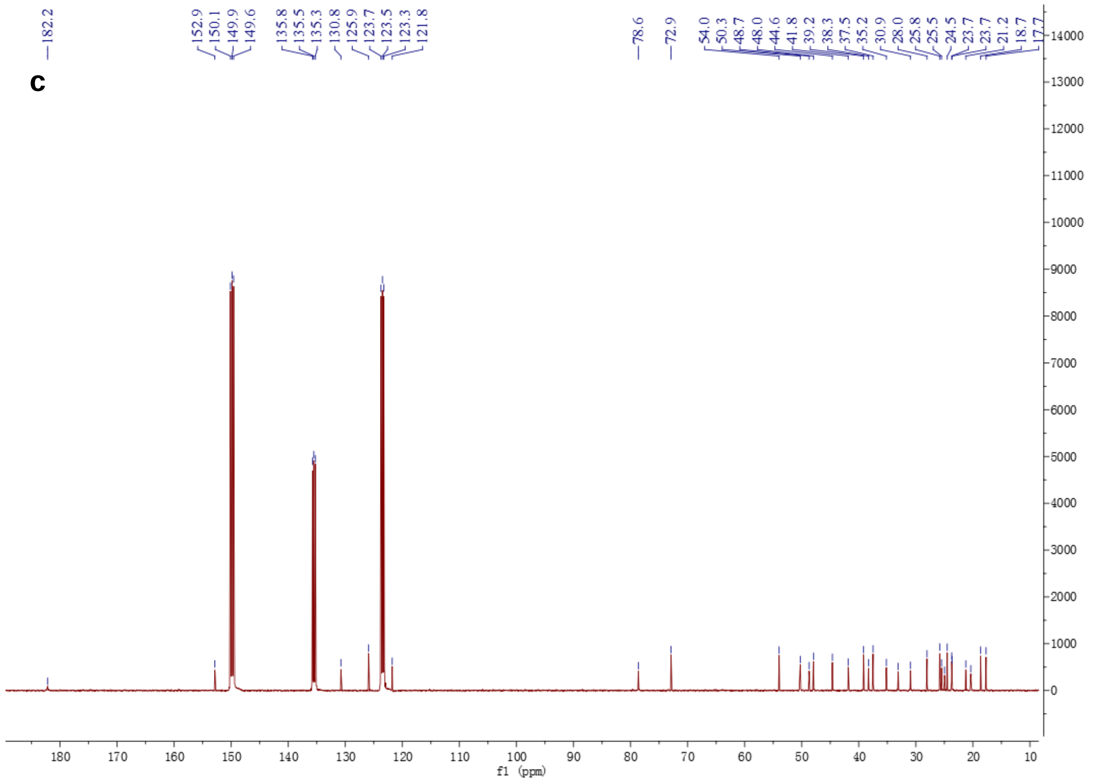
182.2
152.9
150.1
149.9
149.6
135.8
135.5
135.3
130.8
125.9
123.7
123.5
123.3
121.8
78.6
72.9
54.0
50.3
48.7
48.0
44.6
41.8
39.2
38.3
37.5
35.2
30.9
28.0
25.8
25.5
24.5
23.7
23.7
21.2
18.7
17.7
f1 (ppm)

d

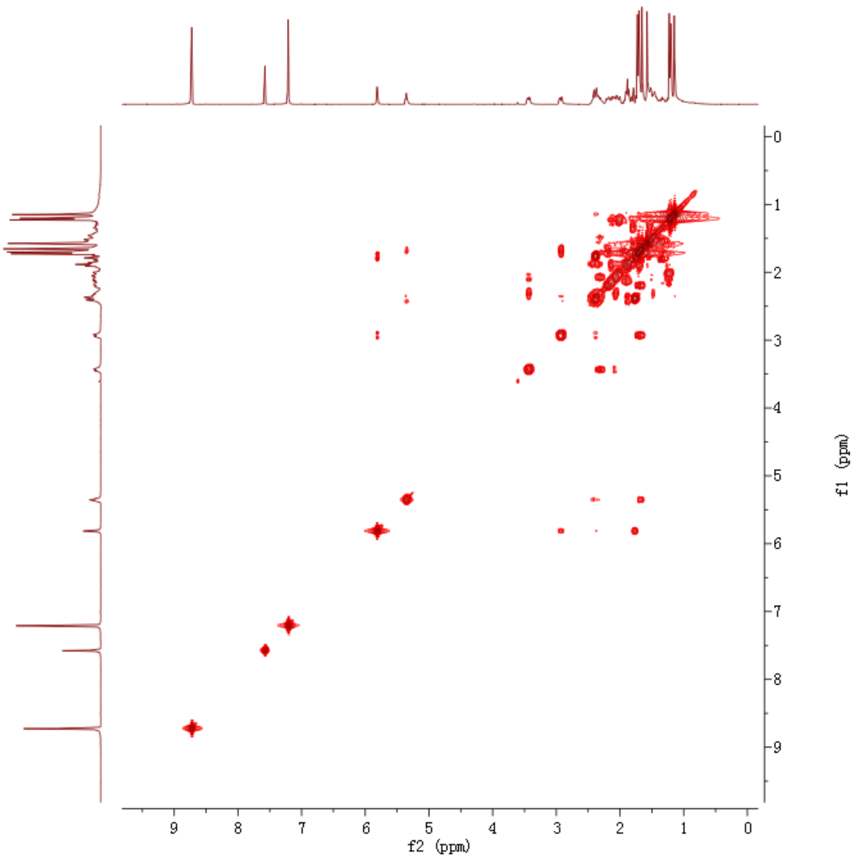
f2 (ppm)
f1 (ppm)

e

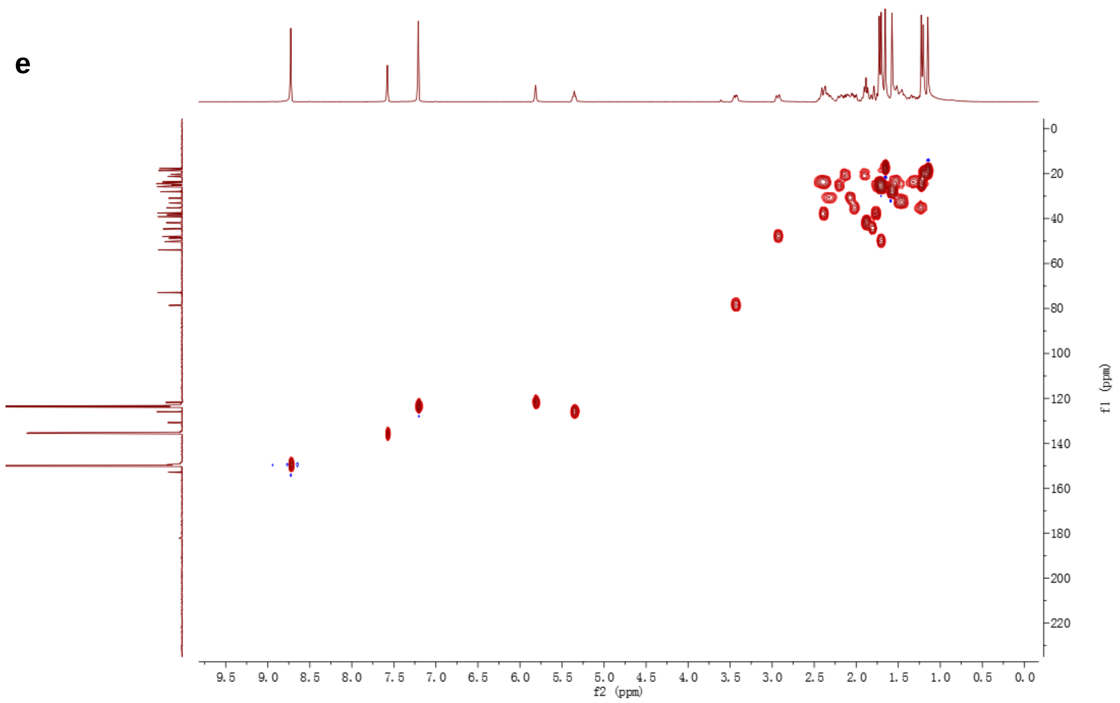
9.5 9.0 8.5 8.0 7.5 7.0 6.5 6.0 5.5 5.0 4.5 4.0 3.5 3.0 2.5 2.0 1.5 1.0 0.5 0.0
f2 (ppm)
0 20 40 60 80 100 120 140 160 180 200 220
f1 (ppm)

f

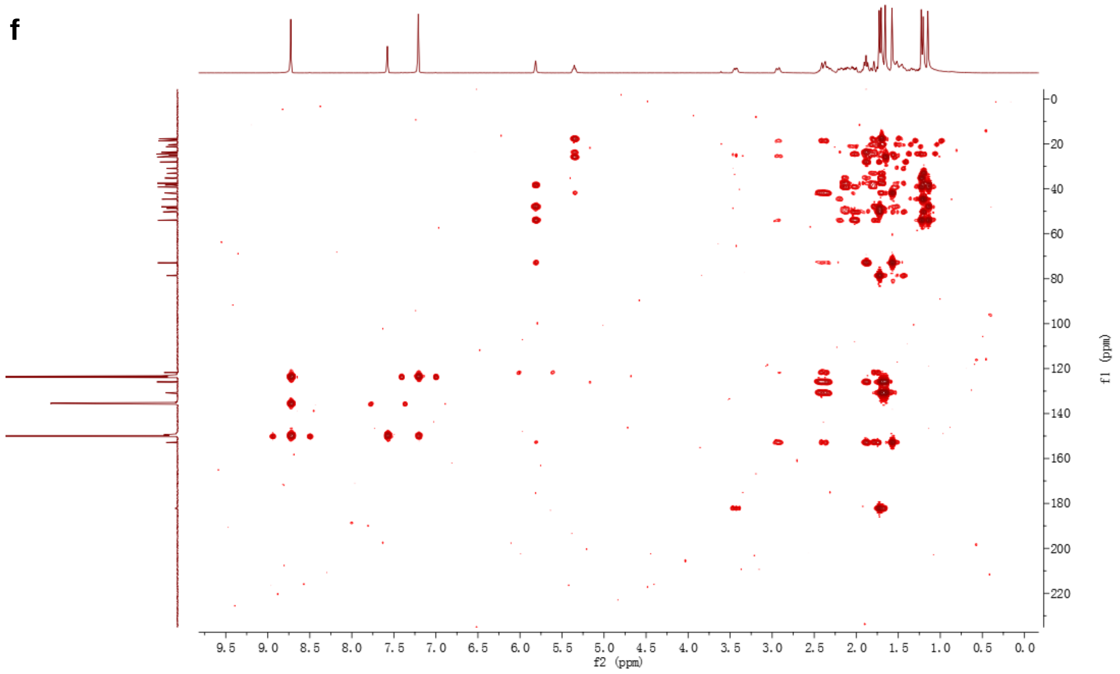
9.5 9.0 8.5 8.0 7.5 7.0 6.5 6.0 5.5 5.0 4.5 4.0 3.5 3.0 2.5 2.0 1.5 1.0 0.5 0.0
f2 (ppm)
0 20 40 60 80 100 120 140 160 180 200 220
f1 (ppm)

g

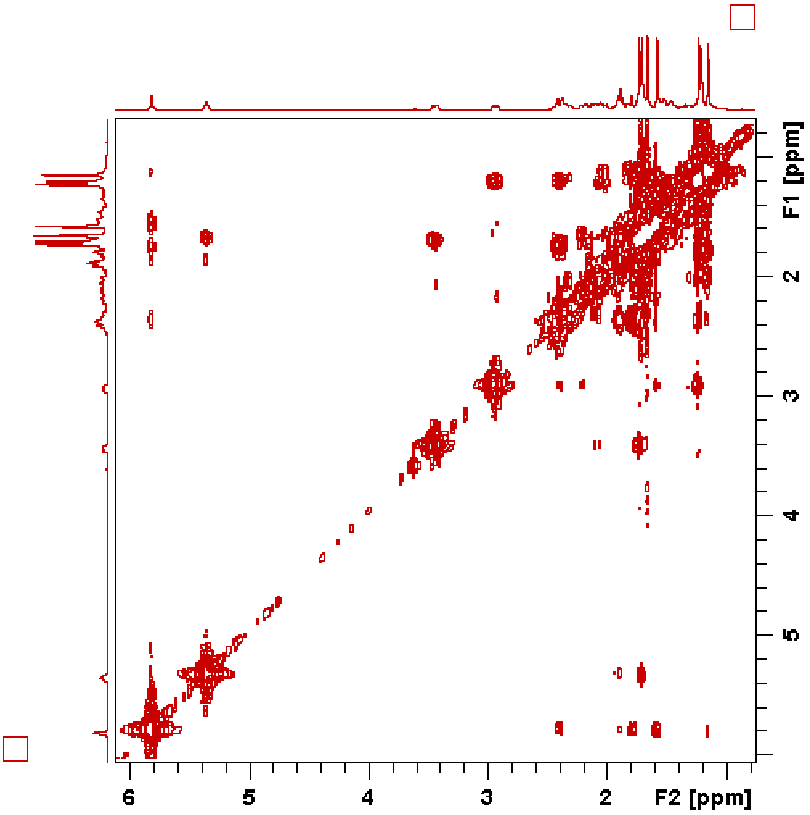

Supplementary Figure 38. HRESIMS and NMR spectra of 11

a HRESIMS spectrum; **b** ^{1}H NMR spectrum in pyridine-d_5 at 400 MHz; **c** ^{13}C NMR spectrum in pyridine-d_5 at 100 MHz; **d** ^{1}H-^{1}H COSY spectrum in pyridine-d_5 at 400 MHz; **e** HSQC spectrum in pyridine-d_5 at 400 MHz; **f** HMBC spectrum in pyridine-d_5 at 400 MHz; **g** NOESY spectrum in pyridine-d_5 at 400 MHz.

a

Single Mass Analysis

Tolerance = 5.0 PPM / DBE: min = -1.5, max = 50.0
Element prediction: Off
Number of isotope peaks used for i-FIT = 3

Monoisotopic Mass, Even Electron Ions
90 formula(e) evaluated with 1 results within limits (up to 50 closest results for each mass)
Elements Used:
C: 0-80 H: 0-100 O: 0-20
AFU-51
2016110722 282 (2.278) 1: TOF MS ES+
3.85e+003

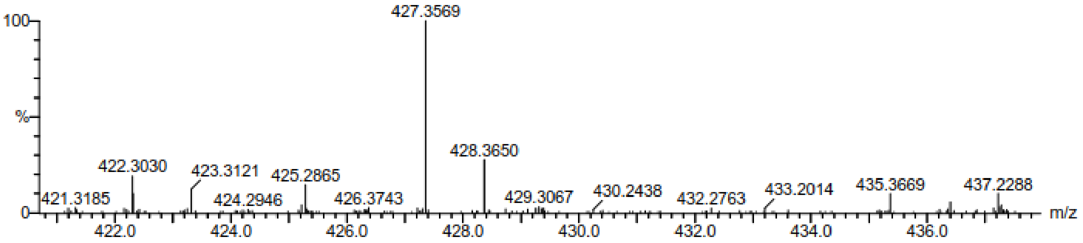

Minimum:				-1.5				
Maximum:		5.0	5.0	50.0				
Mass	Calc. Mass	mDa	PPM	DBE	i-FIT	Norm	Conf(%)	Formula
427.3569	427.3576	-0.7	-1.6	6.5	156.8	n/a	n/a	C29 H47 O2

b

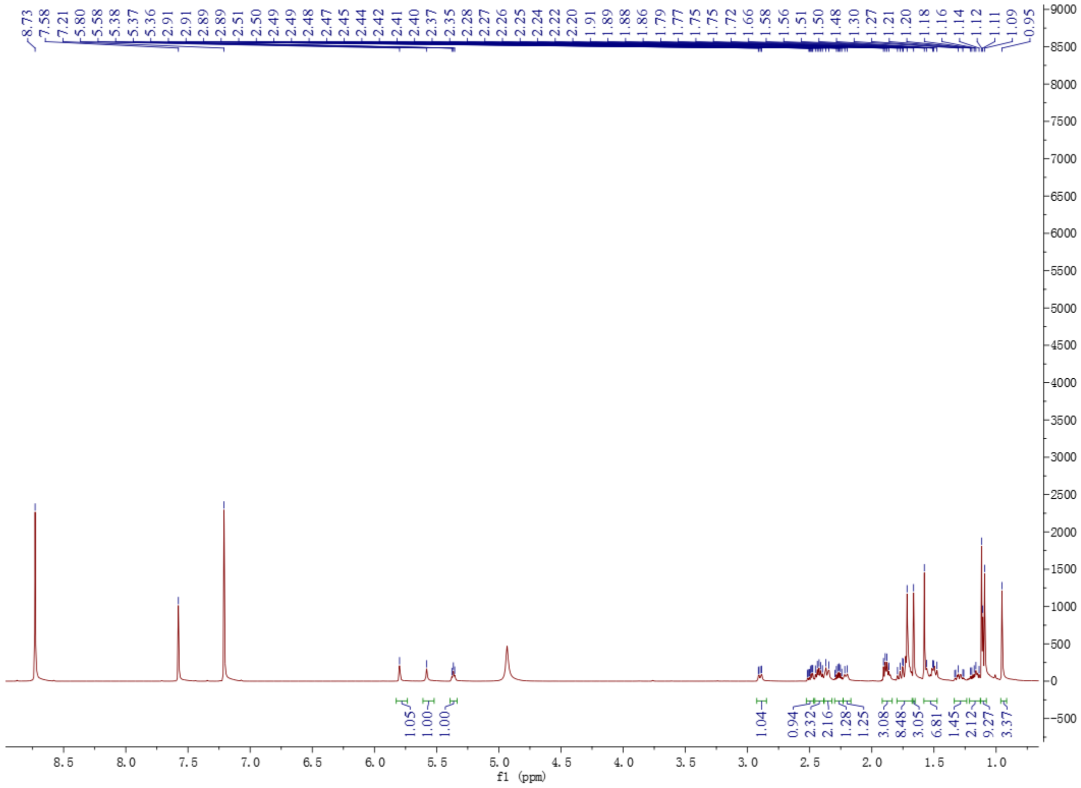

c

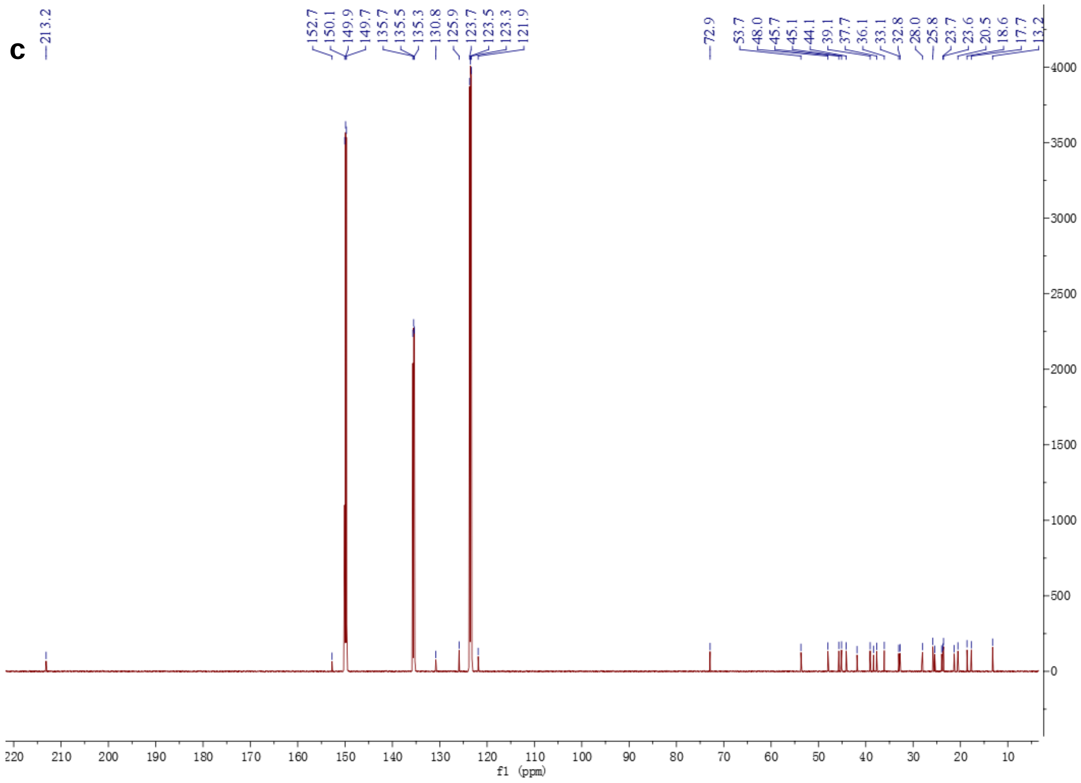
213.2
152.7
150.1
149.9
149.7
135.7
135.5
135.3
130.8
125.9
123.7
123.5
123.3
121.9
72.9
53.7
48.0
45.7
45.1
44.1
39.1
37.7
36.1
33.1
32.8
28.0
25.8
23.7
23.6
20.5
18.6
17.7
13.2
f1 (ppm)

d

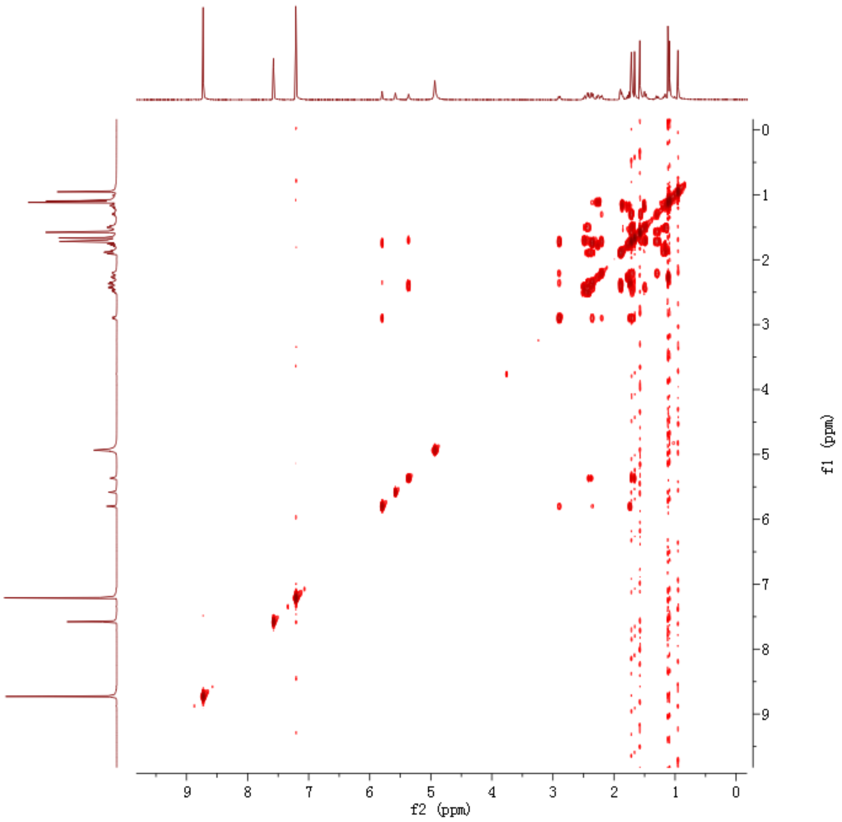
f2 (ppm)
f1 (ppm)

e

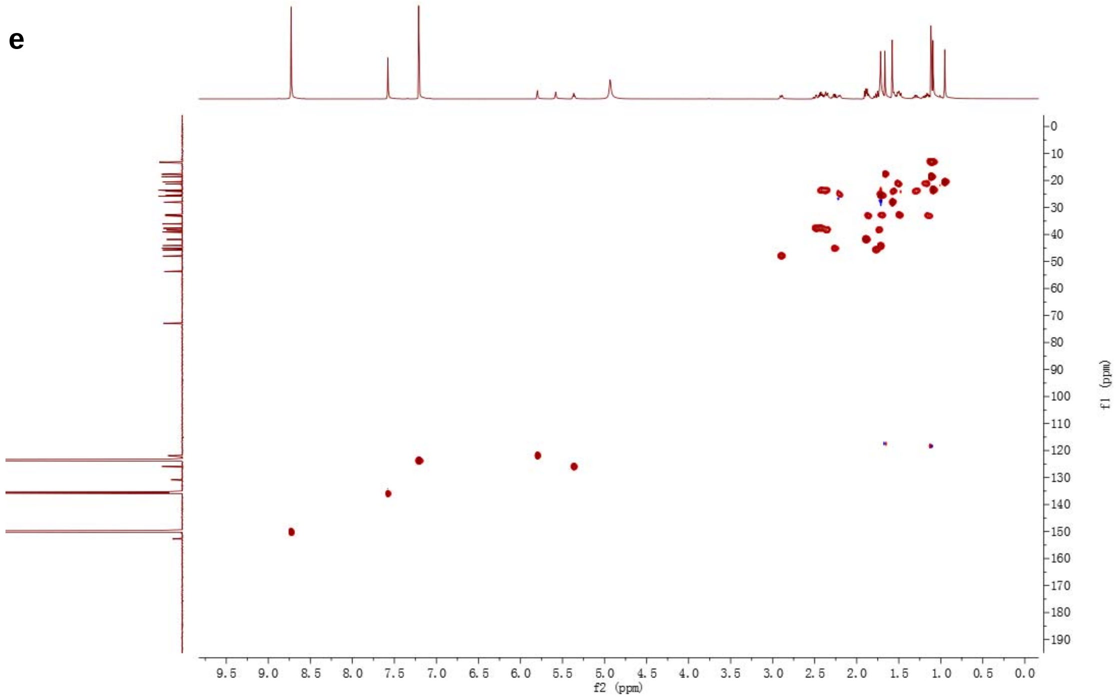

f

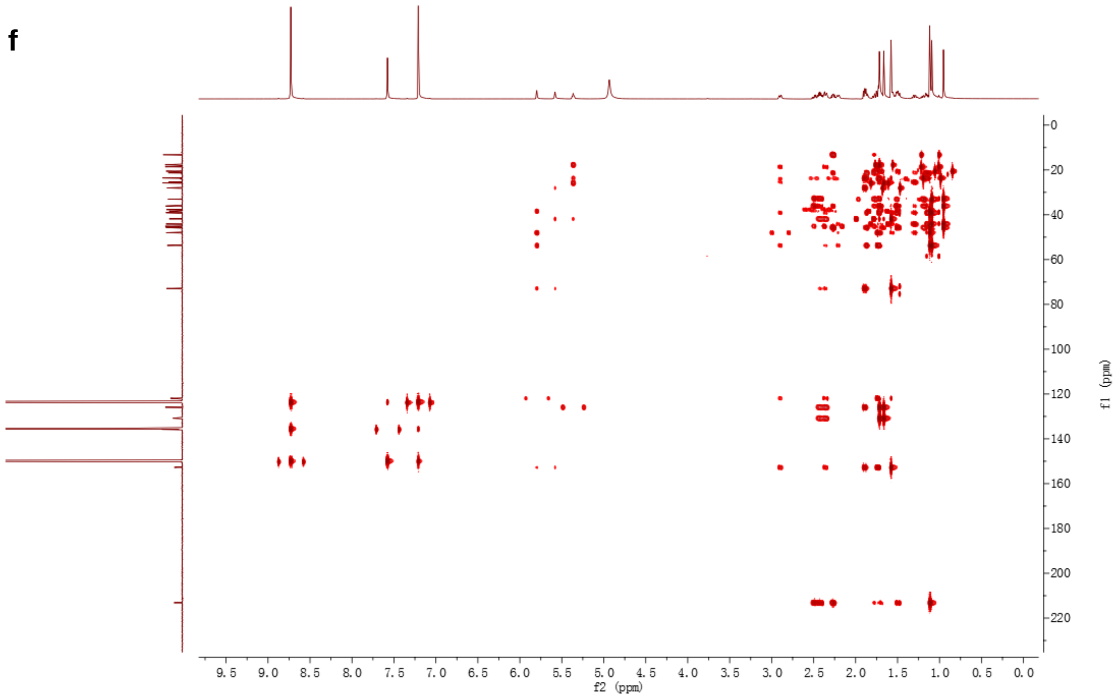

g

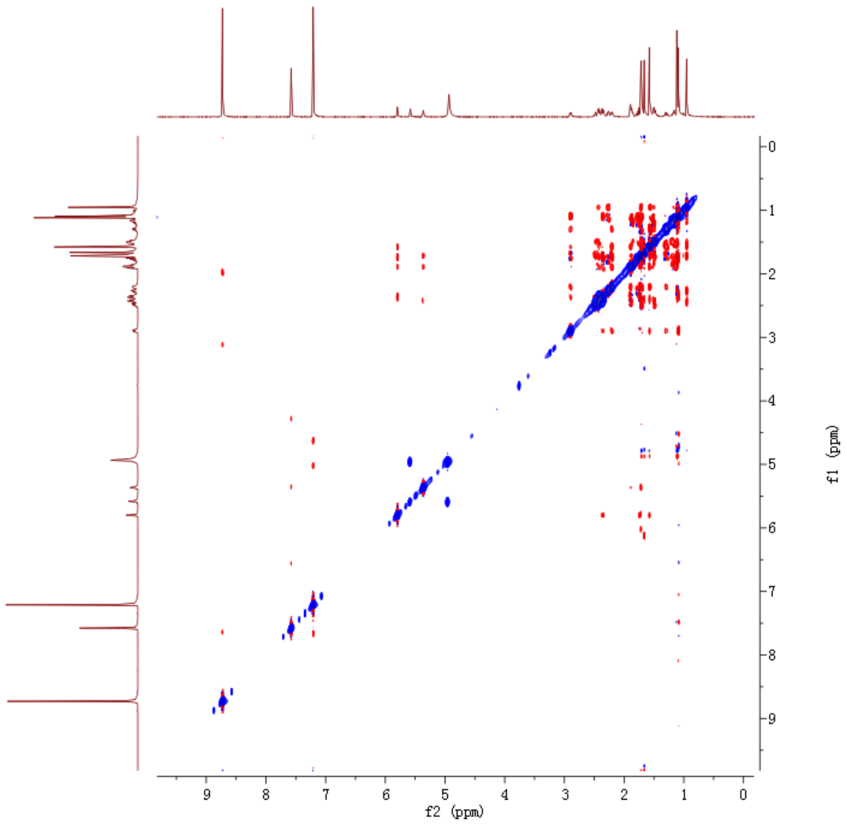

Supplementary Figure 39. HRESIMS and NMR spectra of 12

a HRESIMS spectrum; **b** ^{1}H NMR spectrum in pyridine-d_5 at 600 MHz; **c** ^{13}C NMR spectrum in pyridine-d_5 at 150 MHz; **d** ^{1}H-^{1}H COSY spectrum in pyridine-d_5 at 600 MHz; **e** HSQC spectrum in pyridine-d_5 at 600 MHz; **f** HMBC spectrum in pyridine-d_5 at 600 MHz; **g** ROESY spectrum in pyridine-d_5 at 600 MHz.

a

Single Mass Analysis
Tolerance = 5.0 PPM / DBE: min = -1.5, max = 50.0
Element prediction: Off
Number of isotope peaks used for i-FIT = 3

Monoisotopic Mass, Even Electron Ions
90 formula(e) evaluated with 1 results within limits (up to 50 closest results for each mass)
Elements Used:
C: 0-80 H: 0-100 O: 0-20
AFU-52
2016110723 282 (2.278)

1: TOF MS ES+
4.24e+003

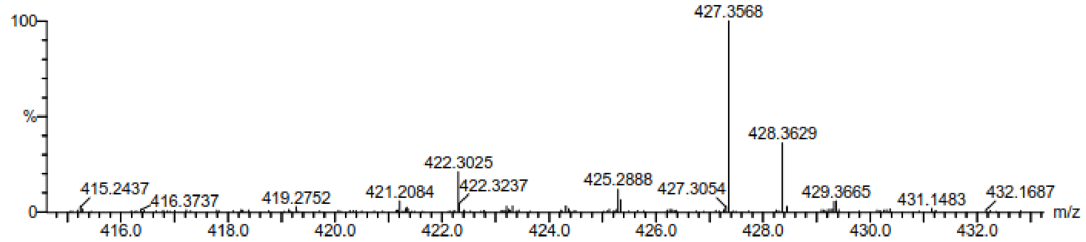

Minimum:				-1.5				
Maximum:		5.0	5.0	50.0				
Mass	Calc. Mass	mDa	PPM	DBE	i-FIT	Norm	Conf(%)	Formula
427.3568	427.3576	-0.8	-1.9	6.5	126.3	n/a	n/a	C29 H47 O2

b

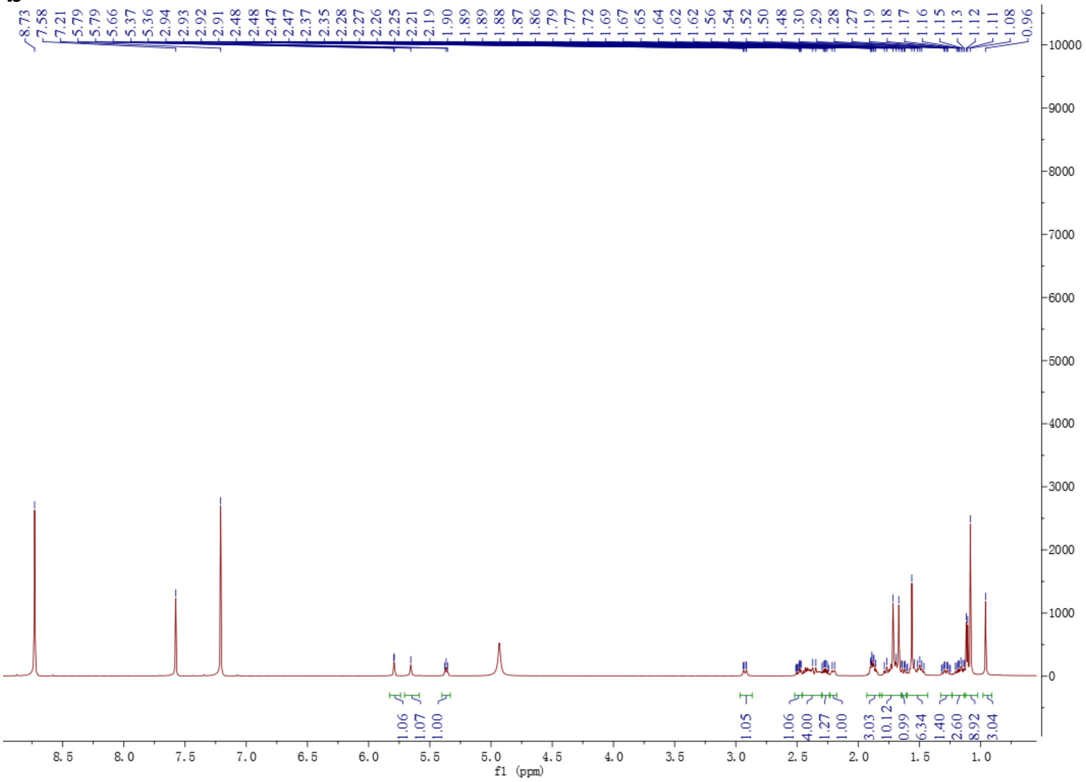

c

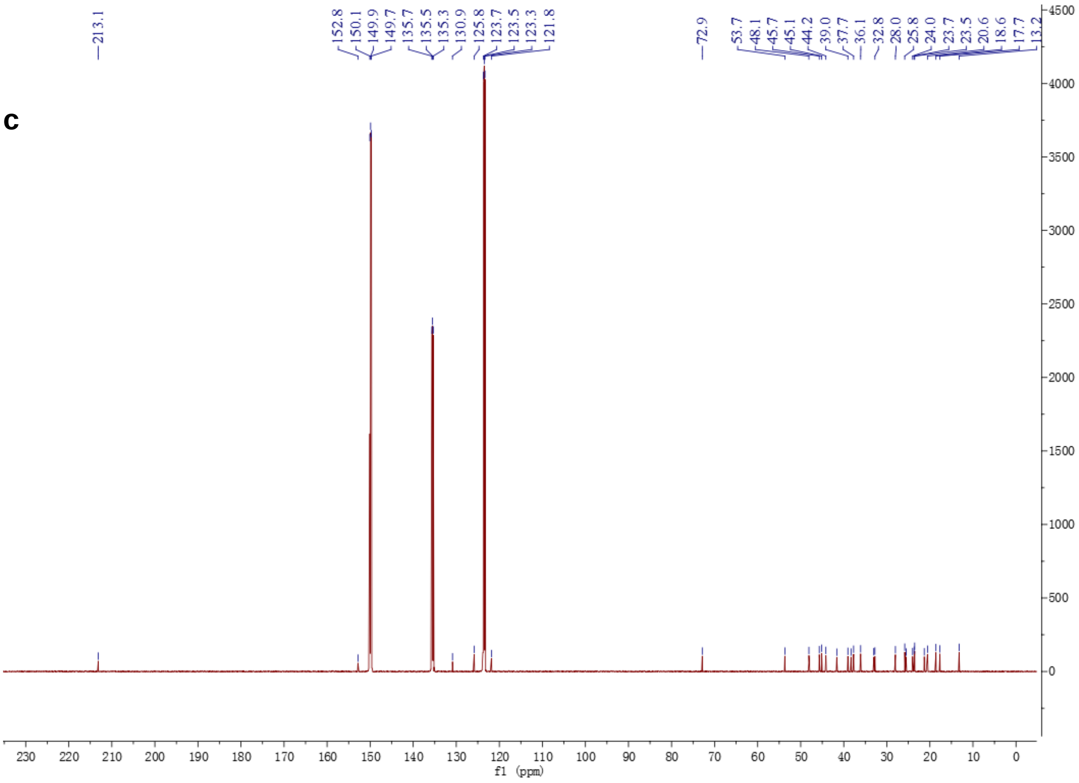
213.1
152.8
150.1
149.9
149.7
135.7
135.5
135.3
130.9
125.8
123.7
123.5
123.3
121.8
72.9
53.7
48.1
45.7
45.1
44.2
39.0
37.7
36.1
32.8
28.0
25.8
24.0
23.7
23.5
20.6
18.6
17.7
13.2
f1 (ppm)

d

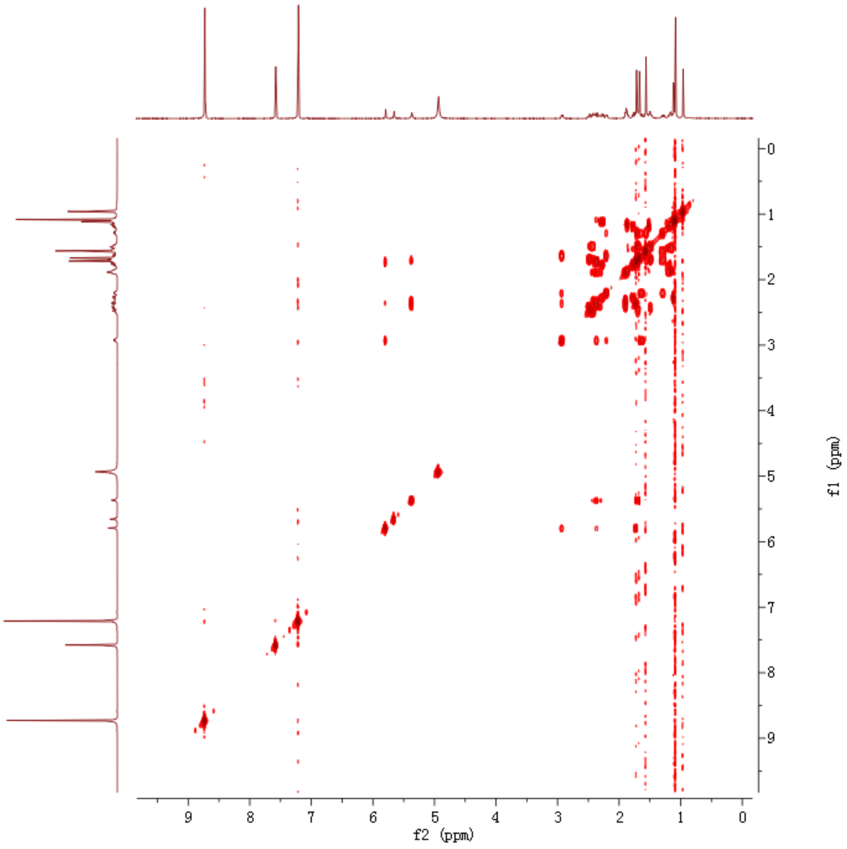
f2 (ppm)
f1 (ppm)

e

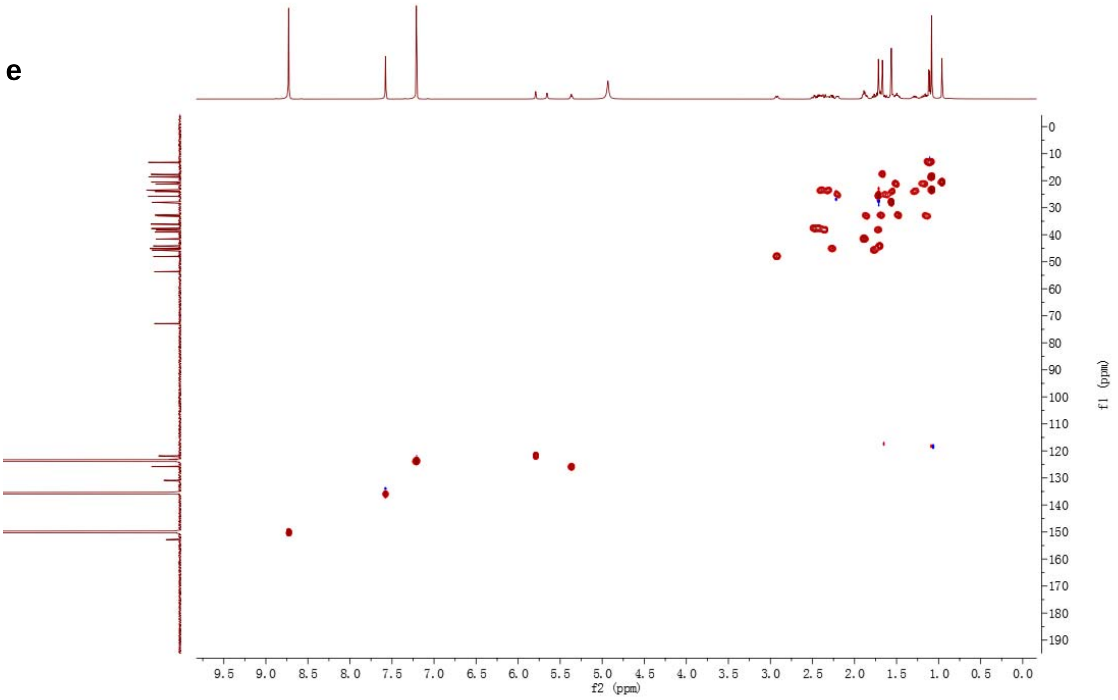

f

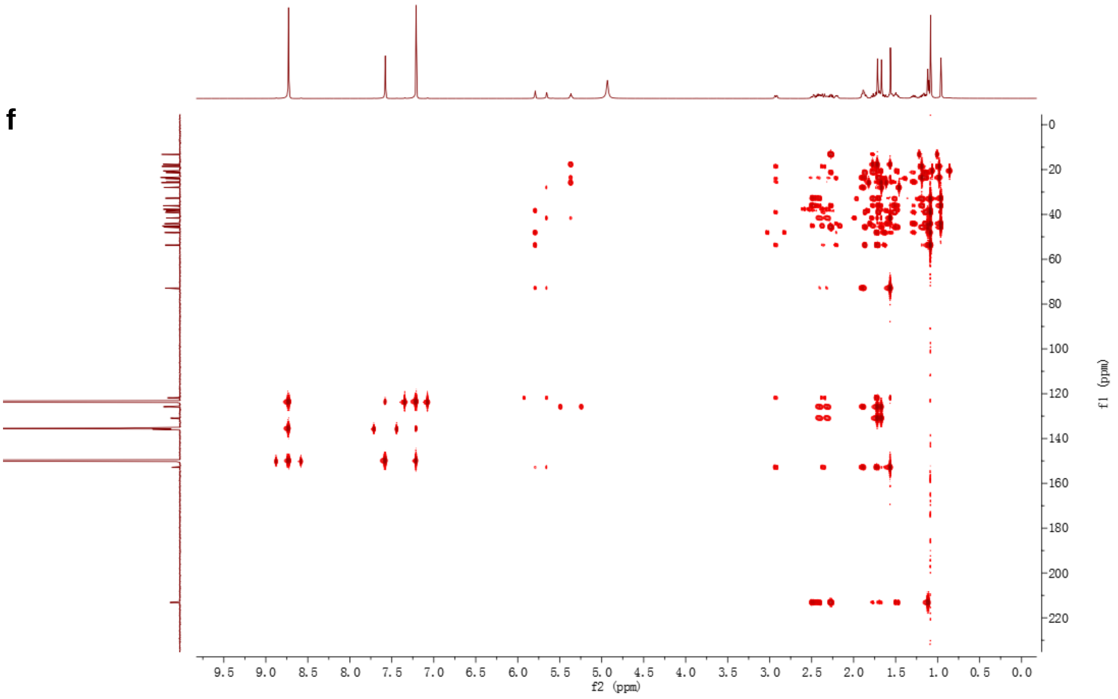

g

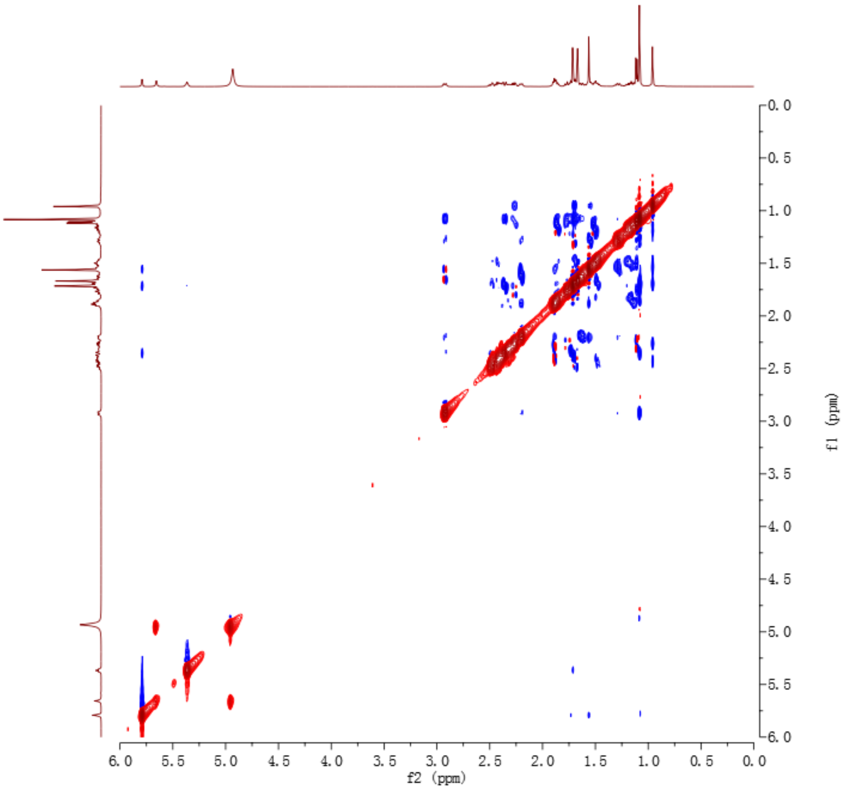

Supplementary Figure 40. HRESIMS and NMR spectra of 13

a HRESIMS spectrum; **b** ^{1}H NMR spectrum in pyridine-d_5 at 600 MHz; **c** ^{13}C NMR spectrum in pyridine-d_5 at 150 MHz; **d** ^{1}H-^{1}H COSY spectrum in pyridine-d_5 at 600 MHz; **e** HSQC spectrum in pyridine-d_5 at 600 MHz; **f** HMBC spectrum in pyridine-d_5 at 600 MHz; **g** ROESY spectrum in pyridine-d_5 at 600 MHz.

a

Single Mass Analysis
Tolerance = 5.0 mDa / DBE: min = -1.5, max = 15.0
Element prediction: Off
Number of isotope peaks used for i-FIT = 3

Monoisotopic Mass, Even Electron Ions
256 formula(e) evaluated with 2 results within limits (up to 50 closest results for each mass)
Elements Used:
C: 0-500 H: 0-1000 O: 0-200 Na: 0-1

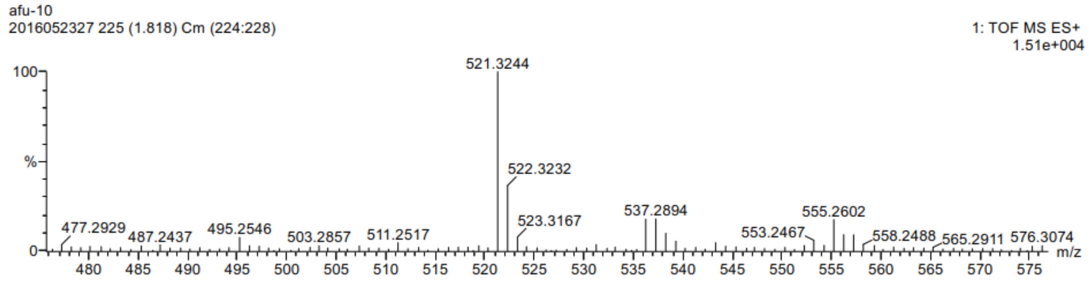

Minimum:				-1.5				
Maximum:		5.0	10.0	15.0				

Mass	Calc. Mass	mDa	PPM	DBE	i-FIT	Norm	Conf(%)	Formula
521.3244	521.3243	0.1	0.2	8.5	20.8	4.341	1.30	C31 H46 O5 Na
	521.3267	-2.3	-4.4	11.5	16.5	0.013	98.70	C33 H45 O5

b

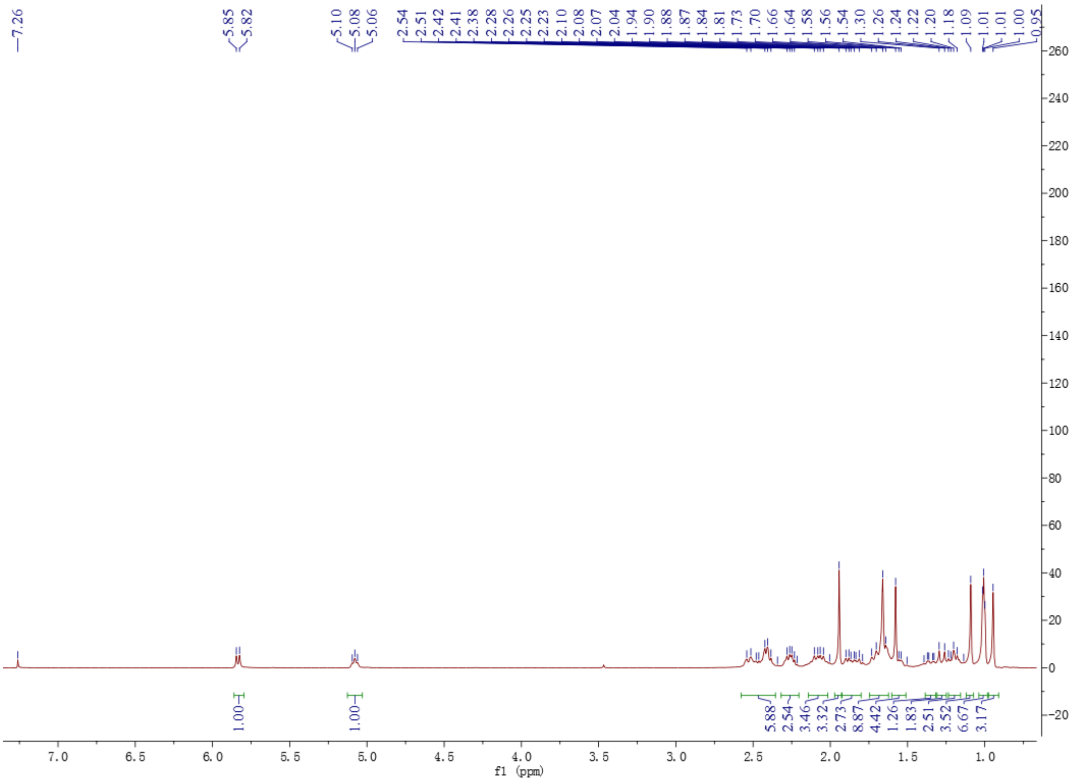

c

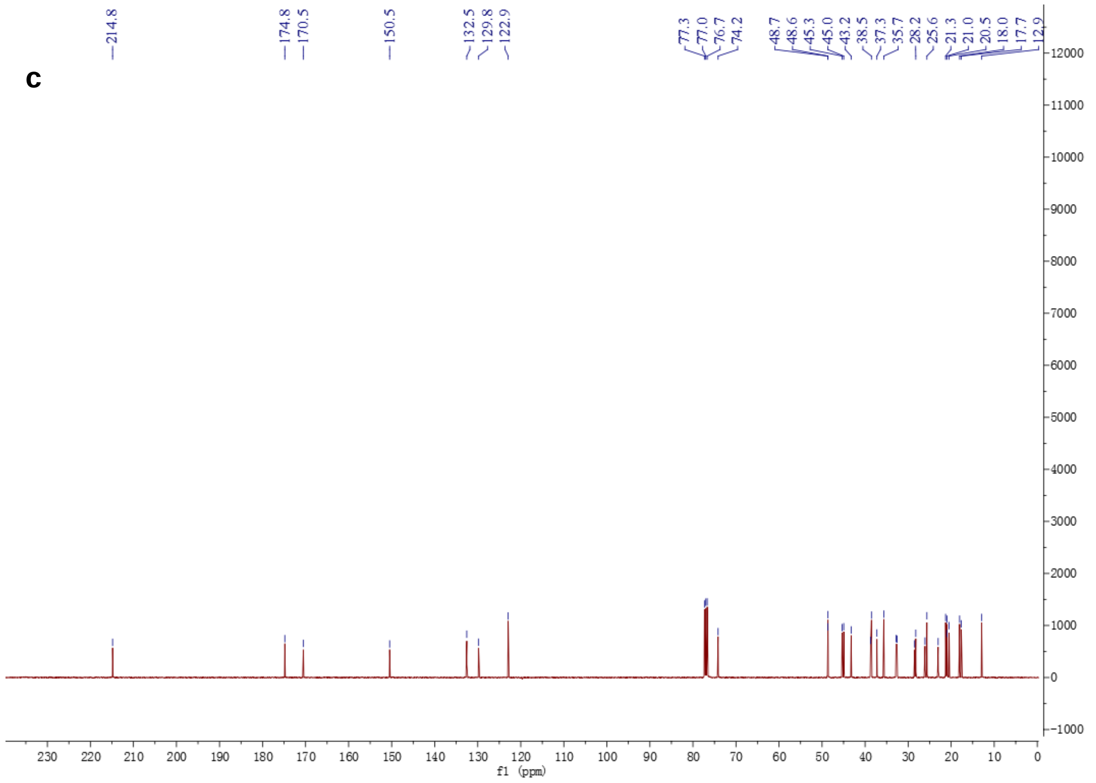

d

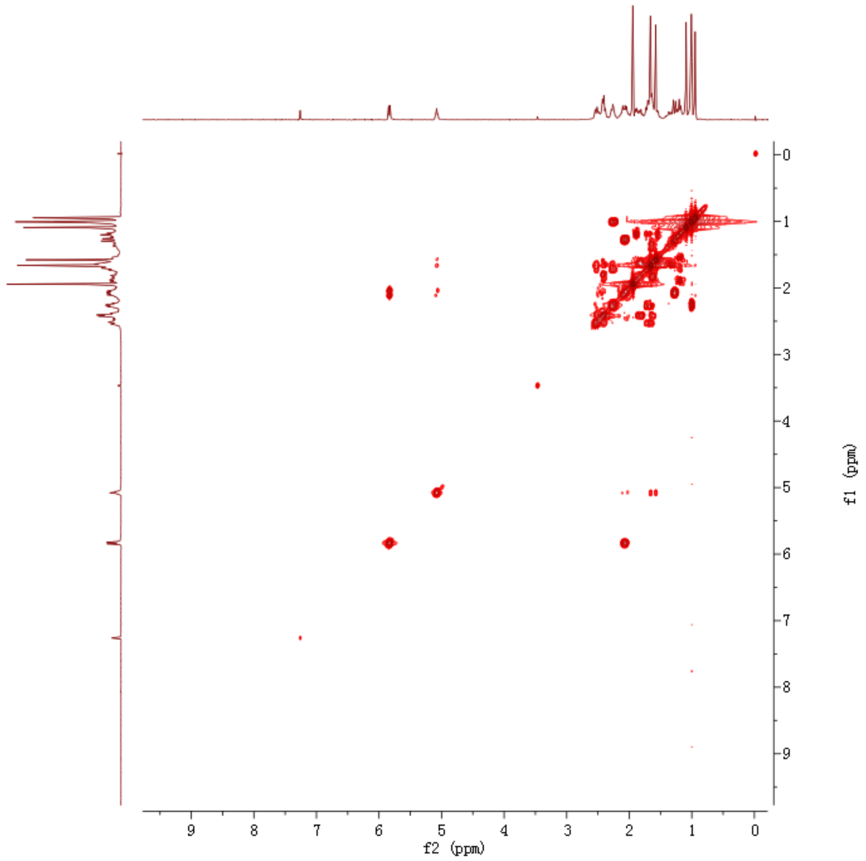

e

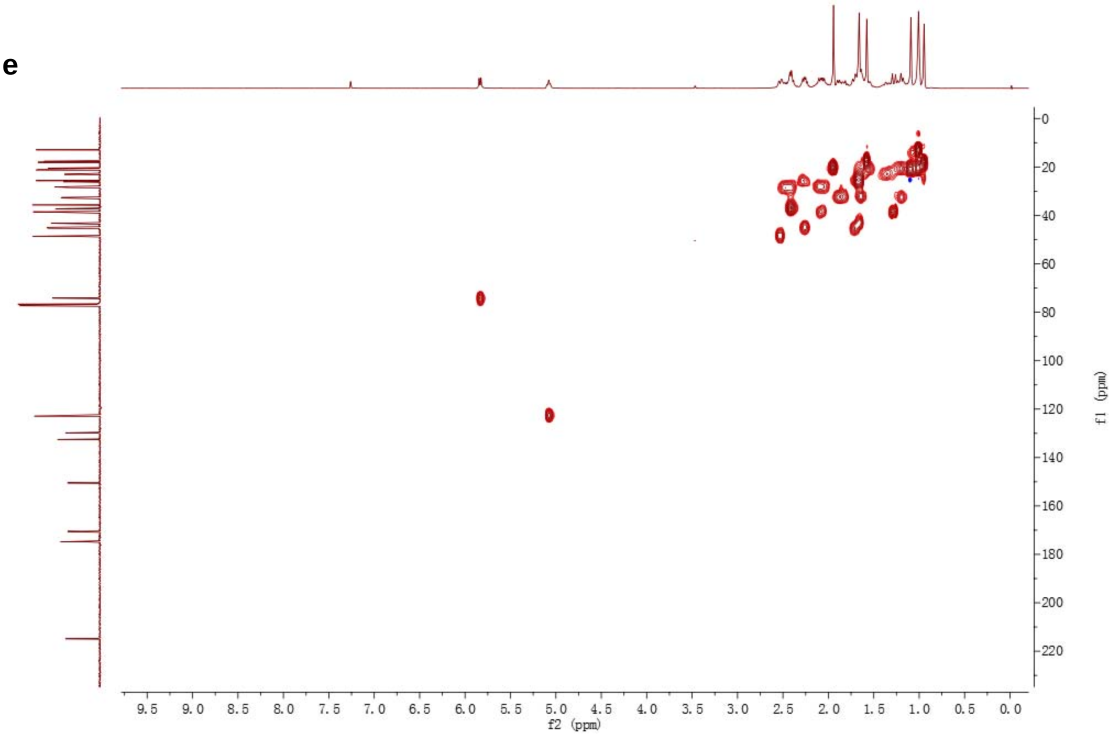

9.5
9.0
8.5
8.0
7.5
7.0
6.5
6.0
5.5
5.0
4.5
4.0
3.5
3.0
2.5
2.0
1.5
1.0
0.5
0.0
f2 (ppm)
f1 (ppm)

f

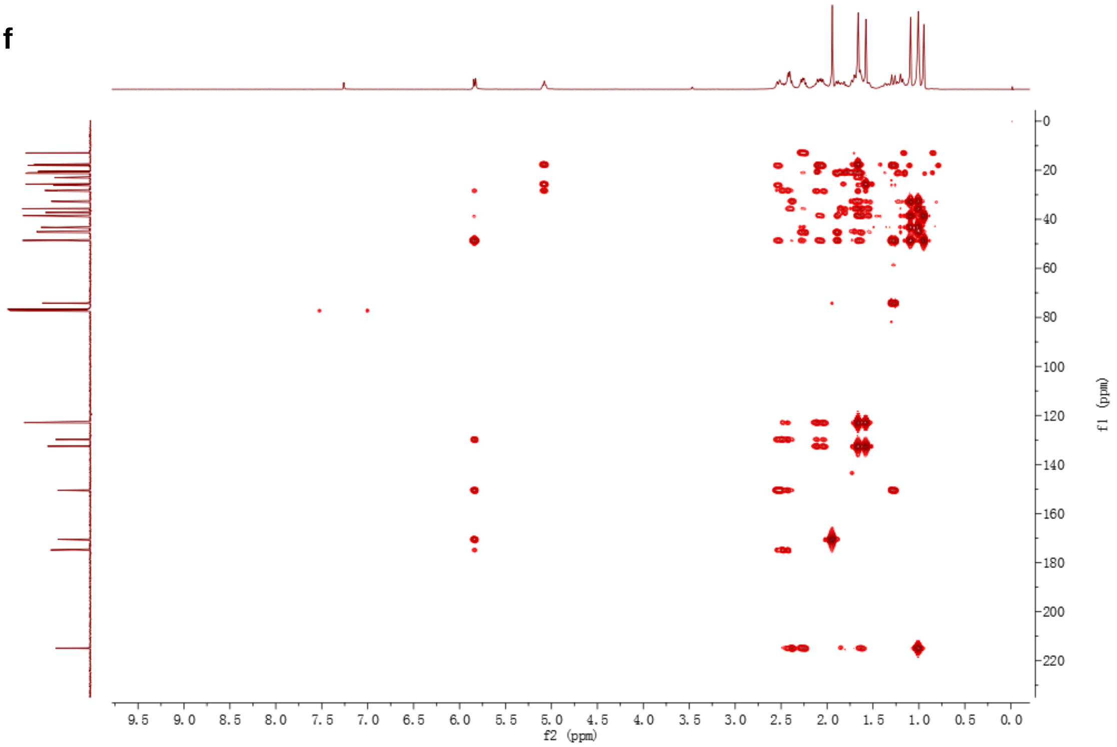

9.5
9.0
8.5
8.0
7.5
7.0
6.5
6.0
5.5
5.0
4.5
4.0
3.5
3.0
2.5
2.0
1.5
1.0
0.5
0.0
f2 (ppm)
f1 (ppm)

g

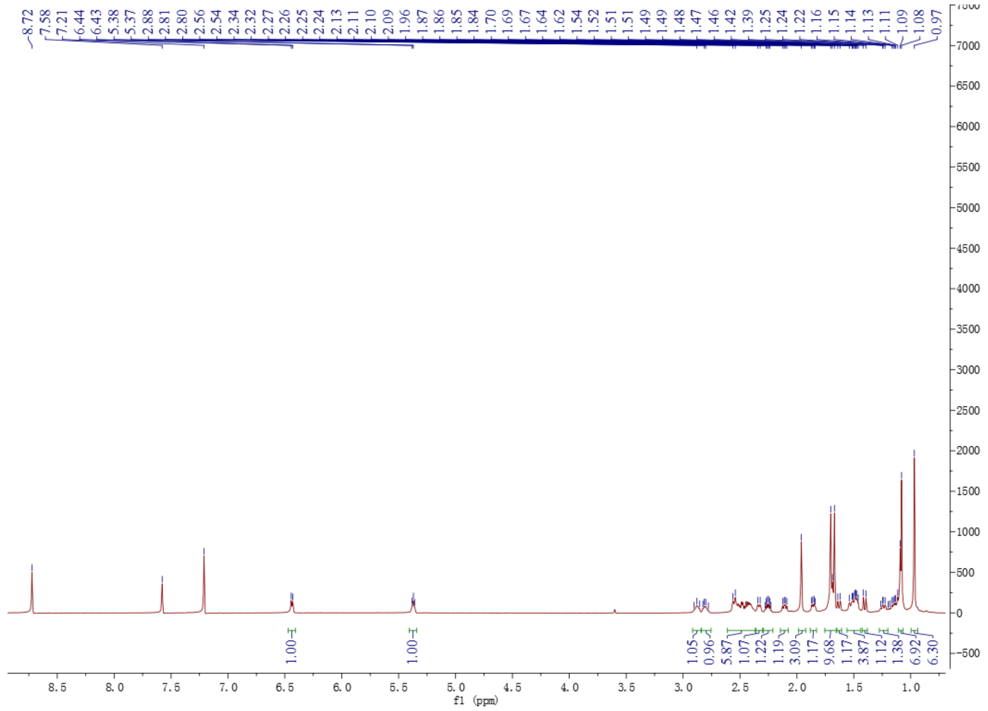

h

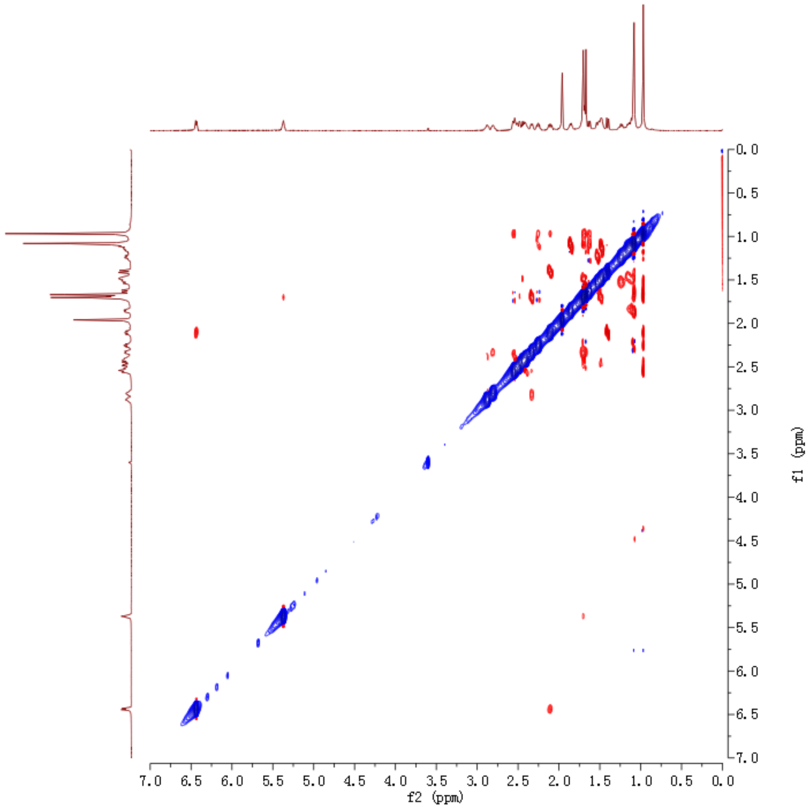

Supplementary Figure 41. HRESIMS and NMR spectra of 14

a HRESIMS spectrum; **b** ^{1}H NMR spectrum in $CDCl_3$ at 400 MHz; **c** ^{13}C NMR spectrum in $CDCl_3$ at 100 MHz; **d** ^{1}H-^{1}H COSY spectrum in $CDCl_3$ at 400 MHz; **e** HSQC spectrum in $CDCl_3$ at 400 MHz; **f** HMBC spectrum in $CDCl_3$ at 400 MHz; **g** ^{1}H NMR spectrum in pyridine-d_5 at 600 MHz; **h** ROESY spectrum in pyridine-d_5 at 600 MHz.

a

Single Mass Analysis
Tolerance = 5.0 mDa / DBE: min = -1.5, max = 15.0
Element prediction: Off
Number of isotope peaks used for i-FIT = 3

Monoisotopic Mass, Even Electron Ions
268 formula(e) evaluated with 2 results within limits (up to 50 closest results for each mass)
Elements Used:
C: 0-500 H: 0-1000 O: 0-200 Na: 0-1
afu-9
2016053005 206 (1.664) Cm (205:208)

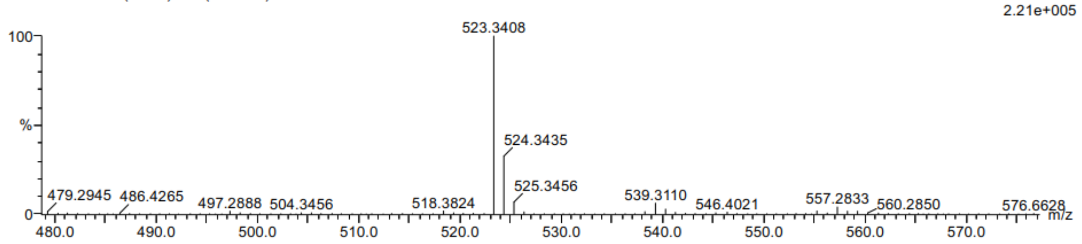

Minimum:				-1.5				
Maximum:		5.0	10.0	15.0				
Mass	Calc. Mass	mDa	PPM	DBE	i-FIT	Norm	Conf(%)	Formula
523.3408	523.3399	0.9	1.7	7.5	19.1	0.020	97.99	C31 H48 O5 Na
	523.3423	-1.5	-2.9	10.5	23.0	3.909	2.01	C33 H47 O5

b

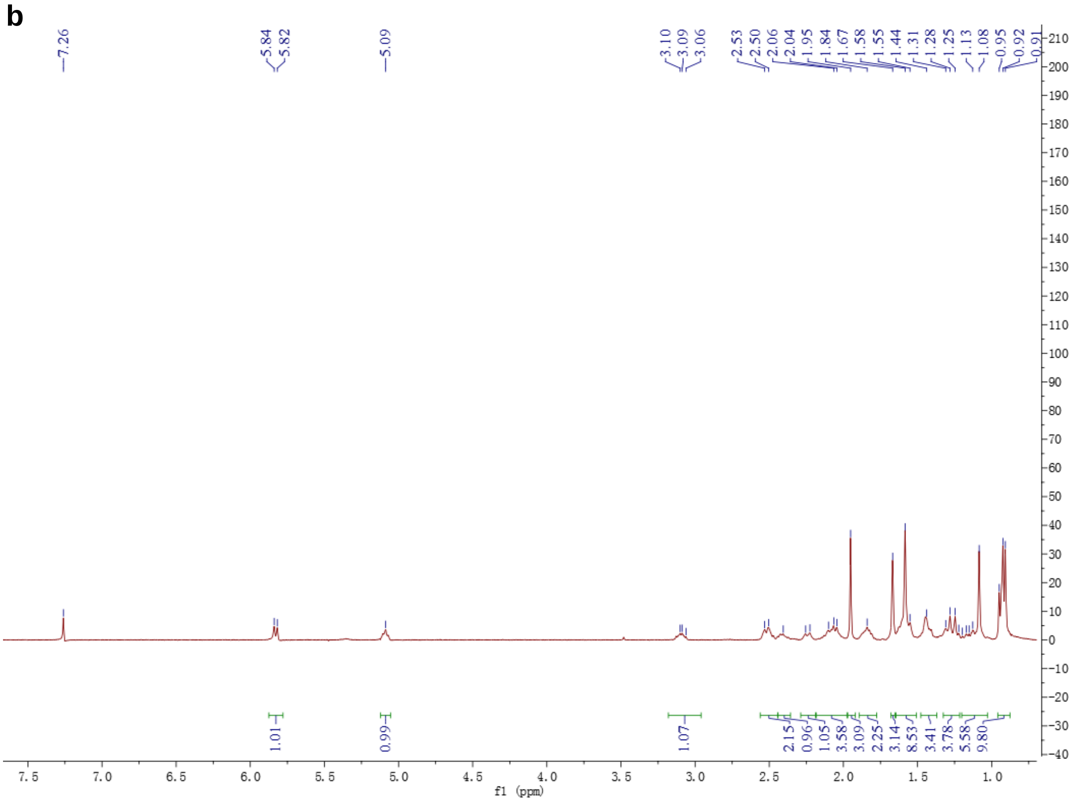

c

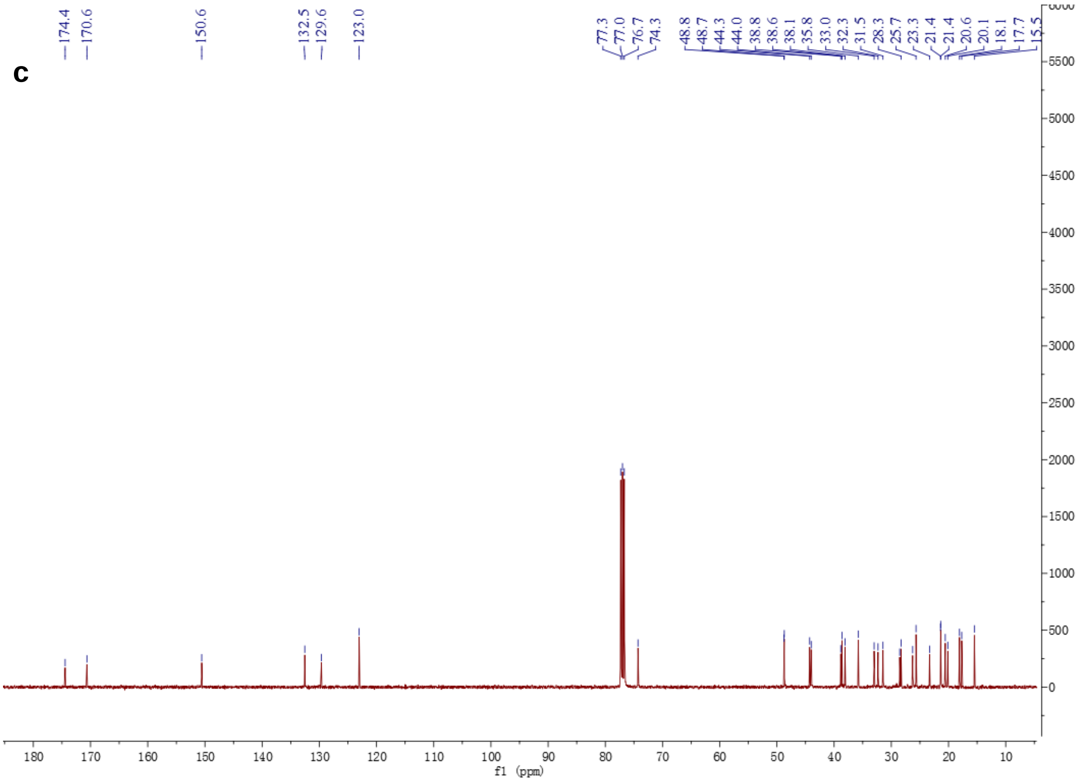
174.4
170.6
150.6
132.5
129.6
123.0
77.3
77.0
76.7
74.3
48.8
48.7
44.3
44.0
38.8
38.6
38.1
35.8
33.0
32.3
31.5
28.3
25.7
23.3
21.4
21.4
20.6
20.1
18.1
17.7
15.5
f1 (ppm)

d

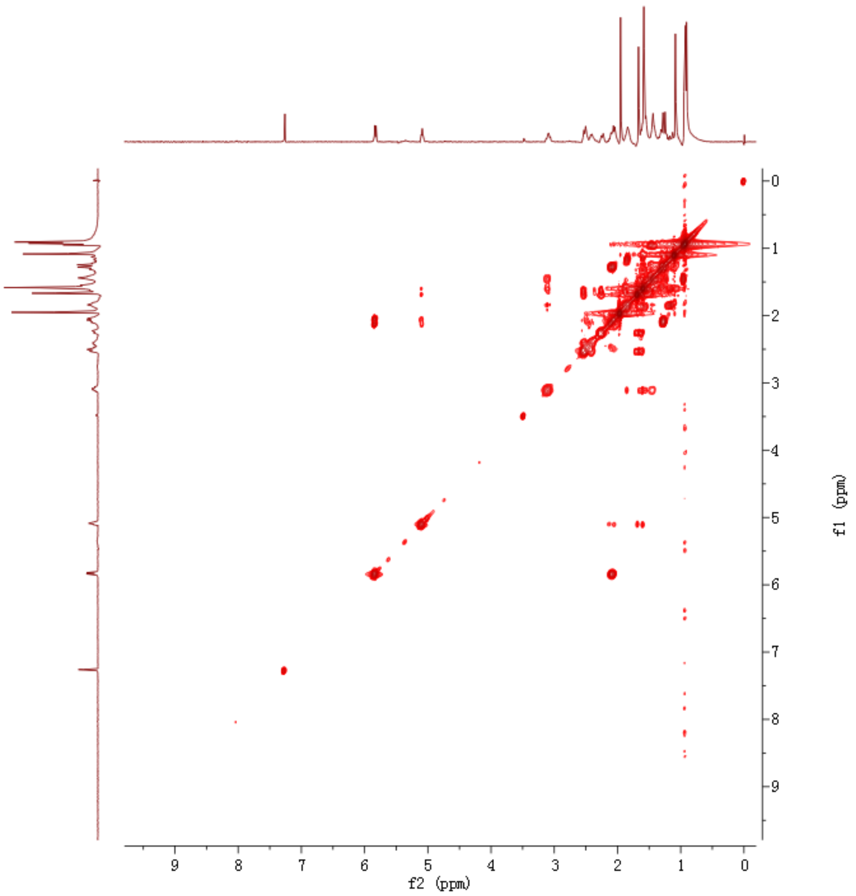
f2 (ppm)
f1 (ppm)

e

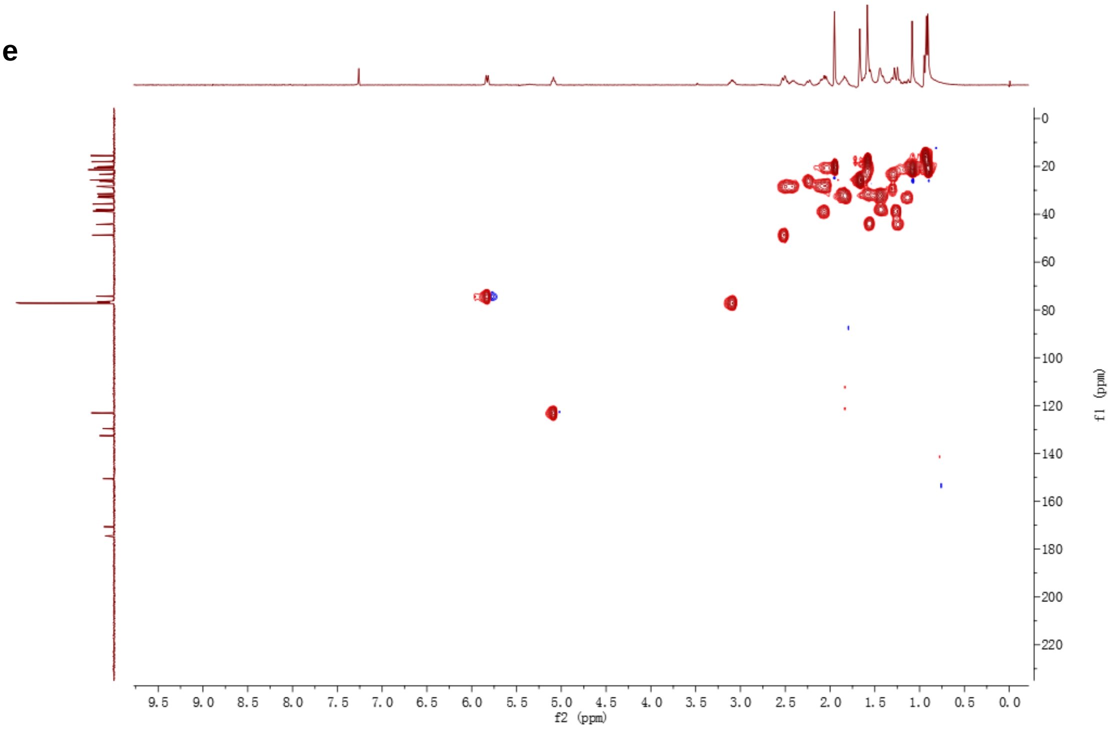

f

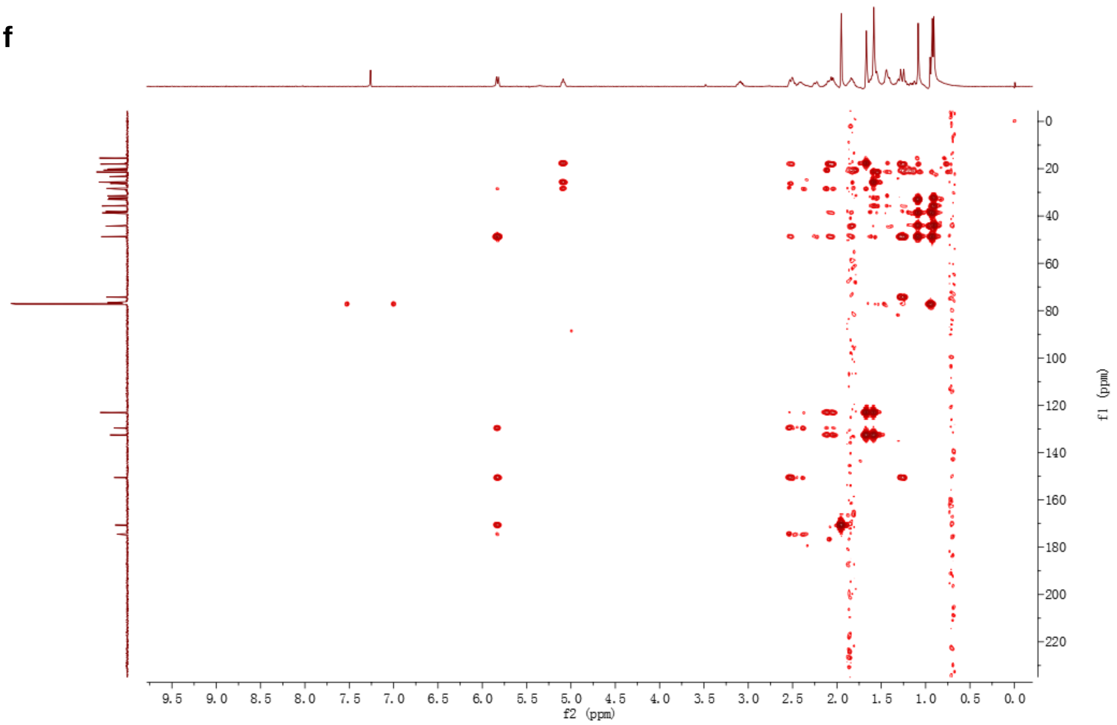

g

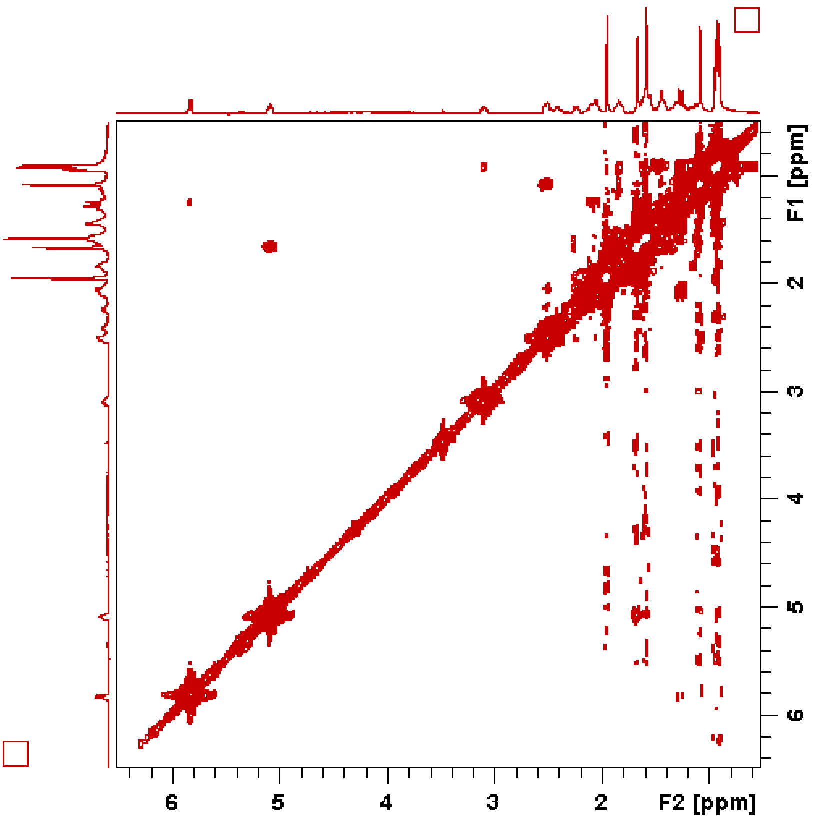

Supplementary Figure 42. HRESIMS and NMR spectra of 15

a HRESIMS spectrum; **b** ^{1}H NMR spectrum in $CDCl_3$ at 400 MHz; **c** ^{13}C NMR spectrum in $CDCl_3$ at 100 MHz; **d** ^{1}H-^{1}H COSY spectrum in $CDCl_3$ at 400 MHz; **e** HSQC spectrum in $CDCl_3$ at 400 MHz; **f** HMBC spectrum in $CDCl_3$ at 400 MHz; **g** NOESY spectrum in $CDCl_3$ at 400 MHz.

a

Single Mass Analysis
Tolerance = 5.0 mDa / DBE: min = -1.5, max = 15.0
Element prediction: Off
Number of isotope peaks used for i-FIT = 3

Monoisotopic Mass, Even Electron Ions
147 formula(e) evaluated with 1 results within limits (up to 50 closest results for each mass)
Elements Used:
C: 0-500 H: 0-1000 O: 0-200
AFU-19
2016101037 184 (1.489) Cm (182:186)

1: TOF MS ES+
2.19e+005

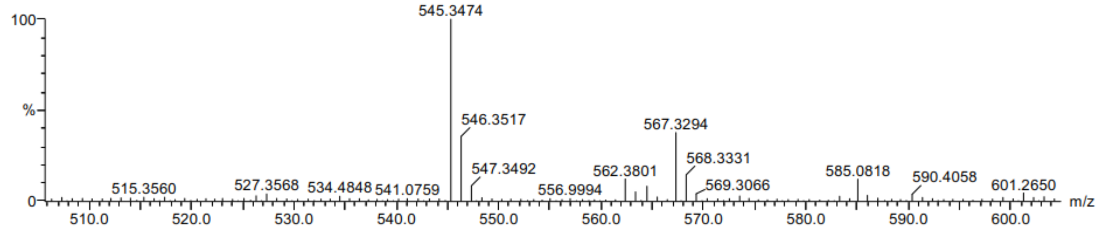

Minimum:				-1.5				
Maximum:		5.0	10.0	15.0				
Mass	Calc. Mass	mDa	PPM	DBE	i-FIT	Norm	Conf(%)	Formula
545.3474	545.3478	-0.4	-0.7	8.5	22.5	n/a	n/a	C32 H49 O7

b

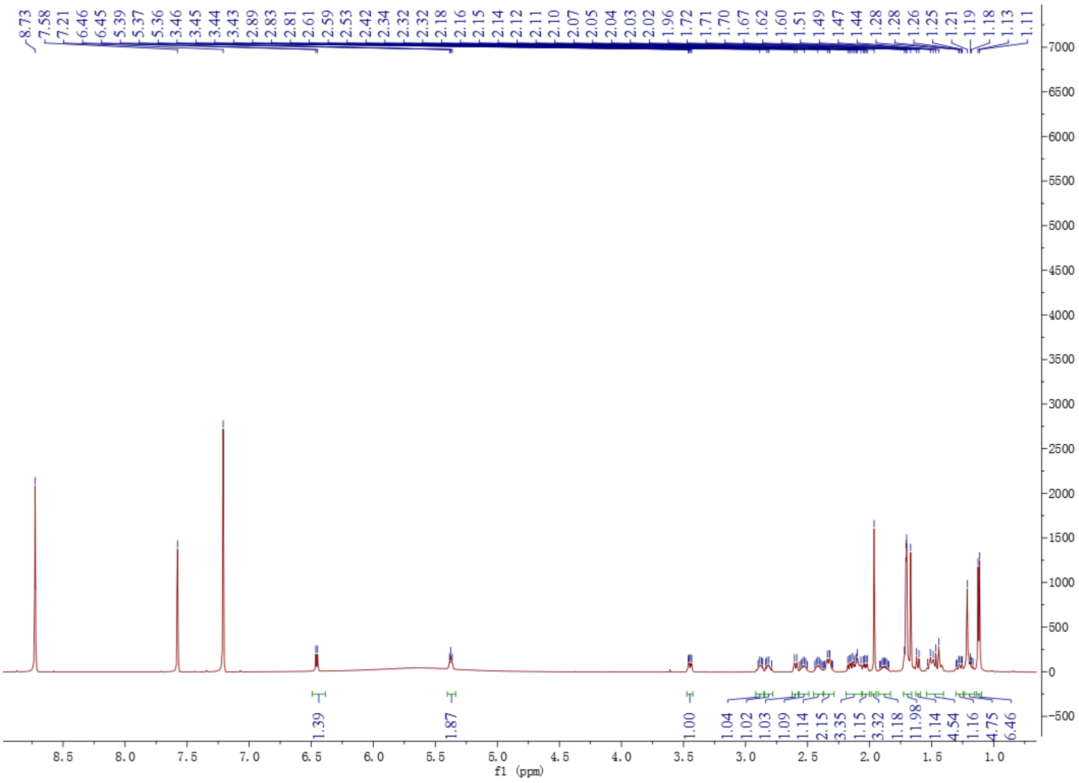

c

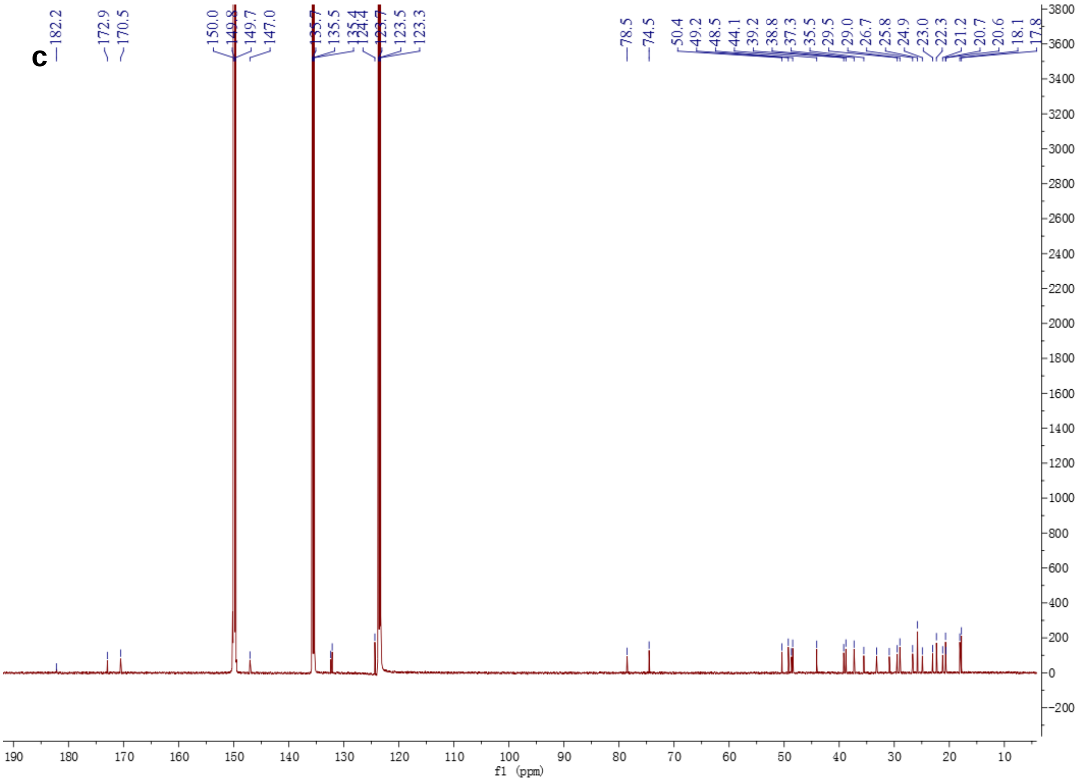
182.2
172.9
170.5
150.0
149.8
149.7
147.0
135.7
135.5
124.4
123.7
123.5
123.3
78.5
74.5
50.4
49.2
48.5
44.1
39.2
38.8
37.3
35.5
29.5
29.0
26.7
25.8
24.9
23.0
22.3
21.2
20.7
20.6
18.1
17.8
f1 (ppm)

d

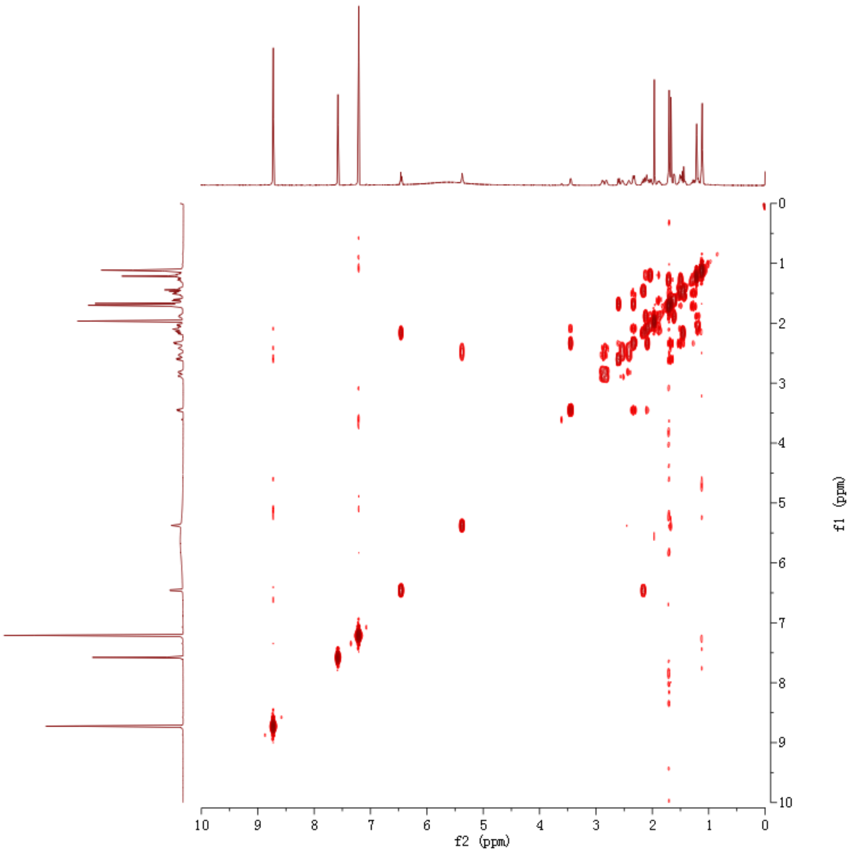
f2 (ppm)
f1 (ppm)

e

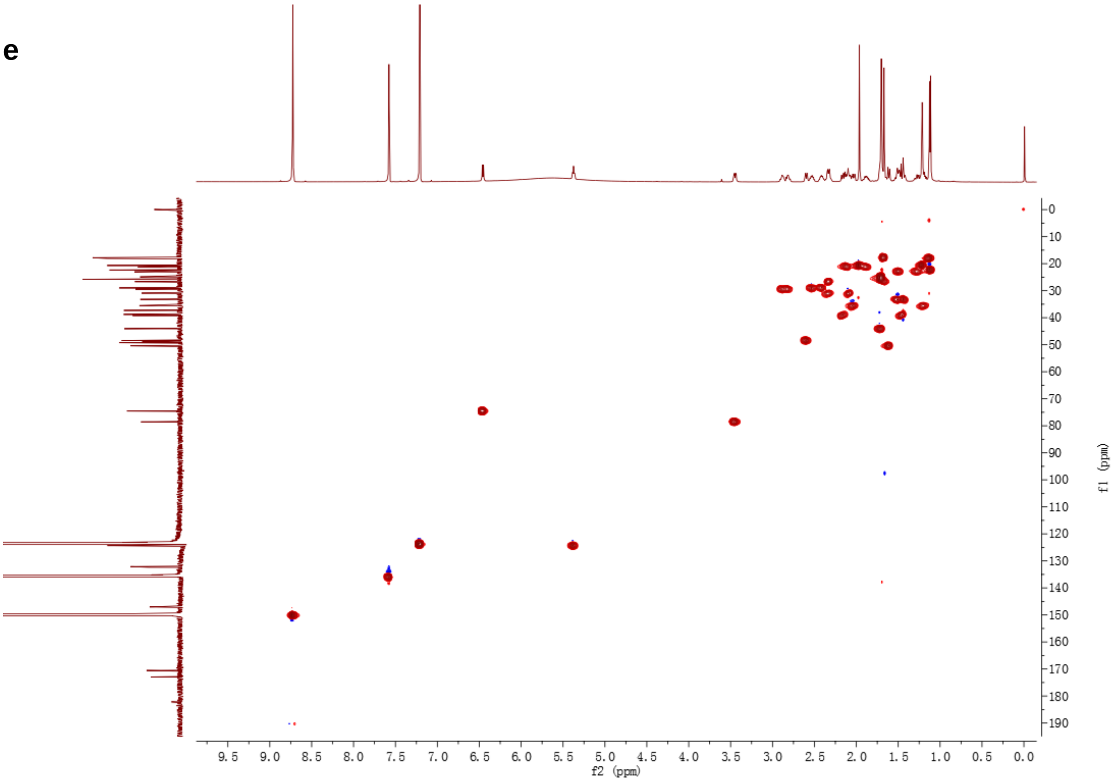

f

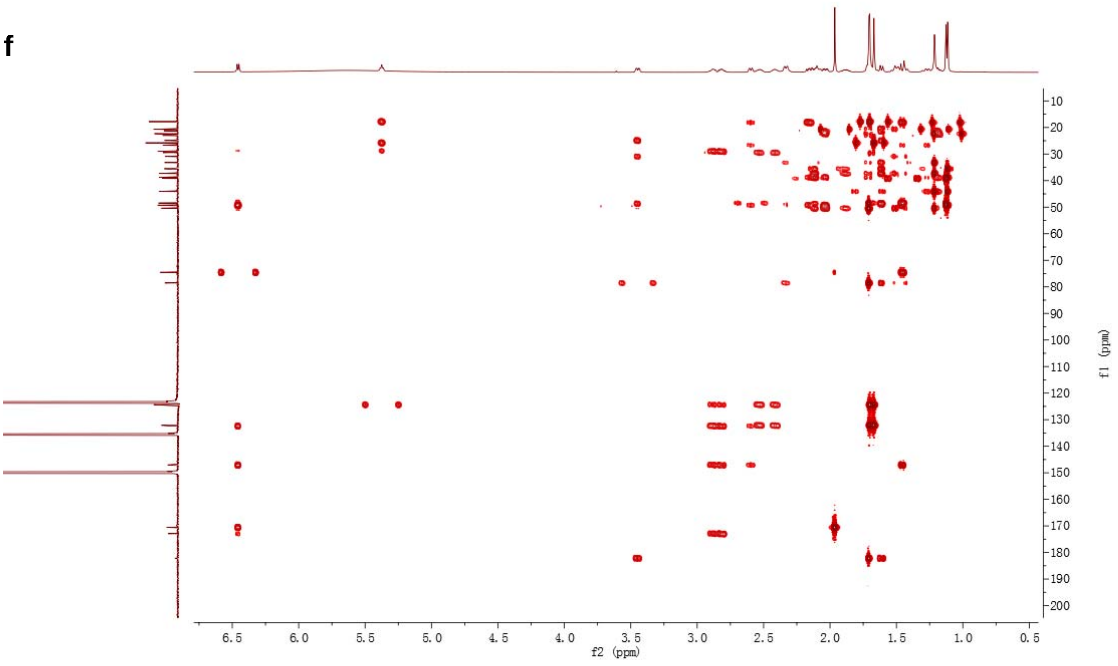

g

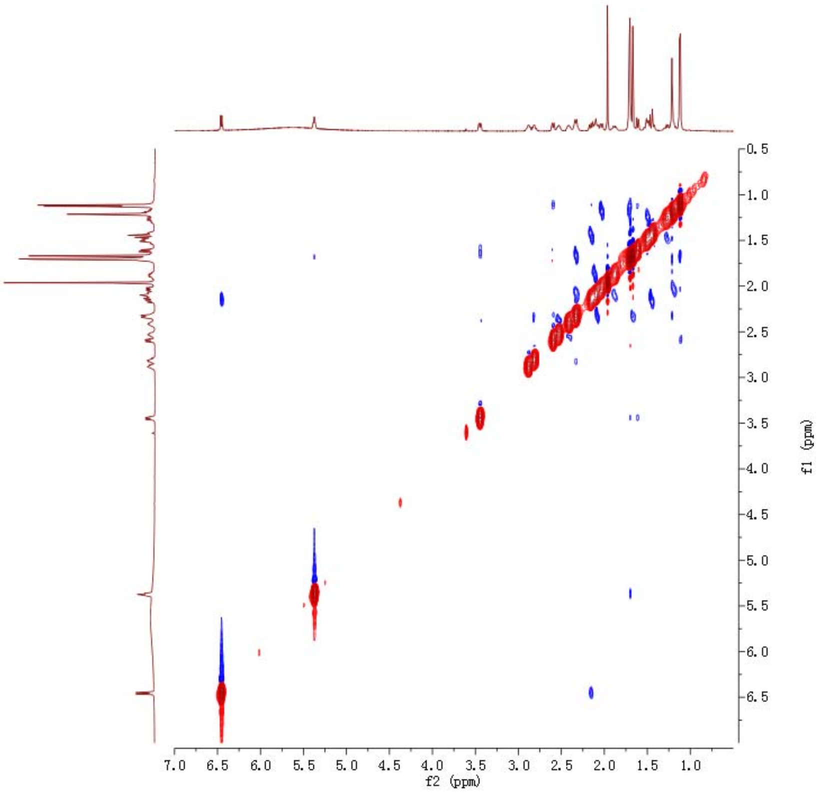

Supplementary Figure 43. HRESIMS and NMR spectra of 16

a HRESIMS spectrum; **b** ^{1}H NMR spectrum in pyridine-d_5 at 600 MHz; **c** ^{13}C NMR spectrum in pyridine-d_5 at 150 MHz; **d** ^{1}H-^{1}H COSY spectrum in pyridine-d_5 at 600 MHz; **e** HSQC spectrum in pyridine-d_5 at 600 MHz; **f** HMBC spectrum in pyridine-d_5 at 600 MHz; **g** ROESY spectrum in pyridine-d_5 at 600 MHz.

a

Single Mass Analysis

Tolerance = 10.0 PPM / DBE: min = -1.5, max = 50.0
Element prediction: Off
Number of isotope peaks used for i-FIT = 3

Monoisotopic Mass, Even Electron Ions
295 formula(e) evaluated with 3 results within limits (up to 20 best isotopic matches for each mass)
Elements Used:
C: 0-800 H: 0-200 O: 0-100 Na: 0-1

AFU-17
2016082228 184 (1.488)

1: TOF MS ES+
5.07e+003

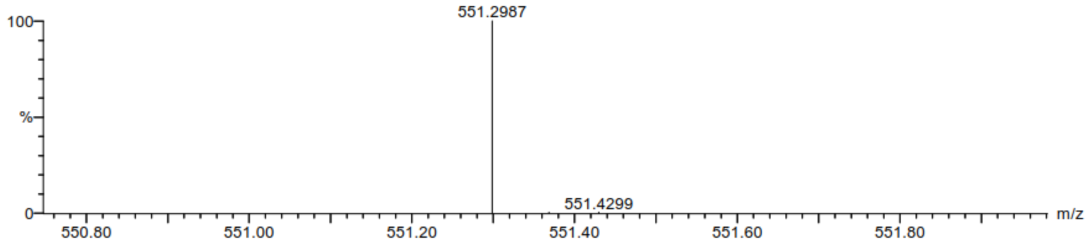

Minimum:				-1.5				
Maximum:		5.0	10.0	50.0				

Mass	Calc. Mass	mDa	PPM	DBE	i-FIT	Norm	Conf(%)	Formula
551.2987	551.2985	0.2	0.4	9.5	40.6	0.595	55.14	C31 H44 O7 Na
	551.3009	-2.2	-4.0	12.5	41.1	1.160	31.36	C33 H43 O7
	551.2950	3.7	6.7	21.5	42.0	2.002	13.50	C40 H39 O2

b

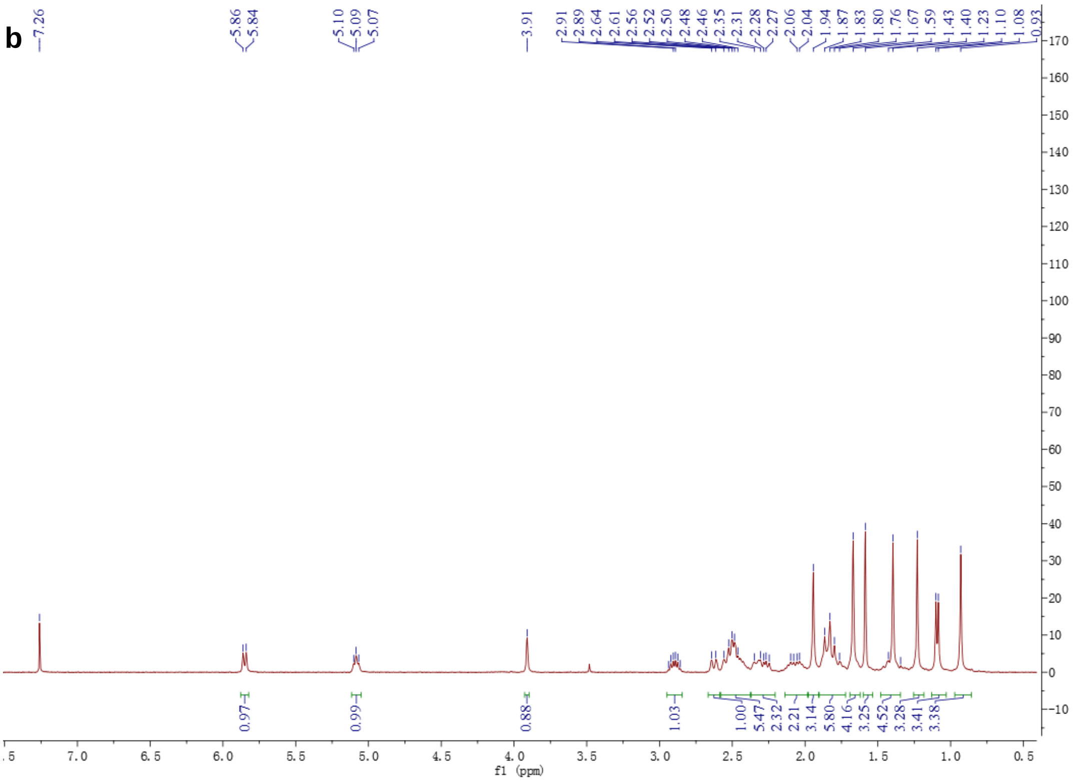

c

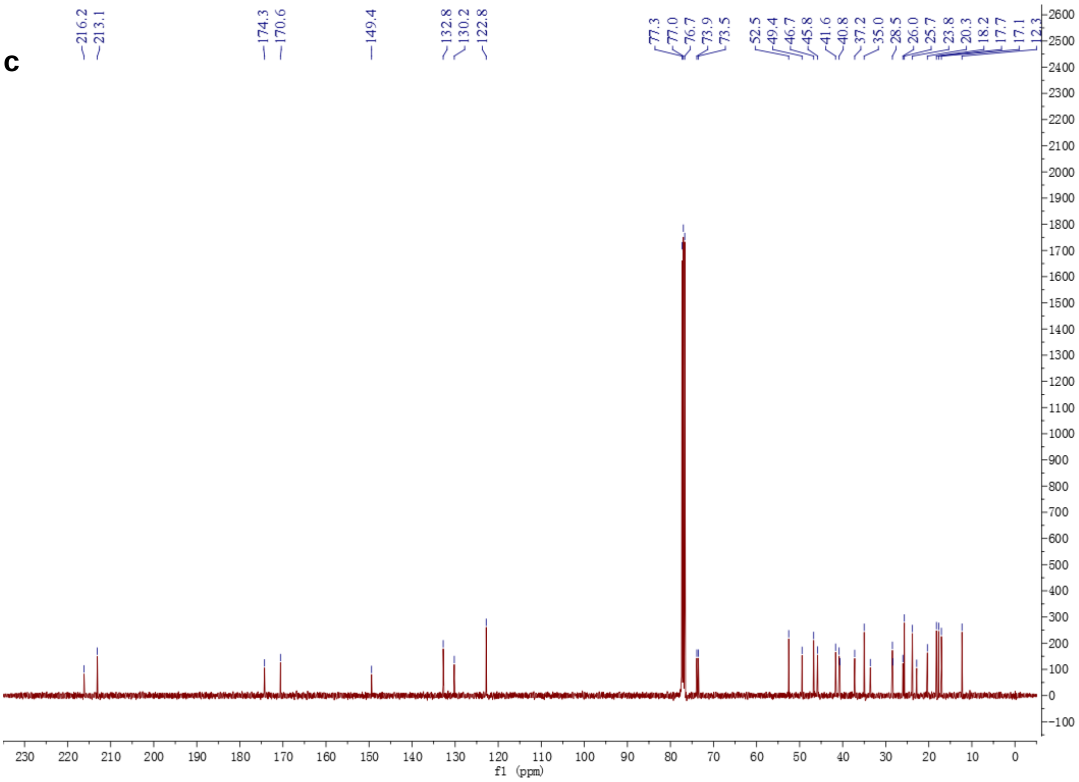

216.2
213.1
174.3
170.6
149.4
132.8
130.2
122.8
77.3
77.0
76.7
73.9
73.5
52.5
49.4
46.7
45.8
41.6
40.8
37.2
35.0
28.5
26.0
25.7
23.8
20.3
18.2
17.7
17.1
12.3
f1 (ppm)

d

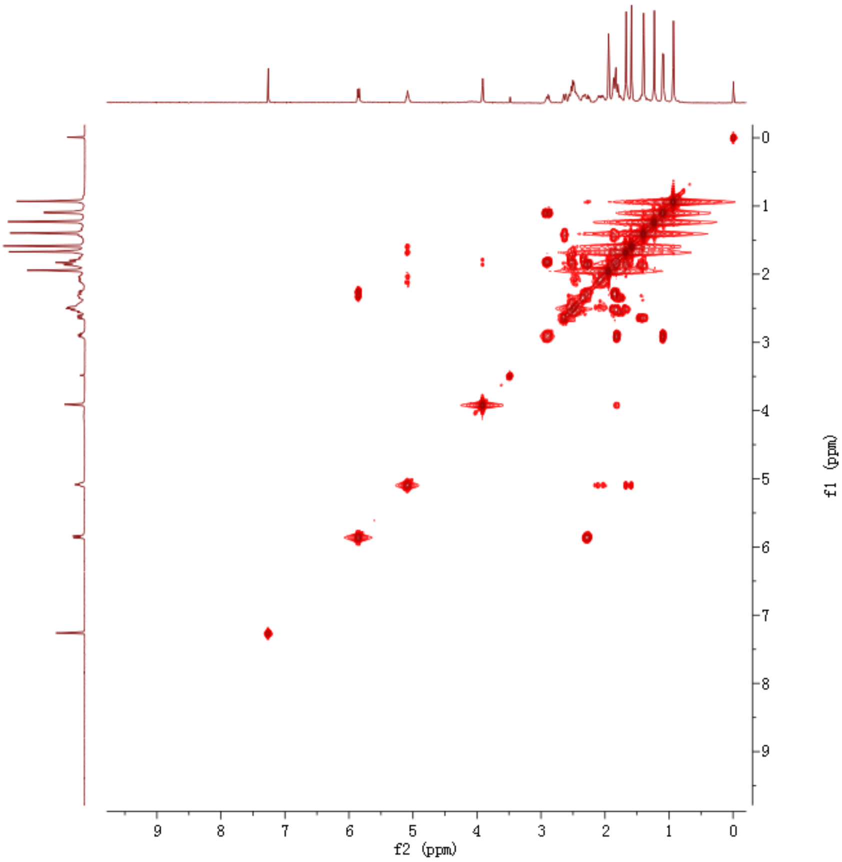

f2 (ppm)
f1 (ppm)

e

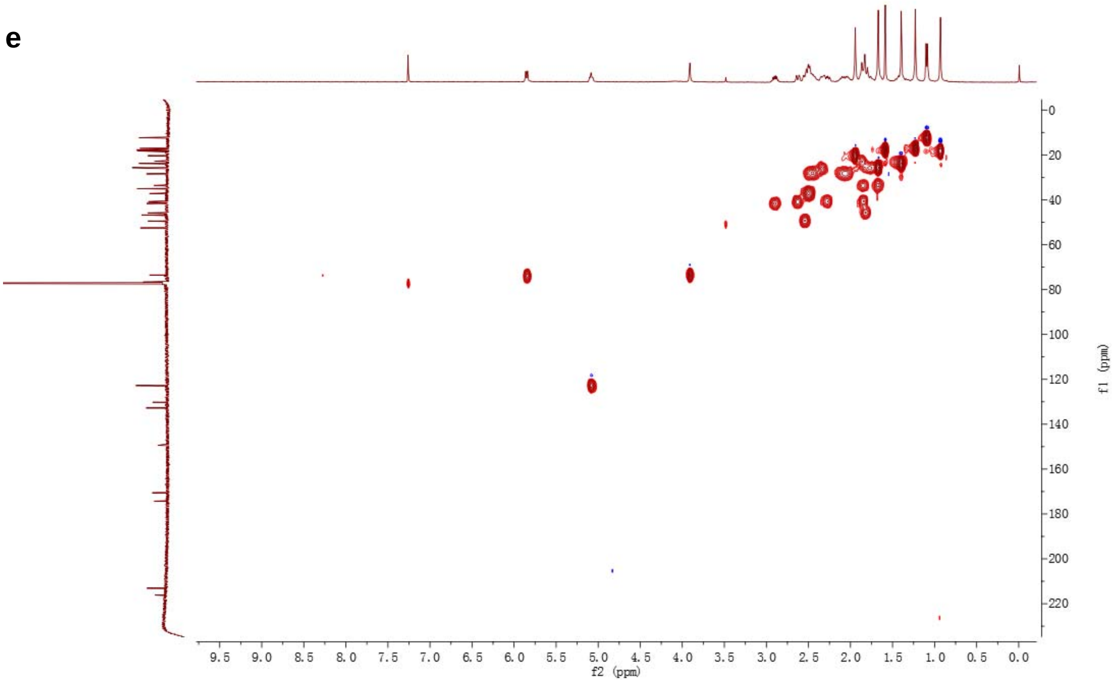

f

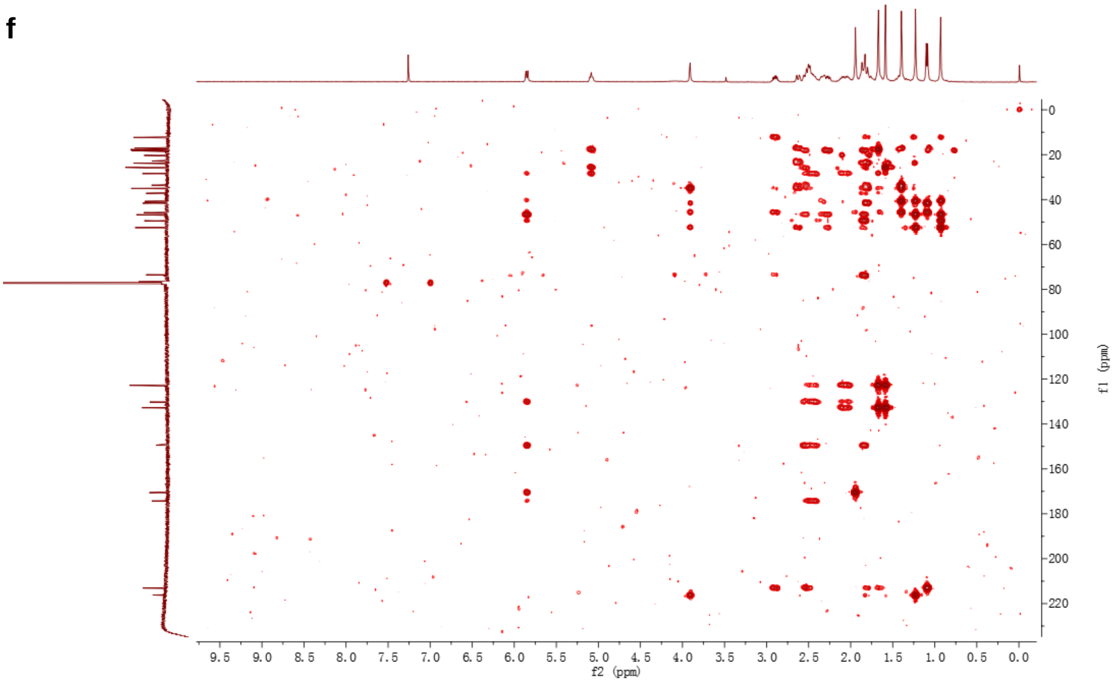

g

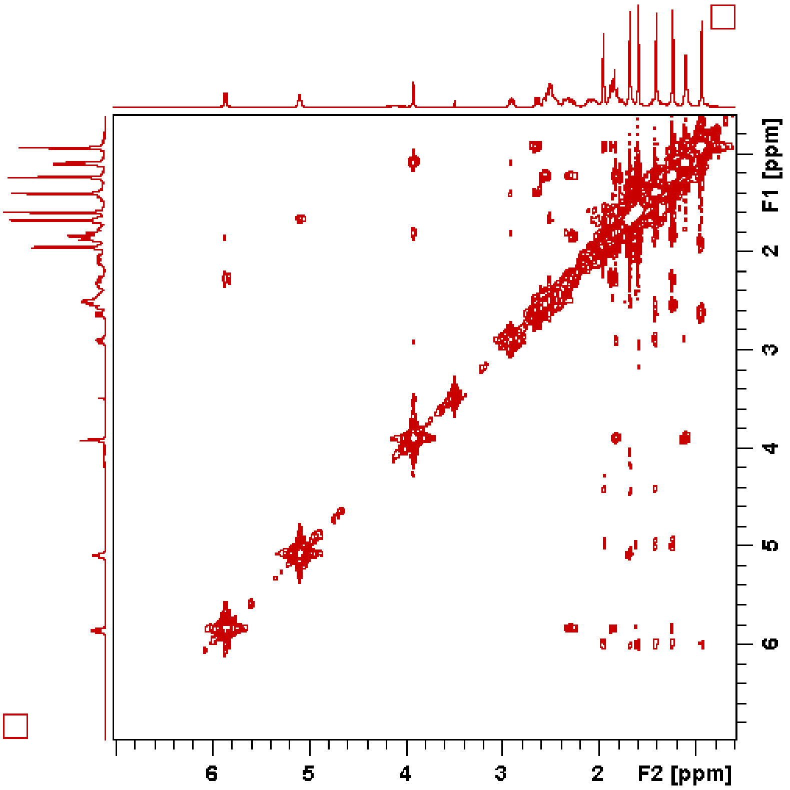

Supplementary Figure 44. HRESIMS and NMR spectra of 17

a HRESIMS spectrum; **b** ^{1}H NMR spectrum in $CDCl_3$ at 400 MHz; **c** ^{13}C NMR spectrum in $CDCl_3$ at 100 MHz; **d** ^{1}H-^{1}H COSY spectrum in $CDCl_3$ at 400 MHz; **e** HSQC spectrum in $CDCl_3$ at 400 MHz; **f** HMBC spectrum in $CDCl_3$ at 400 MHz; **g** NOESY spectrum in $CDCl_3$ at 400 MHz.

a

Single Mass Analysis
Tolerance = 10.0 PPM / DBE: min = -1.5, max = 50.0
Element prediction: Off
Number of isotope peaks used for i-FIT = 3

Monoisotopic Mass, Even Electron Ions
285 formula(e) evaluated with 3 results within limits (up to 20 best isotopic matches for each mass)
Elements Used:
C: 0-800 H: 0-200 O: 0-100 Na: 0-1
AFU-16
2016082227 164 (1.328)

1: TOF MS ES+
3.79e+003

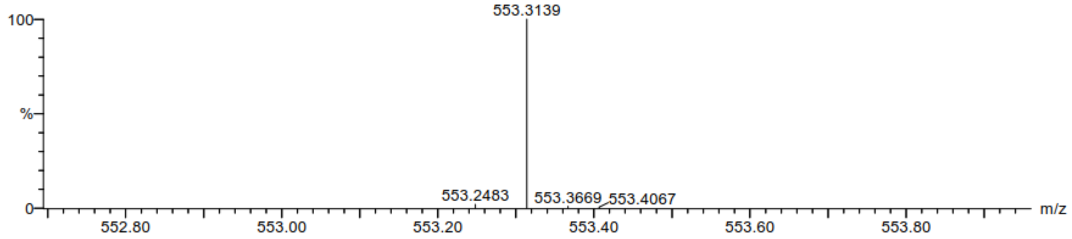

Minimum:				-1.5				
Maximum:		5.0	10.0	50.0				
Mass	Calc. Mass	mDa	PPM	DBE	i-FIT	Norm	Conf(%)	Formula
553.3139	553.3141	-0.2	-0.4	8.5	40.7	0.579	56.02	C31 H46 O7 Na
	553.3165	-2.6	-4.7	11.5	41.4	1.329	26.48	C33 H45 O7
	553.3107	3.2	5.8	20.5	41.8	1.743	17.50	C40 H41 O2

b

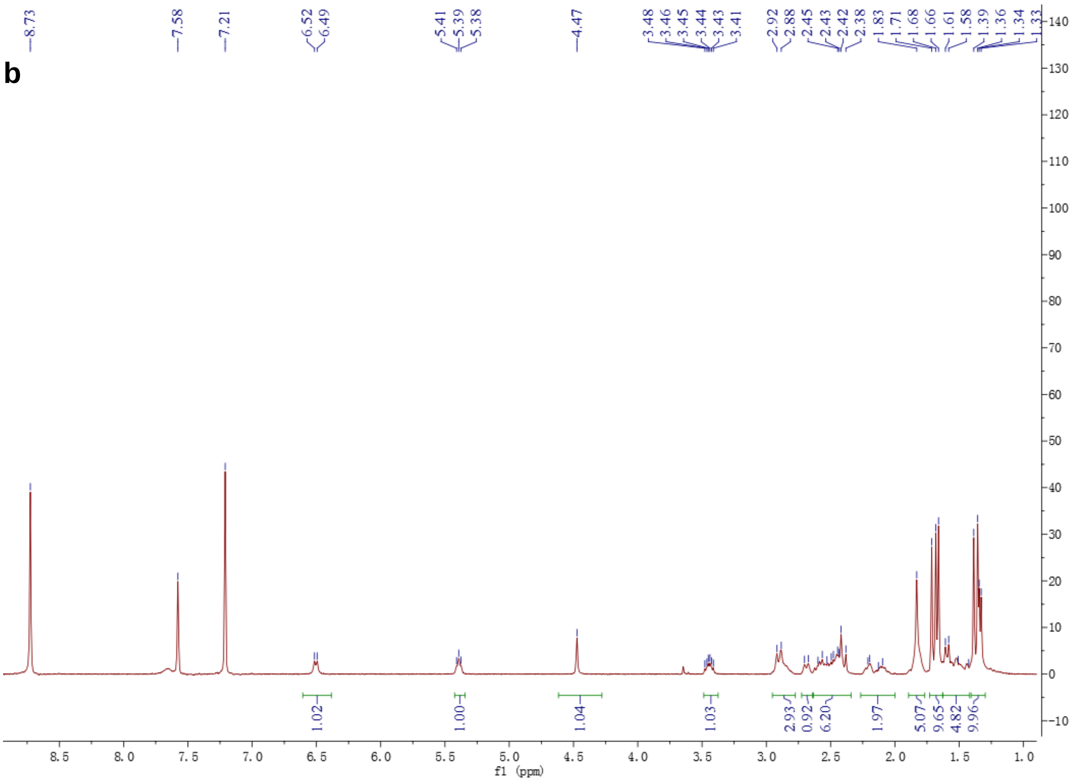

c

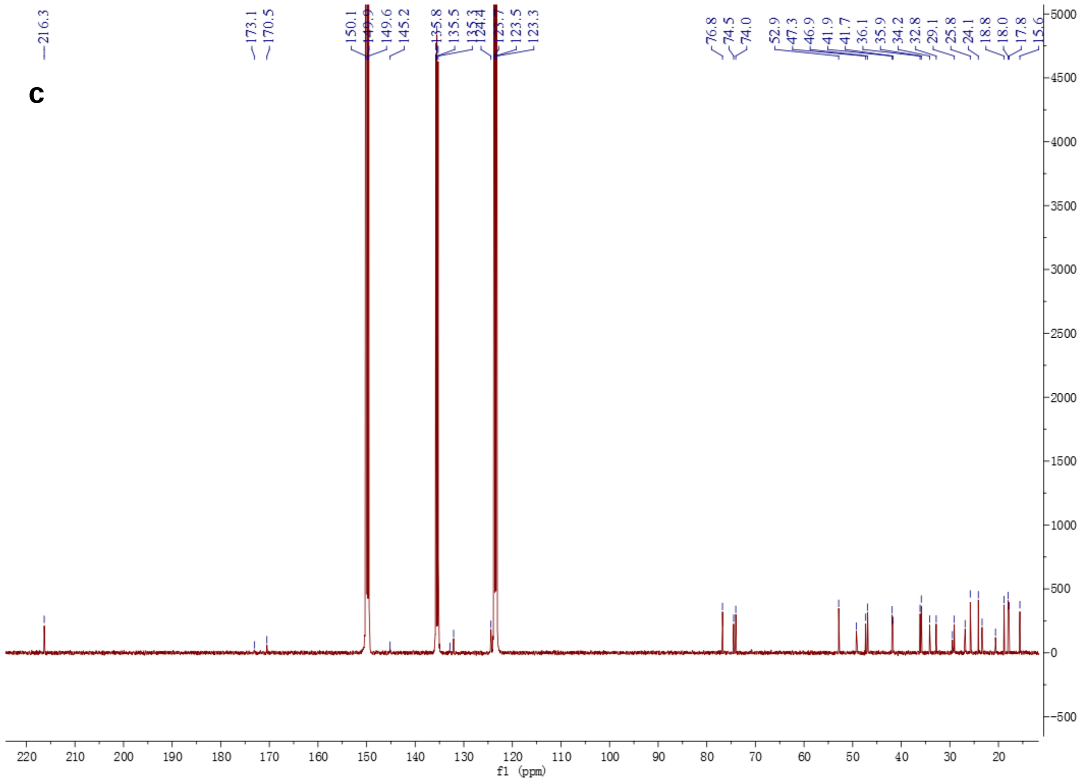

216.3
173.1
170.5
150.1
149.6
145.2
135.8
135.5
135.3
124.4
123.7
123.5
123.3
76.8
74.5
74.0
52.9
47.3
46.9
41.9
41.7
36.1
35.9
34.2
32.8
29.1
25.8
24.1
18.8
18.0
17.8
15.6
f1 (ppm)

d

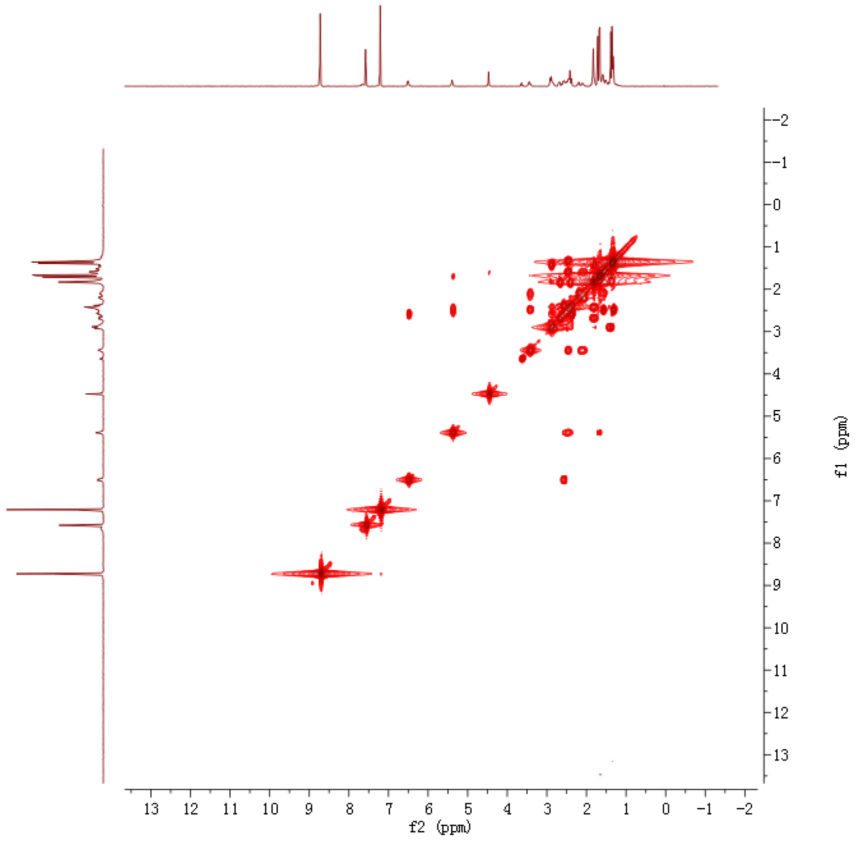

f2 (ppm)
f1 (ppm)

e

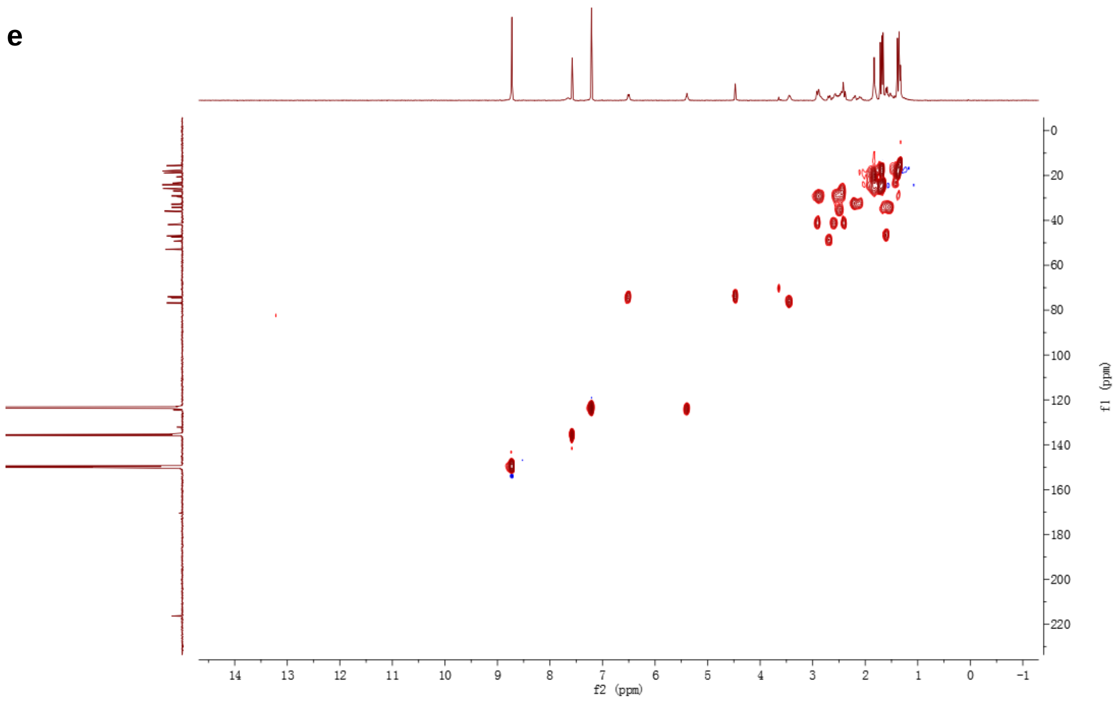

f

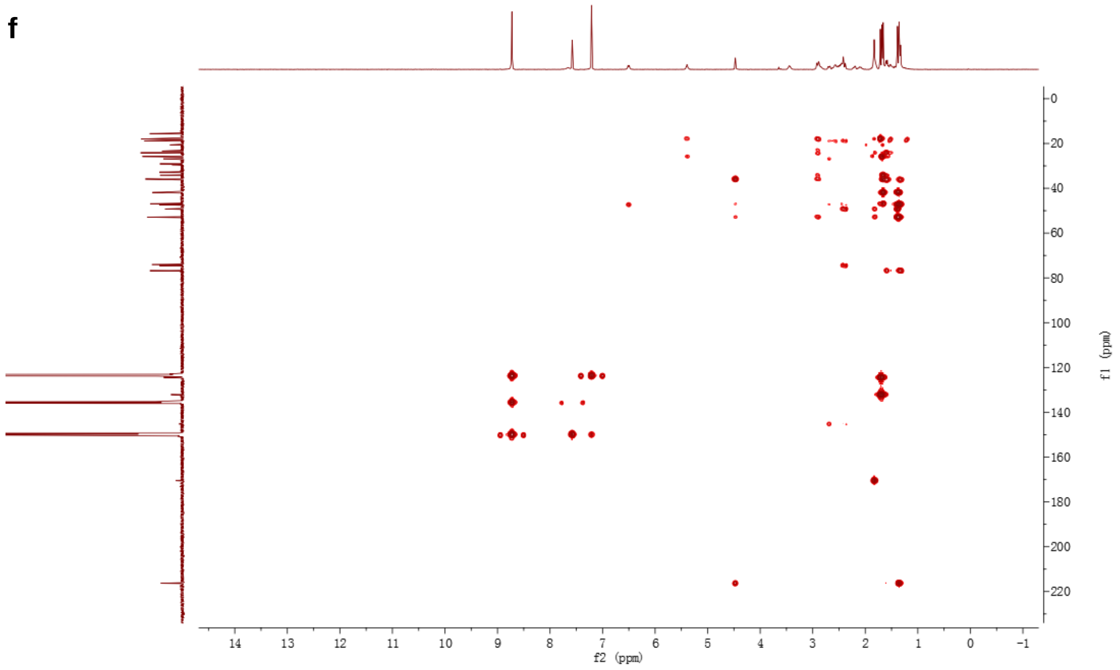

g

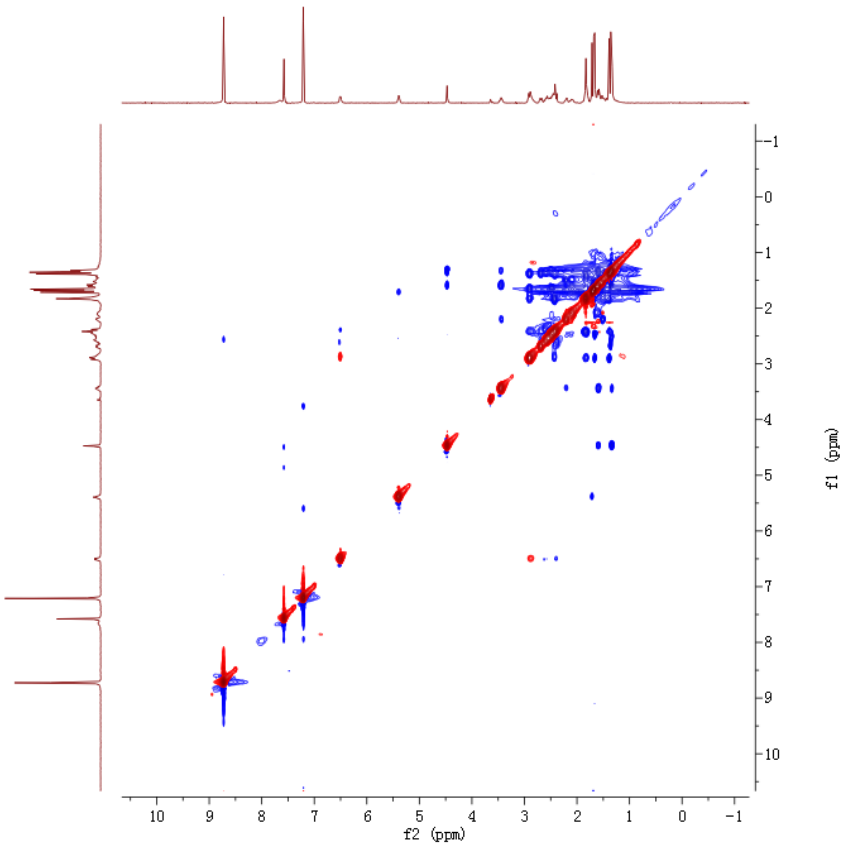

Supplementary Figure 45. HRESIMS and NMR spectra of 18

a HRESIMS spectrum; **b** ^{1}H NMR spectrum in pyridine-d_5 at 400 MHz; **c** ^{13}C NMR spectrum in pyridine-d_5 at 100 MHz; **d** ^{1}H-^{1}H COSY spectrum in pyridine-d_5 at 400 MHz; **e** HSQC spectrum in pyridine-d_5 at 400 MHz; **f** HMBC spectrum in pyridine-d_5 at 400 MHz; **g** ROESY spectrum in pyridine-d_5 at 400 MHz.

a

Single Mass Analysis
Tolerance = 5.0 mDa / DBE: min = -1.5, max = 15.0
Element prediction: Off
Number of isotope peaks used for i-FIT = 3

Monoisotopic Mass, Even Electron Ions
125 formula(e) evaluated with 1 results within limits (up to 50 closest results for each mass)
Elements Used:
C: 0-500 H: 0-1000 O: 0-200
AFU-20
2016101709 218 (1.765) Cm (218)

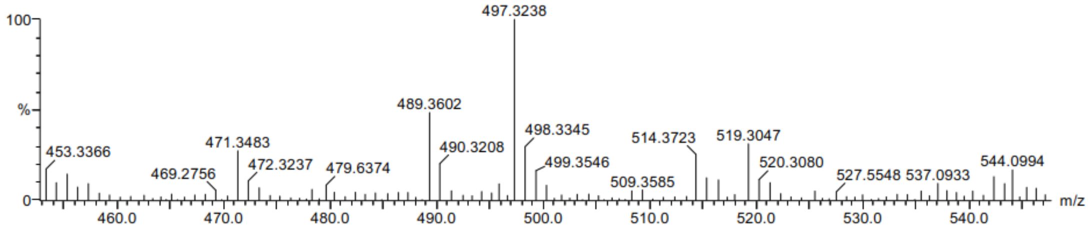

Minimum:				-1.5				
Maximum:		5.0	10.0	15.0				
Mass	Calc. Mass	mDa	PPM	DBE	i-FIT	Norm	Conf(%)	Formula
497.3238	497.3267	-2.9	-5.8	9.5	30.2	n/a	n/a	C31 H45 O5

b

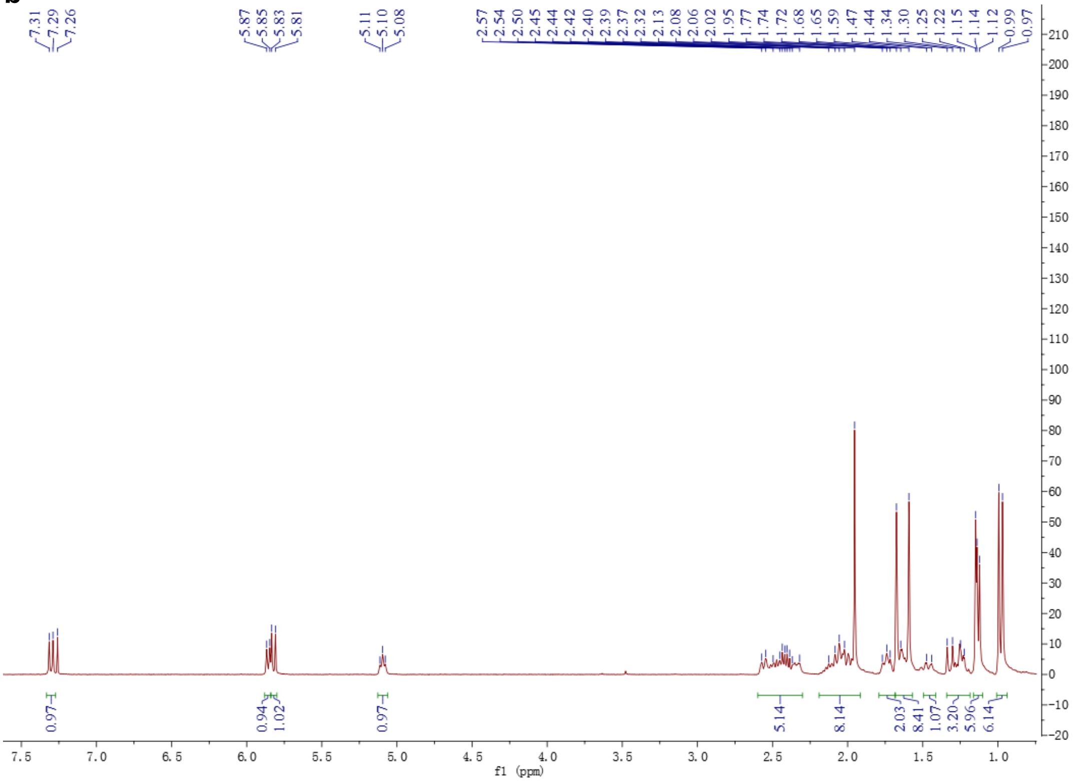

c

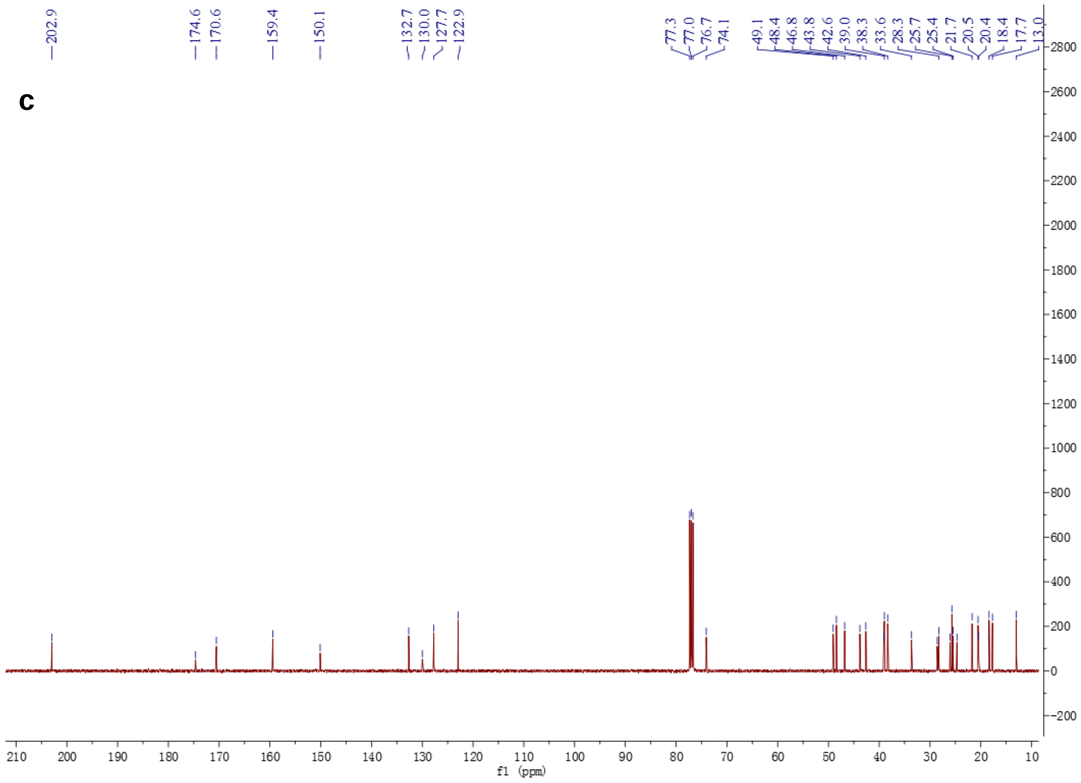

202.9
174.6
170.6
159.4
150.1
132.7
130.0
127.7
122.9
77.3
77.0
76.7
74.1
49.1
48.4
46.8
43.8
42.6
39.0
38.3
33.6
28.3
25.7
25.4
21.7
20.5
20.4
18.4
17.7
13.0
f1 (ppm)

d

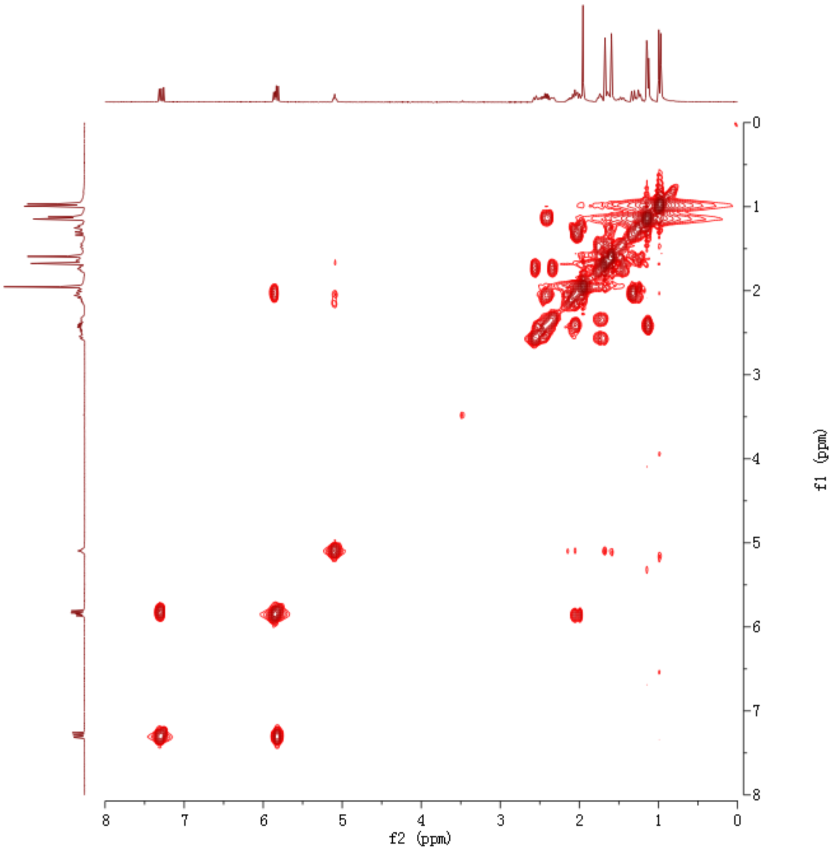

f2 (ppm)
f1 (ppm)

e

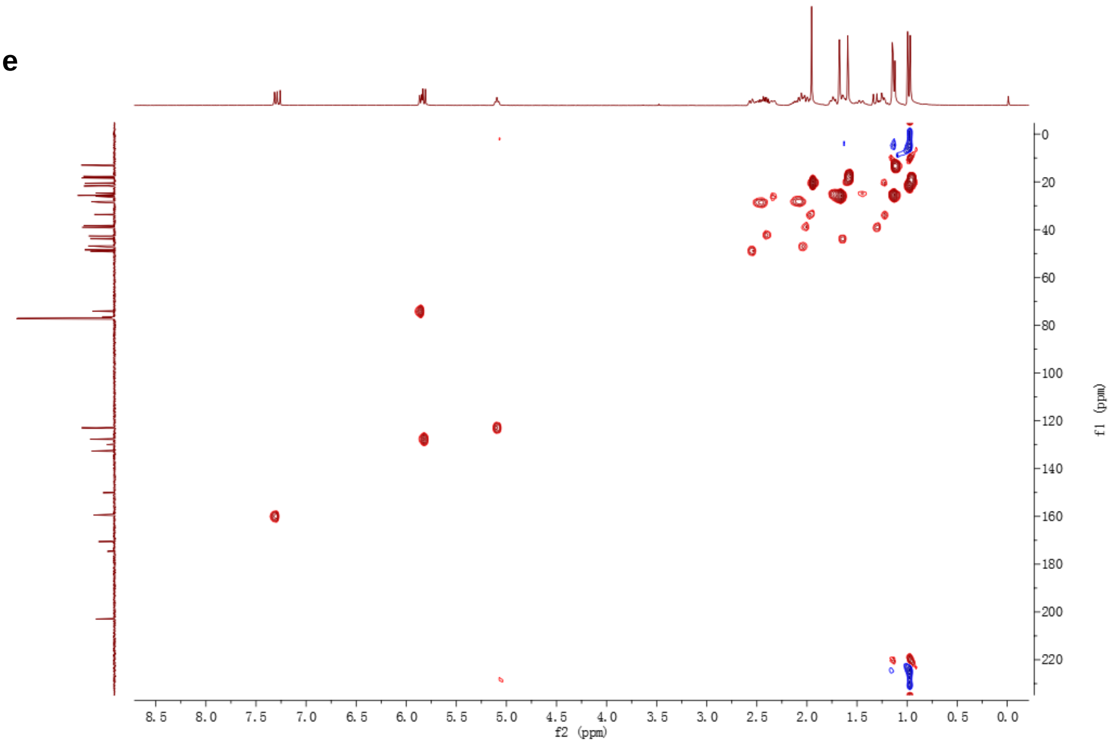

f

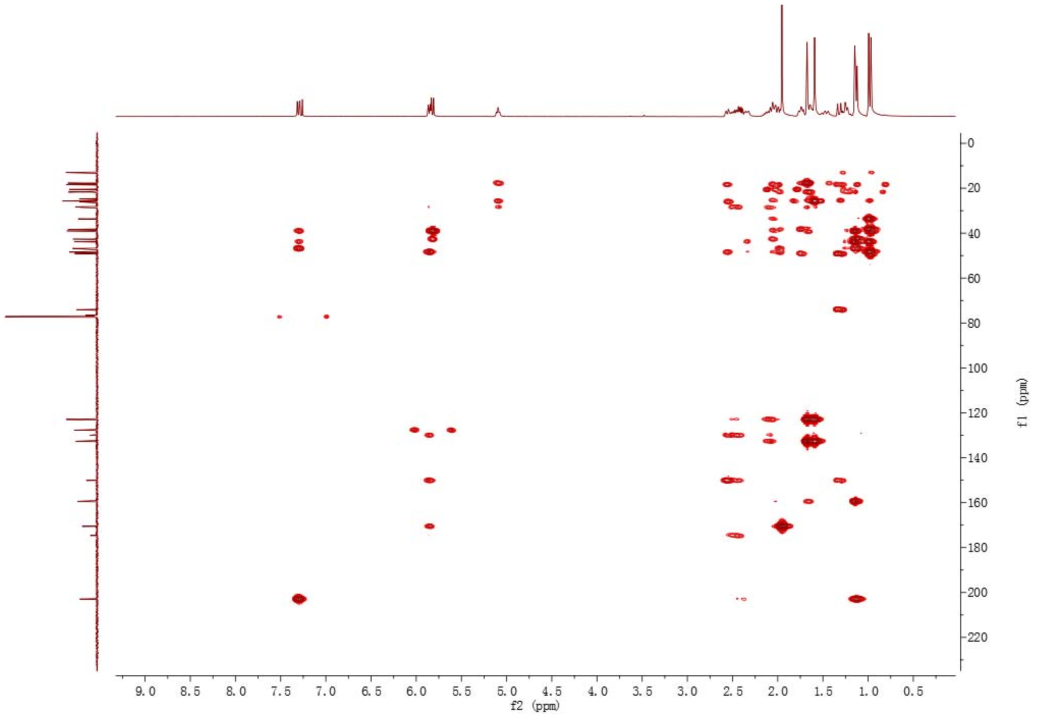

g

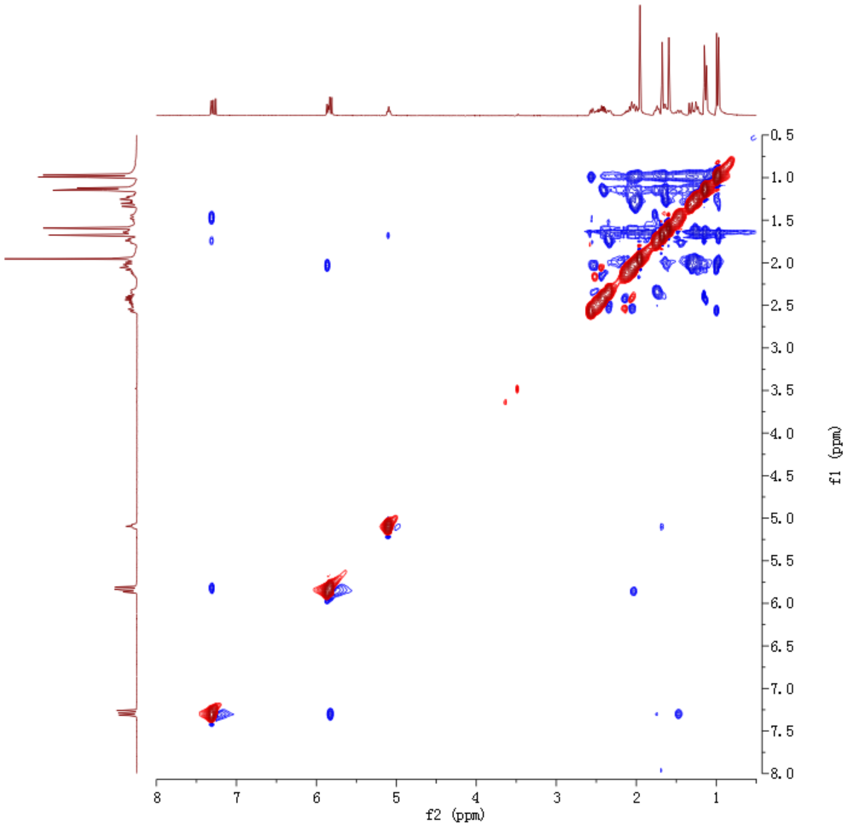

Supplementary Figure 46. HRESIMS and NMR spectra of 19

a HRESIMS spectrum; **b** ^{1}H NMR spectrum in $CDCl_3$ at 400 MHz; **c** ^{13}C NMR spectrum in $CDCl_3$ at 100 MHz; **d** ^{1}H-^{1}H COSY spectrum in $CDCl_3$ at 400 MHz; **e** HSQC spectrum in $CDCl_3$ at 400 MHz; **f** HMBC spectrum in $CDCl_3$ at 400 MHz; **g** ROESY spectrum in $CDCl_3$ at 400 MHz.

a

Single Mass Analysis

Tolerance = 5.0 mDa / DBE: min = -1.5, max = 15.0
Element prediction: Off
Number of isotope peaks used for i-FIT = 3

Monoisotopic Mass, Even Electron Ions
324 formula(e) evaluated with 2 results within limits (up to 50 closest results for each mass)
Elements Used:
C: 0-500 H: 0-1000 O: 0-200 Na: 0-1

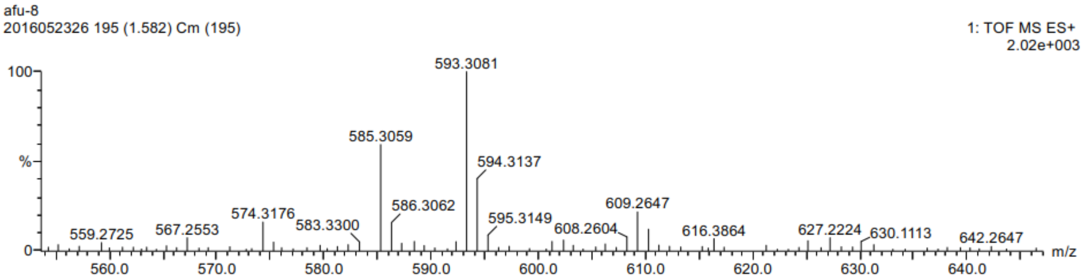

Minimum:				-1.5				
Maximum:		5.0	10.0	15.0				

Mass	Calc. Mass	mDa	PPM	DBE	i-FIT	Norm	Conf(%)	Formula
593.3081	593.3090	-0.9	-1.5	10.5	8.8	0.838	43.25	C33 H46 O8 Na
	593.3114	-3.3	-5.6	13.5	8.5	0.566	56.75	C35 H45 O8

b

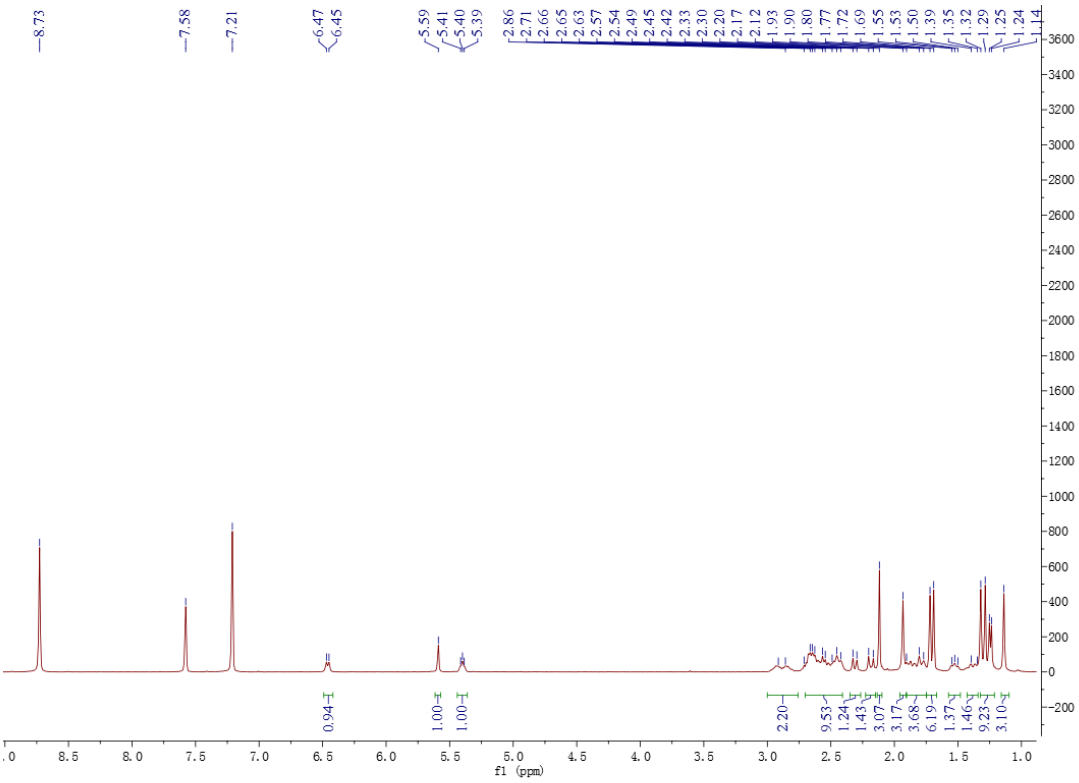

c

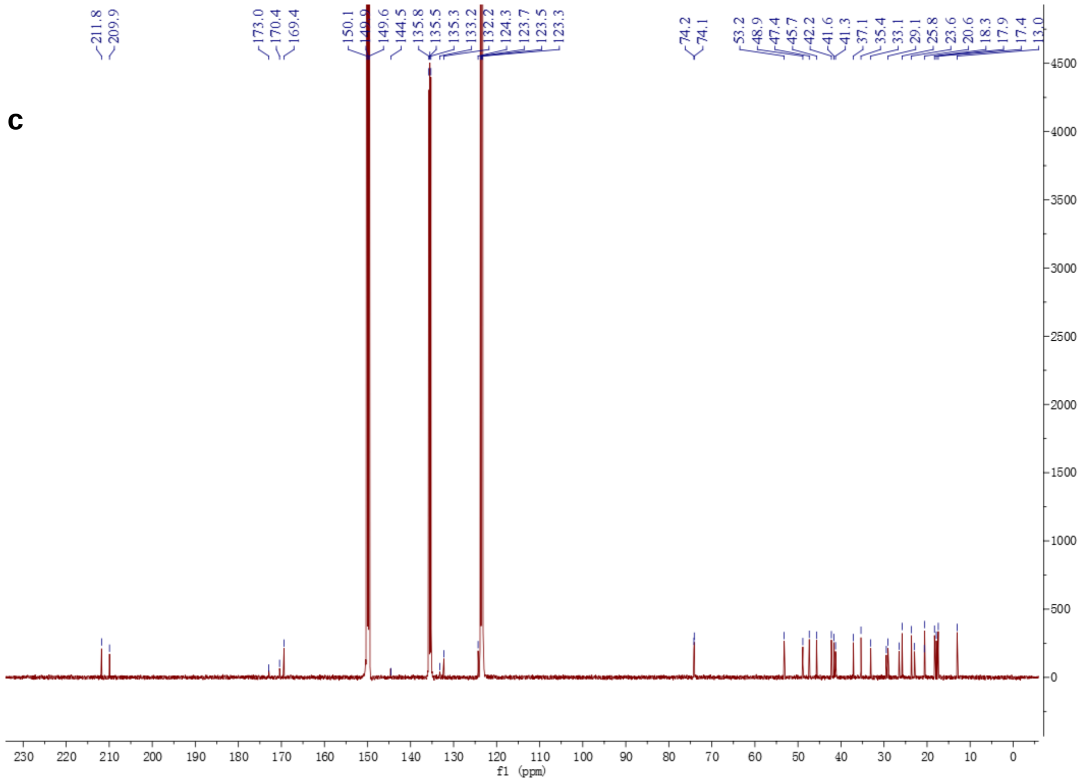

Supplementary Figure 47. HRESIMS and NMR spectra of 20

a HRESIMS spectrum; **b** ^{1}H NMR spectrum in pyridine-d_5 at 400 MHz; **c** ^{13}C NMR spectrum in pyridine-d_5 at 100 MHz.

a

Single Mass Analysis
Tolerance = 5.0 mDa / DBE: min = -1.5, max = 50.0
Element prediction: Off
Number of isotope peaks used for i-FIT = 3

Monoisotopic Mass, Even Electron Ions
123 formula(e) evaluated with 1 results within limits (up to 50 best isotopic matches for each mass)
Elements Used:
C: 0-80 H: 0-100 O: 0-20
AFU-21
2016102407 140 (1.141)

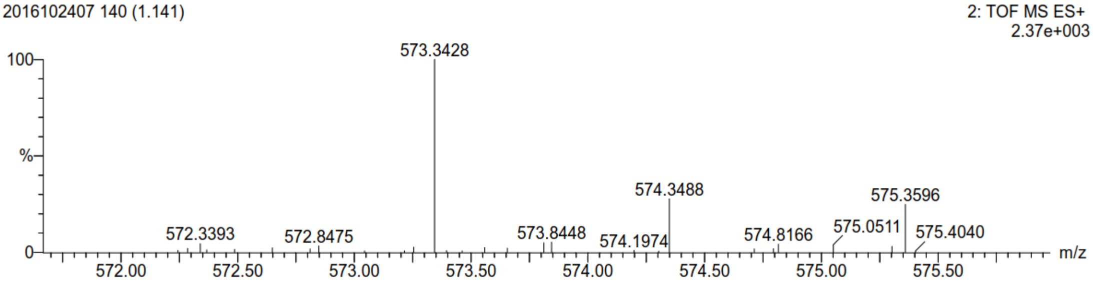

Minimum:				-1.5				
Maximum:		5.0	10.0	50.0				
Mass	Calc. Mass	mDa	PPM	DBE	i-FIT	Norm	Conf(%)	Formula
573.3428	573.3427	0.1	0.2	9.5	127.5	n/a	n/a	C33 H49 O8

b

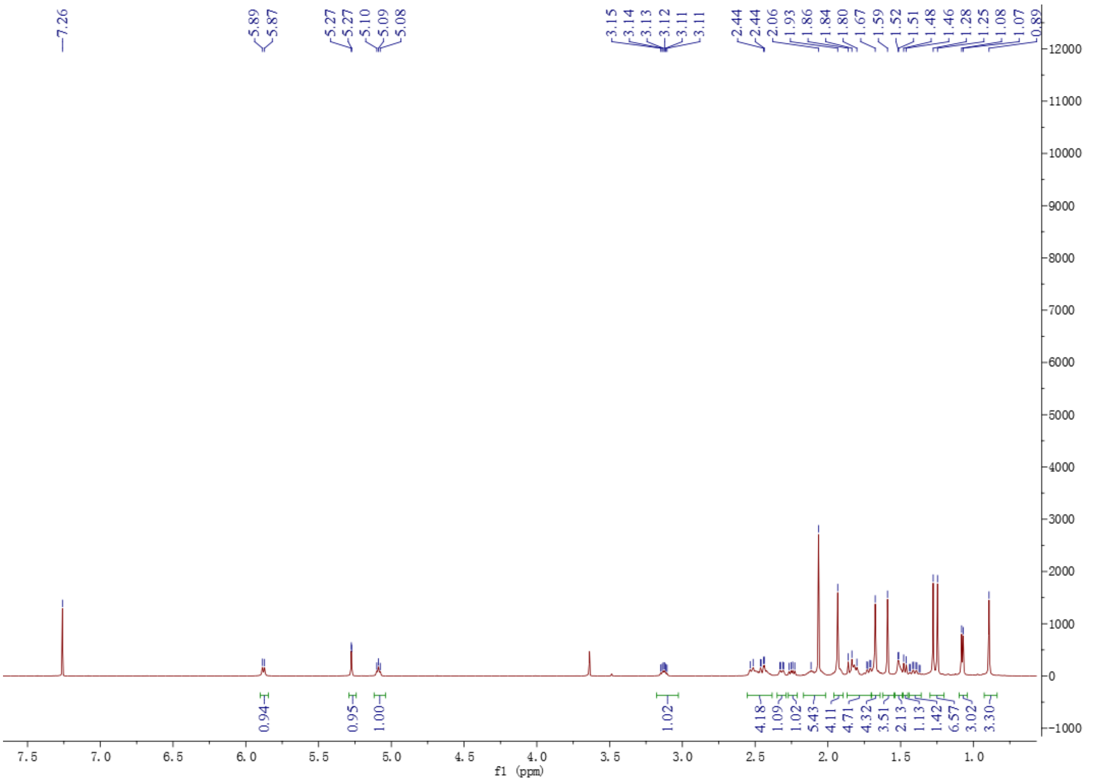

c

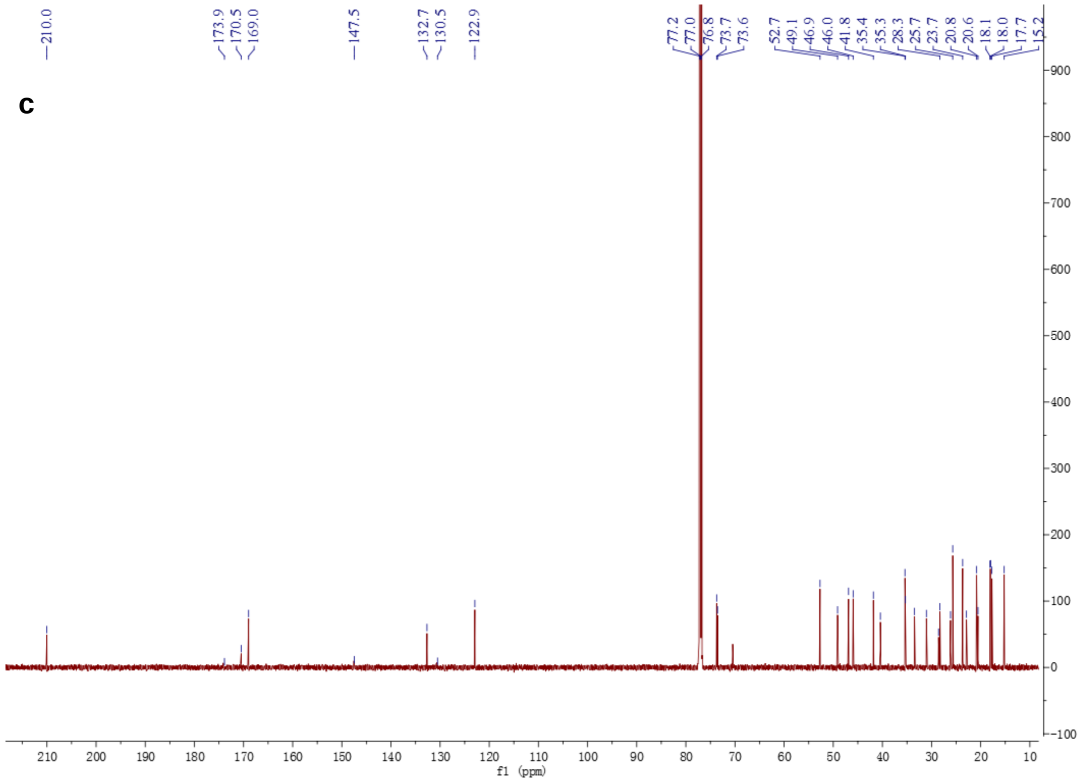

210.0
173.9
170.5
169.0
147.5
132.7
130.5
122.9
77.2
77.0
76.8
73.7
73.6
52.7
49.1
46.9
46.0
41.8
35.4
35.3
28.3
25.7
23.7
20.8
20.6
18.1
18.0
17.7
15.2
f1 (ppm)

d

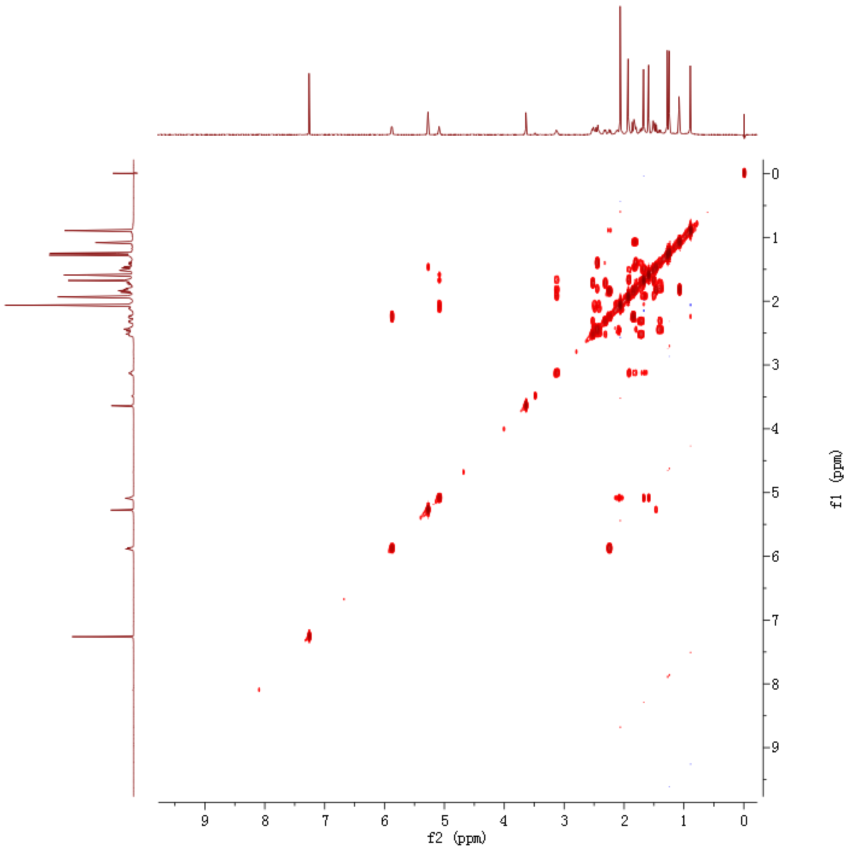

f2 (ppm)
f1 (ppm)

e

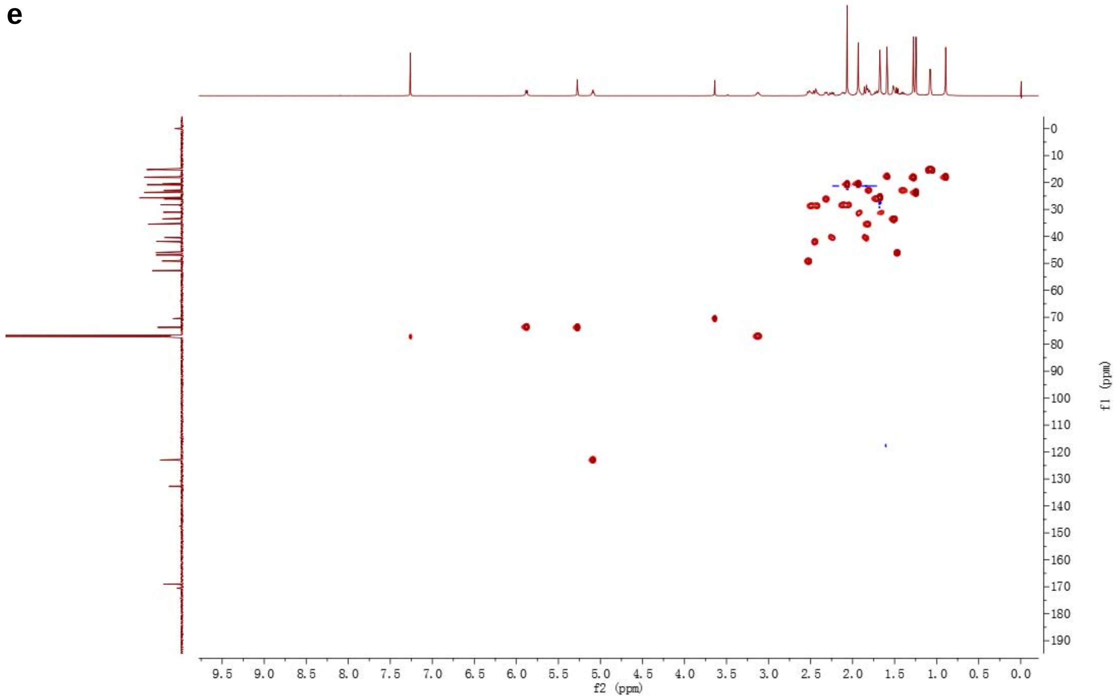

f

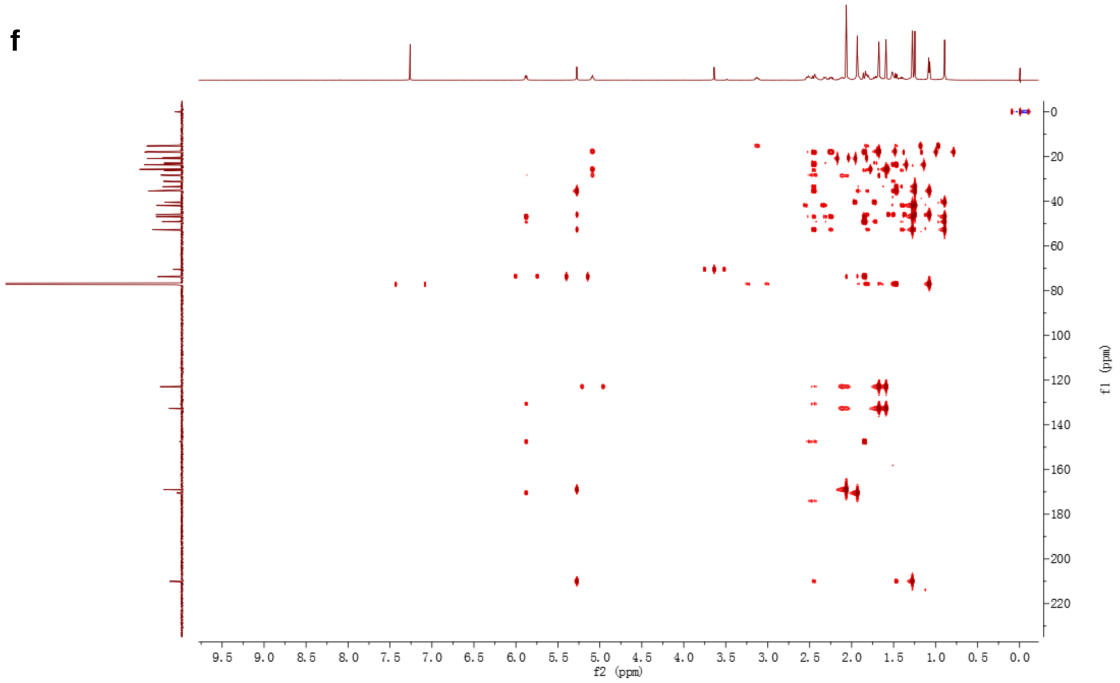

g

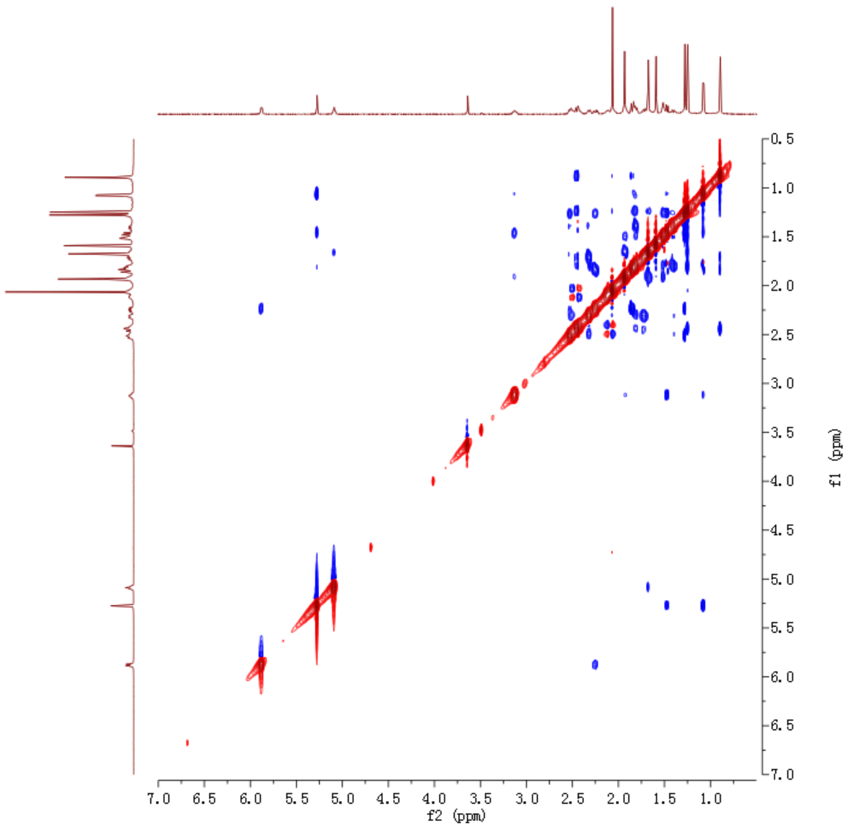

Supplementary Figure 48. HRESIMS and NMR spectra of 21

a HRESIMS spectrum; **b** ^{1}H NMR spectrum in $CDCl_3$ at 600 MHz; **c** ^{13}C NMR spectrum in $CDCl_3$ at 150 MHz; **d** ^{1}H-^{1}H COSY spectrum in $CDCl_3$ at 600 MHz; **e** HSQC spectrum in $CDCl_3$ at 600 MHz; **f** HMBC spectrum in $CDCl_3$ at 600 MHz; **g** ROESY spectrum in $CDCl_3$ at 600 MHz.

a

Single Mass Analysis
Tolerance = 5.0 mDa / DBE: min = -1.5, max = 50.0
Element prediction: Off
Number of isotope peaks used for i-FIT = 3

Monoisotopic Mass, Even Electron Ions
115 formula(e) evaluated with 1 results within limits (up to 50 best isotopic matches for each mass)
Elements Used:
C: 0-80 H: 0-100 O: 0-20

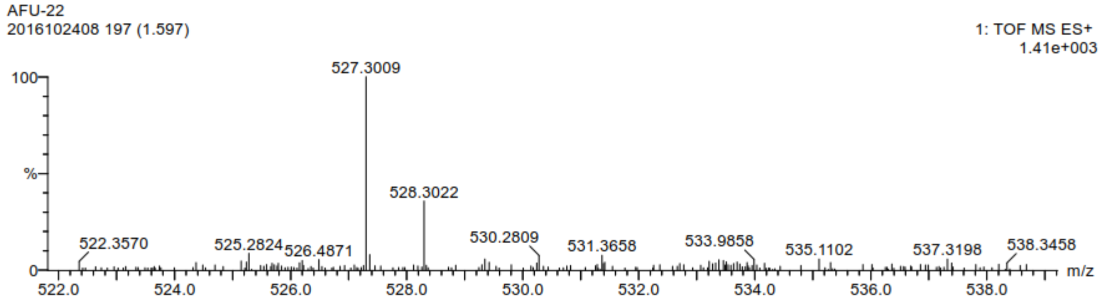

Minimum:				-1.5				
Maximum:		5.0	10.0	50.0				

Mass	Calc. Mass	mDa	PPM	DBE	i-FIT	Norm	Conf(%)	Formula
527.3009	527.3009	0.0	0.0	10.5	147.2	n/a	n/a	C31 H43 O7

b

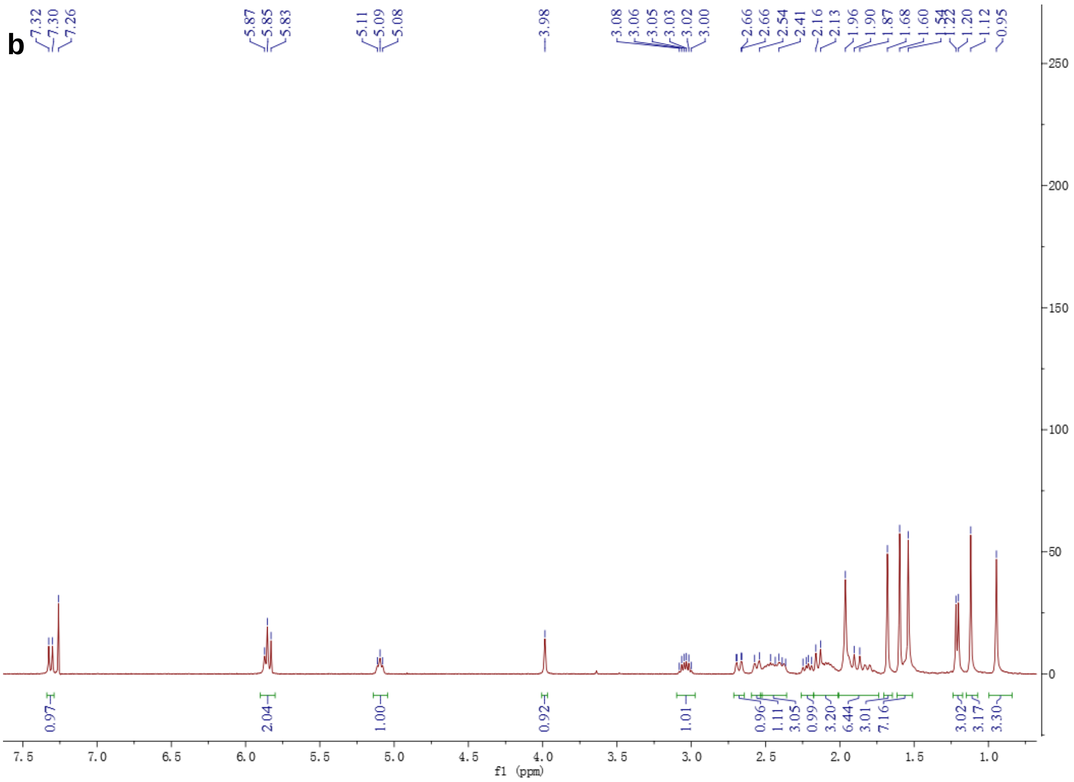

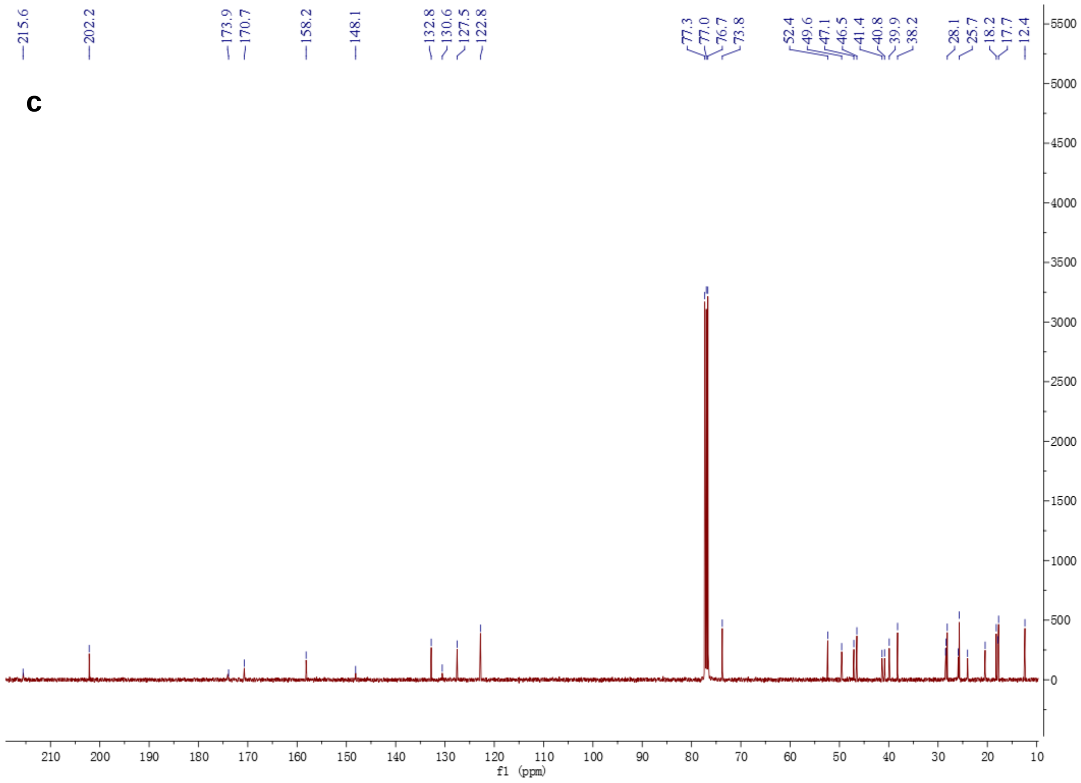

Supplementary Figure 49. HRESIMS and NMR spectra of 22

a HRESIMS spectrum; **b** ^{1}H NMR spectrum in $CDCl_3$ at 400 MHz; **c** ^{13}C NMR spectrum in $CDCl_3$ at 100 MHz.

Supplementary Tables

Supplementary Table 1. NMR assignments for 4 (^{1}H for 400 MHz and ^{13}C for 100 MHz in $CDCl_3$)

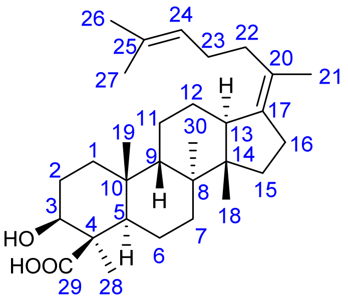

4

No.	δ_C, type	δ_H (J in Hz)[a]	^{1}H-^{1}H COSY	HMBC	NOESY
1	32.6, CH_2	1.47	2a, 2b		
2	29.8, CH_2	a: 1.94	1, 2b, 3		19
		b: 1.90	1, 2a, 3		
3	78.3, CH	3.17, dd (11.4, 4.8)	2a, 2b	29	5, 28
4	48.3, C				
5	50.1, CH	1.52	6a, 6b	6, 29	3, 30
6	20.5, CH_2	a: 1.84	5, 6b, 7a, 7b	4, 5, 7, 10	
		b: 1.52	5, 6a, 7a, 7b		
7	35.3, CH_2	a: 1.99	6a, 6b, 7b	5	18
		b: 1.16	6a, 6b, 7a	30	
8	38.8, C				
9	44.2, CH	1.58	11a, 11b	8, 10, 30	18, 19
10	37.1, C				
11	23.0, CH_2	a: 1.45	9, 11b, 12a, 12b	8	
		b: 1.29	9, 11a, 12a, 12b	12	13
12	27.1, CH_2	a: 2.22	11a, 11b, 12b, 13	9, 14	
		b: 1.35	11a, 11b, 12a, 13	11	18
13	46.7, CH	2.30, br d (11.6)	12a, 12b	12, 17, 18, 20	11b, 30
14	50.5, C				
15	30.3, CH_2	a: 1.45	15b, 16a, 16b	16, 18	
		b: 1.14	15a, 16a, 16b	17, 18	
16	29.2, CH_2	a: 2.15	15a, 15b, 16b	15, 17, 20	
		b: 2.09	15a, 15b, 16a	14, 15, 17, 20	21
17	136.6, C				
18	16.8, CH_3	0.76, s		8, 13, 14, 15	7a, 9, 12b
19	20.0, CH_3	0.91, s		1, 5, 9, 10	2a, 9
20	126.5, C				
21	20.8, CH_3	1.57, s		17, 20, 22	16b
22	33.7, CH_2	2.06	23a, 23b	21, 23, 24	
23	28.0, CH_2	a: 2.07	22, 23b, 24	22	
		b: 1.97	22, 23a, 24	22	
24	124.7, CH	5.10	23a, 23b, 26, 27	22, 23, 26, 27	26
25	131.1, C				
26	25.7, CH_3	1.67, br s	24	24, 25, 27	24
27	17.6, CH_3	1.59, br s	24	24, 25, 26	
28	24.2, CH_3	1.44, s		3, 4, 5, 29	3
29	183.9, C				
30	22.2, CH_3	1.11, s		7, 8, 9, 14	5, 13

[a] The indiscernible signals from overlap or the complex multiplicity are reported without designating multiplicity.

Supplementary Table 2. NMR assignments for 5 (^{1}H for 400 MHz and ^{13}C for 100 MHz in $CDCl_3$)

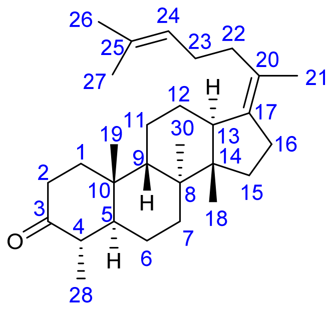

5

No.	δ_C, type	δ_H (J in Hz)[a]	^{1}H-^{1}H COSY	HMBC	ROESY
1	32.7, CH_2	a: 1.85	1b, 2	2, 5, 9, 10, 19	
		b: 1.64	1a, 2	5, 10, 19	
2	37.5, CH_2	2.41	1a, 1b	1, 3	19
3	215.0, C				
4	45.0, CH	2.27	5, 28		19
5	45.5, CH	1.74, br t (12.0)	4, 6a, 6b	4, 6, 7, 10	30
6	21.2, CH_2	a: 1.55	5, 6b, 7a, 7b	8	
		b: 1.21	5, 6a, 7a, 7b	5, 7, 10	
7	33.2, CH_2	a: 1.94	6a, 6b, 7b	5, 8, 14	18
		b: 1.22	6a, 6b, 7a	5, 6	
8	38.9, C				
9	43.8, CH	1.62	11a, 11b	8, 10, 11, 19, 30	18, 19
10	35.8, C				
11	23.4, CH_2	a: 1.57	9, 11b, 12a, 12b	8, 9, 13	
		b: 1.37	9, 11a, 12a, 12b	8, 9, 12, 13	13
12	27.1, CH_2	a: 2.24	11a, 11b, 12b, 13	9, 14	
		b: 1.41	11a, 11b, 12a, 13	11, 13	
13	46.7, CH	2.31	12a, 12b	12, 17, 18, 20	11b, 30
14	50.3, C				
15	30.4, CH_2	a: 1.49	15b, 16a, 16b	14, 16, 18	
		b: 1.14, br dd (11.2, 7.6)	15a, 16a, 16b	13, 17, 18	
16	29.2, CH_2	a: 2.17	15a, 15b, 16b	14, 17, 20	
		b: 2.12	15a, 15b, 16a	14, 17, 20	21
17	136.5, C				
18	16.9, CH_3	0.78, s		8, 13, 14, 15	7a, 9
19	20.9, CH_3	1.00, s		1, 5, 9, 10	2, 4, 9
20	126.6, C				
21	20.8, CH_3	1.58, s		17, 20, 22	16b
22	33.7, CH_2	2.07	23a, 23b	17, 20, 23, 24	
23	28.0, CH_2	a: 2.09	22, 23b, 24	22, 24	
		b: 1.97	22, 23a, 24	22, 24, 25	
24	124.6, CH	5.11	23a, 23b, 26, 27	23, 26, 27	26
25	131.1, C				
26	25.7, CH_3	1.68, br s	24	24, 25, 27	24
27	17.6, CH_3	1.60, br s	24	24, 25, 26	
28	13.0, CH_3	1.02, d (6.8)	4	3, 4, 5	
30	21.4, CH_3	1.10, s		7, 8, 9, 14	5, 13

[a] The indiscernible signals from overlap or the complex multiplicity are reported without designating multiplicity.

Supplementary Table 3. NMR assignments for 6 (^{1}H for 400 MHz and ^{13}C for 100 MHz in $CDCl_3$)

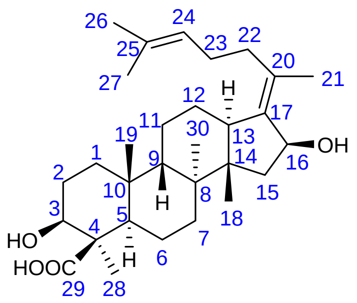

6

No.	δ_C, type	δ_H (J in Hz)[a]	^{1}H-^{1}H COSY	HMBC	δ_H (J in Hz)[a, b]	NOESY[b]
1	32.6, CH_2	1.45	2a, 2b	10, 19	a: 1.52	3
					b: 1.47	
2	29.8, CH_2	a: 1.93	1, 2b, 3		a: 2.31	19
		b: 1.85	1, 2a, 3		b: 2.07	
3	78.3, CH	3.16, dd (11.2, 4.4)	2a, 2b		3.44, dd (11.4, 4.0)	1a, 5, 28
4	48.3, C					
5	50.0, CH	1.51	6a, 6b	3, 4, 6, 29	1.66	3, 30
6	20.5, CH_2	a: 1.85	5, 6b, 7a, 7b		a: 2.10	
		b: 1.51	5, 6a, 7a, 7b		b: 1.90	
7	35.0, CH_2	a: 2.00	6a, 6b, 7b	5, 30	a: 2.16	18
		b: 1.15	6a, 6b, 7a	30	b: 1.22	
8	38.7, C					
9	43.6, CH	1.65	11a, 11b	8, 10, 14	1.80, br d (12.0)	18, 19
10	37.0, C					
11	23.0, CH_2	a: 1.47	9, 11b, 12a, 12b	8, 9, 13	a: 1.51	
		b: 1.25	9, 11a, 12a, 12b	9	b: 1.31	
12	26.8, CH_2	a: 2.20	11a, 11b, 12b, 13	9, 13, 14	a: 2.33	
		b: 1.48	11a, 11b, 12a, 13	9, 13	b: 1.65	
13	48.0, CH	2.33, br d (11.2)	12a, 12b	17	2.49, br d (12.0)	15a, 16, 30
14	49.5, C					
15	41.1, CH_2	a: 1.80	15b, 16	14, 17, 18	a: 1.94	13, 30
		b: 1.35, br d (13.2)	15a, 16	13, 14, 17, 18	b: 1.62	
16	73.1, CH	4.60, br d (8.0)	15a, 15b, 21	14, 17, 20	4.89, br d (6.8)	13, 21
17	140.8, C					
18	19.2, CH_3	1.05, s		8, 13, 14, 15	1.39, s	7a, 9
19	20.1, CH_3	0.90, s		1, 5, 9, 10	1.22, s	2a, 9
20	134.0, C					
21	21.6, CH_3	1.83, br s	16	17, 20, 22	2.14, br s	16
22	33.9, CH_2	2.06	23a, 23b	23	2.29	
23	27.5, CH_2	a: 2.04	22, 23b, 24	22	a: 2.29	
		b: 1.99	22, 23a, 24	22	b: 2.20	
24	124.1, CH	5.09	23a, 23b, 26, 27	22, 23, 26, 27	5.31	26
25	131.6, C					
26	25.7, CH_3	1.68, br s	24	24, 25, 27	1.72, br s	24
27	17.7, CH_3	1.60, br s	24	24, 25, 26	1.66, br s	
28	24.3, CH_3	1.43, s		3, 4, 5, 29	1.71, s	3
29	183.3, C					
30	22.2, CH_3	1.09, s		7, 8, 9, 14	1.17, s	5, 13, 15a

[a] The indiscernible signals from overlap or the complex multiplicity are reported without designating multiplicity.

[b] The data were measured in pyridine-d_5 at 400 MHz.

Supplementary Table 4. NMR assignments for 7 (^{1}H for 400 MHz and ^{13}C for 100 MHzin $CDCl_3$)

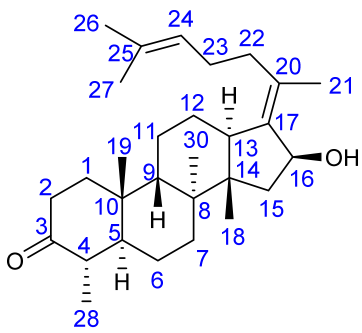

7

No.	δ_C, type	δ_H (J in Hz)[a]	^{1}H-^{1}H COSY	HMBC	δ_H (J in Hz)[a, b]	ROESY[b]
1	32.6, CH_2	a: 1.85	1b, 2	2, 3, 10, 19	a: 1.73	
		b: 1.64	1a, 2	2, 3, 5, 10	b: 1.50	
2	37.4, CH_2	2.41	1a, 1b	1, 3	2.47	19
3	214.9, C					
4	45.0, CH	2.27	5, 28	3, 5, 28	2.26	19
5	45.4, CH	1.73, br t (12.0)	4, 6a, 6b	19	1.74	30
6	21.2, CH_2	a: 1.57	5, 6b, 7a, 7b	7, 8, 10	a: 1.47	28
		b: 1.21	5, 6a, 7a, 7b	7, 8, 10	b: 1.15	
7	33.0, CH_2	a: 1.95	6a, 6b, 7b	8, 9, 14, 30	a: 1.98	18
		b: 1.22	6a, 6b, 7a	8, 9, 30	b: 1.13	
8	38.7, C					
9	43.3, CH	1.68	11a, 11b	7, 8, 10, 11	1.72	18, 19
10	35.8, C					
11	23.4, CH_2	a: 1.58	9, 11b, 12a, 12b	8, 9, 10, 12, 13	a: 1.53	
		b: 1.31	9, 11a, 12a, 12b	9, 12, 13	b: 1.28, qd (12.6, 4.8)	13
12	26.8, CH_2	a: 2.24	11a, 11b, 12b, 13	9, 14	a: 2.34	
		b: 1.55	11a, 11b, 12a, 13	9	b: 1.67	18
13	48.0, CH	2.37	12a, 12b	12, 17	2.46	11b, 15a, 16, 30
14	49.4, C					
15	41.3, CH_2	a: 1.84	15b, 16	13, 14, 17, 18	a: 1.90, dd (12.6, 7.2)	13, 30
		b: 1.35, br d (13.4)	15a, 16	13, 16, 17, 18	b: 1.59, br d (13.2)	
16	73.1, CH	4.61, br d (7.2)	15a, 15b, 21	14, 17, 20	4.89, br d (7.2)	13, 21
17	140.8, C					
18	19.3, CH_3	1.08, s		8, 13, 14, 15	1.37, s	7a, 9, 12b
19	20.8, CH_3	1.01, s		1, 5, 9, 10	0.98, s	2, 4, 9
20	134.1, C					
21	21.6, CH_3	1.85, d (2.0)	16	17, 20, 22	2.15, d (2.4)	16
22	33.9, CH_2	2.11	23a, 23b	17, 20, 23	2.30	
23	27.5, CH_2	a: 2.09	22, 23b, 24		a: 2.28	
		b: 2.02	22, 23a, 24		b: 2.21	
24	124.1, CH	5.11	23a, 23b, 26, 27	23	5.32	26
25	131.6, C					
26	25.8, CH_3	1.69, br s	24	24, 25, 27	1.73, br s	24
27	17.7, CH_3	1.61, br s	24	24, 25, 26	1.68, br s	
28	13.0, CH_3	1.02, d (5.7)	4	3, 4, 5	1.09, d (6.6)	6a
30	21.5, CH_3	1.08, s		7, 8, 9, 14	1.04, s	5, 13, 15a

[a] The indiscernible signals from overlap or the complex multiplicity are reported without designating multiplicity.

[b] The data were measured in pyridine-d_5 at 600 MHz.

Supplementary Table 5. NMR assignments for 8 (^{1}H for 400 MHz and ^{13}C for 100 MHz in $CDCl_3$)

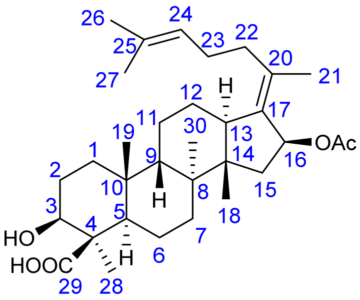

8

No.	δ_C, type	δ_H (J in Hz)[a]	^{1}H-^{1}H COSY	HMBC	ROESY
1	32.6, CH_2	1.45	2a, 2b	2	3
2	29.7, CH_2	a: 1.94	1, 2b, 3		19
		b: 1.85	1, 2a, 3	10	
3	78.3, CH	3.15, dd (12.4, 4.8)	2a, 2b	29	1, 5, 28
4	48.3, C				
5	49.9, CH	1.51	6a, 6b	3, 6, 29	3, 30
6	20.4, CH_2	a: 1.84	5, 6b, 7a, 7b	8	28
		b: 1.49	5, 6a, 7a, 7b		19
7	34.9, CH_2	a: 1.96	6a, 6b, 7b	5, 14, 30	18
		b: 1.13	6a, 6b, 7a	5, 14, 30	
8	38.6, C				
9	43.7, CH	1.62	11a, 11b	8, 10, 14	18, 19
10	37.0, C				
11	23.0, CH_2	a: 1.48	9, 11b, 12a, 12b	8, 9, 13	
		b: 1.24	9, 11a, 12a, 12b	9	
12	26.8, CH_2	a: 2.24	11a, 11b, 12b, 13	9, 14	
		b: 1.48	11a, 11b, 12a, 13	9, 13	18
13	47.3, CH	2.35, br d (10.0)	12a, 12b	17,	15a, 16, 30
14	49.5, C				
15	39.3, CH_2	a: 1.89	15b, 16	14, 18	13, 30
		b: 1.27	15a, 16	13, 14, 16, 17, 18	
16	75.7, CH	5.50, br d (7.2)	15a, 15b, 21	14, 17, 20, 16-<u>C</u>OCH$_3$	13, 21
17	135.3, C				
18	18.1, CH_3	0.96, s		8, 13, 14, 15	7a, 9, 12b
19	20.1, CH_3	0.91, s		1, 5, 9, 10	2a, 6b, 9
20	135.4, C				
21	21.4, CH_3	1.59, br s	16	17, 20, 22	16
22	34.0, CH_2	a: 2.14	22b, 23	23	24
		b: 2.05	22a, 23	23	
23	27.4, CH_2	2.05	22a, 22b, 24	22	
24	124.1, CH	5.09, br t (5.6)	23, 26, 27	22, 23, 26, 27	22a, 26
25	131.6, C				
26	25.7, CH_3	1.68, br s	24	24, 25, 27	24
27	17.7, CH_3	1.59, br s	24	24, 25, 26	
28	24.3, CH_3	1.43, s		3, 4, 5, 29,	3, 6a
29	183.6, C				
30	22.2, CH_3	1.09, s		7, 8, 9, 14	5, 13, 15a
16-<u>C</u>OCH$_3$	171.1, C				
16-CO<u>C</u>H$_3$	21.2, CH_3	2.02, s		16-<u>C</u>OCH$_3$	

[a] The indiscernible signals from overlap or the complex multiplicity are reported without designating multiplicity.

Supplementary Table 6. NMR assignments for 9 (^{1}H for 600 MHz and ^{13}C for 150 MHz in $CDCl_3$)

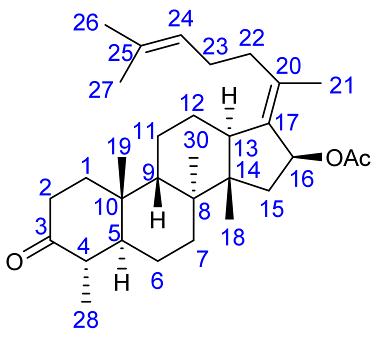

9

No.	δ_C, type	δ_H (J in Hz)[a]	^{1}H-^{1}H COSY	HMBC	ROESY
1	32.5, CH_2	a: 1.84	1b, 2	2, 3, 5, 9, 10, 19	
		b: 1.63	1a, 2	2, 3, 5, 10, 19	
2	37.4, CH_2	2.41	1a, 1b	1, 3, 4, 10	19
3	214.9, C				
4	45.0, CH	2.26	5, 28	3, 5, 6, 10, 28	19
5	45.3, CH	1.74, br t (12.0)	4, 6a, 6b	3, 4, 6, 7, 10, 19, 28	30
6	21.2, CH_2	a: 1.55	5, 6b, 7a, 7b	5, 7, 8, 10	28
		b: 1.20	5, 6a, 7a, 7b	5, 7, 10	19
7	32.8, CH_2	a: 1.90	6a, 6b, 7b	5, 6, 8	18
		b: 1.18	6a, 6b, 7a	8, 9	
8	38.6, C				
9	43.3, CH	1.69	11a, 11b	7, 8, 10, 11, 12	18, 19
10	35.7, C				
11	23.4, CH_2	a: 1.60	9, 11b, 12a, 12b	8, 10, 13	19
		b: 1.33, qd (12.6, 4.2)	9, 11a, 12a, 12b	8, 9, 12	13
12	26.7, CH_2	a: 2.26	11a, 11b, 12b, 13	11, 14	
		b: 1.55	11a, 11b, 12a, 13	13	18
13	47.3, CH	2.38	12a, 12b	12, 14, 17, 18, 20	11b, 15a, 16, 30
14	49.4, C				
15	39.4, CH_2	a: 1.93	15b, 16	14, 18	13, 30
		b: 1.29, br d (13.8)	15a, 16	13, 14, 16, 17, 18	
16	75.6, CH	5.52, br d (7.2)	15a, 15b, 21	13, 14, 17, 20, 16-$\underline{C}OCH_3$	13, 21
17	135.4, C				
18	18.3, CH_3	0.98, s		8, 13, 14, 15	7a, 9, 12b
19	20.9, CH_3	1.01, s		1, 5, 9, 10	2, 4, 6b, 9, 11a
20	135.4, C				
21	21.4, CH_3	1.61, br s	16	17, 20, 22	16
22	34.0, CH_2	a: 2.17	22b, 23	17, 20, 21, 23, 24	24
		b: 2.08	22a, 23	17, 20, 21, 23, 24	
23	27.4, CH_2	2.07	22a, 22b, 24	20, 22, 24, 25	
24	124.1, CH	5.11, br t (6.0)	23, 26, 27	23, 26, 27	22a, 26
25	131.7, C				
26	25.8, CH_3	1.69, br s	24	24, 25, 27	24
27	17.7, CH_3	1.61, br s	24	24, 25, 26	
28	13.0, CH_3	1.02, d (7.2)	4	3, 4, 5	6a
30	21.4, CH_3	1.08, s		7, 8, 9, 14	5, 13. 15a
16-$\underline{C}OCH_3$	171.0, C				
16-$CO\underline{C}H_3$	21.1, CH_3	2.03, s		16, 16-$\underline{C}OCH_3$	

[a] The indiscernible signals from overlap or the complex multiplicity are reported without designating multiplicity.

Supplementary Table 7. NMR assignments for 10 (^{1}H for 400 MHz and ^{13}C for 100 MHz in pyridine-d_5)

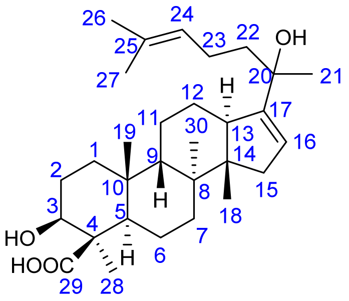

10

No.	δ_C, type	δ_H (J in Hz)[a]	^{1}H-^{1}H COSY	HMBC	NOESY
1	33.1, CH_2	1.47	2a, 2b	3, 5	3
2	31.0, CH_2	a: 2.34	1, 2b, 3		19
		b: 2.08	1, 2a, 3		
3	78.7, CH	3.44, dd (11.4, 3.2)	2a, 2b	29	1, 5, 28
4	48.7, C				
5	50.3, CH	1.70	6a, 6b	7, 10	3, 30
6	21.2, CH_2	a: 2.14	5, 6b, 7a, 7b	8, 10	28
		b: 1.91	5, 6a, 7a, 7b		19
7	35.2, CH_2	a: 2.02	6a, 6b, 7b	5, 8, 14	18
		b: 1.25	6a, 6b, 7a	8, 9	
8	39.1, C				
9	44.7, CH	1.80, br d (11.6)	11a, 11b	8, 10, 19, 30	18, 19
10	37.5, C				
11	23.7, CH_2	a: 1.52	9, 11b, 12a, 12b		
		b: 1.33	9, 11a, 12a, 12b		13
12	25.6, CH_2	a: 2.20	11a, 11b, 12b, 13	9, 14	
		b: 1.62	11a, 11b, 12a, 13		18
13	48.1, CH	2.96, br d (11.6)	12a, 12b, 15a, 16	17	11b, 15a, 30
14	54.0, C				
15	38.3, CH_2	a: 2.39	13, 15b, 16		13, 30
		b: 1.76	15a, 16	16, 17	
16	121.7, CH	5.81, br s	13, 15a, 15b	13, 14, 15, 20	21
17	152.9, C				
18	18.6, CH_3	1.12, s		8, 13, 14, 15	7a, 9, 12b
19	20.4, CH_3	1.22, s		1, 5, 9, 10	2a, 6b, 9
20	72.9, C				
21	28.0, CH_3	1.56, s		17, 20, 22	16
22	41.6, CH_2	1.89, t (8.8)	23a, 23b	17, 20, 21, 23, 24	24
23	23.7, CH_2	a: 2.39	22, 23b, 24	24, 25	
		b: 2.33	22, 23a, 24	22, 24, 25	
24	125.8, CH	5.36, br t (6.4)	23a, 23b, 26, 27	23, 26, 27	22, 26
25	130.8, C				
26	25.8, CH_3	1.71, br s	24	24, 25, 27	24
27	17.7, CH_3	1.66, br s	24	24, 25, 26	
28	25.0, CH_3	1.73, s		3, 4, 5, 29	3, 6a
29	182.2[b], C				
30	24.5, CH_3	1.22, s		7, 8, 9, 14	5, 13, 15a

[a] The indiscernible signals from overlap or the complex multiplicity are reported without designating multiplicity.

[b] The data is obtained from HMBC.

Supplementary Table 8. NMR assignments for 11 (^{1}H for 400 MHz and ^{13}C for 100 MHz in pyridine-d_5)

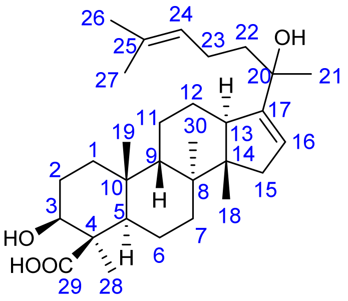

11

No.	δ_C, type	δ_H (J in Hz)[a]	^{1}H-^{1}H COSY	HMBC	NOESY
1	33.1, CH_2	1.46	2a, 2b	2, 3, 5, 10	3
2	30.9, CH_2	a: 2.32	1, 2b, 3		19
		b: 2.06	1, 2a, 3		
3	78.6, CH	3.44, dd (11.6, 4.4)	2a, 2b	28, 29	1, 5, 28
4	48.7, C				
5	50.3, CH	1.70	6a, 6b	1, 7, 10, 19, 28	3, 30
6	21.2, CH_2	a: 2.14	5, 6b, 7a, 7b	4, 5, 7, 8, 10	28
		b: 1.90	5, 6a, 7a, 7b	5, 7, 10	
7	35.2, CH_2	a: 2.02	6a, 6b, 7b	5, 6, 8, 14, 30	18
		b: 1.23	6a, 6b, 7a	5, 6, 8, 9, 14, 30	
8	39.2, C				
9	44.6, CH	1.80	11a, 11b	1, 8, 10, 14, 19, 30	18
10	37.5, C				
11	23.7, CH_2	a: 1.52	9, 11b, 12a, 12b	8, 10, 13	
		b: 1.32	9, 11a, 12a, 12b		
12	25.5, CH_2	a: 2.20	11a, 11b, 12b, 13	9, 14	
		b: 1.62	11a, 11b, 12a, 13		
13	48.0, CH	2.93, br d (12.0)	12a, 12b, 15a, 16	12, 14, 16, 17, 18	15a, 30
14	54.0, C				
15	38.3, CH_2	a: 2.39	13, 15b, 16	16, 17, 18	13, 30
		b: 1.76	15a, 16	14, 16, 17	
16	121.8, CH	5.81, br s	13, 15a, 15b	13, 14, 15, 20	21
17	152.9, C				
18	18.7, CH_3	1.15, s		8, 13, 14, 15	7a, 9
19	20.4, CH_3	1.20, s		1, 5, 9, 10	2a
20	72.9, C				
21	28.1, CH_3	1.56, s		17, 20, 22	16
22	41.8, CH_2	1.89, t (8.8)	23a, 23b	17, 20, 21, 23, 24	24
23	23.7, CH_2	a: 2.42	22, 23b, 24	20, 22, 24, 25	
		b: 2.35	22, 23a, 24	20, 22, 24, 25	
24	125.9, CH	5.36, br t (6.4)	23a, 23b, 26, 27	23, 26, 27	22, 26
25	130.8, C				
26	25.8, CH_3	1.70, br s	24	24, 25, 27	24
27	17.7, CH_3	1.66, br s	24	24, 25, 26	
28	25.0, CH_3	1.73, s		3, 4, 5, 29	3, 6a
29	182.2, C				
30	24.5, CH_3	1.23, s		7, 8, 9, 14	5, 13, 15a

[a] The indiscernible signals from overlap or the complex multiplicity are reported without designating multiplicity.

Supplementary Table 9. NMR assignments for 12 (^{1}H for 600 MHz and ^{13}C for 150 MHz in pyridine-d_5)

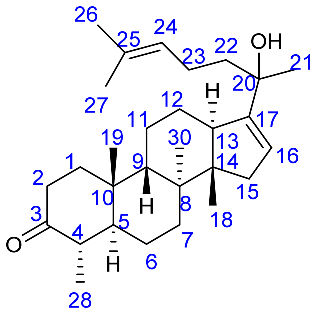

12

No.	δ_C, type	δ_H (J in Hz)[a]	^{1}H-^{1}H COSY	HMBC	ROESY
1	32.8, CH_2	a: 1.70	1b, 2a, 2b	3, 10, 19	
		b: 1.49	1a, 2a, 2b	3	
2	37.7, CH_2	a: 2.50, ddd (16.2, 7.8, 2.4)	1a, 1b, 2b	1, 3, 4, 10	
		b: 2.44	1a, 1b, 2a	1, 3, 10	19
3	213.2, C				
4	45.1, CH	2.27, dq (12.0, 5.4)	5, 28	3, 5, 6, 10, 28	19
5	45.7, CH	1.76, br t (12.0)	4, 6a, 6b	4, 6, 7, 10, 28	30
6	21.3, CH_2	a: 1.51	5, 6b, 7a, 7b	5, 7, 8, 10	28
		b: 1.17	5, 6a, 7a, 7b	5, 7, 10	19
7	33.1, CH_2	a: 1.88	6a, 6b, 7b	5, 6, 8, 14	9, 18
		b: 1.15	6a, 6b, 7a	6, 8, 9, 30	
8	39.1, C				
9	44.1, CH	1.71	11a, 11b	7, 8, 10, 11, 14, 19	7a, 18
10	36.1, C				
11	24.0, CH_2	a: 1.56	9, 11b, 12a, 12b		
		b: 1.29	9, 11a, 12a, 12b	8, 9, 10, 12, 13	13, 30
12	25.4, CH_2	a: 2.21, br d (12.0)	11a, 11b, 12b, 13	9, 11, 13, 14	22
		b: 1.70	11a, 11b, 12a, 13		18
13	48.0, CH	2.90, br dd (12.6, 3.0)	12a, 12b, 15a, 16	8, 11, 12, 14, 16, 17, 18, 20	11b, 15a, 21, 30
14	53.7, C				
15	38.4, CH_2	a: 2.36	13, 15b, 16	8, 14, 16, 17, 18	13, 30
		b: 1.73	15a, 16	13, 14, 16, 17	
16	121.9, CH	5.80, br s	13, 15a, 15b	13, 14, 15, 17, 20	21, 22
17	152.7, C				
18	18.6, CH_3	1.12, s		8, 13, 14, 15	7a, 9, 12b
19	20.5, CH_3	0.95, s		1, 5, 9, 10	2b, 4, 6b
20	72.9, C				
21	28.0, CH_3	1.58, s		17, 20, 22	13, 16
22	41.9, CH_2	1.89	23a, 23b	17, 20, 21, 23, 24	12a, 16, 24
23	23.7, CH_2	a: 2.42	22, 23b, 24	20, 22, 24, 25	
		b: 2.37	22, 23a, 24	20, 22, 24, 25	
24	125.9, CH	5.37, br t (6.6)	23a, 23b, 26, 27	22, 23, 26, 27	22, 26
25	130.8, C				
26	25.8, CH_3	1.72, br s	24	24, 25, 27	24
27	17.7, CH_3	1.66, br s	24	24, 25, 26	
28	13.2 CH_3	1.11, d (5.4)	4	3, 4, 5	6a
30	23.6, CH_3	1.09, s		7, 8, 9, 14	5, 11b, 13, 15a
20-OH		5.58, s		17, 20, 21, 22	

[a] The indiscernible signals from overlap or the complex multiplicity are reported without designating multiplicity.

Supplementary Table 10. NMR assignments for 13 (^{1}H for 600 MHz and ^{13}C for 150 MHz in pyridine-d_5)

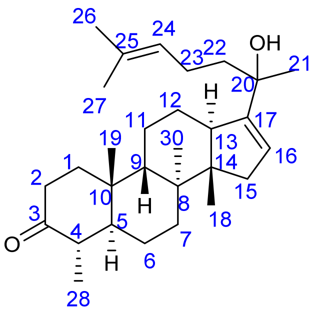

13

No.	δ_C, type	δ_H (J in Hz)[a]	^{1}H-^{1}H COSY	HMBC	ROESY
1	32.8, CH_2	a: 1.69	1b, 2a, 2b	3, 10, 19	
		b: 1.48	1a, 2a, 2b	3	
2	37.7, CH_2	a: 2.50, ddd (15.6, 7.8, 3.0)	1a, 1b, 2b	1, 3, 4, 10	
		b: 2.43	1a, 1b, 2a	1, 3, 10	19
3	213.1, C				
4	45.1, CH	2.27, dq (12.0, 6.6)	5, 28	3, 5, 6, 10, 28	19
5	45.7, CH	1.78, br t (12.0)	4, 6a, 6b	4, 6, 7, 10, 28	30
6	21.3, CH_2	a: 1.51	5, 6b, 7a, 7b	5, 7, 8, 10	28
		b: 1.17	5, 6a, 7a, 7b	5, 7, 10	19
7	33.0, CH_2	a: 1.86	6a, 6b, 7b	5, 6, 8, 14	9, 18
		b: 1.15	6a, 6b, 7a	6, 8, 9, 30	
8	39.0, C				
9	44.2, CH	1.69	11a, 11b	7, 8, 10, 11, 14, 19	7a, 18
10	36.1, C				
11	24.0, CH_2	a: 1.55	9, 11b, 12a, 12b		
		b: 1.29, qd (13.2, 4.2)	9, 11a, 12a, 12b	8, 9, 10, 12, 13	13, 30
12	25.5, CH_2	a: 2.20, br d (12.0)	11a, 11b, 12b, 13	9, 11, 13, 14	22
		b: 1.62, qd (12.6, 4.2)	11a, 11b, 12a, 13		18
13	48.1, CH	2.93, br dd (12.9, 3.0)	12a, 12b, 15a, 16	8, 11, 12, 14, 16, 17, 18, 20	11b, 15a, 30
14	53.7, C				
15	38.3, CH_2	a: 2.36	13, 15b, 16	8, 14, 16, 17, 18	13, 30
		b: 1.72	15a, 16	14, 16, 17	
16	121.8, CH	5.80, br d (1.8)	13, 15a, 15b	13, 14, 15, 17, 20	21, 22
17	152.8, C				
18	18.6, CH_3	1.08, s		8, 13, 14, 15	7a, 9, 12b
19	20.6, CH_3	0.96, s		1, 5, 9, 10	2b, 4, 6b
20	72.9, C				
21	28.0, CH_3	1.56, s		17, 20, 22	16
22	41.6, CH_2	1.88	23a, 23b	17, 20, 21, 23, 24	12a, 16, 24
23	23.7, CH_2	a: 2.40	22, 23b, 24	20, 22, 24, 25	
		b: 2.31	22, 23a, 24	20, 22, 24, 25	
24	125.8, CH	5.37, br t (7.2)	23a, 23b, 26, 27	22, 23, 26, 27	22, 26
25	130.9, C				
26	25.8, CH_3	1.72, br s	24	24, 25, 27	24
27	17.7, CH_3	1.67, br s	24	24, 25, 26	
28	13.2 CH_3	1.11, d (6.6)	4	3,4,5	6a
30	23.5, CH_3	1.08, s		7, 8, 9, 14	5, 11b, 13, 15a
20-OH		5.66, s		17, 20, 21, 22	

[a] The indiscernible signals from overlap or the complex multiplicity are reported without designating multiplicity.

Supplementary Table 11. NMR assignments for 14 (^{1}H for 400 MHz and ^{13}C for 100 MHz in $CDCl_3$)

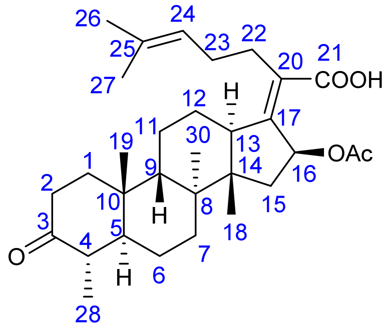

14

No.	δ_C, type	δ_H (J in Hz)[a]	^{1}H-^{1}H COSY	HMBC	δ_H (J in Hz)[a, b]	ROESY[b]
1	32.6, CH_2	a: 1.84	1b, 2	2, 3, 10, 19	a: 1.69	
		b: 1.64	1a, 2	3, 5, 10	b: 1.48	
2	37.3, CH_2	2.41	1a, 1b	1, 3, 10	2.46	19
3	214.8, C					
4	45.0, CH	2.25	5, 28	3, 5, 6, 10, 28	2.25, dq (13.2, 6.6)	19
5	45.4, CH	1.71	4, 6a, 6b	4, 7, 10, 28	1.68	30
6	21.0, CH_2	a: 1.56	5, 6b, 7a, 7b	5, 7, 8, 10	a: 1.47	28
		b: 1.20	5, 6a, 7a, 7b	5, 7, 8, 10	b: 1.13	
7	32.8, CH_2	a: 1.88	6a, 6b, 7b	5, 8, 14, 30	a: 1.85, br dd (13.8, 6.0)	18
		b: 1.19	6a, 6b, 7a	5, 8, 9, 30	b: 1.08	
8	38.5, C					
9	43.3, CH	1.66	11a, 11b	8, 10, 11, 12, 30	1.63, br d (12.6)	18, 19
10	35.7, C					
11	23.0, CH_2	a: 1.64	9, 11b, 12a, 12b	8, 10, 12, 13	a: 1.53	
		b: 1.35	9, 11a, 12a, 12b	8, 9, 10, 12	b: 1.24	13
12	26.1, CH_2	a: 2.27	11a, 11b, 12b, 13	9, 13, 14	a: 2.33, br d (11.4)	
		b: 1.68	11a, 11b, 12a, 13	11, 13, 14	b: 1.69	18
13	48.6, CH	2.53	12a, 12b	11, 12, 14, 15, 17, 18, 20	2.55, br d (12.6)	11b, 15a, 16, 30
14	48.7, C					
15	38.7, CH_2	a: 2.07	15b, 16	8, 14, 18	a: 2.10, dd (13.2, 7.8)	13, 30
		b: 1.28, br d (14.0)	15a, 16	13, 14, 16, 17, 18	b: 1.40, br d (13.2)	
16	74.2, CH	5.83, br d (8.4)	15a, 15b	13, 14, 15, 17, 20, 16-C̲OCH$_3$	6.44, br d (7.8)	13
17	150.5, C					
18	18.0, CH_3	0.95, s		8, 13, 14, 15	1.08, s	7a, 9, 12b
19	21.0, CH_3	1.01, s		1, 5, 9, 10	0.97, s	2, 4, 9
20	129.8, C					
21	174.8, C					
22	28.5, CH_2	a: 2.50	22b, 23a, 23b	17, 20, 21, 23, 24	a: 2.87	24
		b: 2.40	22a, 23a, 23b	17, 20, 21, 23, 24	b: 2.81	24
23	28.2, CH_2	a: 2.11	22a, 22b, 23b, 24	20, 22, 24, 25	a: 2.52	
		b: 2.02	22a, 22b, 23a, 24	20, 22, 24, 25	b: 2.39	
24	122.9, CH	5.08, br t (6.8)	23a, 23b, 26, 27	22, 23, 26, 27	5.37, br t (6.6)	22a, 22b, 26
25	132.6, C					
26	25.6, CH_3	1.66, br s	24	24, 25, 27	1.70, br s	24
27	17.7, CH_3	1.58, br s	24	24, 25, 26	1.67, br s	
28	12.9, CH_3	1.01, d (6.0)	4	3, 4, 5	1.09, d (6.6)	6a
30	21.3, CH_3	1.09, s		7, 8, 9, 14	0.97, s	5, 13, 15a
16-C̲OCH$_3$	170.5, C					
16-COC̲H$_3$	20.5, CH_3	1.94, s		16-C̲OCH$_3$	1.96, s	

[a] The indiscernible signals from overlap or the complex multiplicity are reported without designating multiplicity.

[b] The data were measured in pyridine-d_5 at 600 MHz.

Supplementary Table 12. NMR assignments for 15 (^{1}H for 400 MHz and ^{13}C for 100 MHz in $CDCl_3$)

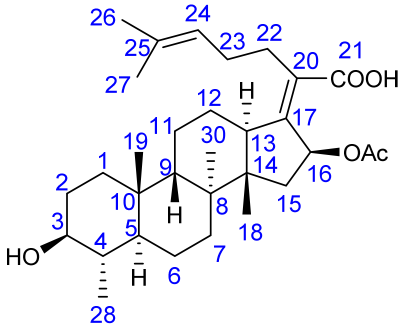

15

No.	δ_C, type	δ_H (J in Hz)[a]	^{1}H-^{1}H COSY	HMBC	NOESY
1	32.3, CH_2	1.43	2a, 2b	2, 10, 19	3
2	31.5, CH_2	a: 1.83	1, 2b, 3		
		b: 1.58	1, 2a, 3	1	19
3	77.3, CH	3.10, td (10.0, 5.6)	2a, 2b, 4	28	1, 5, 28
4	38.1, CH	1.44	3, 5, 28	2, 3, 10	19
5	44.3, CH	1.24	4, 6a, 6b	19	3, 30
6	20.1, CH_2	a: 1.58	5, 6b, 7a, 7b	10	
		b: 1.11	5, 6a, 7a, 7b		
7	33.0, CH_2	a: 1.85	6a, 6b, 7b	5, 6, 8, 9, 14	18
		b: 1.13	6a, 6b, 7a	8, 9	
8	38.6, C				
9	44.0, CH	1.56	11a, 11b	10, 11, 14, 19	18, 19
10	35.8, C				
11	23.3, CH_2	a: 1.58	9, 11b, 12a, 12b		
		b: 1.29	9, 11a, 12a, 12b		
12	26.3, CH_2	a: 2.24	11a, 11b, 12b, 13	13, 14	
		b: 1.63	11a, 11b, 12a, 13	13, 14	18
13	48.8, CH	2.52	12a, 12b	11, 14, 17, 18, 20	15a, 16, 30
14	48.7, C				
15	38.8, CH_2	a: 2.06	15b, 16	13, 14, 18	13
		b: 1.26	15a, 16	13, 14, 16, 17, 18	
16	74.3, CH	5.83, br d (8.4)	15a, 15b	13, 14, 17, 20, 16-<u>C</u>OCH$_3$	13
17	150.6, C				
18	18.1, CH_3	0.92, s		8, 13, 14, 15	7a, 9, 12b
19	21.4, CH_3	0.91, s		1, 5, 9, 10	2b, 4, 9
20	129.6, C				
21	174.5, C				
22	28.5, CH_2	a: 2.48	22b, 23a, 23b	17, 20, 21	24
		b: 2.38	22a, 23a, 23b	17, 20, 21	24
23	28.3, CH_2	a: 2.13	22a, 22b, 23b, 24	22, 24, 25	
		b: 2.06	22a, 22b, 23a, 24	22, 24, 25	
24	123.0, CH	5.09, br t (6.8)	23a, 23b, 26, 27	23, 26, 27	22a, 22b, 26
25	132.6, C				
26	25.7, CH_3	1.67, br s	24	24, 25, 27	24
27	17.7, CH_3	1.58, br s	24	24, 25, 26	
28	15.5, CH_3	0.94, d (6.4)	4	3, 4, 5	3
30	21.4, CH_3	1.08, s		7, 8, 9, 14	5, 13
16-<u>C</u>OCH$_3$	170.6, C				
16-CO<u>C</u>H$_3$	20.6, CH_3	1.95, s		16-<u>C</u>OCH$_3$	

[a] The indiscernible signals from overlap or the complex multiplicity are reported without designating multiplicity.

Supplementary Table 13. NMR assignments for 16 (^{1}H for 600 MHz and ^{13}C for 150 MHz in pyridine-d_5)

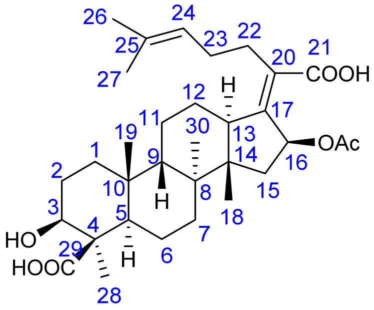

16

No.	δ_C, type	δ_H (J in Hz)[a]	^{1}H-^{1}H COSY	HMBC	ROESY
1	33.2, CH_2	a: 1.51	1b, 2a, 2b	2, 3, 5, 10, 19	3
		b: 1.44	1a, 2a, 2b	2, 3, 10	
2	30.9, CH_2	a: 2.34	1a, 1b, 2b, 3	1, 3, 4	19
		b: 2.09	1a, 1b, 2a, 3	1, 3	
3	78.5, CH	3.45, dd (12.0, 4.8)	2a, 2b	2, 4, 5, 28, 29	1a, 5, 28
4	48.7, C				
5	50.4, CH	1.61, br d (12.0)	6a, 6b	1, 3, 4, 6, 7, 9, 10, 19, 28, 29	3, 30
6	21.2, CH_2	a: 2.13	5, 6b, 7a, 7b	4, 5, 7, 8, 10	28
		b: 1.88	5, 6a, 7a, 7b	5, 7, 10	19
7	35.5, CH_2	a: 2.04, br dd (13.8, 6.0)	6a, 6b, 7b	5, 6, 8, 14, 30	18
		b: 1.20	6a, 6b, 7a	5, 6, 8, 9, 30	
8	38.8, C				
9	44.1, CH	1.72	11a, 11b	1, 8, 10, 11, 19, 30	18, 19
10	37.3, C				
11	23.0, CH_2	a: 1.50	9, 11b, 12a, 12b	10	
		b: 1.27	9, 11a,12a, 12b	8, 9, 12, 13	13
12	26.7, CH_2	a: 2.33	11a, 11b, 12b, 13	9, 11	
		b: 1.69	11a, 11b, 12a, 13	11	18
13	48.5, CH	2.60, br d (11.4)	12a, 12b	12, 14, 17, 18, 20	11b, 15a, 16, 30
14	49.2, C				
15	39.2, CH_2	a: 2.16, dd 13.8, 8.4)	15b, 16	8, 14, 18	13, 30
		b: 1.46, br d (13.8)	15a, 16	12, 13, 14, 16, 17, 18	
16	74.5, CH	6.46, br d (8.4)	15a, 15b	14, 15, 17, 20, 16-<u>C</u>OCH$_3$	13
17	147.0, C				
18	18.1, CH_3	1.13, s		8, 13, 14, 15	7a, 9, 12b
19	20.6, CH_3	1.21, s		1, 5, 9, 10	2a, 6b, 9
20	132.4, C				
21	172.9, C				
22	29.5, CH_2	a: 2.88	22b, 23a, 23b	17, 20, 21, 23, 24	24
		b: 2.82	22a, 23a, 23b	17, 20, 21, 23, 24	24
23	29.0, CH_2	a: 2.53	22a, 22b, 23b, 24	22, 24, 25	
		b: 2.42	22a, 22b, 23a, 24	22, 24, 25	
24	124.4, CH	5.38,br t (6.6)	23a, 23b, 26, 27	23, 26, 27	22a, 22b, 26
25	132.1, C				
26	25.8, CH_3	1.70, br s	24	24, 25, 27	24
27	17.8, CH_3	1.67, br s	24	24, 25, 26	
28	24.9, CH_3	1.71, s		3, 4, 5, 29	3, 6a
29	182.2, C				
30	22.3, CH_3	1.11, s		7, 8, 9, 14	5, 13, 15a
16-<u>C</u>OCH$_3$	170.5, C				
16-CO<u>C</u>H$_3$	20.7, CH_3	1.96, s		16-<u>C</u>OCH$_3$	

[a] The indiscernible signals from overlap or the complex multiplicity are reported without designating multiplicity.

Supplementary Table 14. NMR assignments for 17 (^{1}H for 400 MHz and ^{13}C for 100 MHz in $CDCl_3$)

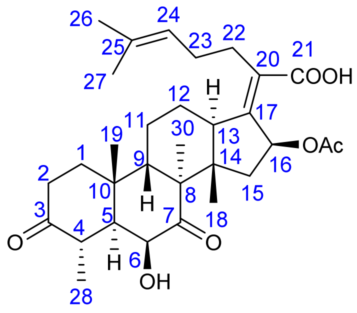

17

No.	δ_C, type	δ_H (J in Hz)[a]	^{1}H-^{1}H COSY	HMBC	NOESY
1	33.6, CH_2	a: 1.84	1b, 2	2, 3, 5, 10, 19	
		b: 1.67	1a, 2	2, 3, 5, 10	
2	37.2, CH_2	2.49	1a, 1b	1, 3	19
3	213.1, C				
4	41.6, CH	2.90, dq (13.0, 6.8)	5, 28	3, 5, 6, 10, 28	19
5	45.8, CH	1.82	4, 6	1, 4, 10, 19, 28	28, 30
6	73.5 CH	3.91, br s	5	4, 5, 7, 8, 10	28
7	216.2, C				
8	52.5, C				
9	40.8, CH	2.63, br d (11.2)	11a, 11b	8, 10, 11, 19, 30	18, 19
10	35.0, C				
11	22.8, CH_2	a: 1.87	9, 11b, 12a, 12b	13	
		b: 1.43	9, 11a, 12a, 12b		13
12	26.0, CH_2	a: 2.34	11a, 11b, 12b, 13	9, 14	
		b: 1.78	11a, 11b, 12a, 13		18
13	49.4, CH	2.54	12a, 12b	12, 14, 17, 18, 20	11b, 16, 30
14	46.7, C				
15	40.6, CH_2	a: 2.28	15b, 16	8, 14, 18	30
		b: 1.84	15a, 16	8, 13, 14, 16, 17, 18	
16	73.9, CH	5.85, br d (8.4)	15a, 15b	13, 14, 17, 20, 21, 22, 16-<u>C</u>OCH$_3$	13, 30
17	149.4, C				
18	18.2, CH_3	0.93, s		8, 13, 14, 15	9, 12b, 16-COC<u>H</u>$_3$
19	23.8, CH_3	1.40, s		1, 5, 9, 10	2, 4, 9
20	130.2, C				
21	174.3, C				
22	28.5, CH_2	2.45	23a, 23b	17, 20, 21, 23, 24	24
23	28.4, CH_2	a: 2.11	22, 23b, 24	20, 22, 24, 25	
		b: 2.03	22, 23a, 24	20, 22, 24, 25	
24	122.8, CH	5.09, br t (6.8)	23a, 23b, 26, 27	22, 26, 27	22, 26
25	132.8, C				
26	25.7, CH_3	1.67, br s	24	24, 25, 27	24
27	17.7, CH_3	1.58, br s	24	24, 25, 26	
28	12.3, CH_3	1.09, d (6.8)	4	3, 4, 5	5, 6
30	17.1, CH_3	1.23, s		7, 8, 9, 14	5, 13, 15a, 16
16-<u>C</u>OCH$_3$	170.6, C				
16-CO<u>C</u>H$_3$	20.3, CH_3	1.94, s		16-<u>C</u>OCH$_3$	18

[a] The indiscernible signals from overlap or the complex multiplicity are reported without designating multiplicity.

Supplementary Table 15. NMR assignments for 18 (^{1}H for 400 MHz and ^{13}C for 100 MHz in pyridine-d_5)

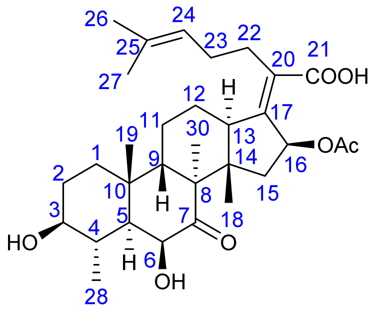

18

No.	δ_C, type	δ_H (J in Hz)[a]	^{1}H-^{1}H COSY	HMBC	ROESY
1	34.2, CH_2	1.57	2a, 2b		3
2	32.8, CH_2	a: 2.19	1, 2b, 3		
		b: 2.11	1, 2a, 3		19
3	76.8, CH	3.45, td (10.1, 5.9)	2a, 2b, 4		1, 5, 28
4	36.1, CH	2.49	3, 5, 28		19
5	46.9, CH	1.59, br d (10.4)	4, 6	3, 4, 7, 19	3, 30
6	74.0, CH	4.47, br s	5	5, 7, 8, 10	28
7	216.3, C				
8	52.9, C				
9	41.9, CH	2.90	11a, 11b	1, 8, 10, 11, 19, 30	18, 19
10	35.9, C				
11	23.4, CH_2	a: 1.81	9, 11b, 12a, 12b	8, 13	
		b: 1.40	9, 11a, 12a, 12b		
12	26.9, CH_2	a: 2.43	11a, 11b, 12b, 13	9, 14	
		b: 1.82	11a, 11b, 12a, 13	13	
13	49.2, CH	2.69, br d (11.6)	12a, 12b	12, 14, 17, 18, 20	16, 30
14	47.3, C				
15	41.7, CH_2	a: 2.59	15b, 16	18	30
		b: 2.41	15a, 16	13, 14, 16, 17, 18	
16	74.5, CH	6.50, br d (8.0)	15a, 15b	14, 17, 20, 16-<u>C</u>OCH$_3$	13
17	145.2, C				
18	18.8, CH_3	1.39, s		8, 13, 14, 15	9
19	24.1, CH_3	1.66, s		1, 5, 9, 10	2b, 4, 9
20	132.8, C				
21	173.1, C				
22	29.5, CH_2	2.89	23a, 23b	20, 21	24
23	29.1, CH_2	a: 2.52	22, 23b	20, 22, 24	
		b: 2.45	22, 23a	22, 24	
24	124.4, CH	5.39, br t (6.8)	23a, 23b, 26, 27	23, 26, 27	22, 26
25	132.1, C				
26	25.8, CH_3	1.71, br s	24	24, 25, 27	24
27	18.0, CH_3	1.68, br s	24	24, 25, 26	
28	15.6, CH_3	1.33, d (6.4)	4	3, 4 ,5	3, 6
30	17.8, CH_3	1.36, s		7, 8, 9, 14	5, 13, 15a
16-<u>C</u>OCH$_3$	170.5, C				
16-CO<u>C</u>H$_3$	20.6, CH_3	1.83, s		16-<u>C</u>OCH$_3$	

[a] The indiscernible signals from overlap or the complex multiplicity are reported without designating multiplicity.

Supplementary Table 16. NMR assignments for 19 (^{1}H for 400 MHz and ^{13}C for 100 MHz in $CDCl_3$)

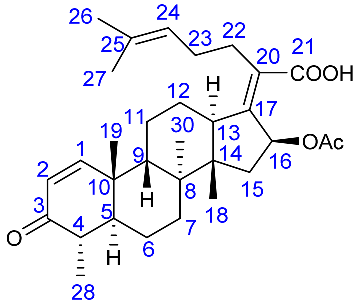

19

No.	δ_C, type	δ_H (J in Hz)[a]	^{1}H-^{1}H COSY	HMBC	ROESY
1	159.4, CH	7.30, d (10.0)	2	3, 5, 9, 10	11a, 11b
2	127.7, CH	5.82, d (10.0)	1	3, 4, 10	
3	202.9, C				
4	42.6, CH	2.40	5, 28		19
5	46.8, CH	2.03	4, 6a, 6b	4, 6, 7, 10, 19	28, 30
6	20.4, CH_2	a: 1.61	5, 6b, 7a, 7b	10	
		b: 1.24	5, 6a, 7a, 7b		
7	33.6, CH_2	a: 1.96	6a, 6b, 7b	5, 8, 30	
		b: 1.23	6a, 6b, 7a	30	9
8	38.3, C				
9	43.8, CH	1.64	11a, 11b	10, 11, 19, 30	7b, 18, 19
10	39.0, C				
11	24.7, CH_2	a: 1.74	9, 11b, 12a, 12b	8, 13	1
		b: 1.45	9, 11a, 12a, 12b		1, 13
12	26.0, CH_2	a: 2.33	11a, 11b, 12b, 13	9, 11, 14	
		b: 1.72	11a, 11b, 12a, 13	13	18
13	49.1, CH	2.56, br d (10.8)	12a, 12b	12, 14, 17, 18, 20	11b, 16, 30
14	48.4, C				
15	39.1, CH_2	a: 2.01	15b, 16	8, 14, 18	30
		b: 1.31, br d (14.0)	15a, 16	13, 16, 17, 18	
16	74.1, CH	5.86, br d (8.4)	15a, 15b	14, 15, 17, 20, 16-<u>C</u>OCH_3	13
17	150.1, C				
18	18.4, CH_3	0.97, s		8, 13, 14, 15	9, 12b
19	25.7, CH_3	1.15, s		1, 5, 9, 10	4, 9
20	130.0, C				
21	174.6, C				
22	28.6, CH_2	2.45	23a, 23b	17, 20, 21, 23	24
23	28.3, CH_2	a: 2.15	22, 23b, 24	22, 24, 25	
		b: 2.05	22, 23a, 24	22, 24, 25	
24	122.9, CH	5.10, br t (7.2)	23a, 23b, 26, 27	26, 27	22, 26
25	132.7, C				
26	25.4, CH_3	1.68, br s	24	24, 25, 27	24
27	17.7, CH_3	1.59, br s	24	24, 25, 26	
28	13.0 CH_3	1.13, d (6.8)	4	3, 4, 5	5
30	21.7, CH_3	0.99, s		7, 8, 9, 14	5, 13, 15a
16-<u>C</u>OCH_3	170.6, C				
16-CO<u>C</u>H_3	20.5, CH_3	1.95, s		16-<u>C</u>OCH_3	

[a] The indiscernible signals from overlap or the complex multiplicity are reported without designating multiplicity.

Supplementary Table 17. NMR assignments for 21 (^{1}H for 600 MHz and ^{13}C for 150 MHz in $CDCl_3$)

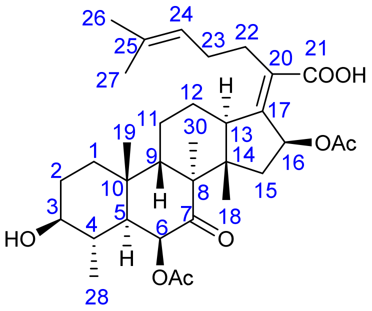

21

No.	δ_C, type	δ_H (J in Hz)[a]	^{1}H-^{1}H COSY	HMBC	ROESY
1	33.5, CH_2	1.51	2a, 2b	2, 3, 9, 10, 19	3
2	31.0, CH_2	a: 1.92	1, 2b, 3	3, 4	
		b: 1.66	1, 2a, 3	3, 4	19
3	77.0, CH	3.13, td (10.1, 5.4)	2a, 2b, 4	28	1, 5, 28
4	35.3, CH	1.82	3, 5, 28	3, 6, 10, 28	19
5	46.0, CH	1.47, br d (10.8)	4, 6	1, 3, 4, 7, 10, 19, 28	3, 28, 30
6	73.7, CH	5.27, d (1.2)	5	4, 5, 7, 8, 6-<u>C</u>OCH$_3$	28
7	210.0, C				
8	52.7, C				
9	41.8, CH	2.45	11a, 11b	1, 7, 8, 10, 12, 14, 19, 30	12b, 18, 19
10	35.4, C				
11	22.9, CH_2	a: 1.80	9, 11b, 12a, 12b	10, 12, 13	
		b: 1.40	9, 11a, 12a, 12b	8, 9, 12, 13	13
12	26.1, CH_2	a: 2.32, br dd (12.6, 3.0)	11a, 11b, 12b, 13	9, 11, 13, 14	
		b: 1.72	11a, 11b, 12a, 13	11, 13	9, 18
13	49.1, CH	2.52	12a, 12b	12, 14, 17, 18, 20	11b, 15a, 16, 30
14	46.9, C				
15	40.4, CH_2	a: 2.24, dd (15.0, 8.4)	15b, 16	8, 14, 18	13, 30
		b: 1.84, br d (15.0)	15a, 16	13, 14, 17	
16	73.6, CH	5.88, br d (8.4)	15a, 15b	14, 17, 20, 16-<u>C</u>OCH$_3$	13
17	147.5, C				
18	18.0, CH_3	0.89, s		8, 13, 14, 15	9, 12b, 6-COC<u>H</u>$_3$
19	23.7, CH_3	1.25, s		1, 5, 9, 10	2b, 4, 9, 6-COC<u>H</u>$_3$
20	130.5, C				
21	173.9, C				
22	28.6, CH_2	a: 2.49	22b, 23a, 23b	17, 20, 21, 23, 24	24
		b: 2.42	22a, 23a, 23b	17, 20, 21, 23, 24	24
23	28.3, CH_2	a: 2.11	22a, 22b, 23b, 24	22, 24, 25	
		b: 2.05	22a, 22b, 23a, 24	22, 24, 25	
24	122.9, CH	5.09, br t (7.2)	23a, 23b, 26, 27	23, 26, 27	22a, 22b, 26
25	132.7, C				
26	25.7, CH_3	1.68, br s	24	23, 24, 25, 27	24
27	17.7, CH_3	1.59, br s	24	23, 24, 25, 26	
28	15.2, CH_3	1.08, d (6.0)	4	3, 4, 5	3, 5, 6
30	18.1, CH_3	1.28, s		7, 8, 9, 14	5, 13, 15a
6-<u>C</u>OCH$_3$	169.0, C				
6-CO<u>C</u>H$_3$	20.8, CH_3	2.06, s		6-<u>C</u>OCH$_3$	18, 19
16-<u>C</u>OCH$_3$	170.5, C				
16-CO<u>C</u>H$_3$	20.6, CH_3	1.93, s		16-<u>C</u>OCH$_3$	

[a] The indiscernible signals from overlap or the complex multiplicity are reported without designating multiplicity.

Supplementary Table 18. Primers used for constructing recombinant plasmids

Primer	Sequence (5' to 3')	Usage
HelA-F	TCGAGCTCGGTACCCATGGCGACAGACAGCAGCAT	Cloning of *helA* from *A. fumigatus* Af293 genome
HelA-R	CTACTACAGATCCCCGGTGTCACTCAGTACCAGAC	
HelB1-KpnI-F	CATTAA**GGTACC**ATGATTAGAGTCGCTGAG	Cloning of *helB1* from *A. fumigatus* Af293 genome
HelB1-KpnI-R	CATTAA**GGTACC**CTAGCAAGGTGAATACTCT	
HelB2-EcoRI-F	AAC**GAATTC**ATGGCCCTCCCCATTATTCT	Cloning of *helB2* from *A. fumigatus* Af293 genome
HelB2-KpnI-R	CATTAA**GGTACC**CTAGGTCTTTGGCCGGCTTAT	
HelB3-EcoRI-F	AAC**GAATTC**ATGGCCGTCGCCACCCTGAT	Cloning of *helB3* from *A. fumigatus* Af293 genome
HelB3-KpnI-R	CATTAA**GGTACC**TCACTCTGTAATTCTCACCG	
HelB4-KpnI-F	CATTAA**GGTACC**ATGGTAGAAATCCTCCGT	Cloning of *helB4* from *A. fumigatus* Af293 genome
HelB4-KpnI-R	CATTAA**GGTACC**TTACTGCCTGCGAGACAC	
HelC-EcoRI-F	AAC**GAATTC**ATGAACACGGCCATCATC	Cloning of *helC* from *A. fumigatus* Af293 genome
HelC-EcoRI-R	AAC**GAATTC**CTCTACGTATATCCATCATG	
HelD1-EcoRI-F	AAC**GAATTC**ATGGAGTCAATCCCCCTCTC	Cloning of *helD1* from *A. fumigatus* Af293 genome
HelD1-KpnI-R	CATTAA**GGTACC**CTACCAAATTATCTCTACCAGCC	
HelD2-KpnI-F	CATTAA**GGTACC**ATGGAAGGCCTCAACATAT	Cloning of *helD2* from *A. fumigatus* Af293 genome
HelD2-KpnI-R	CATTAA**GGTACC**GGCGATGACGTTTGCTTTTATAG	
HelE-EcoRI-F	AAC**GAATTC**ATGGCCGCAAGACAGCT	Cloning of *helE* from *A. fumigatus* Af293 genome
HelE-EcoRI-R	AAC**GAATTC**CTATACATGCTCAGAAGCAATATGG	
pTAex3-Pamy-F	CGACTCCAATCTTCAAGAGC	Cloning of target gene containing amyB promoter and terminator from recombinant pTAex3 plasmid
pTAex3-Tamy-R	GTAAGATACATGAGCTTCGG	
Inf-pAdeA-Pamy-F	GCAGGTCGACTCTAGACGACTCCAATCTTCAAGAGC	Construction of recombinant pAdeA and pPTRI plasmids containing two or three exogenous genes using the In-Fusion® HD Cloning Kit
Inf-pAdeA-Tamy-R	TAGTAGATCCTCTAGAGTAAGATACATGAGCTTCGG	
Inf-pPTRI-Pamy-F	TGATTACGCCAAGCTCGACTCCAATCTTCAAGAGC	
Inf-pPTRI-Tamy-R	GCAGGCATGCAAGCTGTAAGATACATGAGCTTCGG	
Inf-pTAex3-Pamy-F1	GCTCGCGAGCGCGTTCCACTGCATCATCAGTCTAG	
Inf-pTAex3-Pamy-F2	TCGCGTGCGCGTTTACCCATCATGGTGTTTTGATC	
Inf-pTAex3-Tamy-R1	AACGCGCTCGCGAGCAAGTACCATACAGTACCGCG	
Inf-pTAex3-Tamy-R2	TAAACGCGCACGCGACATTAATCCGGATCCTTTCC	
HelC-NdeI-F	GGAATTC**CATATG**ATGAACACGGCCATCATCA	Cloning of *helC* from pTAex3-*helC* for construction of pET28a-*helC*
HelC-HindIII-R	CCC**AAGCTT**CTCTACGTATATCCATCATG	

Supplementary Table 19. Plasmids used in the study

Plasmids	Characteristics	Source
pTAex3	Plasmid containing *argB* maker gene cassette for gene expression in *A. oryzae* NSAR1, (*Amp^R*)	Fujii, T. *et al.*[1]
pUSA	Plasmid containing *sC* maker gene cassette for gene expression in *A. oryzae* NSAR1, (*Amp^R*)	Yamada, O. *et al.*[2]
pAdeA	Plasmid containing *adeA* maker gene cassette for gene expression in *A. oryzae* NSAR1, (*Amp^R*)	Jin, F. *et al.*[3]
pPTRI	Plasmid containing *ptrA* maker gene cassette for gene expression in *A. oryzae* NSAR1, (*Amp^R*)	TaKaRa
pET28a	Plasmid for gene expression in *E. coli* BL21(DE3)	Novagen
pTAex3-*helA*	pTAex3 containing *helA* whose expression is regulated by *amyB* promoter, (*Amp^R*)	This work
pTAex3-*helB2*	pTAex3 containing *helB2* whose expression is regulated by *amyB* promoter, (*Amp^R*)	This work
pTAex3-*helB3*	pTAex3 containing *helB3* whose expression is regulated by *amyB* promoter, (*Amp^R*)	This work
pTAex3-*helB4*	pTAex3 containing *helB4* whose expression is regulated by *amyB* promoter, (*Amp^R*)	This work
pTAex3-*helC*	pTAex3 containing *helC* whose expression is regulated by *amyB* promoter, (*Amp^R*)	This work
pTAex3-*helD1*	pTAex3 containing *helD1* whose expression is regulated by *amyB* promoter, (*Amp^R*)	This work
pTAex3-*helD2*	pTAex3 containing *helD2* whose expression is regulated by *amyB* promoter, (*Amp^R*)	This work
pTAex3-*helE*	pTAex3 containing *helE* whose expression is regulated by *amyB* promoter, (*Amp^R*)	This work
pUSA-*helB1*	pUSA containing *helB1* whose expression is regulated by *amyB* promoter, (*Amp^R*)	This work
pUSA-*helC*	pUSA containing *helC* whose expression is regulated by *amyB* promoter, (*Amp^R*)	This work
pUSA-*helB1-helC*	pUSA containing *helB1* and *helC* whose expressions are independently regulated by *amyB* promoter, (*Amp^R*)	This work
pAdeA-*helB2*	pAdeA containing *helB2* whose expression is regulated by *amyB* promoter, (*Amp^R*)	This work
pAdeA-*helB3*	pAdeA containing *helB3* whose expression is regulated by *amyB* promoter, (*Amp^R*)	This work
pAdeA-*helB4*	pAdeA containing *helB4* whose expression is regulated by *amyB* promoter, (*Amp^R*)	This work
pAdeA-*helE*	pAdeA containing *helE* whose expression is regulated by *amyB* promoter, (*Amp^R*)	This work
pAdeA-*helB2-helB3*	pAdeA containing *helB2* and *helB3* whose expressions are independently regulated by *amyB* promoter, (*Amp^R*)	This work
pAdeA-*helB2-helB4*	pAdeA containing *helB2* and *helB4* whose expressions are independently regulated by *amyB* promoter, (*Amp^R*)	This work
pAdeA-*helB2-helE*	pAdeA containing *helB2* and *helE* whose expressions are independently regulated by *amyB* promoter, (*Amp^R*)	This work
pAdeA-*helB2-helD1*	pAdeA containing *helB2* and *helD1* whose expressions are independently regulated by *amyB* promoter, (*Amp^R*)	This work
pAdeA-*helB2-helD2*	pAdeA containing *helB2* and *helD2* whose expressions are independently regulated by *amyB* promoter, (*Amp^R*)	This work
pAdeA-*helB2-helD2-helB3*	pAdeA containing *helB2*, *helD2* and *helB3* whose expressions are independently regulated by *amyB* promoter, (*Amp^R*)	This work
pAdeA-*helB2-helD2-helB4*	pAdeA containing *helB2*, *helD2* and *helB4* whose expressions are independently regulated by *amyB* promoter, (*Amp^R*)	This work
pAdeA-*helB2-helD2-helE*	pAdeA containing *helB2*, *helD2* and *helE* whose expressions are independently regulated by *amyB* promoter, (*Amp^R*)	This work
pAdeA-*helB2-helB3-helB4*	pAdeA containing *helB2*, *helB3* and *helB4* whose expressions are independently regulated by *amyB* promoter, (*Amp^R*)	This work
pPTRI-*helB3*	pPTRI containing *helB3* whose expression is regulated by *amyB* promoter, (*Amp^R*)	This work
pPTRI-*helE*	pPTRI containing *helE* whose expression is regulated by *amyB* promoter, (*Amp^R*)	This work
pPTRI-*helB3-helD1*	pPTRI containing *helB3* and *helD1* whose expressions are independently regulated by *amyB* promoter, (*Amp^R*)	This work
pPTRI-*helB3-helE*	pPTRI containing *helB3* and *helE* whose expressions are independently regulated by *amyB* promoter, (*Amp^R*)	This work
pPTRI-*helD1-helD2*	pPTRI containing *helD1* and *helD2* whose expressions are independently regulated by *amyB* promoter, (*Amp^R*)	This work
pPTRI-*helD1-helD2-helE*	pPTRI containing *helD1*, *helD2* and *helE* whose expressions are independently regulated by *amyB* promoter, (*Amp^R*)	This work
pET28a-*helC*	pET28a containing *helC* whose expression is regulated by T7 promoter, (*Kan^R*)	This work

Supplementary Table 20. Strains used in the study

Strains	Characteristics	Source
E. coli DH5α	Host for general plasmid cloning	TaKaRa
E. coli BL21(DE3)	Host for gene expression	TaKaRa
A. oryzae NSAR1	Host for gene expression, a quadruple auxotrophic mutant strain (*niaD*$^-$, *sC*$^-$, *ΔargB*, *adeA*$^-$)	Jin, F. J. *et al.*[4]
AO1	*A. oryzae* NSAR1 transformant harboring *helA* and *helC*	This work
AO2	*A. oryzae* NSAR1 transformant harboring *helA* and *helB1*	This work
AO3	*A. oryzae* NSAR1 transformant harboring *helA*, *helB1* and *helC*	This work
AO4	*A. oryzae* NSAR1 transformant harboring *helA*, *helB1*, *helC* and *helB2*	This work
AO5	*A. oryzae* NSAR1 transformant harboring *helA*, *helB1*, *helC* and *helB3*	This work
AO6	*A. oryzae* NSAR1 transformant harboring *helA*, *helB1*, *helC* and *helB4*	This work
AO7	*A. oryzae* NSAR1 transformant harboring *helA*, *helB1*, *helC* and *helE*	This work
AO8	*A. oryzae* NSAR1 transformant harboring *helA*, *helB1*, *helC*, *helB2* and *helB3*	This work
AO9	*A. oryzae* NSAR1 transformant harboring *helA*, *helB1*, *helC*, *helB2* and *helB4*	This work
AO10	*A. oryzae* NSAR1 transformant harboring *helA*, *helB1*, *helC*, *helB2* and *helE*	This work
AO11	*A. oryzae* NSAR1 transformant harboring *helA*, *helB1*, *helC*, *helB2* and *helD1*	This work
AO12	*A. oryzae* NSAR1 transformant harboring *helA*, *helB1*, *helC*, *helB2* and *helD2*	This work
AO13	*A. oryzae* NSAR1 transformant harboring *helA*, *helB1*, *helC*, *helB2*, *helD2* and *helB3*	This work
AO14	*A. oryzae* NSAR1 transformant harboring *helA*, *helB1*, *helC*, *helB2*, *helD2* and *helB4*	This work
AO15	*A. oryzae* NSAR1 transformant harboring *helA*, *helB1*,*helC*, *helB2*, *helD2* and *helE*	This work
AO16	*A. oryzae* NSAR1 transformant harboring *helA*, *helB1*, *helB2*, *helD2* and *helB4*	This work
AO17	*A. oryzae* NSAR1 transformant harboring *helA*, *helB1*, *helC*, *helB2*, *helD2*, *helB4* and *helB3*	This work
AO18	*A. oryzae* NSAR1 transformant harboring *helA*, *helB1*, *helC*, *helB2*, *helD2*, *helB4* and *helE*	This work
AO19	*A. oryzae* NSAR1 transformant harboring *helA*, *helB1*, *helC*, *helB2*, *helD2*, *helB4*, *helB3* and *helD1*	This work
AO20	*A. oryzae* NSAR1 transformant harboring *helA*, *helB1*, *helC*, *helB2*, *helD2*, *helB4*, *helB3* and *helE*	This work
AO21	*A. oryzae* NSAR1 transformant harboring *helA*, *helB1*, *helC*, *helB2*, *helD2*, *helB4*, *helB3*, *helD1* and *helE*	This work
AO22	*A. oryzae* NSAR1 transformant harboring *helA*, *helB1*, *helB2*, *helD2*, *helB4*, *helB3* and *helE*	This work
AO23	*A. oryzae* NSAR1 transformant harboring *helB1*	This work
AO24	*A. oryzae* NSAR1 transformant harboring *helB2*	This work
AO25	*A. oryzae* NSAR1 transformant harboring *helB4*	This work
AO26	*A. oryzae* NSAR1 transformant harboring *helD2*	This work

Supplementary Notes

Supplementary Note 1. Structural characterization of compound 1

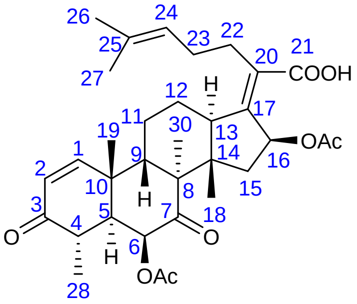

1

Compound **1** was obtained as a white powder. The quasi-molecular ion at *m/z* 591.2932 $[M + Na]^+$ (calcd for $C_{33}H_{44}O_8Na$, 591.2934) by HRESIMS indicated that the molecular formula of **1** was $C_{33}H_{44}O_8$ (12 degrees of unsaturation). ^{1}H NMR (400 MHz, $CDCl_3$) δ_H 7.30 (d, J = 10.0 Hz, 1H), 5.87 (br d, J = 7.6 Hz, 1H), 5.86 (d, J = 10.0 Hz, 1H), 5.23 (br s, 1H), 5.10 (br t, J = 6.8 Hz, 1H), 2.77 (dq, J = 13.2, 6.4 Hz, 1H), 2.61 (br d, J = 12.8 Hz, 1H), 2.57 (br d, J = 12.8 Hz, 1H), 2.48 (m, 2H), 2.43 (m, 1H), 2.26 (br d, J = 13.2 Hz, 1H), 2.24 (m, 1H), 2.13 (m, 1H), 2.10 (s, 3H), 2.08 (m, 1H), 1.96 (m, 1H), 1.94 (s, 3H), 1.91 (br d, J = 15.6 Hz, 1H), 1.80 (m, 1H), 1.68 (br s, 3H), 1.60 (br s, 3H), 1.56 (m, 1H), 1.44 (s, 3H), 1.27 (d, J = 6.4 Hz, 3H), 1.18 (s, 3H), 0.92 (s, 3H); ^{13}C NMR (100 MHz, $CDCl_3$) δ_C 208.7, 201.3, 173.9, 170.2, 168.9, 157.2, 147.7, 132.9, 130.3, 127.8, 122.8, 73.8, 73.4, 52.6, 49.4, 47.2, 46.6, 41.7, 40.6, 40.4, 38.1, 28.6, 28.3, 27.5, 25.9, 25.7, 23.9, 20.7, 20.5, 18.3, 17.9, 17.7, 13.1. The NMR data were in good agreement with those of 6*β*,16*β*-diacetyloxy-29-norprotosta-1,17(20)*Z*,24-trien-3,7-dione-21-oic acid (helvolic acid[5]).

Supplementary Note 2. Structural characterization of compound 2

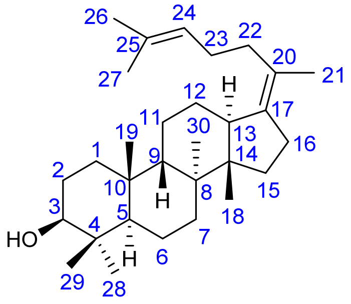

2

Compound **2** was obtained as a colorless oil. ^{1}H NMR (400 MHz, $CDCl_3$) δ_H 5.10 (m, 1H), 3.24 (dd, J = 11.6, 5.2 Hz, 1H), 2.30 (m, 1H), 2.21 (m, 1H), 2.14 (m, 1H), 2.07 (m, 1H), 2.06 (m, 1H), 2.05 (m, 2H), 1.98 (m, 1H), 1.95 (m, 1H), 1.70 (m, 1H), 1.68 (br s, 3H), 1.60 (br s, 3H), 1.60 (m, 1H), 1.58 (s, 3H), 1.53 (m, 1H), 1.50 (m, 1H), 1.49 (m, 1H), 1.47 (m, 1H), 1.43 (m, 1H), 1.42 (m, 2H), 1.35 (m, 1H), 1.27 (m, 1H), 1.20 (m, 1H), 1.19 (m, 1H), 1.12 (s, 3H), 1.11 (m, 1H), 0.98 (s, 3H), 0.92 (s, 3H), 0.79 (s, 3H), 0.75 (s, 3H); ^{13}C NMR (100 MHz, $CDCl_3$) δ_C 136.7, 131.1, 126.4, 124.7, 79.3, 50.6, 47.6, 46.8, 45.4, 39.2, 38.9, 36.7, 34.7, 33.7, 33.0, 30.3, 29.3, 29.1, 29.1, 28.1, 27.3, 25.7, 22.9, 22.6, 22.0, 20.8, 18.3, 17.7, 16.9, 16.1. The NMR data were in good agreement with those of protosta-17(20)*Z*,24-dien-3*β*-ol[6].

Supplementary Note 3. Structural characterization of compound 3

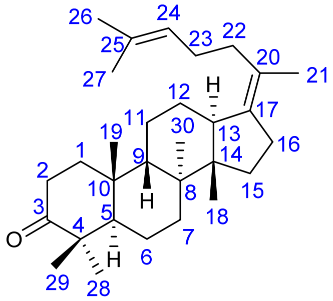

3

Compound **3** was obtained as a colorless oil. The quasi-molecular ion at *m/z* 425.3793 $[M + H]^+$ (calcd for $C_{30}H_{49}O$, 425.3783) by HRESIMS indicated that the molecular formula of **3** was $C_{30}H_{48}O$ (7 degrees of unsaturation). ^1H NMR (400 MHz, $CDCl_3$) δ_H 5.09 (m, 1H), 2.65 (ddd, J = 15.2, 12.0, 6.0 Hz, 1H), 2.31 (m, 1H), 2.26 (m, 1H), 2.23 (m, 1H), 2.15 (m, 1H), 2.14 (m, 1H), 2.11 (m, 1H), 2.09 (m, 1H), 2.08 (m, 1H), 2.05 (m, 2H), 2.01 (m, 1H), 1.98 (m, 1H), 1.66 (br s, 3H), 1.58 (br s, 3H), 1.57 (br s, 3H), 1.56 (m, 1H), 1.50 (m, 1H), 1.46 (m, 1H), 1.45 (m, 1H), 1.41 (m, 1H), 1.40 (m, 1H), 1.35 (m, 1H), 1.21 (m, 1H), 1.21 (m, 1H), 1.16 (s, 3H), 1.14 (m, 1H), 1.05 (s, 3H), 1.02 (s, 3H), 0.78 (s, 3H), 0.76 (s, 3H); ^{13}C NMR (100 MHz, $CDCl_3$) δ_C 220.3, 136.5, 131.1, 126.6, 124.6, 50.4, 47.0, 47.0, 46.8, 42.9, 39.0, 35.9, 33.9, 33.7, 33.7, 31.3, 30.3, 29.3, 29.1, 28.0, 27.0, 25.7, 23.4, 22.4, 21.5, 20.8, 19.8, 19.5, 17.6, 16.8. The NMR data were in good agreement with those of protosta-17(20)*Z*,24-dien-3-one[7].

Supplementary Note 4. Structural characterization of compound 4

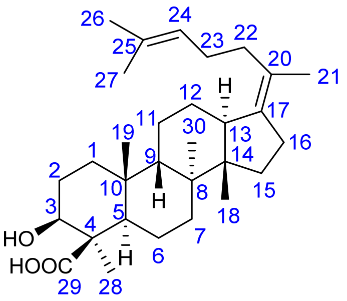

4

Compound **4** was obtained as a white powder. The quasi-molecular ion at *m/z* 457.3665 $[M + H]^+$ (calcd for $C_{30}H_{49}O_3$, 457.3682) by HRESIMS indicated that the molecular formula of **4** was $C_{30}H_{48}O_3$ (7 degrees of unsaturation). Combined with the DEPT-135 experiment, the ^{13}C NMR spectrum of **4** showed 30 carbons, assigned to one carbonyl carbon, four olefinic carbons (including one sp^2 methine carbon), four sp^3 quaternary carbons, four sp^3 methine carbons (including one oxygenated carbon), ten sp^3 methylene carbons, and seven sp^3 methyl carbons. In the ^1H NMR spectrum of **4**, the characteristic signals of seven sets of methyl protons [δ_H 1.67 (3H, br s), 1.59 (3H, br s), 1.57 (3H, s), 1.44 (3H, s), 1.11 (3H, s), 0.91 (3H, s), 0.76 (3H, s)] were observed. The nonexchangeable proton resonances were associated with the directly attached carbon atoms in the HSQC experiment (Supplementary Table 1). Except for the loss of one methyl carbon (δ_C 16.1), and the appearance of one carbonyl carbon (δ_C 183.9), the NMR data of **4** were similar to those of **2**, which revealed that **4** might be an oxygenated derivative of **2**. Combined with molecular formula and degrees of unsaturation, further detailed NMR analyses of ^1H-^1H COSY and HMBC spectra established the planar structure of **4**, in which the assignments of C-26 and C-27 were determined on the basis of comparison of chemical shifts with those of C-26 and C-27 in **2**, and the NOESY correlation between H-24 and H_3-26. On the basis of the NOESY correlations between Ha-2 and H_3-19, between H-3 and H-5/H_3-28, between H_3-30 and H-5/H-13, and between H-9 and H_3-18 (Supplementary Table 1), the relative configuration of **4** was determined as 3*S**, 4*R**, 5*R**, 8*S**, 9*S**, 10*R**, 13*S**, 14*S**. Because NMR data of C-13/C-14/C-15/C-16/C-17/C-18/C-20/C-21/C-22/C-23 in **4** were the same as those in **2**, and the NOESY correlation between Hb-16 and H_3-21 was observed (Supplementary Table 1), the geometric configuration of the double bond between C-17 and C-20 was determined as *Z*.

Considering that **4** was biosynthesized from the precursor **2**, the absolute configuration of C-10 in **4** was assigned as *R*. Thus, the absolute configuration of **4** was assigned as 3*S*, 4*R*, 5*R*, 8*S*, 9*S*, 10*R*, 13*S*, 14*S*. Based on the above analyses, the structure of **4** was the same as that of 3*β*-hydroxy-protosta-17(20)*Z*,24-dien-29-oic acid [protosta-17(20)*Z*,24-dien-3*β*-ol-29-oic acid[7]], but the NMR data were slightly revised.

Supplementary Note 5. Structural characterization of compound 5

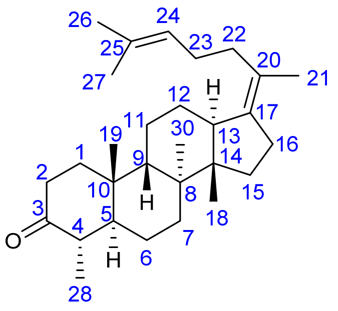

5

Compound **5** was obtained as a colorless oil. The quasi-molecular ion at *m/z* 411.3600 $[M + H]^+$ (calcd for $C_{29}H_{47}O$, 411.3627) by HRESIMS indicated that the molecular formula of **5** was $C_{29}H_{46}O$ (7 degrees of unsaturation). Combined with the DEPT-135 experiment, the ^{13}C NMR spectrum of **5** showed 29 carbons, assigned to one carbonyl carbon, four olefinic carbons (including one sp^2 methine carbon), three sp^3 quaternary carbons, four sp^3 methine carbons, ten sp^3 methylene carbons, and seven sp^3 methyl carbons. In the ^1H NMR spectrum of **5**, the characteristic signals of seven sets of methyl protons [δ_H 1.68 (3H, br s), 1.60 (3H, br s), 1.58 (3H, s), 1.10 (3H, s), 1.02 (3H, d, 6.8), 1.00 (3H, s), 0.78 (3H, s)] were observed. The nonexchangeable proton resonances were associated with the directly attached carbon atoms in the HSQC experiment (Supplementary Table 2). Except for the loss of one methyl carbon (δ_C 19.5) and one sp^3 quaternary carbon (δ_C 47.0), and the appearance of one sp^3 methine carbon (δ_C 45.0), the NMR data of **5** were similar to those of **3**, which revealed that **5** might be a demethylated derivative of **3**. Combined with molecular formula and degrees of unsaturation, further detailed NMR analyses of ^1H-^1H COSY and HMBC spectra established the planar structure of **5**, in which the assignments of C-26 and C-27 were determined on the basis of comparison of chemical shifts with those of C-26 and C-27 in **3**, and the ROESY correlation between H-24 and H_3-26. On the basis of the coupling constant between H-4 and H-5 ($^3J_{H\text{-}4,\ H\text{-}5}$ = 12.0 Hz), and the ROESY correlations between H-4 and H_3-19, between H_3-30 and H-5/H-13, and between H-9 and H_3-18 (Supplementary Table 2), the relative configuration of **5** was determined as 4*S**, 5*S**, 8*S**, 9*S**, 10*S**, 13*S**, 14*S**. Because NMR data of C-13/C-14/C-15/C-16/C-17/C-18/C-20/C-21/C-22/C-23 in **5** were the same as those in **3**, and the ROESY correlation between Hb-16 and H_3-21 was observed (Supplementary Table 2), the geometric configuration of the double bond between C-17 and C-20 was determined as

Z. Considering that **5** was biosynthesized from the precursor **2**, the absolute configuration of C-10 in **5** was assigned as *S*. Thus, the absolute configuration of **5** was assigned as 4*S*, 5*S*, 8*S*, 9*S*, 10*S*, 13*S*, 14*S*. Based on the above analyses, the structure of **5** was the same as that of 29-norprotosta-17(20)*Z*,24-dien-3-one [3-oxofusida-17(20)*Z*,24-diene[8]], the detailed NMR assignments of which were not available prior to the present study.

Supplementary Note 6. Structural characterization of compound 6

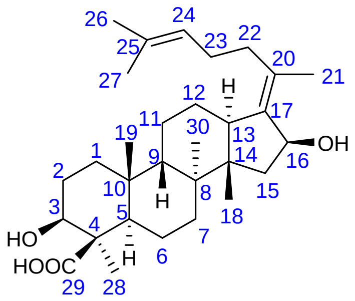

6

Compound **6** was obtained as a white powder. The quasi-molecular ion at *m/z* 473.3622 $[M + H]^+$ (calcd for $C_{30}H_{49}O_4$, 473.3631) by HRESIMS indicated that the molecular formula of **6** was $C_{30}H_{48}O_4$ (7 degrees of unsaturation). Combined with the DEPT-135 experiment, the ^{13}C NMR spectrum of **6** showed 30 carbons, assigned to one carbonyl carbon, four olefinic carbons (including one sp^2 methine carbon), four sp^3 quaternary carbons, five sp^3 methine carbons (including two oxygenated carbons), nine sp^3 methylene carbons, and seven sp^3 methyl carbons. In the ^1H NMR spectrum of **6**, the characteristic signals of seven sets of methyl protons [δ_H 1.83 (3H, br s), 1.68 (3H, br s), 1.60 (3H, br s), 1.43 (3H, s), 1.09 (3H, s), 1.05 (3H, s), 0.90 (3H, s)] were observed. The nonexchangeable proton resonances were associated with the directly attached carbon atoms in the HSQC experiment (Supplementary Table 3). Except for the loss of one methylene carbon (δ_C 29.2) and the appearance of one oxygenated methine carbon (δ_C 73.1), the NMR data of **6** were similar to those of **4**, which revealed that **6** might be a hydroxylated derivative of **4**. Combined with molecular formula and degrees of unsaturation, further detailed NMR analyses of ^1H-^1H COSY and HMBC spectra established the planar structure of **6**, in which the assignments of C-26 and C-27 were determined on the basis of comparison of chemical shifts with those of C-26 and C-27 in **4**. On the basis of the NOESY correlations between Ha-2 and H_3-19, between H-3 and H-5/H_3-28, between H_3-30 and H-5/H-13, between H-9 and H_3-18, and between H-13 and H-16 (Supplementary Table 3), the relative configuration of **6** was determined as 3*S**, 4*R**, 5*R**, 8*S**, 9*S**, 10*R**, 13*R**, 14*S**, 16*S**. Combined with the correlation between H-24 and H-13 in the 1D-selective NOE experiment (Supplementary Fig. 33i) and the NOESY correlation between H-16 and H_3-21 (Supplementary Table 3), the geometric configuration of the double bond between C-17 and C-20 was determined as *E*. Considering that **6** was

biosynthesized from the precursor **2**, the absolute configuration of C-10 in **6** was assigned as *R*. Thus, the absolute configuration of **6** was assigned as 3*S*, 4*R*, 5*R*, 8*S*, 9*S*, 10*R*, 13*R*, 14*S*, 16*S*. Therefore, **6** was identified as (3*S*,4*R*,5*R*,8*S*,9*S*,10*R*,13*R*,14*S*,16*S*,*E*)-3,16-dihydroxy-4,8,10,14-tetramethyl-17-(6-methylhept-5-en-2-ylidene)hexadecahydro-1*H*-cyclopenta[*a*]phenanthrene-4-carboxylic acid, and named 3*β*,16*β*-dihydroxy-protosta-17(20)*E*,24-dien-29-oic acid.

Supplementary Note 7. Structural characterization of compound 7

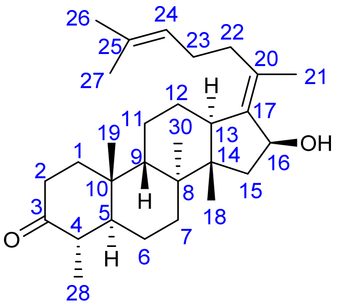

7

Compound **7** was obtained as a colorless oil. The quasi-molecular ion at *m/z* 427.3572 $[M + H]^+$ (calcd for $C_{29}H_{47}O_2$, 427.3576) by HRESIMS indicated that the molecular formula of **7** was $C_{29}H_{46}O_2$ (7 degrees of unsaturation). Combined with the DEPT-135 experiment, the ^{13}C NMR spectrum of **7** showed 29 carbons, assigned to one carbonyl carbon, four olefinic carbons (including one sp^2 methine carbon), three sp^3 quaternary carbons, five sp^3 methine carbons (including one oxygenated carbon), nine sp^3 methylene carbons, and seven sp^3 methyl carbons. In the ^1H NMR spectrum of **7**, the characteristic signals of seven sets of methyl protons [δ_H 1.85 (3H, d, 2.0), 1.69 (3H, br s), 1.61 (3H, br s), 1.08 (3H, s), 1.08 (3H, s), 1.02 (3H, d, 5.7), 1.01 (3H, s)] were observed. The nonexchangeable proton resonances were associated with the directly attached carbon atoms in the HSQC experiment (Supplementary Table 4). Except for the loss of one methylene carbon (δ_C 29.2) and the appearance of one oxygenated methine carbon (δ_C 73.1), the NMR data of **7** were similar to those of **5**, which revealed that **7** might be a hydroxylated derivative of **5**. Combined with molecular formula and degrees of unsaturation, further detailed NMR analyses of ^1H-^1H COSY and HMBC spectra established the planar structure of **7**, in which the assignments of C-26 and C-27 were determined on the basis of comparison of chemical shifts with those of C-26 and C-27 in **5**. On the basis of the coupling constant between H-4 and H-5 ($^3J_{H\text{-}4,\ H\text{-}5}$ = 12.0 Hz), and the ROESY correlations between H-4 and H_3-19, between H_3-30 and H-5/H-13, between H-9 and H_3-18, and between H-13 and H-16 (Supplementary Table 4), the relative configuration of **7** was determined as 4*S**, 5*S**, 8*S**, 9*S**, 10*S**, 13*R**, 14*S**, 16*S**. Because NMR data of C-13/C-14/C-15/C-16/C-17/C-18/C-20/C-21/C-22/C-23 in **7** were the same as those in **6**, and the ROESY correlation between H-16 and H_3-21 was observed (Supplementary Table 4), the geometric configuration of the double bond between C-17 and C-20 was determined as *E*.

Considering that **7** was biosynthesized from the precursor **2**, the absolute configuration of C-10 in **7** was assigned as *S*. Thus, the absolute configuration of **7** was assigned as 4*S*, 5*S*, 8*S*, 9*S*, 10*S*, 13*R*, 14*S*, 16*S*. Therefore, **7** was identified as (4*S*,5*S*,8*S*,9*S*,10*S*,13*R*,14*S*,16*S*,*E*)-16-hydroxy-4,8,10,14-tetramethyl-17-(6-methylhept-5-en-2-ylidene)hexadecahydro-3*H*-cyclopenta[*a*]phenanthren-3-one, and named 16*β*-hydroxy-29-norprotosta-17(20)*E*,24-dien-3-one.

Supplementary Note 8. Structural characterization of compound 8

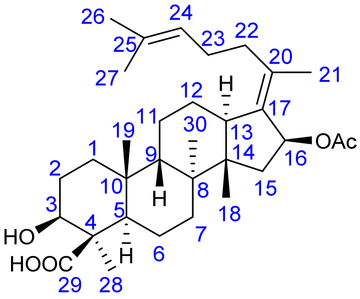

8

Compound **8** was obtained as a yellowish powder. The quasi-molecular ion at *m/z* 537.3557 $[M + Na]^+$ (calcd for $C_{32}H_{50}O_5Na$, 537.3556) by HRESIMS indicated that the molecular formula of **8** was $C_{32}H_{50}O_5$ (8 degrees of unsaturation). Combined with the DEPT-135 experiment, the ^{13}C NMR spectrum of **8** showed 32 carbons, assigned to two carbonyl carbons, four olefinic carbons (including one sp^2 methine carbon), four sp^3 quaternary carbons, five sp^3 methine carbons (including two oxygenated carbons), nine sp^3 methylene carbons, and eight sp^3 methyl carbons. In the ^1H NMR spectrum of **8**, the characteristic signals of eight sets of methyl protons [δ_H 2.02 (3H, s), 1.68 (3H, br s), 1.59 (3H, br s), 1.59 (3H, br s), 1.43 (3H, s), 1.09 (3H, s), 0.96 (3H, s), 0.91 (3H, s)] were observed. The nonexchangeable proton resonances were associated with the directly attached carbon atoms in the HSQC experiment (Supplementary Table 5). Except for the appearance of one carbonyl carbon (δ_C 171.1) and one methyl carbon (δ_C 21.2), the NMR data of **8** were similar to those of **6**, which revealed that **8** might be an acetylated derivative of **6**. Combined with molecular formula and degrees of unsaturation, further detailed NMR analyses of ^1H-^1H COSY and HMBC spectra established the planar structure of **8**, in which the assignments of C-26 and C-27 were determined on the basis of comparison of chemical shifts with those of C-26 and C-27 in **6**, and the ROESY correlation between H-24 and H_3-26. On the basis of the ROESY correlations between Ha-2 and H_3-19, between H-3 and H-5/H_3-28, between H_3-30 and H-5/H-13, between H-9 and H_3-18, and between H-13 and H-16 (Supplementary Table 5), the relative configuration of **8** was determined as 3*S**, 4*R**, 5*R**, 8*S**, 9*S**, 10*R**, 13*R**, 14*S**, 16*S**. Combined with the correlation betweeen H-24 and H-13 in the 1D-selective NOE experiment (Supplementary Fig. 35h) and the ROESY correlation between H-16 and H_3-21 (Supplementary Table 5), the geometric configuration of the double bond between C-17 and

C-20 was determined as *E*. Considering that **8** was biosynthesized from the precursor **2**, the absolute configuration of C-10 in **8** was assigned as *R*. Thus, the absolute configuration of **8** was assigned as 3*S*, 4*R*, 5*R*, 8*S*, 9*S*, 10*R*, 13*R*, 14*S*, 16*S*. Therefore, **8** was identified as (3*S*,4*R*,5*R*,8*S*,9*S*,10*R*,13*R*,14*S*,16*S*,*E*)-16-acetoxy-3-hydroxy-4,8,10,14-tetramethyl-17-(6-methylhept-5-en-2-ylidene)hexadecahydro-1*H*-cyclopenta[*a*]phenanthrene-4-carboxylic acid, and named 16*β*-acetyloxy-3*β*-hydroxy-protosta-17(20)*E*,24-dien-29-oic acid.

Supplementary Note 9. Structural characterization of compound 9

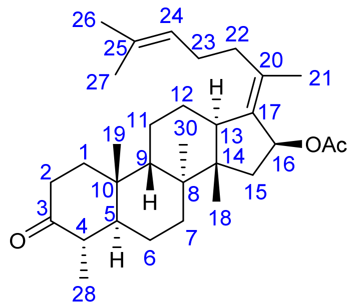

9

Compound **9** was obtained as a colorless oil. The quasi-molecular ion at *m/z* 491.3472 $[M + Na]^+$ (calcd for $C_{31}H_{48}O_3Na$, 491.3501) by HRESIMS indicated that the molecular formula of **9** was $C_{31}H_{48}O_3$ (8 degrees of unsaturation). Combined with the DEPT-135 experiment, the ^{13}C NMR spectrum of **9** showed 31 carbons, assigned to two carbonyl carbons, four olefinic carbons (including one sp^2 methine carbon), three sp^3 quaternary carbons, five sp^3 methine carbons (including one oxygenated carbon), nine sp^3 methylene carbons, and eight sp^3 methyl carbons. In the ^1H NMR spectrum of **9**, the characterized signals of eight sets of methyl protons [δ_H 2.03 (3H, s), 1.69 (3H, br s), 1.61 (3H, br s), 1.61 (3H, br s), 1.08 (3H, s), 1.02 (3H, d, 7.2), 1.01 (3H, s), 0.98 (3H, s)] were observed. The nonexchangeable proton resonances were associated with the directly attached carbon atoms in the HSQC experiment (Supplementary Table 6). Except for the appearance of one carbonyl carbon (δ_C 171.0) and one methyl carbon (δ_C 21.1), the NMR data of **9** were similar to those of **7**, which revealed that **9** may be an acetylated derivative of **7**. Combined with molecular formula and degrees of unsaturation, further detailed NMR analyses of ^1H-^1H COSY and HMBC spectra established the planar structure of **9**, in which the assignments of C-26 and C-27 were determined on the basis of comparison of chemical shifts with those of C-26 and C-27 in **7**, and the ROESY correlation between H-24 and H_3-26. On the basis of the coupling constant between H-4 and H-5 ($^3J_{H\text{-}4,\,H\text{-}5}$ = 12.0 Hz), and the ROESY correlations between H-4 and H_3-19, between H_3-30 and H-5/H-13, between H-9 and H_3-18, and between H-13 and H-16 (Supplementary Table 6), the relative configuration of **9** was determined as 4*S**, 5*S**, 8*S**, 9*S**, 10*S**, 13*R**, 14*S**, 16*S**. Because NMR data of C-13/C-14/C-15/C-16/C-17/C-18/C-20/C-21/C-22/C-23 in **9** were the same as those in **8**, and the ROESY correlation between H-16 and H_3-21 was observed (Supplementary Table 6), the

geometric configuration of the double bond between C-17 and C-20 was determined as *E*. Considering that **9** was biosynthesized from the precursor **2**, the absolute configuration of C-10 in **9** was assigned as *S*. Thus, the absolute configuration of **9** was assigned as 4*S*, 5*S*, 8*S*, 9*S*, 10*S*, 13*R*, 14*S*, 16*S*. Therefore, **9** was identified as (4*S*,5*S*,8*S*,9*S*,10*S*,13*R*,14*S*,16*S*,*E*)-4,8,10,14-tetramethyl-17-(6-methylhept-5-en-2-ylidene)-3-oxohexadecahydro-1*H*-cyclopenta[*a*]phenanthren-16-yl acetate, and named 16*β*-acetyloxy-29-norprotosta-17(20)*E*,24-dien-3-one.

Supplementary Note 10. Structural characterization of compound 10

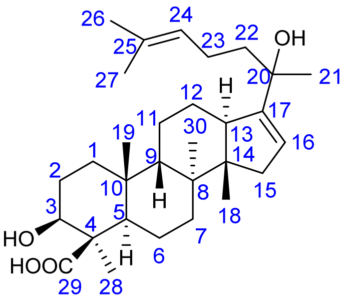

10

Compound **10** was obtained as a white powder. The quasi-molecular ion at *m/z* 473.3615 $[M + H]^+$ (calcd for $C_{30}H_{49}O_4$, 473.3631) by HRESIMS indicated that the molecular formula of **10** was $C_{30}H_{48}O_4$ (7 degrees of unsaturation). Combined with the DEPT-135 experiment, the ^{13}C NMR spectrum of **10** showed 29 carbons, assigned to four olefinic carbons (including two sp^2 methine carbons), five sp^3 quaternary carbons (including one oxygenated carbon), four sp^3 methine carbons (including one oxygenated carbon), nine sp^3 methylene carbons, and seven sp^3 methyl carbons. In the ^1H NMR spectrum of **10**, the characteristic signals of seven sets of methyl protons [δ_H 1.73 (3H, s), 1.71 (3H, br s), 1.66 (3H, br s), 1.56 (3H, s), 1.22 (3H, s), 1.22 (3H, s), 1.12 (3H, s)] were observed. The nonexchangeable proton resonances were associated with the directly attached carbon atoms in the HSQC experiment (Supplementary Table 7). The analyses of the ^1H-^1H COSY and ^1H NMR spectra of **10** revealed the presence of some spin systems: C-1–C-2–C-3, C-5–C-6–C-7, C-9–C-11–C-12–C-13, C-15–C-16, C-22–C-23–C-24. Combined with the ^1H-^1H COSY data, molecular formula and degrees of unsaturation, the HMBC correlations from H-13 to C-17, from Hb-15 to C-16/C-17, from H-16 to C-14, from H_3-18 to C-8/C-13/C-14/C-15, from H_3-19 to C-1/C-5/C-9/C-10, from H_3-21 to C-17/C-20/C-22, from H_3-26 to C-24/C-25/C-27, from H_3-27 to C-24/C-25/C-26, from H_3-28 to C-3/C-4/C-5/C-29, and from H_3-30 to C-7/C-8/C-9/C-14 built up the planar structure of **10** (Supplementary Table 7), in which the assignments of C-26 and C-27 were determined on the basis of comparison of chemical shifts with those of C-26 and C-27 in **1** obtained in pyridine-d_5[9], and the NOESY correlation between H-24 and H_3-26. On the basis of the NOESY correlations between Ha-2 and H_3-19, between H-3 and H-5/H_3-28, between H_3-30 and H-5/H-13, and between H-9 and H_3-18 (Supplementary Table 7), the relative configurations of C-3, C-4, C-5, C-8, C-9, C-10, C-13, and C-14 were assigned as 3*S**, 4*R**,

5*R**, 8*S**, 9*S**, 10*R**, 13*R**, and 14*S**. Considering that **10** was biosynthesized from the precursor **2**, the absolute configuration of C-10 in **10** was assigned as *R*. Thus, the absolute configurations of C-3, C-4, C-5, C-8, C-9, C-10, C-13, and C-14 in **10** were assigned as 3*S*, 4*R*, 5*R*, 8*S*, 9*S*, 10*R*, 13*R*, and 14*S*, with the absolute configuration of C-20 unsolved. Therefore, **10** was identified as (3*S*,4*R*,5*R*,8*S*,9*S*,10*R*,13*R*,14*S*)-3-hydroxy-17-(2-hydroxy-6-methylhept-5-en-2-yl)-4,8,10,14-tetramethyl-2,3,4,5,6,7,8,9,10,11,12,13,14,15-tetradecahydro-1*H*-cyclopenta[*a*]phenanthrene-4-carboxylic acid, and named 3*β*,20-dihydroxy-protosta-16,24-dien-29-oic acid.

Supplementary Note 11. Structural characterization of compound 11

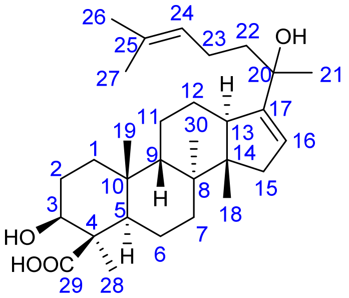

11

Compound **11** was obtained as a white powder. The quasi-molecular ion at *m/z* 473.3611 $[M + H]^+$ (calcd for $C_{30}H_{49}O_4$, 473.3631) by HRESIMS indicated that the molecular formula of **11** was $C_{30}H_{48}O_4$ (7 degrees of unsaturation). Combined with the DEPT-135 experiment, the ^{13}C NMR spectrum of **11** showed 30 carbons, assigned to one carbonyl carbon, four olefinic carbons (including two sp^2 methine carbons), five sp^3 quaternary carbons (including one oxygenated carbon), four sp^3 methine carbons (including one oxygenated carbon), nine sp^3 methylene carbons, and seven sp^3 methyl carbons. In the ^1H NMR spectrum of **11**, the characteristic signals of seven sets of methyl protons [δ_H 1.73 (3H, s), 1.70 (3H, br s), 1.66 (3H, br s), 1.56 (3H, s), 1.23 (3H, s), 1.20 (3H, s), 1.15 (3H, s)] were observed. The nonexchangeable proton resonances were associated with the directly attached carbon atoms in the HSQC experiment (Supplementary Table 8). The NMR data of **11** were similar to those of **10**, which revealed that the planar structure of **11** was the same as that of **10**. Combined with molecular formula and degrees of unsaturation, further detailed NMR analyses of ^1H-^1H COSY and HMBC spectra established the planar structure of **11**, in which the assignments of C-26 and C-27 were determined on the basis of comparison of chemical shifts with those of C-26 and C-27 in **10**, and the NOESY correlation between H-24 and H_3-26. On the basis of the NOESY correlations between Ha-2 and H_3-19, between H-3 and H-5/H_3-28, between H_3-30 and H-5/H-13, and between H-9 and H_3-18 (Supplementary Table 8), the relative configurations of C-3, C-4, C-5, C-8, C-9, C-10, C-13, and C-14 were assigned as 3*S**, 4*R**, 5*R**, 8*S**, 9*S**, 10*R**, 13*R**, and 14*S**. Considering that **11** was biosynthesized from the precursor **2**, the absolute configuration of C-10 in **11** was assigned as *R*. Thus, the absolute configurations of C-3, C-4, C-5, C-8, C-9, C-10, C-13, and C-14 in **11** were assigned as 3*S*,

4*R*, 5*R*, 8*S*, 9*S*, 10*R*, 13*R*, and 14*S*, with the absolute configuration of C-20 unsolved. Therefore, **11** was identified as (3*S*,4*R*,5*R*,8*S*,9*S*,10*R*,13*R*,14*S*)-3-hydroxy-17-(2-hydroxy-6-methylhept-5-en-2-yl)-4,8,10,14-tetramethyl-2,3,4,5,6,7,8,9,10,11,12,13,14,15-tetradecahydro-1*H*-cyclopenta[*a*]phenanthrene-4-carboxylic acid, and named 3*β*,20-dihydroxy-protosta-16,24-dien-29-oic acid.

Supplementary Note 12. Structural characterization of compound 12

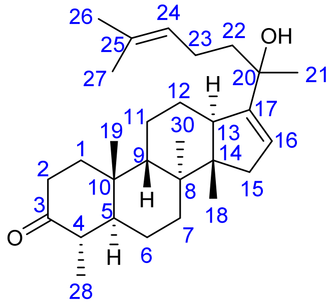

12

Compound **12** was obtained as a yellowish powder. The quasi-molecular ion at *m/z* 427.3569 $[M + H]^+$ (calcd for $C_{29}H_{47}O_2$, 427.3576) by HRESIMS indicated that the molecular formula of **12** was $C_{29}H_{46}O_2$ (7 degrees of unsaturation). Combined with the DEPT-135 experiment, the ^{13}C NMR spectrum of **12** showed 29 carbons, assigned to one carbonyl carbon, four olefinic carbons (including two sp^2 methine carbons), four sp^3 quaternary carbons (including one oxygenated carbon), four sp^3 methine carbons, nine sp^3 methylene carbons, and seven sp^3 methyl carbons. In the ^1H NMR spectrum of **12**, the characteristic signals of seven sets of methyl protons [δ_H 1.72 (3H, br s), 1.66 (3H, br s), 1.58 (3H, s), 1.12 (3H, s), 1.11 (3H, d, 5.4), 1.09 (3H, s), 0.95 (3H, s)] were observed. The nonexchangeable proton resonances were associated with the directly attached carbon atoms in the HSQC experiment (Supplementary Table 9). The analyses of the ^1H-^1H COSY and ^1H NMR spectra of **12** revealed the presence of some spin systems: C-1–C-2, C-28–C-4–C-5–C-6–C-7, C-9–C-11–C-12–C-13, C-15–C-16, C-22–C-23–C-24. Combined with the ^1H-^1H COSY data, molecular formula and degrees of unsaturation, the HMBC correlations from Ha-1/Hb-1/Ha-2/Hb-2 to C-3, from H-13 to C-17, from Ha-15/Hb-15 to C-16/C-17, from H-16 to C-14, from H_3-18 to C-8/C-13/C-14/C-15, from H_3-19 to C-1/C-5/C-9/C-10, from H_3-21 to C-17/C-20/C-22, from H_3-26 to C-24/C-25/C-27, from H_3-27 to C-24/C-25/C-26, from H_3-28 to C-3/C-4/C-5, from H_3-30 to C-7/C-8/C-9/C-14, and from 20-O<u>H</u> to C-17/C-20/C-21/C-22 built up the planar structure of **12** (Supplementary Table 9), in which the assignments of C-26 and C-27 were determined on the basis of comparison of chemical shifts with those of C-26 and C-27 in **1** obtained in pyridine-d_5[9], and the ROESY correlation between H-24 and H_3-26. On the basis of the coupling constant between H-4 and H-5 ($^3J_{H\text{-}4,}$

$_{H-5}$ = 12.0 Hz), and the ROESY correlations between H-4 and H_3-19, between H_3-30 and H-5/H-13, and between H-9 and H_3-18 (Supplementary Table 9), the relative configurations of C-4, C-5, C-8, C-9, C-10, C-13, and C-14 in **12** were assigned as 4*S**, 5*S**, 8*S**, 9*S**, 10*S**, 13*R**, and 14*S**. Considering that **12** was biosynthesized from the precursor **2**, the absolute configuration of C-10 in **12** was assigned as *S*. Thus, the absolute configurations of C-4, C-5, C-8, C-9, C-10, C-13, and C-14 in **12** were assigned as 4*S*, 5*S*, 8*S*, 9*S*, 10*S*, 13*R*, and 14*S*, with the absolute configuration of C-20 unsolved. Therefore, **12** was identified as (4*S*,5*S*,8*S*,9*S*,10*S*,13*R*,14*S*)-17-(2-hydroxy-6-methylhept-5-en-2-yl)-4,8,10,14-tetramethyl-1,2,4,5,6,7,8,9,10,11,12,13,14,15-tetradecahydro-3*H*-cyclopenta[*a*]phenanthren-3-one, and named 20-hydroxy-29-norprotosta-16,24-dien-3-one.

Supplementary Note 13. Structural characterization of compound 13

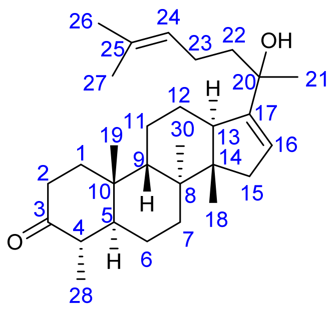

13

Compound **13** was obtained as a yellowish powder. The quasi-molecular ion at *m/z* 427.3568 $[M + H]^+$ (calcd for $C_{29}H_{47}O_2$, 427.3576) by HRESIMS indicated that the molecular formula of **13** was $C_{29}H_{46}O_2$ (7 degrees of unsaturation). Combined with the DEPT-135 experiment, the ^{13}C NMR spectrum of **13** showed 29 carbons, assigned to one carbonyl carbon, four olefinic carbons (including two sp^2 methine carbons), four sp^3 quaternary carbons (including one oxygenated carbon), four sp^3 methine carbons, nine sp^3 methylene carbons, and seven sp^3 methyl carbons. In the ^1H NMR spectrum of **13**, the characteristic signals of seven sets of methyl protons [δ_H 1.72 (3H, br s), 1.67 (3H, br s), 1.56 (3H, s), 1.11 (3H, d, 6.6), 1.08 (3H, s), 1.08 (3H, s), 0.96 (3H, s)] were observed. The nonexchangeable proton resonances were associated with the directly attached carbon atoms in the HSQC experiment (Supplementary Table 10). The NMR data of **13** were similar to those of **12**, which revealed that the planar structure of **13** was the same as that of **12**. Combined with molecular formula and degrees of unsaturation, further detailed NMR analyses of ^1H-^1H COSY and HMBC spectra established the planar structure of **13**, in which the assignments of C-26 and C-27 were determined on the basis of comparison of chemical shifts with those of C-26 and C-27 in **12**, and the ROESY correlation between H-24 and H_3-26. On the basis of the coupling constant between H-4 and H-5 ($^3J_{H\text{-}4,\,H\text{-}5}$ = 12.0 Hz), and the ROESY correlations between H-4 and H_3-19, between H_3-30 and H-5/H-13, and between H-9 and H_3-18 (Supplementary Table 10), the relative configurations of C-4, C-5, C-8, C-9, C-10, C-13, and C-14 were assigned as 4*S**, 5*S**, 8*S**, 9*S**, 10*S**, 13*R**, and 14*S**. Considering that **13** was biosynthesized from the precursor **2**, the absolute configuration of C-10 in **13** was assigned as *S*. Thus, the absolute configurations of C-4, C-5, C-8, C-9, C-10, C-13, and C-14 in **13** were

assigned as 4*S*, 5*S*, 8*S*, 9*S*, 10*S*, 13*R*, and 14*S*, with the absolute configuration of C-20 unsolved. Therefore, **13** was identified as (4*S*,5*S*,8*S*,9*S*,10*S*,13*R*,14*S*)-17-(2-hydroxy-6-methylhept-5-en-2-yl)-4,8,10,14-tetramethyl-1,2,4,5,6,7,8,9,10,11,12,13,14,15-tetradecahydro-3*H*-cyclopenta[*a*]phenanthren-3-one, and named 20-hydroxy-29-norprotosta-16,24-dien-3-one.

Supplementary Note 14. Structural characterization of compound 14

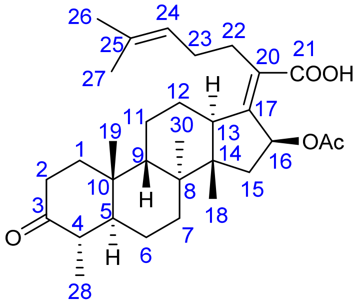

14

Compound **14** was obtained as a yellowish powder. The quasi-molecular ion at *m/z* 521.3244 $[M + Na]^+$ (calcd for $C_{31}H_{46}O_5Na$, 521.3243) by HRESIMS indicated that the molecular formula of **14** was $C_{31}H_{46}O_5$ (9 degrees of unsaturation). Combined with the DEPT-135 experiment, the ^{13}C NMR spectrum of **14** showed 31 carbons, assigned to three carbonyl carbons, four olefinic carbons (including one sp^2 methine carbon), three sp^3 quaternary carbons, five sp^3 methine carbons (including one oxygenated carbon), nine sp^3 methylene carbons, and seven sp^3 methyl carbons. In the ^1H NMR spectrum of **14**, the characteristic signals of seven sets of methyl protons [δ_H 1.94 (3H, s), 1.66 (3H, br s), 1.58 (3H, br s), 1.09 (3H, s), 1.01 (3H, d, 6.0), 1.01 (3H, s), 0.95 (3H, s)] were observed. The nonexchangeable proton resonances were associated with the directly attached carbon atoms in the HSQC experiment (Supplementary Table 11). Except for the loss of one methyl carbon (δ_C 21.4) and the appearance of one carbonyl carbon (δ_C 174.8), the NMR data of **14** were similar to those of **9**, which revealed that **14** may be an oxygenated derivative of **9**. Combined with molecular formula and degrees of unsaturation, further detailed NMR analyses of ^1H-^1H COSY and HMBC spectra established the planar structure of **14**, in which the assignments of C-26 and C-27 were determined on the basis of comparison of chemical shifts with those of C-26 and C-27 in **9**. On the basis of the coupling constant between H-4 and H-5 ($^3J_{H\text{-}4,\, H\text{-}5}$ = 13.2 Hz), and the ROESY correlations between H-4 and H_3-19, between H_3-30 and H-5/H-13, between H-9 and H_3-18, and between H-13 and H-16 (Supplementary Table 11), the relative configuration of **14** was determined as 4*S**, 5*S**, 8*S**, 9*S**, 10*S**, 13*R**, 14*S**, 16*S**. Considering that **14** was biosynthesized from the precursor **2**, the geometric configuration of the double bond between C-17 and C-20 was determined as *Z*, and the absolute configuration

of C-10 in **14** was assigned as *S*. Thus, the absolute configuration of **14** was assigned as 4*S*, 5*S*, 8*S*, 9*S*, 10*S*, 13*R*, 14*S*, 16*S*. Therefore, **14** was identified as (*Z*)-2-((4*S*,5*S*,8*S*,9*S*,10*S*,13*R*,14*S*,16*S*)-16-acetoxy-4,8,10,14-tetramethyl-3-oxohexadecahydro-17*H*-cyclopenta[*a*]phenanthren-17-ylidene)-6-methylhept-5-enoic acid, and named 16*β*-acetyloxy-29-norprotosta-17(20)*Z*,24-dien-3-one-21-oic acid.

Supplementary Note 15. Structural characterization of compound 15

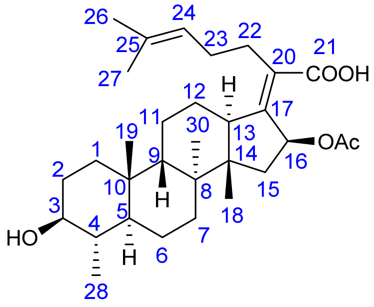

15

Compound **15** was obtained as a yellowish powder. The quasi-molecular ion at *m/z* 523.3408 $[M + Na]^+$ (calcd for $C_{31}H_{48}O_5Na$, 523.3399) by HRESIMS indicated that the molecular formula of **15** was $C_{31}H_{48}O_5$ (8 degrees of unsaturation). Combined with the DEPT-135 experiment, the ^{13}C NMR spectrum of **15** showed 31 carbons, assigned to two carbonyl carbons, four olefinic carbons (including one sp^2 methine carbon), three sp^3 quaternary carbons, six sp^3 methine carbons (including two oxygenated carbons), nine sp^3 methylene carbons, and seven sp^3 methyl carbons. In the ^1H NMR spectrum of **15**, the characteristic signals of seven sets of methyl protons [δ_H 1.95 (3H, s), 1.67 (3H, br s), 1.58 (3H, br s), 1.08 (3H, s), 0.94 (3H, d, 6.4), 0.92 (3H, s), 0.91 (3H, s)] were observed. The nonexchangeable proton resonances were associated with the directly attached carbon atoms in the HSQC experiment (Supplementary Table 12). Except for the loss of one carbonyl carbon (δ_C 214.8) and the appearance of one oxygenated methine carbon (δ_C 77.3), the NMR data of **15** were similar to those of **14**, which revealed that **15** might be the 3-keto reduced form of **14**. Combined with molecular formula and degrees of unsaturation, further detailed NMR analyses of ^1H-^1H COSY and HMBC spectra established the planar structure of **15**, in which the assignments of C-26 and C-27 were determined on the basis of comparison of chemical shifts with those of C-26 and C-27 in **14**, and the NOESY correlation between H-24 and H_3-26. On the basis of the NOESY correlations between H-3 and H-5/H_3-28, between H-4 and H_3-19, between H_3-30 and H-5/H-13, between H-9 and H_3-18, and between H-13 and H-16 (Supplementary Table 12), the relative configuration of **15** was assigned as $3S^*,4S^*, 5S^*, 8S^*, 9S^*, 10S^*, 13R^*, 14S^*, 16S^*$. Considering that **15** was biosynthesized from the precursor **2**, the geometric configuration of the double bond between C-17 and C-20 was determined as *Z*,

and the absolute configuration of C-10 in **15** was assigned as *S*. Thus, the absolute configuration of **15** was assigned as 3*S*, 4*S*, 5*S*, 8*S*, 9*S*, 10*S*, 13*R*, 14*S*, 16*S*. Therefore, **15** was identified as (*Z*)-2-((3*S*,4*S*,5*S*,8*S*,9*S*,10*S*,13*R*,14*S*,16*S*)-16-acetoxy-3-hydroxy-4,8,10,14-tetramethylhexadecahydro-17*H*-cyclopenta[*a*]phenanthren-17-ylidene)-6-methylhept-5-enoic acid, and named 16*β*-acetyloxy-3*β*-hydroxy-29-norprotosta-17(20)*Z*,24-dien-21-oic acid.

Supplementary Note 16. Structural characterization of compound 16

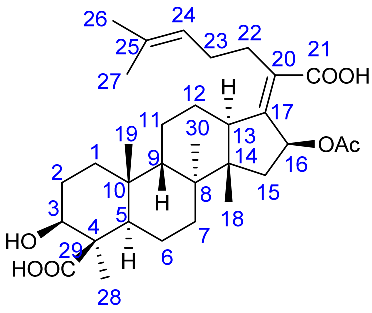

16

Compound **16** was obtained as a yellowish powder. The quasi-molecular ion at *m/z* 545.3474 $[M + H]^+$ (calcd for $C_{32}H_{49}O_7$, 545.3478) by HRESIMS indicated that the molecular formula of **16** was $C_{32}H_{48}O_7$ (9 degrees of unsaturation). Combined with the DEPT-135 experiment, the ^{13}C NMR spectrum of **16** showed 32 carbons, assigned to three carbonyl carbons, four olefinic carbons (including one sp^2 methine carbon), four sp^3 quaternary carbons, five sp^3 methine carbons (including two oxygenated carbons), nine sp^3 methylene carbons, and seven sp^3 methyl carbons. In the ^1H NMR spectrum of **16**, the characteristic signals of seven sets of methyl protons [δ_H 1.96 (3H, s), 1.71 (3H, s), 1.70 (3H, br s), 1.67 (3H, br s), 1.21 (3H, s), 1.13 (3H, s), 1.11 (3H, s)] were observed. The nonexchangeable proton resonances were associated with the directly attached carbon atoms in the HSQC experiment (Supplementary Table 13). The analyses of the ^1H-^1H COSY and ^1H NMR spectra of **16** revealed the presence of some spin systems: C-1–C-2–C-3, C-5–C-6–C-7, C-9–C-11–C-12–C-13, C-15–C-16, C-22–C-23–C-24. Combined with the ^1H-^1H COSY data, molecular formula and degrees of unsaturation, the HMBC correlations from H-13 to C-17/C-20, from Hb-15 to C-17, from H-16 to C-14/C-17/C-20/16-$\underline{C}OCH_3$, from H_3-18 to C-8/C-13/C-14/C-15, from H_3-19 to C-1/C-5/C-9/C-10, from Ha-22/Hb-22 to C-17/C-20/C-21, from H_3-26 to C-24/C-25/C-27, from H_3-27 to C-24/C-25/C-26, from H_3-28 to C-3/C-4/C-5/C-29, from H_3-30 to C-7/C-8/C-9/C-14, and from 16-$COC\underline{H}_3$ to 16-$\underline{C}OCH_3$ built up the planar structure of **16** (Supplementary Table 13), in which the assignments of C-26 and C-27 were determined on the basis of comparison of chemical shifts with those of C-26 and C-27 in **1** obtained in pyridine-d_5[9], and the ROESY correlation between H-24 and H_3-26. On the basis of the ROESY correlations between Ha-2 and H_3-19, between H-3 and

H-5/H_3-28, between H_3-30 and H-5/H-13, between H-9 and H_3-18, and between H-13 and H-16 (Supplementary Table 13), the relative configuration of **16** was determined as 3*S**, 4*R**, 5*R**, 8*S**, 9*S**, 10*R**, 13*R**, 14*S**, 16*S**. Considering that **16** was biosynthesized from the precursor **2**, the geometric configuration of the double bond between C-17 and C-20 was determined as *Z*, and the absolute configuration of C-10 in **16** was assigned as *R*. Thus, the absolute configuration of **16** was assigned as 3*S*, 4*R*, 5*R*, 8*S*, 9*S*, 10*R*, 13*R*, 14*S*, 16*S*. Therefore, **16** was indentified as (3*S*,4*R*,5*R*,8*S*,9*S*,10*R*,13*R*,14*S*,16*S*,*Z*)-16-acetoxy-17-(1-carboxy-5-methylhex-4-en-1-ylidene) -3-hydroxy-4,8,10,14-tetramethylhexadecahydro-1*H*-cyclopenta[*a*]phenanthrene-4-carboxylic acid, and named 16*β*-acetyloxy-3*β*-hydroxy-protosta-17(20)*Z*,24-dien-21,29-dioic acid.

Supplementary Note 17. Structural characterization of compound 17

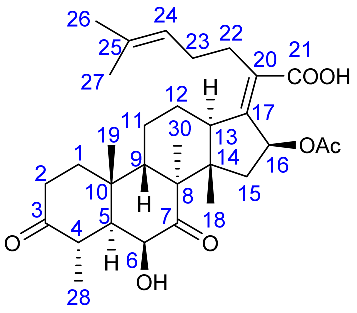

17

Compound **17** was obtained as a yellowish powder. The quasi-molecular ion at *m/z* 551.2987 $[M + Na]^+$ (calcd for $C_{31}H_{44}O_7Na$, 551.2985) by HRESIMS indicated that the molecular formula of **17** was $C_{31}H_{44}O_7$ (10 degrees of unsaturation). Combined with the DEPT-135 experiment, the ^{13}C NMR spectrum of **17** showed 31 carbons, assigned to four carbonyl carbons, four olefinic carbons (including one sp^2 methine carbon), three sp^3 quaternary carbons, six sp^3 methine carbons (including two oxygenated carbons), seven sp^3 methylene carbons, and seven sp^3 methyl carbons. In the ^1H NMR spectrum of **17**, the characteristic signals of seven sets of methyl protons [δ_H 1.94 (3H, s), 1.67 (3H, br s), 1.58 (3H, br s), 1.40 (3H, s), 1.23 (3H, s), 1.09 (3H, d, 6.8), 0.93 (3H, s)] were observed. The nonexchangeable proton resonances were associated with the directly attached carbon atoms in the HSQC experiment (Supplementary Table 14). Except for the loss of two methylene carbons (δ_C 32.8, 21.0) and the appearance of one carbonyl carbon (δ_C 216.2) and one oxygenated methine carbon (δ_C 73.5), the NMR data of **17** were similar to those of **14**, which revealed that **17** might be an oxygenated derivative of **14**. Combined with molecular formula and degrees of unsaturation, further detailed NMR analyses of ^1H-^1H COSY and HMBC spectra established the planar structure of **17**, in which the assignments of C-26 and C-27 were determined on the basis of comparison of chemical shifts with those of C-26 and C-27 in **14**, and the NOESY correlation between H-24 and H_3-26. On the basis of the coupling constant between H-4 and H-5 ($^3J_{H\text{-}4,\, H\text{-}5}$ = 13.0 Hz), and the NOESY correlations between H-4 and H_3-19, between H_3-30 and H-5/H-13, between H-9 and H_3-18, and between H-13 and H-16 (Supplementary Table 14), the relative configurations of C-4, C-5, C-8, C-9, C-10, C-13, C-14, and C-16 in **17** were determined as 4*S**, 5*S**, 8*S**, 9*S**, 10*R**, 13*R**, 14*S**, and 16*S**.

Because $^{3}J_{H\text{-}5, H\text{-}6}$ in **17** was as small as that in **1** (Supplementary Table 14), indicating that H-5 and H-6 in **17** were also on the same side of B-ring, the relative configuration of **17** was assigned as 4*S**, 5*S**, 6*S**, 8*S**, 9*S**, 10*R**, 13*R**, 14*S**, 16*S**. Considering that **17** was biosynthesized from the precursor **2**, the geometric configuration of the double bond between C-17 and C-20 was determined as *Z*, and the absolute configuration of C-10 in **17** was assigned as *R*. Thus, the absolute configuration of **17** was assigned as 4*S*, 5*S*, 6*S*, 8*S*, 9*S*, 10*R*, 13*R*, 14*S*, 16*S*. Based on the above analyses, the structure of **17** was the same as that of 16*β*-acetyloxy-6*β*-hydroxy-29-norprotosta-17(20)*Z*,24-dien-3,7-dione-21-oic acid (CAS: 1379525-35-5), the detailed NMR assignments of which were not available prior to the present study.

Supplementary Note 18. Structural characterization of compound 18

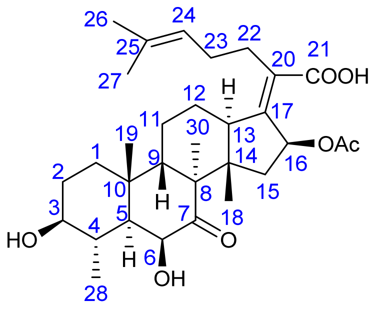

18

Compound **18** was obtained as a yellowish powder. The quasi-molecular ion at *m/z* 553.3139 $[M + Na]^+$ (calcd for $C_{31}H_{46}O_7Na$, 553.3141) by HRESIMS indicated that the molecular formula of **18** was $C_{31}H_{46}O_7$ (9 degrees of unsaturation). Combined with the DEPT-135 experiment, the ^{13}C NMR spectrum of **18** showed 31 carbons, assigned to three carbonyl carbons, four olefinic carbons (including one sp^2 methine carbon), three sp^3 quaternary carbons, seven sp^3 methine carbons (including three oxygenated carbons), seven sp^3 methylene carbons, and seven sp^3 methyl carbons. In the ^1H NMR spectrum of **18**, the characteristic signals of seven sets of methyl protons [δ_H 1.83 (3H, s), 1.71 (3H, br s), 1.68 (3H, br s), 1.66 (3H, s), 1.39 (3H, s), 1.36 (3H, s), 1.33 (3H, d, 6.4)] were observed. The nonexchangeable proton resonances were associated with the directly attached carbon atoms in the HSQC experiment (Supplementary Table 15). The analyses of the ^1H-^1H COSY and ^1H NMR spectra of **18** revealed the presence of some spin systems: C-1–C-2–C-3–C-4(–C-28)–C-5–C-6, C-9–C-11–C-12–C-13, C-15–C-16, C-22–C-23–C-24. Combined with the ^1H-^1H COSY data, molecular formula and degrees of unsaturation, the HMBC correlations from H-6 to C7/C8, from H-13 to C-17/C-20, from Hb-15 to C-17, from H-16 to C-14/C-17/C-20/16-$\underline{C}OCH_3$, from H_3-18 to C-8/C-13/C-14/C-15, from H_3-19 to C-1/C-5/C-9/C-10, from H_2-22 to C-20/C-21, from H_3-26 to C-24/C-25/C-27, from H_3-27 to C-24/C-25/C-26, from H_3-28 to C-3/C-4/C-5, from H_3-30 to C-7/C-8/C-9/C-14, and from 16-$COC\underline{H}_3$ to 16-$\underline{C}OCH_3$ built up the planar structure of **18** (Supplementary Table 15), in which the assignments of C-26 and C-27 were determined on the basis of comparison of chemical shifts with those of C-26 and C-27 in **1** obtained in pyridine-d_5[9], and the ROESY correlation between H-24 and H_3-26. On the basis of the ROESY correlations between H-3

and H-5/H_3-28, between H-4 and H_3-19, between H_3-30 and H-5/H-13, between H-9 and H_3-18, and between H-13 and H-16 (Supplementary Table 15), the relative configurations of C-3, C-4, C-5, C-8, C-9, C-10, C-13, C-14, and C-16 in **18** were determined as 3*S**, 4*S**, 5*S**, 8*S**, 9*S**, 10*R**, 13*R**, 14*S**, and 16*S**. Because $^3J_{\text{H-5, H-6}}$ in **18** was as small as that in **1** (Supplementary Table 15), indicating H-5 and H-6 in **18** were also on the same side of B-ring, the relative configuration of **18** was assigned as 3*S**, 4*S**, 5*S**, 6*S**, 8*S**, 9*S**, 10*R**, 13*R**, 14*S**, 16*S**. Considering that **18** was biosynthesized from the precursor **2**, the geometric configuration of the double bond between C-17 and C-20 was determined as *Z*, and the absolute configuration of C-10 in **18** was assigned as *R*. Thus, the absolute configuration of **18** was assigned as 3*S*, 4*S*, 5*S*, 6*S*, 8*S*, 9*S*, 10*R*, 13*R*, 14*S*, 16*S*. Therefore, **18** was identified as (*Z*)-2-((3*S*,4*S*,5*S*,6*S*,8*S*,9*S*,10*R*,13*R*,14*S*,16*S*)-16-acetoxy-3,6-dihydroxy-4,8,10,14-tetramethyl-7-oxohexadecahydro-17*H*-cyclopenta[*a*]phenanthren-17-ylidene)-6-methylhept-5-enoic acid, and named 16*β*-acetyloxy-3*β*,6*β*-dihydroxy-29-norprotosta-17(20)*Z*,24-dien-7-one-21-oic acid.

Supplementary Note 19. Structural characterization of compound 19

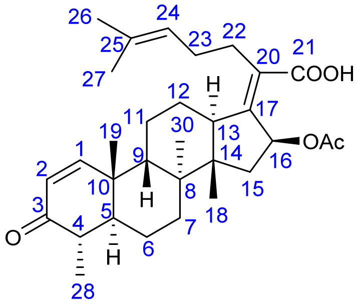

19

Compound **19** was obtained as a white powder. The quasi-molecular ion at *m/z* 497.3238 $[M + H]^+$ (calcd for $C_{31}H_{45}O_5$, 497.3267) indicated that the molecular formula of **19** was $C_{31}H_{44}O_5$ (10 degrees of unsaturation). Combined with the DEPT-135 experiment, the ^{13}C NMR spectrum of **19** showed 31 carbons, assigned to three carbonyl carbons, six olefinic carbons (including three sp^2 methine carbons), three sp^3 quaternary carbons, five sp^3 methine carbons (including one oxygenated carbon), seven sp^3 methylene carbons, and seven sp^3 methyl carbons. In the ^1H NMR spectrum of **19**, the characteristic signals of seven sets of methyl protons [δ_H 1.95 (3H, s), 1.68 (3H, br s), 1.59 (3H, br s), 1.15 (3H, s), 1.13 (3H, d, 6.8), 0.99 (3H, s), 0.97 (3H, s)] were observed. The nonexchangeable proton resonances were associated with the directly attached carbon atoms in the HSQC experiment (Supplementary Table 16). Except for the loss of two methylene carbons (δ_C 37.3, 32.6) and the appearance of two sp^2 methine carbons (δ_C 159.4, 127.7), the NMR data of **19** were similar to those of **14**, which revealed that **19** might be a dehydrogenated derivative of **14**. Combined with molecular formula and degrees of unsaturation, further detailed NMR analyses of ^1H-^1H COSY and HMBC spectra established the planar structure of **19**, in which the assignments of C-26 and C-27 were determined on the basis of comparison of chemical shifts with those of C-26 and C-27 in **14**, and the ROESY correlation between H-24 and H_3-26. In view of the fact that **19** was biosynthesized from the precursor **2**, A-ring and B-ring were *in trans* fused, and H-5 and C-19 were on the opposite side of A-ring. On the basis of the ROESY correlations between H-4 and H_3-19, between H_3-30 and H-5/H-13, between H-9 and H_3-18, and between H-13 and H-16 (Supplementary Table 16), the relative configuration of **19** was determined as 4*S**, 5*S**, 8*S**, 9*S**, 10*S**, 13*R**, 14*S**, 16*S**. In addition, the geometric configuration of the double

bond between C-17 and C-20 was determined as *Z*, and the absolute configuration of C-10 in **19** was assigned as *S*. Thus, the absolute configuration of **19** was assigned as 4*S*, 5*S*, 8*S*, 9*S*, 10*S*, 13*R*, 14*S*, 16*S*. Based on the above analyses, the structure of **19** was the same as that of 16*β*-acetyloxy-29-norprotosta-1,17(20)*Z*,24-trien-3-one-21-oic acid [3-oxo-16*β*-acetoxyfusida-1,17(20)(16,21-cis),24-trien-21-oic acid[10]], the detailed NMR assignments of which were not available prior to the present study.

Supplementary Note 20. Structural characterization of compound 20

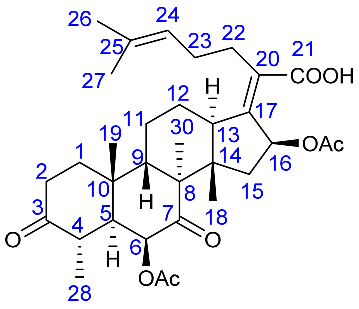

20

Compound **20** was obtained as a white powder. The quasi-molecular ion at *m/z* 593.3081 $[M + Na]^+$ (calcd for $C_{33}H_{46}O_8Na$, 593.3090) by HRESIMS indicated that the molecular formula of **20** was $C_{33}H_{46}O_8$ (11 degrees of unsaturation). ^1H NMR (400 MHz, pyridine-d_5) δ_H 6.46 (br d, J = 8.0 Hz, 1H), 5.59 (br s, 1H), 5.40 (br t, J = 6.0 Hz, 1H), 2.89 (m, 2H), 2.67 (m, 1H), 2.67 (m, 1H), 2.64 (m, 1H), 2.58 (m, 2H), 2.56 (m, 1H), 2.48 (m, 1H), 2.45 (m, 1H), 2.44 (m, 1H), 2.31 (br d, J = 12.4 Hz, 1H), 2.18 (br d, J = 14.8 Hz, 1H), 2.12 (s, 3H), 1.93 (s, 3H), 1.88 (m, 1H), 1.83 (m, 1H), 1.77 (m, 1H), 1.72 (br s, 3H), 1.69 (br s, 3H), 1.52 (m, 1H), 1.38 (m, 1H), 1.32 (s, 3H), 1.28 (s, 3H), 1.24 (d, J = 6.8 Hz, 3H), 1.14 (s, 3H); ^{13}C NMR (100 MHz, pyridine-d_5) δ_C 211.8, 209.9, 173.0, 170.4, 169.4, 144.5, 133.2, 132.3, 124.3, 74.2, 74.1, 53.2, 48.9, 47.4, 45.7, 42.2, 41.6, 41.3, 37.1, 35.4, 33.1, 29.5, 29.1, 26.5, 25.8, 23.6, 22.9, 20.6, 20.6, 18.3, 17.9, 17.4, 13.0. The NMR data were in good agreement with those of 6β,16β-diacetyloxy-29-norprotosta-17(20)*Z*,24-dien-3,7-dione-21-oic acid (1,2-dihydrohelvolic acid[9]).

Supplementary Note 21. Structural characterization of compound 21

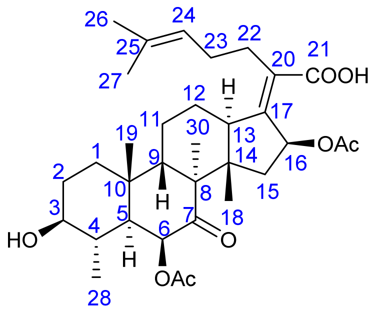

21

Compound **21** was obtained as a white powder. The quasi-molecular ion at *m/z* 573.3428 $[M + H]^+$ (calcd for $C_{33}H_{49}O_8$, 573.3427) by HRESIMS indicated that the molecular formula of **21** was $C_{33}H_{48}O_8$ (10 degrees of unsaturation). Combined with the DEPT-135 experiment, the ^{13}C NMR spectrum of **21** showed 31 carbons, assigned to four carbonyl carbons, four olefinic carbons (including one sp^2 methine carbon), three sp^3 quaternary carbons, seven sp^3 methine carbons (including three oxygenated carbons), seven sp^3 methylene carbons, and eight sp^3 methyl carbons. In the ^1H NMR spectrum of **21**, the characteristic signals of eight sets of methyl protons [δ_H 2.06 (3H, s), 1.93 (3H, s), 1.68 (3H, br s), 1.59 (3H, br s), 1.28 (3H, s), 1.25 (3H, s), 1.08 (3H, d, 6.0), 0.89 (3H, s)] were observed. The nonexchangeable proton resonances were associated with the directly attached carbon atoms in the HSQC experiment (Supplementary Table 17). The analyses of the ^1H-^1H COSY and ^1H NMR spectra of **21** revealed the presence of some spin systems: C-1–C-2–C-3–C-4(–C-28)–C-5–C-6, C-9–C-11–C-12–C-13, C-15–C-16, C-22–C-23–C-24. Combined with the ^1H-^1H COSY data, molecular formula and degrees of unsaturation, the HMBC correlations from H-6 to C7/C8/6-$\underline{C}OCH_3$, from H-13 to C-17/C-20, from Hb-15 to C-17, from H-16 to C-14/C-17/C-20/16-$\underline{C}OCH_3$, from H_3-18 to C-8/C-13/C-14/C-15, from H_3-19 to C-1/C-5/C-9/C-10, from Ha-22/Hb-22 to C-17/C-20/C-21, from H_3-26 to C-24/C-25/C-27, from H_3-27 to C-24/C-25/C-26, from H_3-28 to C-3/C-4/C-5, from H_3-30 to C-7/C-8/C-9/C-14, from 6-$COC\underline{H}_3$ to 6-$\underline{C}OCH_3$, and from 16-$COC\underline{H}_3$ to 16-$\underline{C}OCH_3$ built up the planar structure of **21** (Supplementary Table 17), in which the assignments of C-26 and C-27 were determined on the basis of comparison of chemical shifts with those of C-26 and C-27 in **1**, and the ROESY correlation between H-24 and H_3-26. On the basis of the ROESY correlations

between H-3 and H-5/H_3-28, between H-4 and H_3-19, between H_3-30 and H-5/H-13, between H-9 and H_3-18, and between H-13 and H-16 (Supplementary Table 17), the relative configurations of C-3, C-4, C-5, C-8, C-9, C-10, C-13, C-14, and C-16 in **21** were determined as 3*S**, 4*S**, 5*S**, 8*S**, 9*S**, 10*R**, 13*R**, 14*S**, and 16*S**. Because $^3J_{\text{H-5, H-6}}$ in **21** was as small as that in **1** (Supplementary Table 17), indicating H-5 and H-6 in **21** were also on the same side of B-ring, the relative configuration of **21** was assigned as 3*S**, 4*S**, 5*S**, 6*S**, 8*S**, 9*S**, 10*R**, 13*R**, 14*S**, 16*S**. Considering that **21** was biosynthesized from the precursor **2**, the geometric configuration of the double bond between C-17 and C-20 was determined as *Z*, and the absolute configuration of C-10 in **21** was assigned as *R*. Thus, the absolute configuration of **21** was assigned as 3*S*, 4*S*, 5*S*, 6*S*, 8*S*, 9*S*, 10*R*, 13*R*, 14*S*, 16*S*. Therefore, **21** was identified as (*Z*)-2-((3*S*,4*S*,5*S*,6*S*,8*S*,9*S*,10*R*,13*R*,14*S*,16*S*)-6,16-diacetoxy-3-hydroxy-4,8,10,14-tetramethyl-7-oxohexadecahydro-17*H*-cyclopenta[*a*]phenanthren-17-ylidene)-6-methylhept-5-enoic acid, and named 6*β*,16*β*-diacetyloxy-3*β*-hydroxy-29-norprotosta-17(20)*Z*,24-dien-7-one-21-oic acid.

Supplementary Note 22. Structural characterization of compound 22

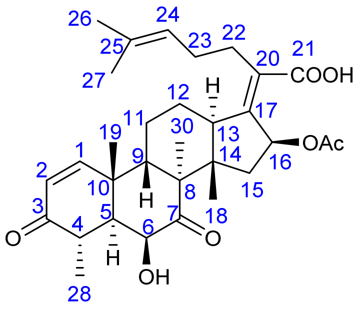

22

Compound **22** was obtained as a white powder. The quasi-molecular ion at *m/z* 527.3009 $[M + H]^+$ (calcd for $C_{31}H_{43}O_7$, 527.3009) by HRESIMS indicated that the molecular formula of **22** was $C_{31}H_{42}O_7$ (11 degrees of unsaturation). ^{1}H NMR (400 MHz, $CDCl_3$) δ_H 7.31 (d, J = 10.0 Hz, 1H), 5.86 (br d, J = 7.2 Hz, 1H), 5.84 (d, J = 10.0 Hz, 1H), 5.10 (br t, J = 7.2 Hz, 1H), 3.99 (br s, 1H), 3.04 (dq, J = 12.8, 6.4 Hz, 1H), 2.68 (dd, J = 13.2, 2.0 Hz, 1H), 2.56 (br d, J = 12.4 Hz, 1H), 2.47 (m, 2H), 2.40 (m, 1H), 2.22 (dd, J = 14.8, 7.2 Hz, 1H), 2.14 (br d, J = 12.8 Hz, 1H), 2.08 (m, 2H), 1.96 (s, 3H), 1.95 (m, 1H), 1.88 (br d, J = 14.8 Hz, 1H), 1.81 (m, 1H), 1.68 (br s, 3H), 1.60 (br s, 3H), 1.56 (m, 1H), 1.54 (s, 3H), 1.21 (d, J = 6.4 Hz, 3H), 1.12 (s, 3H), 0.95 (s, 3H); ^{13}C NMR (100 MHz, $CDCl_3$) δ_C 215.6, 202.2, 173.9, 170.7, 158.2, 148.1, 132.8, 130.6, 127.5, 122.8, 73.8, 73.8, 52.4, 49.6, 47.1, 46.5, 41.4, 40.8, 39.9, 38.2, 28.5, 28.4, 28.1, 25.9, 25.7, 24.0, 20.5, 18.2, 17.8, 17.7, 12.4. The NMR data were in good agreement with those of 16*β*-acetyloxy-6*β*-hydroxy-29-norprotosta-1,17(20)*Z*,24-trien-3,7-dione-21-oic acid (helvolinic acid[5]).

Supplementary Methods

Detailed purification procedure for each metabolite

Purification process for **1**

The mycelia and culture medium extract (2.9 g) from 2 L culture of *A. oryzae* NSAR1 harboring *helA*, *helB1*, *helC*, *helB2*, *helD2*, *helB4*, *helB3*, *helD1* and *helE* was subjected to silica gel column chromatography using petroleum ether and EtOAc (100:20 and 0:100, v/v) to afford 2 fractions. Fraction 2 (245.7 mg) was subjected to MPLC on ODS column chromatography with stepwise elution of MeOH and H_2O (5:5, 8.5:1.5 and 10:0, v/v) to yield 3 subfractions. Subfraction 2 (130.5 mg) was further purified by semi-preparative HPLC (YMC-Pack ODS-A column, 3 mL min^{-1}) with isocratic elution of 60% CH_3CN-H_2O containing 0.1% formic acid to yield **1** (t_R: 22.5 min, 19.5 mg).

Purification process for **2** and **3**

The mycelia extract (3.5 g) from 5 L culture of *A. oryzae* NSAR1 harboring *helA* and *helC* was subjected to silica gel column chromatography with stepwise elution of petroleum ether and EtOAc (100:1, 100:2, 100:3, 100:4, 100:5, 100:6 and 0:100, v/v) to yield 7 fractions. Fraction 3 (575.3 mg) was further purified by semi-preparative HPLC (YMC-Pack ODS-A column, 3 mL min^{-1}) with 100% CH_3CN to **3** (t_R: 43.0 min, 30.2 mg). Fraction 4 (152.4 mg) was further purified by semi-preparative HPLC (YMC-Pack ODS-A column, 3 mL min^{-1}) with 100% CH_3CN to yield **2** (t_R: 54.0 min, 21.3 mg).

Purification process for **4**

The mycelia extract (4.1 g) from 6 L culture of *A. oryzae* NSAR1 harboring *helA* and *helB1* was subjected to silica gel column chromatography with stepwise elution of petroleum ether and EtOAc (100:2, 100:4, 100:6, 100:8, 100:10, 100:12, 100:14, 100:16, 100:20 and 0:100, v/v) to yield 10 fractions. Fraction 7 (218.3 mg) was further purified by semi-preparative HPLC (YMC-Pack ODS-A column, 3 mL min^{-1}) with isocratic elution of 90% CH_3CN-H_2O containing 0.1% formic acid to yield **4** (t_R: 56.3 min, 53.7 mg).

Purification process for **5**

The mycelia extract (3.2 g) from 5 L culture of *A. oryzae* NSAR1 harboring *helA*, *helB1* and *helC* was subjected to silica gel column chromatography with stepwise elution of

petroleum ether and EtOAc (100:1, 100:2, 100:3, 100:4 and 0:100, v/v) to yield 5 fractions. Fraction 2 (51.7 mg) was further purified by semi-preparative HPLC (YMC-Pack ODS-A column, 3 mL min^{-1}) with 100% CH_3CN to yield **5** (t_R: 36.6 min, 16.2 mg).

Purification process for **6** and **7**

The mycelia extract (4.6 g) from 6 L culture of *A. oryzae* NSAR1 harboring *helA*, *helB1*, *helC* and *helB2* was subjected to silica gel column chromatography with stepwise elution of petroleum ether and EtOAc (100:2, 100:4, 100:6, 100:8, 100:10, 100:20 and 0:100, v/v) to yield 7 fractions. Fraction 4 (210.9 mg) was further purified by semi-preparative HPLC (YMC-Pack ODS-A column, 3 mL min^{-1}) with isocratic elution of 85% CH_3CN-H_2O to yield **7** (t_R: 31.5 min, 7.2 mg). Fraction 7 (713.4 mg) was subjected to MPLC on ODS column chromatography with stepwise elution of MeOH and H_2O (5:5, 9:1 and 10:0, v/v) to yield 3 subfractions. Subfraction 2 (150.8 mg) was further purified by semi-preparative HPLC (YMC-Pack ODS-A column, 3 mL min^{-1}) with isocratic elution of 60% CH_3CN-H_2O to yield **6** (t_R: 52.3 min, 10.1 mg).

Purification process for **8**, **9**, **10**, **11**, **12** and **13**

The mycelia extract (3.9 g) from 5 L culture of *A. oryzae* NSAR1 harboring *helA*, *helB1*, *helC*, *helB2* and *helD2* was subjected to silica gel column chromatography with stepwise elution of petroleum ether and EtOAc (100:4, 100:6, 100:10, 100:20, 100:30 and 0:100, v/v) to yield 6 fractions. Fraction 2 (119.0 mg) was further purified by semi-preparative HPLC (YMC-Pack ODS-A column, 3 mL min^{-1}) with isocratic elution of 85% CH_3CN-H_2O to yield **9** (t_R: 58.7 min, 9.5 mg). Fraction 4 (157.9 mg) was further purified by semi-preparative HPLC (YMC-Pack ODS-A column, 3 mL min^{-1}) with isocratic elution of 80% CH_3CN-H_2O to yield mixtures of **12** and **13** (t_R: 48.1 min, 14.2 mg). **12** (t_R: 33.1 min, 6.7 mg) and **13** (t_R: 36.2 min, 5.9 mg) were separated via the EnantioPakOX-352546 column (Guangzhou Research & Creativity Biotechnology Co., Ltd., Guangzhou, China) packed with cellulose tris(3-chloro-4-methylphenylcarbamate) coated silica gel (70% CH_3CN-H_2O, 1 mL min^{-1}). Fraction 5 (291.0 mg) was further purified by semi-preparative HPLC (YMC-Pack ODS-A column, 3 mL min^{-1}) with isocratic elution of 75% CH_3CN-H_2O containing 0.1% formic acid to yield **8** (t_R: 46.8 min, 15.1 mg). Fraction 6 (422.8 mg) was subjected to MPLC on ODS column chromatography with stepwise elution of MeOH and H_2O (5:5, 8:2 and 10:0, v/v) to

yield 3 subfractions. Subfraction 2 (60.0 mg) was further purified by semi-preparative HPLC (YMC-Pack ODS-A column, 3 mL min^{-1}) with isocratic elution of 60% CH_3CN-H_2O to yield **10** (t_R: 58.5 min, 8.1 mg) and **11** (t_R: 61.2 min, 13.5 mg).

Purification process for **14** and **15**

The mycelia and culture medium extract (2.5 g) from 3 L culture of *A. oryzae* NSAR1 harboring *helA*, *helB1*, *helC*, *helB2*, *helD2* and *helB4* was subjected to silica gel column chromatography with stepwise elution of petroleum ether and EtOAc (100:20 and 0:100, v/v) to yield 2 fractions. Fraction 2 (316.5 mg) was further subjected to MPLC on ODS column chromatography with stepwise elution of MeOH and H_2O (5:5, 8.5:1.5 and 10:0, v/v) to yield 3 subfractions. Subfraction 2 (183.2 mg) was further purified by semi-preparative HPLC (YMC-Pack ODS-A column, 3 mL min^{-1}) with isocratic elution of 60% CH_3CN-H_2O containing 0.1% formic acid to yield **15** (t_R: 35.8 min, 10.1 mg) and **14** (t_R: 67.5 min, 50.3 mg).

Purification process for **16**

The mycelia and culture medium extract (2.3 g) from 2 L culture of *A. oryzae* NSAR1 harboring *helA*, *helB1*, *helB2*, *helD2* and *helB4* was subjected to silica gel column chromatography with stepwise elution of petroleum ether and EtOAc (100:20 and 0:100, v/v) to yield 2 fractions. Fraction 2 (382.5 mg) was further subjected to MPLC on ODS column chromatography with stepwise elution of MeOH and H_2O (4:6, 8:2 and 10:0, v/v) to yield 3 subfractions. Subfraction 2 (90.5 mg) was further purified by semi-preparative HPLC (YMC-Pack ODS-A column, 3 mL min^{-1}) with isocratic elution of 70% CH_3CN-H_2O containing 0.1% formic acid to yield **16** (t_R: 12.6 min, 18.7 mg).

Purification process for **17** and **18**

The mycelia and culture medium extract (3.0 g) from 2 L culture of *A. oryzae* NSAR1 harboring *helA*, *helB1*, *helC*, *helB2*, *helD2*, *helB4* and *helB3* was subjected to silica gel column chromatography with stepwise elution of petroleum ether and EtOAc (100:20 and 0:100, v/v) to yield 2 fractions. Fraction 2 (413.5 mg) was further subjected to MPLC on ODS column chromatography with stepwise elution of MeOH and H_2O (4:6, 7:3 and 10:0, v/v) to yield 3 subfractions. Subfraction 2 (100.2 mg) was further purified by semi-preparative HPLC (YMC-Pack ODS-A column, 3 mL min^{-1}) with isocratic elution of 50% CH_3CN-H_2O

containing 0.1% formic acid to yield **18** (t_R: 18.5 min, 9.3 mg) and **17** (t_R: 39.6 min, 21.5 mg).

Purification process for **19**

The mycelia and culture medium extract (3.1 g) from 2 L culture of *A. oryzae* NSAR1 harboring *helA*, *helB1*, *helC*, *helB2*, *helD2*, *helB4* and *helE* was subjected to silica gel column chromatography with stepwise elution of petroleum ether and EtOAc (100:20 and 0:100, v/v) to yield 2 fractions. Fraction 2 (322.4 mg) was subjected to MPLC on ODS column chromatography with stepwise elution of MeOH and H_2O (5:5, 8.5:1.5 and 10:0, v/v) to yield 3 subfractions. Subfraction 2 (87.9 mg) was further purified by semi-preparative HPLC (YMC-Pack ODS-A column, 3 mL min^{-1}) with isocratic elution of 70% CH_3CN-H_2O containing 0.1% formic acid to yield **19** (t_R: 27.1 min, 15.0 mg).

Purification process for **20** and **21**

The mycelia and culture medium extract (2.0 g) from 2 L culture of *A. oryzae* NSAR1 with *helA*, *helB1*, *helC*, *helB2*, *helD2*, *helB4*, *helB3* and *helD1* was subjected to silica gel column chromatography with stepwise elution of petroleum ether and EtOAc (100:20 and 0:100, v/v) to yield 2 fractions. Fraction 2 (210.0 mg) was subjected to MPLC on ODS column chromatography with stepwise elution of MeOH and H_2O (5:5, 8.5:1.5 and 10:0, v/v) to yield 3 subfractions. Subfraction 2 (81.3 mg) was further purified by semi-preparative HPLC (YMC-Pack ODS-A column, 3 mL min^{-1}) with isocratic elution of 50% CH_3CN-H_2O containing 0.1% formic acid to yield **21** (t_R: 30.6 min, 6.3 mg) and **20** (t_R: 59.1 min, 18.9 mg).

Purification process for **22**

The mycelia and culture medium extract (3.9 g) from 2 L culture of *A. oryzae* NSAR1 with *helA*, *helB1*, *helC*, *helB2*, *helD2*, *helB4*, *helB3* and *helE* was subjected to silica gel column chromatography with stepwise elution of petroleum ether and EtOAc (100:20 and 0:100, v/v) to yield 2 fractions. Fraction 2 (267.3 mg) was subjected to MPLC on ODS column chromatography with stepwise elution of MeOH and H_2O (5:5, 8.5:1.5 and 10:0, v/v) to yield 3 subfractions. Subfraction 2 (97.3 mg) was further purified by semi-preparative HPLC (YMC-Pack ODS-A column, 3 mL min^{-1}) with isocratic elution of 50% CH_3CN-H_2O containing 0.1% formic acid to yield **22** (t_R: 36.0 min, 15.9 mg).

Supplementary References

1. Fujii, T., Yamaoka, H., Gomi, K., Kitamoto, K. & Kumaga, C. Cloning and nucleotide sequence of the ribonuclease T_1 gene (*rntA*) from *Aspergillus oryzae* and its expression in *Saccharomyces cerevisiae* and *Aspergillus oryzae*. *Biosci. Biotechnol. Biochem.* **59**, 1869−1874 (1995).

2. Yamada, O. *et al*. *dffA* gene from *Aspergillus oryzae* encodes L-ornithine N^5-oxygenase and is indispensable for deferriferrichrysin biosynthesis. *J. Biosci. Bioeng.* **95**, 82-88 (2003).

3. Jin F. J., Maruyama, J., Juvvadi, P. R., Arioka, M. & Kitamoto, K. Adenine auxotrophic mutants of *Aspergillus oryzae*: development of a novel transformation system with triple auxotrophic hosts. *Biosci. Biotechnol. Biochem.* **68**, 656-662 (2004).

4. Jin, F. J., Maruyama, J., Juvvadi, P. R., Arioka, M. & Kitamoto, K. Development of a novel quadruple auxotrophic host transformation system by *argB* gene disruption using *adeA* gene and exploiting adenine auxotrophy in *Aspergillus oryzae*. *FEMS Microbiol. Lett.* **239**, 79-85 (2004).

5. Fujimoto, H., Negishi, E., Yamaguchi, K., Nishi, N. & Yamazaki, M. Isolation of new tremorgenic metabolites from an Ascomycete, *Corynascus setosus*. *Chem. Pharm. Bull.* **44,** 1843-1848 (1996).

6. Lodeiro, S. *et al*. Protostadienol biosynthesis and metabolism in the pathogenic fungus *Aspergillus fumigatus*. *Org. Lett.* **11**, 1241-1244 (2009).

7. Mitsuguchi, H., Seshime, Y., Fujii, I., Shibuya, M., Ebizuka, Y. & Kushiro, T. Biosynthesis of steroidal antibiotic fusidanes: functional analysis of oxidosqualene cyclase and subsequent tailoring enzymes from *Aspergillus fumigatus*. *J. Am. Chem. Soc.* **131**, 6402-6411 (2009).

8. von Daehne, W. & Godtfredsen, W. O. Tetracyclic triterpenes as cholesterol-lowering and anti-atherosclerosis agents. US Patent No. 6,177,418, 2001.

9. Lee, S.-Y., Kinoshita, H., Ihara, F., Igarashi, Y. & Nihira, T. Identification of novel derivative of helvolic acid from *Metarhizium anisopliae* grown in medium with insect component. *J. Biosci. and Bioeng.* **105**, 476-480 (2008).

10. Okuda, S., Sato, Y., Hattori, T. & Wakabayashi, M. Isolation and structural elucidation of 3-oxo-16*β*-acetoxyfusida-1,17(20)(16,21-cis),24-trien-21-oic acid. *Tetrahedron Lett.* **47**, 4847-4850 (1968).
